# Carbene Analogues of Group 15: Reduction of s‑Hydrindacene-Based Chloropnictogenium Ions To Access an Antimony Hydride Monocation and a Trinuclear Bismuth Dication

**DOI:** 10.1021/jacs.6c07386

**Published:** 2026-07-03

**Authors:** Marvin Janssen, Marian Olaru, Enno Lork, Jens Beckmann, Emanuel Hupf

**Affiliations:** † Institute for Inorganic Chemistry and Crystallography, 9168 University of Bremen, D-28359 Bremen, Germany

## Abstract

We report the synthesis of three chloropnictogenium cations [M^S^FluindECl]^+^ (**2As**, **2Sb** and **2 Bi**), which unravel different reaction pathways toward Et_3_SiH, leading to the formation of the neutral arylarsenic­(III) dihydride M^S^FluindAsH_2_ (**3**), the cationic arylhydridostibenium­(III) [M^S^FluindSbH]^+^ (**4**) and the dicationic aryltribismuth­(I) ion [M^S^FluindBi_3_]^2+^ (**7**). In light of its reactivity, **4** can also be regarded as a protonated stibinidene­(I), as demonstrated by the reaction with trimethylindium and gallium as well as diphenyldichalcogenides, leading to the stibinidene supported dimethylelement cations [M^S^FluindSbEMe_2_]^+^ (**5Ga**, **5In**) and the chalcogenide cations [M^S^FluindSbChPh]^+^ (**6S**, **6Se**, **6Te**) featuring formal SbCh double bonds. The Bi_3_ dication **7** reveals substantial 2-electron-3-center bonding character within the Bi–C–Bi interaction, a feature unprecedented in the chemistry of group 15 elements.

## Graphical abstract

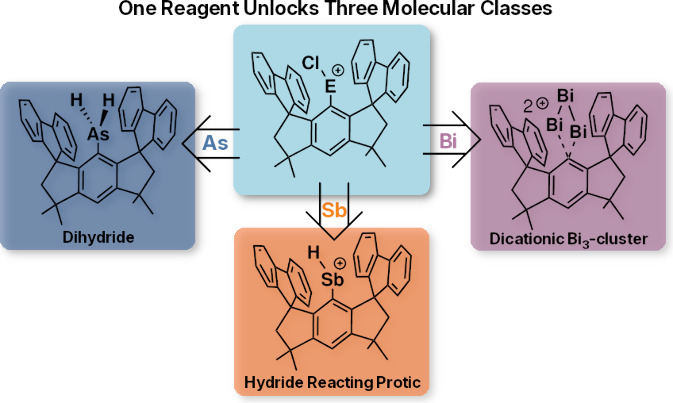

## Introduction

Diarylpnictogenium ions [R_2_E]^+^ (**IE**) and neutral arylpnictinidenes [RE] (**IIE**) (E = N, P, As, Sb, Bi) are fiercely reactive, Lewis amphoteric carbene analogues of group 15.^1^ While most previously prepared members of these compound classes were *electronically* stabilized by electron rich ligands or substituents, very recently, the first *kinetically* stabilized examples were isolated with the aid of bulky and rigid s-hydrindacene-based M^S^Fluind scaffolds (M^S^Fluind = dispiro­[fluorene-9,3′-(1′,1′,7′,7′-tetramethyl-s-hydrindacen-4′-yl)-5′,9′′-fluorene]). In 2021 and 2023, our group reported an almost complete series of pnictogenium ions [M^S^FluindEMes]^+^ (**IE**, E = P, As,^2^ Sb, Bi^3^), which are characterized by two aryl substituents, an electron lone pair, a vacant p-orbital, and a positive charge along with an electronic singlet ground state. Due to the positive charge they are sufficiently more electron deficient compared to the isoelectronic neutral group 14 carbene analogues. Thus, the phosphenium ion, [M^S^FluindPMes]^+^, turned out to be a strong Lewis superacid.^2^ In 2023, Cornella’s group described the reduction of M^S^Fluind^*t*Bu^BiCl_2_ (M^S^Fluind decorated by *t*-Bu groups), which provided the triplet bismuthinidene M^S^Fluind^*t*Bu^Bi (**IIBi**).^4^ Concomitant efforts by the Tan group to reduce M^S^Fluind^*t*Bu^BiCl_2_ and M^S^Fluind*BiCl_2_ (M^S^Fluind* decorated by Me_2_CCH_2_CH_2_CMe_2_ groups) afforded also the triplet bismuthinidenes M^S^Fluind^*t*Bu^Bi and M^S^Fluind*Bi (**IIBi**).^5^ Due to the lower steric demand, the reduction of the parent M^S^FluindBiCl_2_ gave the related dibismuthene M^S^FluindBiBiM^S^Fluind (**IIIBi**) instead.^4^ The reduction of M^S^Fluind*SbCl_2_ produced the triplet stibinidene M^S^Fluind*Sb,^6^ whereas the less bulky substrates let to the distibenes M^S^Fluind^*t*Bu^SbSbM^S^Fluind^*t*Bu^ and M^S^FluindSbSbM^S^Fluind (**IIISb**).^7^

In 2024, the Tan group attempted the preparation of a triplet phosphinidene, M^S^Fluind^*t*Bu^P, but instead obtained a singlet phosphanorcaradiene of the same composition.^8^

In 2024 and 2025, we and the Tan group prepared triplet nitrenes M^S^FluindN^9^ and M^S^Fluind*N^10^ (**IIN**) by the photolysis of the related azides M^S^FluindN_3_ and M^S^Fluind*N_3_. Besides the kinetic stabilization, nitrenes are also electronically stabilized by spin delocalization across the central aryl ring that is absent in the heavier pnictinidenes. In 2025, Cornella’s group reported the adventitious protolysis of bismuthinidene M^S^Fluind^*t*Bu^Bi in the presence of the Lewis acid B­(C_6_F_5_)_3_, which proceeded with partial cleavage of M^S^Fluind^*t*Bu^ groups giving rise to the formation of an allyl type cation [M^S^Fluind^*t*Bu^BiBiBiM^S^Fluind^*t*Bu^]^+^(**IV**, Scheme 1).^11^

**Scheme 1:**
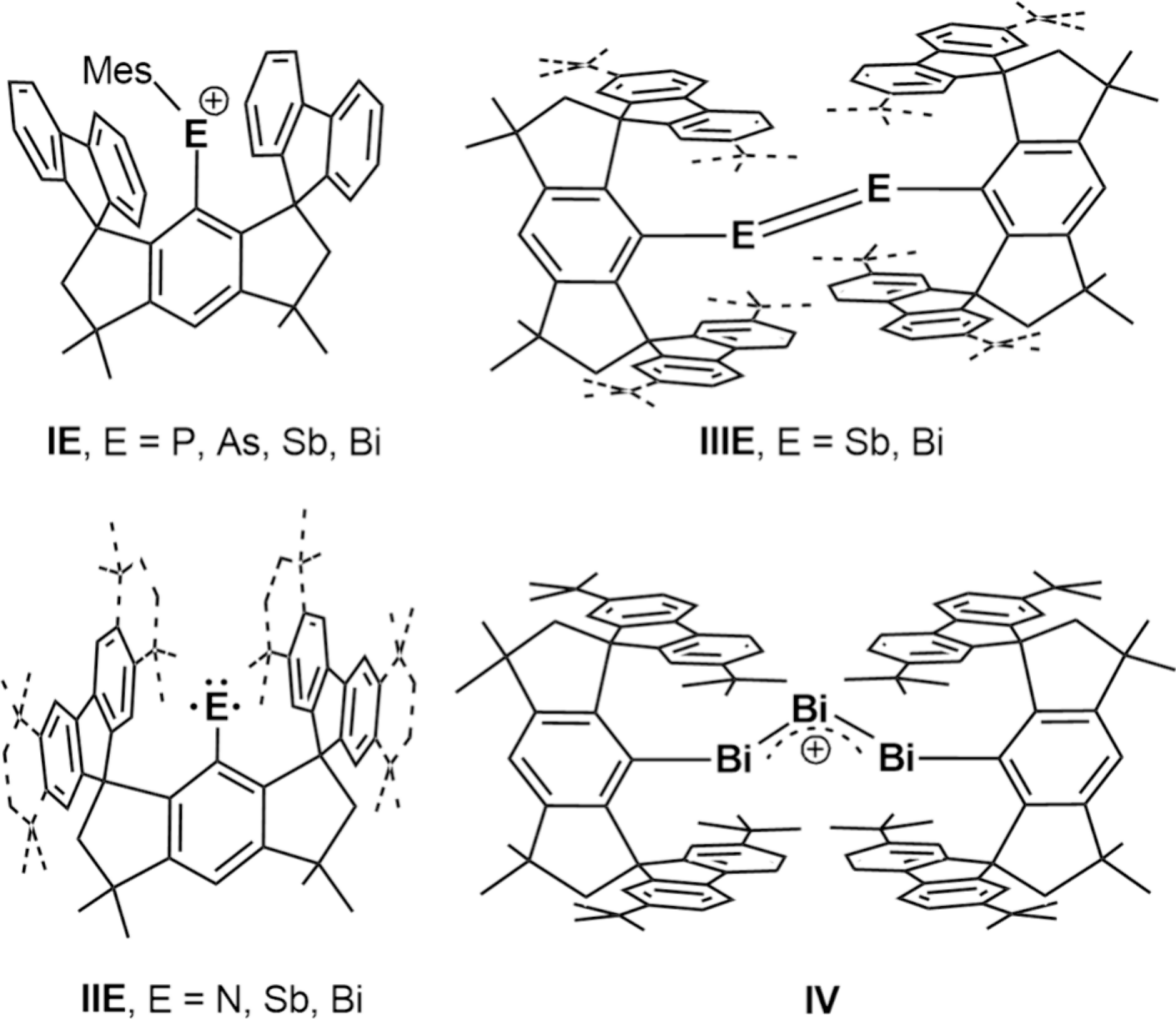
Recent Examples of Pnictogenium Ions [R_2_E**]** ^+^ (**IE**), Neutral Pnictinidenes [RE] (**IIE**), Dimeric Dipnictogenes **IIIE**, as well as a Trimetallic Bi_3_ Allyl Cation **IV**

In this work, we describe M^S^Fluind-based chloropnictogenium ions [M^S^FluindECl]^+^ (E = As, Sb, Bi), which are the link between both types of group 15 carbene analogues **IE** and **IIE**. Subsequent treatment with Et_3_SiH led to distinctively different substance classes for each pnictogen.

## Results and Discussion

Chloride abstraction of the arylpnictogen dichlorides M^s^FluindECl_2_ (**1As**, E = As;^12^ **1Sb**, E = Sb;^7^ **1Bi**, E = Bi;^4^ M^S^Fluind = dispiro­[fluorene-9,3′-(1′,1′,7′,7′-tetramethyl-s-hydrindacen-4′-yl)-5′,9″-fluorene]) using KB­(C_6_F_5_)_4_ gave rise to the respective chloropnictogenium species [M^s^FluindECl]^+^ (**2As**, **2Sb** and **2Bi**) as dark red (**1As**) and dark orange (**2Sb** and **2Bi**) solids in yields of 64%, 74% and 68%, respectively (Scheme 2). The same procedure failed for M^s^FluindPCl_2_ (**1P**),^12^ although the [M^s^FluindPCl]^+^ ion was detected by ESI MS. Under inert conditions, the chloropnictogenium cations **2As**, **2Sb** and **2Bi** are reasonable stable both in solution at room temperature (i.e., several weeks in CH_2_Cl_2_) as well as in the solid state (several months) based on ^1^H NMR data.

**Scheme 2:**
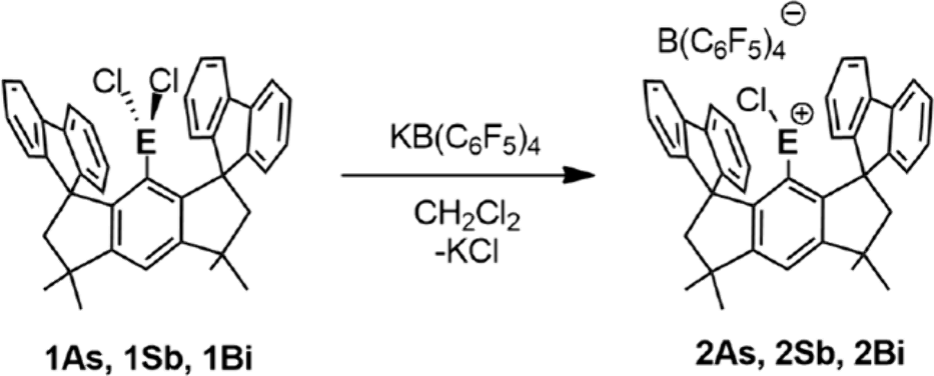
Synthesis of Chloropnictogenium Cations **2As**, **2Sb**, and **2Bi**

The UV–vis spectrum of **2E** featuring absorption maxima at 490 nm (**2As**), 417 nm (**2Sb**) and 430 nm (**2Bi**) consistent with the observed red and orange colors () and the molecular structures are visualized in Figure 1. The spatial arrangement of the pnictogen atoms of **2E** are trigonal planar upon inclusion of the electron lone pair. The E–Cl bond distances increase from **2As** (2.1615(6) Å) over **2Sb** (2.3528(6) Å) to **2Bi** (2.473(2) Å) and is in case of **2As** smaller compared to the As–Cl bond lengths in **1As** (2.1778(6) and 2.2195(6) Å) and in case of **2Sb** and **2Bi** comparable to **1Sb** (2.3536(4) to 2.362(10) Å) as well as **1Bi** (2.458(12) to 2.4877(8) Å), respectively. The E–C bond length of 1.935(3) Å (**2As**), 2.132­(3) Å (**2Sb**) and 2.254(7) Å (**2Bi**) are all smaller compared to the dichlorides **1E** ^4,7,12^ and smaller compared to the diarylpnictogenium cations^2,3^ in case of arsenic and antimony and the same within the standard deviation for bismuth. The C–E–Cl angles differ less ranging from 96.29(7)° (**2Sb**) to 97.03(6)° (**2As**). The Lewis acidity of **2As**, **2Sb** and **2Bi** was investigated using the Gutmann Beckett method by monitoring the change in the ^31^P NMR chemical shift of Et_3_PO upon coordination with a Lewis acid^13^ and revealed acceptor numbers of 130 (**2As**), 111 (**2Sb**) and 71 (**2Bi**). This trend suggests that the elusive **2P** would have an even higher Lewis acidity, which is presumably the reason why the chloride abstraction from M^s^FluindPCl_2_ (**1P**) failed. The chloropnictogenium cations **2E** are thus significantly more Lewis acidic than the related diarylpnictogenium cations **IE** (132 for **IP** to 22 for **IBi**)^2,3^ and (except for **2Bi**) also than the strong Lewis acids B­(C_6_F_5_)_3_ (AN = 82) or [Et_3_Si · C_6_D_6_]^+^ (AN = 105).^13^

**Figure 1:**
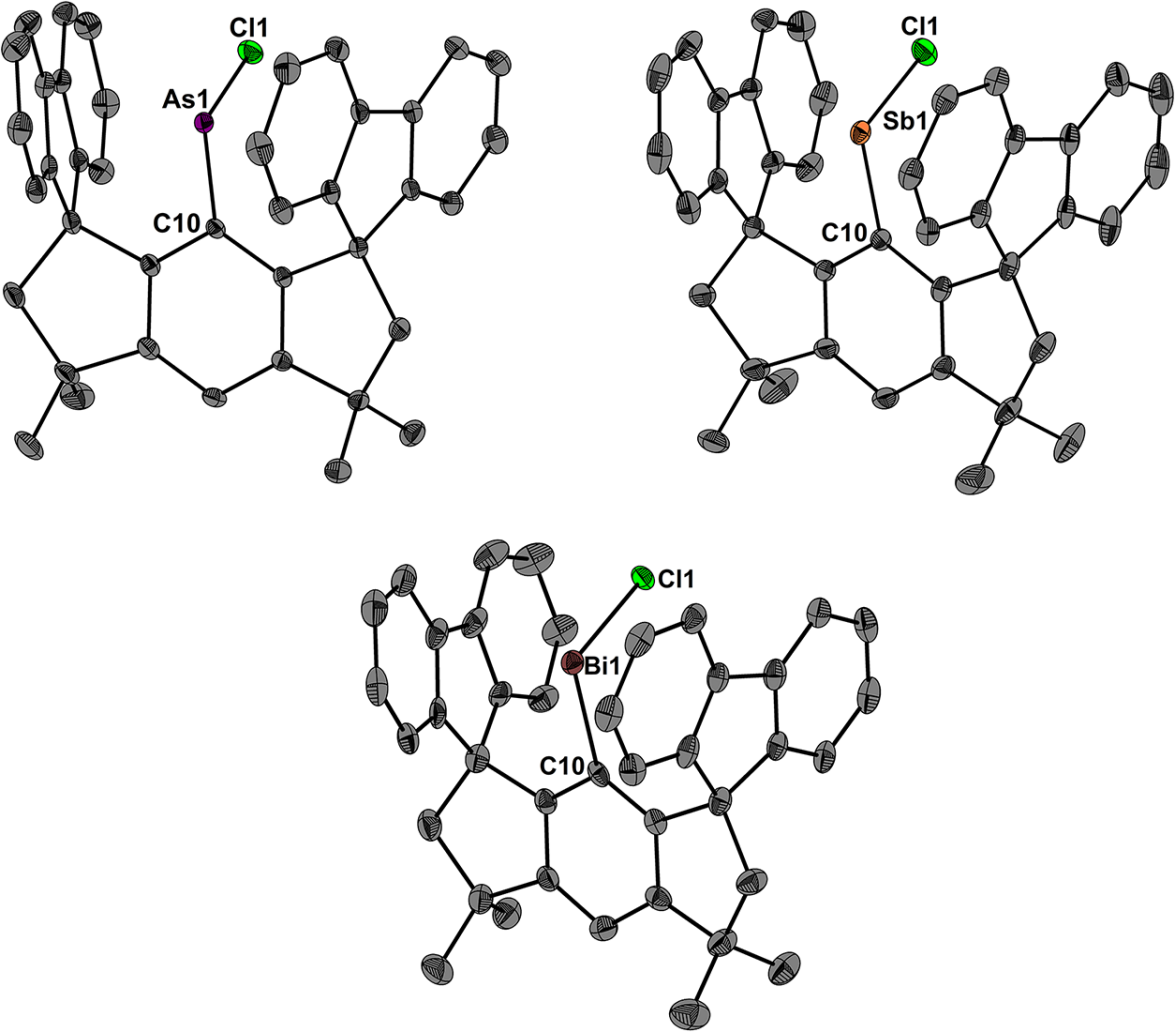
Molecular structures of [M^S^FluindECl]^+^ (**2As**, E = As; **2Sb**, E = Sb; **2Bi**, E = Bi) showing 50% probability ellipsoids and the essential atomic numbering scheme. The [B­(C_6_F_5_)_4_]^−^ anions as well as H atoms are omitted for clarity. Selected bond lengths [Å] and angles [°] for **2As**: As1–Cl1, 2.1615(6); As1–C10, 1.935(3); C10–As1–Cl1, 97.03(6); **2Sb**: Sb1–Cl1, 2.3528(6); Sb1–C10, 2.132(3); C10–Sb1–Cl1, 96.29(7); **2Bi**: Bi1–Cl1, 2.473(2); Bi1–C10, 2.254(7); C10–Bi1–Cl1, 96.7(2).

Surprisingly, all three chloropnictogenium cations show different reactivity toward triethylsilane, which was used as a mild, less basic hydride transfer reagent to abstract chloride. For **2As**, treatment with Et_3_SiH in a 1:1 molar ratio gave rise to the arylarsenic dihydride M^S^FluindAsH_2_ (**3**) as colorless crystals in 46% yield along with the formation of triethylchlorosilane, whereas as the reaction of **2Sb** led to Cl/H exchange and produced the hydridostibenium cation [M^S^FluindSbH]^+^ (**4**) as yellow crystalline solid in 70% (Scheme 3). The hydrogen attached to antimony give rise to a notable downfield chemical shift of 18.06 ppm hinting toward a rather protic character, despite being formally hydridic. We speculate that an analogous hydridoarsenium species forms as transient intermediate also in the formation of **3**, however, all attempts to detect such species failed, e.g., via a similarly downfield shifted resonance in the ^1^H NMR. Surprisingly, increasing the amount of Et_3_SiH did not lead to higher yields of **3**. However, an alternative synthesis of **3** could be realized by the reaction of **1As** with an excess of LiAlH_4_, providing **3** in 57% yield. The molecular structures of **3** and **4** are depicted in Figure 2. The As–C bond of 1.973(3)/1.969(7) Å in **3** as well as Sb–C bond of 2.165(3) Å in **4** are comparable to the respective bonds in **2As** and **2Sb**. Notably, **3** represents the lighter homologue of the recently reported arylbismuth dihydride M^S^Fluind^*t*Bu^BiH_2_ by Cornella et al.^14^

**Scheme 3:**
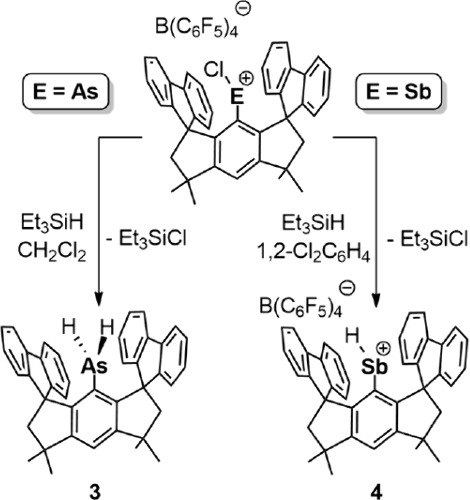
Synthesis of Arylarsenic Dihydride **3** and Hydridostibenium Cation **4**

**Figure 2:**
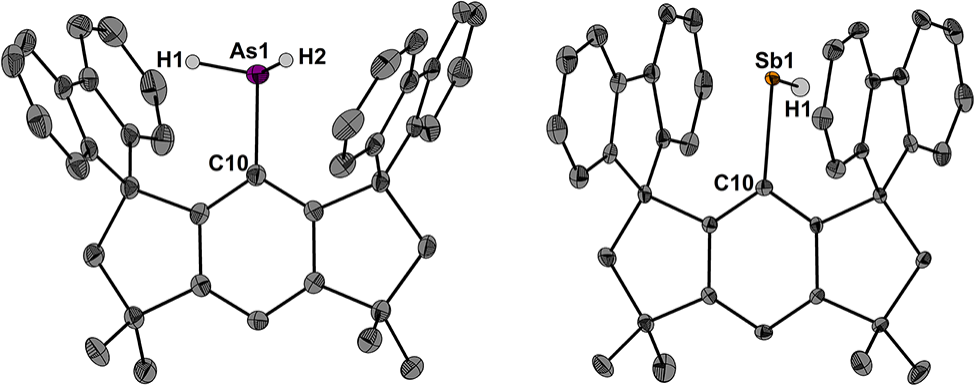
Molecular structure of [M^S^FluindAsH_2_] (**3**) and [M^S^FluindSbH]^+^ (**4**) showing 50% probability ellipsoids. The [B­(C_6_F_5_)_4_]^−^ anions as well as H atoms are omitted for clarity.

In an effort to explore the reactivity of **4**, it was reacted with Me_3_E (E = Ga, In). The reaction with Me_3_In indicated complete consumption of **4**, resulting into the donor–acceptor complex **5In** under the liberation of methane (Scheme 4). The molecular structure of **5In** shows a rather long Sb–In bond length of 2.9539(5) Å, which allows for the interpretation of this interaction as a stibinidene supported indinium Me_2_In^+^ cation; however, the C1–In1–C2 angle of 152.4(2)° deviates substantially from the putative linear arrangement of the free indinium cation making it a stibenium cation (Figure 3). We recently introduced the first stable diarylindium cation with an almost linear C–In–C arrangement of 176.2° and shorter In–C bond lengths.^15^

**Scheme 4:**
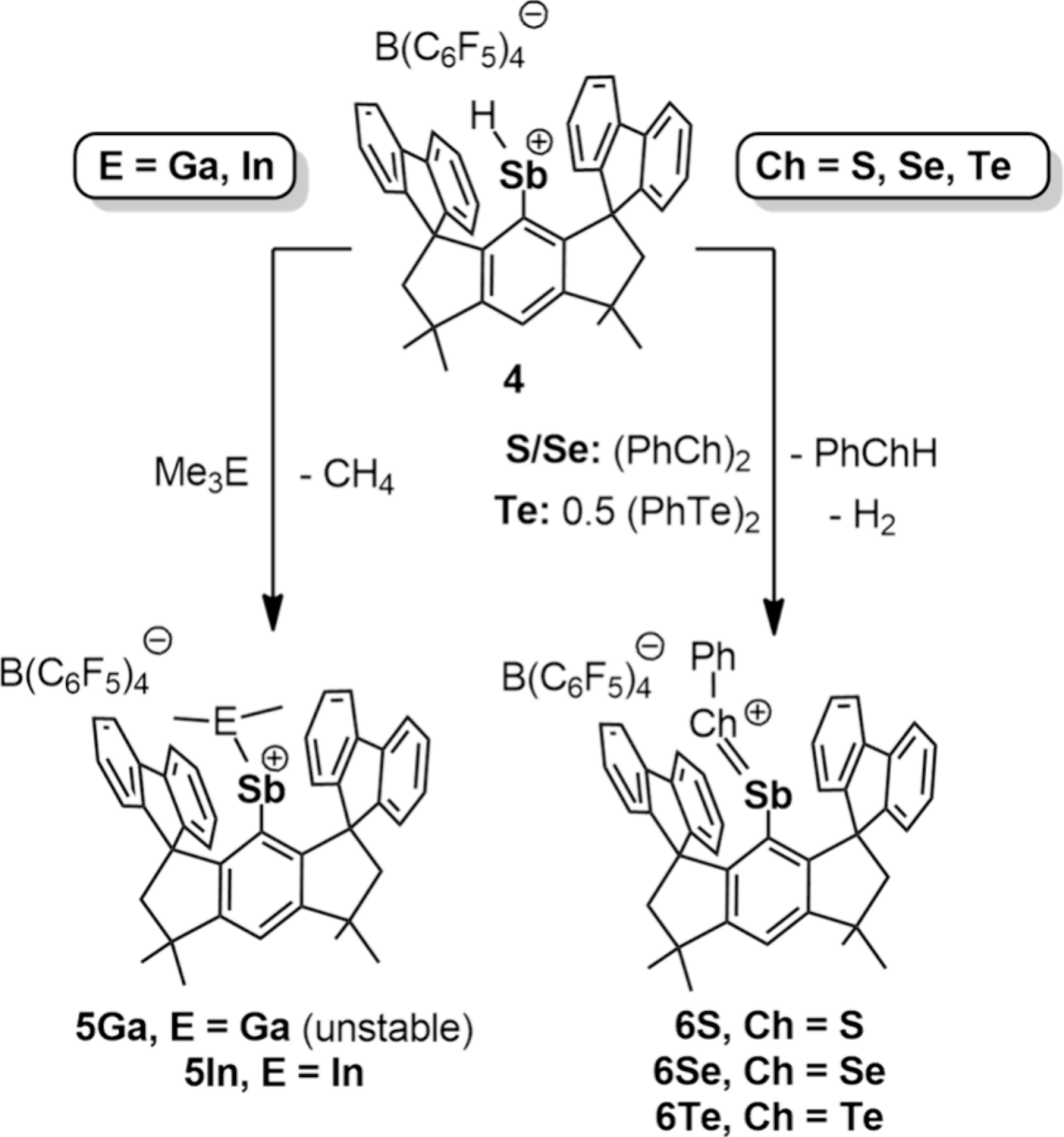
Reactivity of Hydridostibenium Cation **4** towards Me_3_E (E = Ga, In) and PhChChPh (Ch = S, Se, Te)

**Figure 3:**
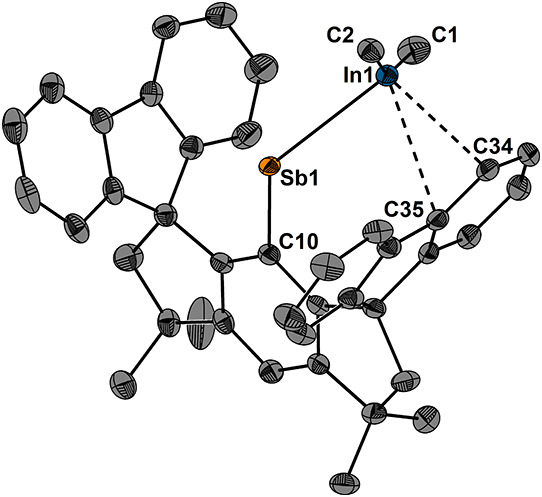
Molecular structure of [M^S^FluindSbInMe_2_]^+^ (**5In**) showing 50% probability ellipsoids and the essential atomic numbering scheme. The [B­(C_6_F_5_)_4_]^−^ anions as well as H atoms are omitted for clarity. Selected bond lengths [Å] and angles [°]: Sb1–In,1 2.9535(5); Sb1–C10, 2.145(4); In1–C1, 2.119(4); In1–C2, 2.128(4); C10–Sb1–In1, 125.77(8); C1–In1–C2, 152.4(2).

For the reaction of **4** with Me_3_Ga, similar crude ^1^H NMR and ^13^C NMR spectra were obtained (see ), but no defined product could be isolated. We assume that a similar species **5Ga** forms, which, however, is too reactive to be isolated.

The reaction of **4** with the diphenyldichalcogenides (PhCh)_2_ (Ch = S, Se, Te) led to the formation of the doubled bonded SbCh species [M^S^FluindSbChPh]^+^ (**6Ch**, Ch = S, Se, Te) and elimination of the thiophenol, selenol, and in the case of tellurium, hydrogen gas (due to the instability of the tellurol) as side products (Scheme 4).

The molecular structures of **6Ch** are depicted in Figure 4 and reveal increasing Sb–Ch bonds lengths of 2.3536(4) Å (**6S**), 2.4721­(4) Å (**6Se**) and 2.6728(5) Å (**6Te**), respectively, and can be regarded as formal double bonds with the formal positive charge located on the chalcogenide. Notably, **6Te** is isoelectronic to the dimeric distibenes RSbSbR species **IIISb** reported by the groups of Cornella and Tan with the Sb–Te bond length being identical (within standard deviation) with the Sb–Sb bond of the smaller distibene M^S^FluindSbSbM^S^Fluind (2.6731(3) Å),^7^ but being smaller compared to the larger M^S^Fluind^*t*Bu^Sb-SbM^S^Fluind^*t*Bu^ of 2.7046(4) Å and 2.7220(6) Å.^6,7^

**Figure 4:**
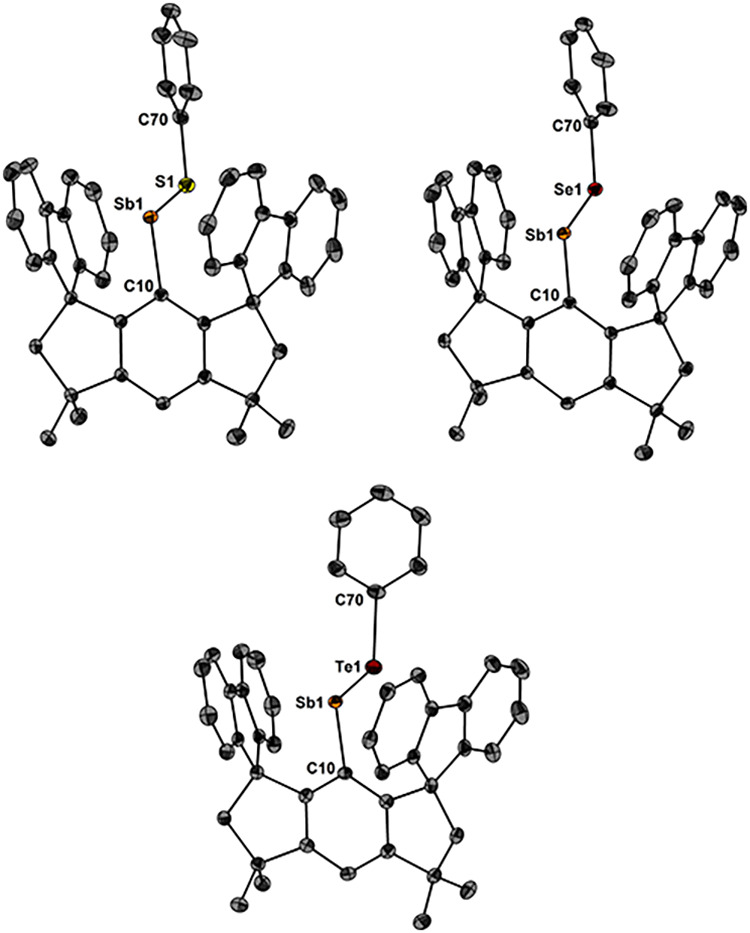
Molecular structure of [M^S^FluindChPh]^+^ (**6Ch**). (**6S**, Ch = S; **6Se**, Ch = Se; **6Te**, Ch = Te) showing 50% probability ellipsoids and the essential atomic numbering scheme. The [B­(C_6_F_5_)_4_]^−^ anions as well as H atoms are omitted for clarity. Selected bond lengths [Å] and angles [°] for **6S**: Sb1–S1, 2.3536(4); Sb1–C10, 2.134(2); S1–C70, 1.782(2); C10–Sb1–S1, 91.55(4); C70–S1–Sb1, 102.57(5); **6Se**: Sb1–Se1, 2.4721(4); Sb1–C10, 2.138(2); Se1–C70, 1.925(2); C10–Sb1–Se1, 90.86(4); C70–Se1–Sb1, 99.51(5); **6Te**: Sb1–Te1, 2.6728(5); Sb1–C10, 2.147(3); Te1–C70, 2.113(2); C10–Sb1–Te1, 97.03(6); C70–Te1–Sb1, 99.12(6).

The bonding situation of the Sb–In and Sb–Ch has been computationally examined by a complementary bonding analysis.^16^ In the framework of the electron density based AIM methodology,^17^ the Sb–In interaction is of weak, ionic character as judged by the bcp parameters, such as the electron density (ρ_bcp_, 0.34 e Å^–3^), Laplacian (∇^2^ρ_bcp_, 0.4 e Å^–5^) and kinetic as well as total energy over electron density ratios (G/ρ_bcp_, 0.34 au; H/ρ_bcp_, −0.27 au, ). This bonding picture is augmented by the Non-Covalent Interaction (NCI) Index,^18^ which shows a large disk-shape area between In and Sb, indicating vanishing covalent contributions (). The Wiberg Bond (WBI) as well as Delocalization indices (δ) of both 0.64 () also indicates a rather weak Sb–In interaction, however, the σ-bonding is the dominating part of the highest occupied molecular orbital (HOMO, Figure 5).

**Figure 5:**
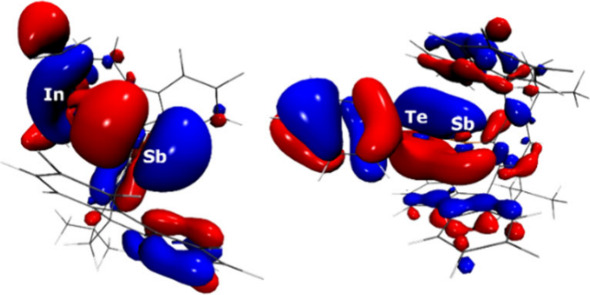
Respective HOMO of **5In** (left) and **6Te** (right) at iso-surfaces at *s*(*r*) = ±0.02 (blue/red).

For the SbCh bonding in compounds **6Ch** two main resonance structures can be formulated with either a Sb=Ch double bond (positive formal charge on the chalcogen) or as a Sb–Ch single bond (with a formally positively charged Sb). This bonding scenario is reminiscent of a series of pincer stabilized formal heavy pnictogen-chalcogen double bonds of type 2,6-(Ph_2_PNMes)_2_C_6_H_3_SbCh, which are better described as polarized single bonds of type ^+^Pn–Ch^–^.^19^ The Wiberg Bond indices of 1.09 (**6S**), 1.22 (**6Se**) and 1.38 (**6Te**) show a larger variation compared to the pincer systems 2,6-(Ph_2_PNMes)_2_C_6_H_3_SbCh (1.19–1.21),^19^ with lower SbS and higher SbTe double bond character (). However, all SbCh WBIs are lower compared to recently reported kinetically stabilized M^S^Fluind*SbCh species (1.69–1.82).^20^ In agreement with the bond indices, NLMO analysis^21^ revealed that one lone pair of each chalcogen showed reduced percentages of the parent lone pair NBO of 83% (S), 81% (Se) and 77% (Te), demonstrating partial delocalization to the Sb atom, which makes up 37 to 38% of the total NLMO bond orders of the SbCh bond (). The π-backdonation from the chalcogen is also visible within the HOMO and exemplarily visualized for **6Te** in Figure 5. Despite the partial double bond character of the SbCh bonds, the AIM derived electron density at the bond critical point are fairly low (0.66 to 0.53 e Å^–3^), but are accompanied by large ellipticities reflecting substantial electron smearing.

Yet another reactivity of the chloropnictogenium cations was observed, when **2Bi** was treated with triethylsilane, giving rise to a significant color change from deep red to a strongly dark brown mixture within 2 h. Layering the reaction mixture with *n*-hexane at room temperature, produced the dicationic trinuclear bismuth compound [M^S^FluindBi_3_]^2+^ (**7**) that was isolated as a dark brown, crystalline and air-sensitive solid in 17% yield (Scheme 5). The hydrocarbon M^S^FluindH is formed as a byproduct of the reaction as confirmed by NMR spectroscopy (Scheme 5). The formation of **7** can be rationalized under the assumption that [M^S^FluindBiH]^+^ formed first, which subsequently underwent immediate autoprotolysis to **7**, M^S^FluindH and H_2_. In chlorinated solvents, such as dichloromethane-*d*_2_ and 1,2-dichlorobenzene-*d*_4_, **7** could be characterized by ^1^H and ^13^C NMR spectroscopy, both spectra consist of sharp signals indicative of a singlet electronic ground state. In the solid state, **7** is stable under an inert atmosphere and can be stored for months, but shows slow decomposition in solutions, i.e., the chlorobismuthenium cation [M^S^FluindBiCl]^+^ (**2Bi**) is formed in dichloromethane and instable Bi­(I) ions that eventually deposit bismuth black, which can be significantly accelerated upon heating at 80 °C (). The UV–vis spectrum of **7** shows a set of absorption maxima at 730, 550, 460, and 410 nm (). The recently reported trimetallic Bi(1) allyl cation **IV** by Cornella and co-workers revealed weak absorption bands between 830 and 1250 nm, which resulted from mixed singlet–triplet states and could only be modeled upon inclusion of spin–orbit coupling (SOC).^11^ Similarly, our time-dependent density functional theory (TD-DFT) computations for **7** without SOC could not model the experimental absorption at 730 nm (). Applying the reported settings by Neese and Cornella and their teams,^11^ such as the two-component relativistic Hamiltonian (X2C),^22^ spin–orbit mean-field operator,^23^ finite nucleus,^24,25^ picture change corrections^26^ as well as incorporating the quasi-degenerate perturbation theory in the TD-DFT section^27^ resulted in strong singlet–triplet mixing of these SOC states, increasing oscillator strengths for the absorption between 700 and 900 nm ( and ).

**Scheme 5:**
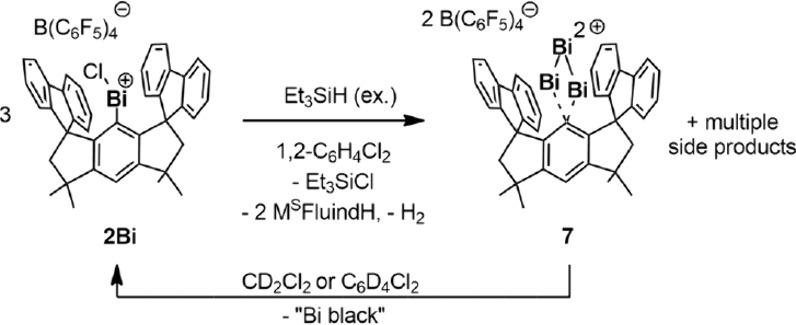
Synthesis of Trinuclear Bi_3_ Dication **7**

The molecular structure of **7** reveals the dicationic nature with a clear separation between the dication and the two weekly coordinating borate anions. The central building block of the dication consists of three bismuth­(I) atoms, which, together with the *ipso*-carbon of the s-hydrindacene based framework, form a 4-membered heterocycle (Figure 6). The central trimetallic Bi building block in **7** shows a Bi1–Bi3 distance of 2.9647(4) Å/2.9303(8) Å (two independent molecules in the asymmetric unit), which is comparable to previously described Bi–Bi single bond lengths.^28^ In contrast, the Bi3–Bi2 distance in **7** (2.8678(7) Å/2.8567(6) Å) is about 3% shorter than Bi1–Bi3 and thus more comparable to bond lengths reported for Bi=Bi double bonds.^4a,29^ The distance of the two outermost Bi atoms Bi1–Bi2 (3.5137(5) Å/3.4828(5) Å) is significantly larger than the respective Bi1–Bi3 and Bi3–Bi2 bond lengths. The average Bi1–Bi3 and Bi3–Bi2 bond lengths in **7** (2.9049 Å) are similar to the averaged Bi–Bi bond lengths of the trimetallic Bi(1) allyl cation **IV** (2.932 Å).^11^ Additionally, the Bi1–Bi3–Bi2 bond angle in **7** (74.066(14)°/ 73.989(18)°) is around 6° smaller and thus significantly more compressed than the corresponding angle in the Bi based allyl cation (80.637(3)°).^11^ Interestingly, the C–Bi bond lengths found in **7** (C10–Bi1, 2.367(12) Å/2.392(12) Å; C10–Bi2, 2.603(8) Å/2.682(13) Å) are considerably larger than the C–Bi distances observed for the chlorobismuthenium cation **2Bi** (2.247(2) Å), **IV** (2.2833(16) Å and 2.2869(16) Å)^11^ as well as previously reported C–Bi single bonds.^3,4a,5,30^ In addition, the C–C bond lengths within the central phenyl ring of the s-hydrindacene framework of **7** have marginal quinoid character (*Q* = 0.01325/0.00025, )^31^ and thus the aromatic system is mostly preserved. This is further corroborated by the C15–C10–C11 bond angle of 117.2(8)°/120.0(11)°) indicating intact sp^2^ hybridization of the *ipso*-substituted carbon atom.

**Figure 6:**
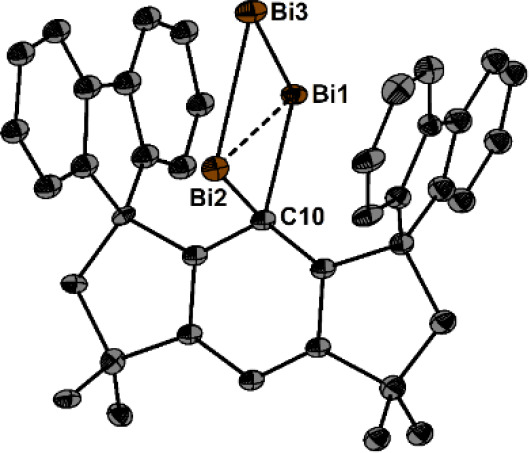
Molecular structure of [M^S^FluindBi_3_]^2+^ (**7**) showing 50% probability ellipsoids and the essential atomic numbering scheme. The [B­(C_6_F_5_)_4_]^−^ anions as well as H atoms are omitted for clarity. Selected bond lengths [Å] and angles [°] for **7** (two independent molecules in the unit cell): C10–Bi1, 2.367(12)/2.392(12); C10–Bi2, 2.603(8)/2.682(13); Bi1–Bi3, 2.9647(4)/2.9303(8); Bi3–Bi2, 2.8678(7)/2.8567(6); Bi1–Bi2, 3.5137(5)/3.4828(5); Bi1–Bi3–Bi2, 74.066(14)/73.989(18); C11–C10–C15, 117.2(8)/120.0(11).

The nature of the bonding situation of the Bi_3_C core within the dicationic species **7** was investigated in detail by a complementary bonding analysis.^16^ First, geometry optimization of the model dication [PhBi_3_]^2+^ led to a different structural motif, with the Bi_3_ core being bent with respect to the *ipso*-C atom, whereas in **7**, the Bi_3_ core and the *ipso*-C atom are within a plane () and highlight the role of dispersion provided by the flanking fluorenyl groups in **7**.

The Bi–Bi–Bi interaction is reminiscent of the bonding situation of Neese’s and Cornella’s allyl cation **IV**. The highest occupied molecular orbital of **7** consists of the delocalized Bi_3_ electrons and is virtually identical to **IV** (Figure 7 and ). Furthermore, the NPA charges^32^ for the bridging bismuth atoms (**7**: + 0.09 e; **IV**: + 0.16 e), the flanking bismuth atoms (**7**: + 0.94/0.82 e; **IV**: + 0.68/0.67 e) as well as the *ipso*-carbons (**7**: −0.57 e; **IV**: −0.34 e) indicate slightly larger charge separation for the dicationic species **7** compared to the monocationic **IV** (). Also, in both cases, the NBO^21^ analysis reveal three bonding orbitals within the Bi_3_ core (). To further facilitate the bonding situation of the Bi_3_ core we extended the bonding analysis to various bonding descriptors not only for the dicationic species **7**, but also for **IV**. NLMO analysis reveals the delocalized nature of the π-electrons as the bonding orbital of one Bi–Bi π-interaction shows only 75% (**7**) and 70% (**IV**) of parent NBO occupation, with contributions of the remaining Bi atom of 22% (**7**) and 28% (**IV**) consistent with the HOMO picture (). Key parameters derived from the electron-density-based AIM^17^ method indicated polar covalent interactions within the Bi_3_ core with slightly positive Laplacians (∇^2^ρ_bcp_) and kinetic energy over electron density ratios (G/ρ_bcp_) along with negative total energy over electron density values (H/ρ_bcp_) (). Within the ELI-D^33^ frame, the disynaptic Bi–Bi bonding basins sum up to 1.97 and 2.52 e for each Bi–Bi interaction in **5** **7** and to 2.40 e each in **IV** (). The NCI^18^ of both compounds shows large donut-shaped areas along the Bi–Bi axes indicative of increased ionicity and augment the AIM and ELI-D approaches ().

**Figure 7:**
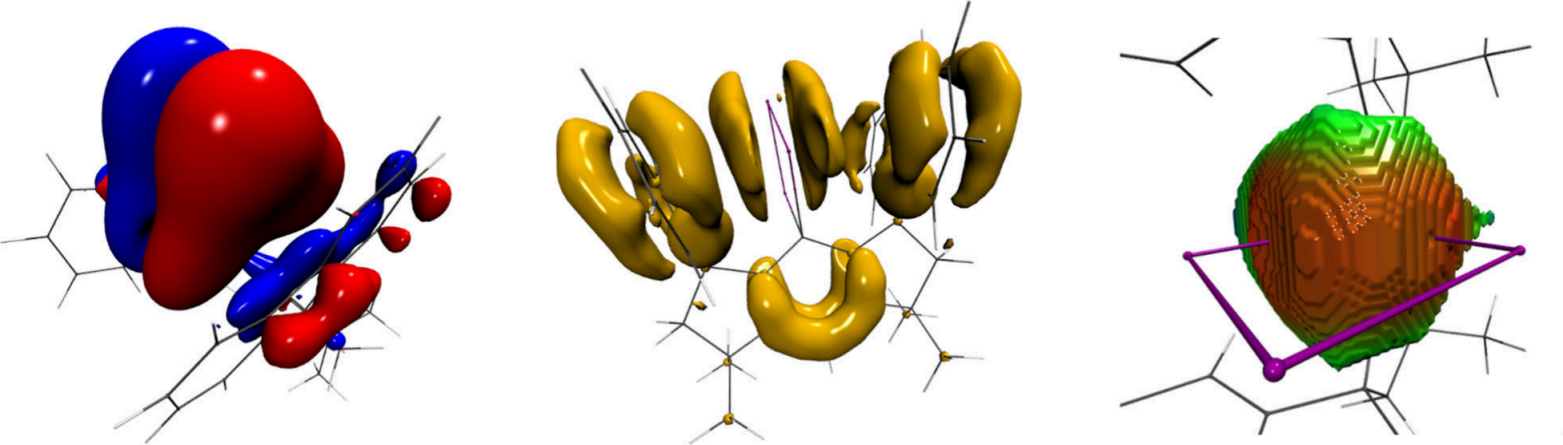
(Left) Highest occupied molecular orbital of **7** at *iso*-surfaces of ±0.02 (blue/red). (Middle) LOL-π of **7** at iso-surfaces of 0.35. (Right) ELI-D distribution (unitless) color-coded onto the ELI-D basin connecting the Bi and the C atoms of **7**, with red indicating higher localizability.

The most striking difference between the dicationic Bi_3_ species **7** and the monocationic **IV** is the Bi–C bonding situation. In **IV**, both terminal Bi atoms are bonded to one C atom each, leading to NBO bonding orbitals with an occupation of 1.95 e each. In contrast neither of the terminal Bi atoms in **7** lead to a NBO bonding orbital, but second order perturbation theory give rise to strong LP_C → LV_Bi donation of E2 = 177 and 44 kcal mol^–1^, respectively (). In agreement with these findings, the NLMO analysis of **7** shows that the Bi atoms contribute to the LP_C with 20.0% and 8.5%, respectively. Interestingly, also the second bonding orbital between the *ipso*- and *ortho*-C atom shows Bi contributions of 5.5% () indicating potential disruption of the central π-conjugation. Within the electron density based ELI-D approach, a disynaptic Bi–C bonding basin with an occupation of 2.04 e is found within **IV**, along with a spherical electron localizability along the Bi–C ( and ). For the dicationic **7**, only a single Bi–C disynaptic ELI basin forms (with a comparable occupation of 2.26 e), but importantly the electron localizability is distributed toward both Bi atoms (Figure 7 and ). This is also reflected in the electron ellipticity at the AIM bond critical points, which are larger for the Bi–C bcps in **7** (0.16 and 0.19) compared to **IV** (0.08). The more negative total energy over electron density values (H/ρ_bcp_) and delocalization indices found for the Bi–C bond in **IV** indicate a higher degree of covalency compared to **7**, which can also be seen from the NCI ( and ). The picture of a more evenly distributed electron density is also supported by the inspection of the integrated gradient model based on the Hirshfeld partitioning (IGMH),^34^ which unravels the interaction between different fragments within a molecule as well as the interaction region indicator (IRI), which is capable of simultaneously uncovering chemical bonds as well as weak noncovalent interactions.^35^ From both methods, the interaction of the *ipso*-C atom toward both Bi atoms is clearly depicted ().

For a pure Bi–C–Bi 2-electron-3-center bond, the *ipso*-C atom should ideally be sp^2^-hybridized and consequently the π-conjugation of the central phenyl-ring should remain intact. In an effort to quantify this, we inspected the IRI including solely the π-electrons (IRI-π).^35b^ From the IRI-π it becomes visible that the π-conjugation is interrupted at the *ipso*-C atom (). In addition, the *iso*-surface of the localized orbital locator^36^ of the π-orbitals (LOL-π) show the same trend () indicating deviations from the ideal sp^2^ hybridization and a bonding situation that might be better described as two 2-electron-2-center bonds.

As a side note, from the IRI-π can be seen, that *iso*-surface of the Bi_3_-core is significantly wider with lower electron density values in comparison to the more compact (and higher electron density values) of the π-delocalization of the phenyl rings. Comparing the Wiberg bond orders, NLMO/NPA bond orders as well as the delocalization indices of the Bi–C bonds within **7** and **IV**, show smaller values for **7** (), again pointing rather to a 2-electron-3-center scenario within **7**. Finally, we computed the force constants and compliance coupling constants in the scheme of the generalized compliance constants method, which allows o studying weak covalent and noncovalent interactions.^37^ The Bi–Bi bond give rise to force constants of 0.969 Å mdyn^–1^ and 0.706 Å mdyn^–1^ (smaller values indicate stronger bonds), whereas the Bi–C lead to force constants of 1.156 Å mdyn^–1^ and 1.535 Å mdyn^–1^. Notably, the compliance coupling constants *C* ranging from +0.203 Å mdyn^–1^ to – 0.331 Å mdyn^–1^ reveal substantial coupling between the individual bonds of the CBi_3_ core ().

## Conclusion

In conclusion, the chloropnictogenium cations [M^s^FluindECl]^+^ (**2As**, **2Sb**, and **2Bi**) are the missing link between the two classes of carbene analogues of group 15, namely, the diarylpnictogenium cations [R_2_E]^+^ (**IE**) and neutral arylpnictinidenes [RE] (**IIE**). They are substantially more Lewis acidic than the corresponding [M^S^FluindEMes]^+^ (**IE**, E = P, As,^2^ Sb, Bi^3^), presumably due to the larger charge separation. They readily react with Et_3_SiH to give the neutral arylarsenic­(III) dihydride M^S^FluindAsH_2_ (**3**), the cationic arylhydridostibenium­(III) [M^S^FluindSbH]^+^ (**4**) and the aryltribismuth­(I) dication [M^S^FluindBi_3_]^2+^ (**7**). We speculate that in all three cases the respective arylhydridopnictogenium ions are formed, being too reactive to be isolated in the case of arsenic. For bismuth, both the Bi–H and Bi–C bonds are weaker compared to the lighter homologues, which leads to the formation of **7** via a complex reaction. The cation **4** reacts as Brønsted acid toward Me_3_E (E = Ga, In) and the diphenyldichalcogenides (PhCh)_2_ (Ch = S, Se, Te) and therefore can also be regarded as the first protonated stibinidene­(I). It provides access to the cation **5In**, which can be viewed as a donor–acceptor complex between a arylstibenidene and the dimethylindenium cation as well as the doubled bonded SbCh species [M^S^FluindSbChPh]^+^ (**6Ch**, Ch = S, Se, Te). We are currently exploring the reactivity of **4** toward purely organic substrates to further explore the synthetic potential of this carbene analogue. The dication **7** extends the small but rapidly growing number of molecular bismuth­(I) species and related species containing redox noninnocent ligands,^4,5,38^ and it shows unprecedented 2-electron-3-center bonding, typically observed for elements of smaller group numbers, such as lithium, beryllium, boron, copper, and their heavier congeners.

## Supplementary Material





## Notes

Coordinates for all computed structures (XYZ)
Detailed experimental procedures along with copies of NMR-spectra of all newly synthesized compounds as well as full crystallographic and computational details (PDF)

## Notes

This paper is dedicated to the memory of Professor Herbert W. Roesky.
