# Supplementary material for: Carbene Analogues of Group 15: Reduction of s‑Hydrindacene-Based Chloropnictogenium Ions To Access an Antimony Hydride Monocation and a Trinuclear Bismuth Dication
Source: J Am Chem Soc. 2026 Jul 3;148(27):29177–85. doi: 10.1021/jacs.6c07386 (PMC13383741; doi:10.1021/jacs.6c07386)
Supplement: Supplementary file 2 [file ja6c07386_si_002.pdf]

Supporting Information for

Carbene Analogues of Group 15: Reduction of s-Hydrindacene-Based Chloropnictogenium Ions to Access an Antimony Hydride Monocation and a Trinuclear Bismuth Dication

Marvin Janssen,[a] Marian Olaru,[a] Enno Lork,[a] Jens Beckmann,[a]* Emanuel Hupf[a]*

[a] Institute for Inorganic Chemistry and Crystallography, University of Bremen, D-28359 Bremen, Germany.

Contents

Experimental procedures 2

Synthesis and characterization of $M^SFluindSbCl_2$ (1Sb) 3

Synthesis and characterization of $M^SFluindBiCl_2$ (1Bi) 8

Synthesis and characterization of $[M^SFluindAsCl][B(C_6F_5)_4]$ (2As) 13

Synthesis and characterization of $[M^SFluindSbCl][B(C_6F_5)_4]$ (2Sb) 20

Synthesis and characterization of $[M^SFluindBiCl][B(C_6F_5)_4]$ (2Bi) 27

Synthesis and characterization of $M^SFluindAsH_2$ (3) 35

Synthesis and characterization of $[M^SFluindSbH][B(C_6F_5)_4]$ (4) 40

Synthesis and characterization of $[M^SFluindSbInMe_2][B(C_6F_5)_4]$ (5In) 47

Reactivity of $[M^SFluindSbH][B(C_6F_5)_4]$ (4) towards Me_3Ga 54

Synthesis and characterization of $[M^SFluindSbSPh][B(C_6F_5)_4]$ (6S) 61

Synthesis and characterization of $[M^SFluindSbSePh][B(C_6F_5)_4]$ (6Se) 68

Synthesis and characterization of $[M^SFluindSbTePh][B(C_6F_5)_4]$ (6Te) 76

Synthesis and characterization of $[M^SFluindBi_3][B(C_6F_5)_4]_2$ (7) 84

Determination of Gutmann-Beckett acceptor numbers 95

UV-vis absorption spectra 97

X-ray crystallography 100

Computational Methods 106

Additional References 130

Experimental procedures

General Information

Unless otherwise stated, all reactions, manipulations, work-up and purifications were performed under inert argon atmosphere using anhydrous solvents. Reagents used in this work including $AsCl_3$, $SbCl_3$, $BiCl_3$, triethylsilane, lithiumaluminiumhydride, Ph_2E_2 (E = S, Se, Te), Me_3E (E = Ga, In) and dimethyl-5-bromoisophthalate were obtained commercially and were used as received. M^SFluindBr, M^SFluind = dispiro[fluorene-9,3'-(1',1',7',7'-tetramethyl-s-hydrindacen-4'-yl)-5',9''-fluorene][S1], M^SFluindLi(THF)$_2$,[12] M^SFluindAsCl$_2$[12] and $K[B(C_6F_5)_4]$[S2] were prepared following the published procedures. Anhydrous dichloromethane, hexane and toluene were collected from an SPS800 mBraun solvent purification system and stored over 3 Å molecular sieves. Other solvents, such as 1,2-$Cl_2C_6H_4$, were dried directly over 3 Å molecular sieves. Deuterated solvents were degassed and dried over 3 Å molecular sieves under argon.

Unless otherwise noted, NMR spectra were recorded at room temperature on Bruker Avance Neo 600 MHz spectrometers. ^{1}H, ^{13}C{^{1}H}, ^{11}B, ^{19}F, ^{77}Se and ^{125}Te NMR spectra are reported on the δ scale (ppm) and are referenced against $SiMe_4$, $BF_3{\cdot}Et_2O$ (15% in $CDCl_3$), $CFCl_3$, Me_2Se and Me_2Te. ^{1}H and ^{13}C{^{1}H} chemical shifts are reported relative to the residual peak of the solvent ($CDHCl_2$: 5.32 ppm, for CD_2Cl_2; C_6HD_5: 7.16 ppm for C_6D_6) in the ^{1}H NMR spectra, and to the peak of the deuterated solvent (CD_2Cl_2: 53.84 ppm; C_6D_6: 128.06 ppm) in the ^{13}C{^{1}H} NMR spectra.[S3] The assignment of the ^{1}H and ^{13}C{^{1}H} resonance signals was made in accordance with the COSY, HSQC and HMBC spectra.

The ESI HRMS spectra were measured on a Bruker Impact II spectrometer. Acetonitrile or dichloromethane/acetonitrile solutions ($c = 1{\cdot}10^{-5}$ mol·L^{-1}) were injected directly into the spectrometer at a flow rate of 3 μL·min^{-1}. Nitrogen was used both as a drying gas and for nebulization with flow rates of approximately 5 L·min^{-1} and a pressure of 5 psi. Pressure in the mass analyzer region was usually about $1{\cdot}10^{-5}$ mbar. Spectra were collected for 1 min and averaged. The nozzle-skimmer voltage was adjusted individually for each measurement.

UV-vis absorption spectra were recorded on a VWR UV-1600PC spectrophotometer.

Synthesis and characterization of M^{S}FluindSbCl$_2$ (1Sb)

The synthesis of **1Sb** followed a modified procedure according to Cornella *et al*. [7]

To a solid mixture of M^{S}FluindLi(THF)$_2$ (1.00 g, 1.51 mmol) and $SbCl_3$ (344 mg, 1.51 mmol) was added toluene (15 mL) and the resulting suspension was stirred at room temperature for 2 hours. The solvent was removed under vacuum and to the remaining solid was added CH_2Cl_2 (45 mL). The turbid solution was filtered under argon to remove LiCl. After evaporation of the solvent the solid product was recrystallized from boiling toluene to obtain M^{S}FluindSbCl$_2$ (**1Sb**) as a colourless crystalline solid (651 mg, 0.921 mmol, 61%).

^{1}H NMR (600 MHz, CD_2Cl_2): δ = 7.63 (dt, $^3J(^1H-^1H)$ = 8 Hz, $^4J(^1H-^1H)$ = 1 Hz, 4H, H14, H21), 7.48 (s, 1H, H4), 7.30 (ddd, $^3J(^1H-^1H)$ = 8 Hz, $^3J(^1H-^1H)$ = 7 Hz $^4J(^1H-^1H)$ = 2 Hz, 4H, H13, H20), 7.22 (m, 8H, H11, H12, H18 and H19), 2.38 (s, 4H, H6a, H6b), 1.56 (s, 12H, H8, H9). **$^{13}C\{^1H\}$ NMR (151 MHz, CD_2Cl_2):** δ = 158.2 (s, C3), 155.9 (s, C10 and C17), 150.9 (s, C2), 149.7 (s, C1), 140.5 (s, C15 and C16), 128.5 (s, C12 and C19) , 128.2 (s, C13 and C20), 125.8 (s, C11 and C18), 121.9 (s, C4), 121.1 (s, C14 and C21), 65.8 (s, C5), 59.8 (s, C6), 43.9 (s, C7), 32.8 (s, C8 and C9).

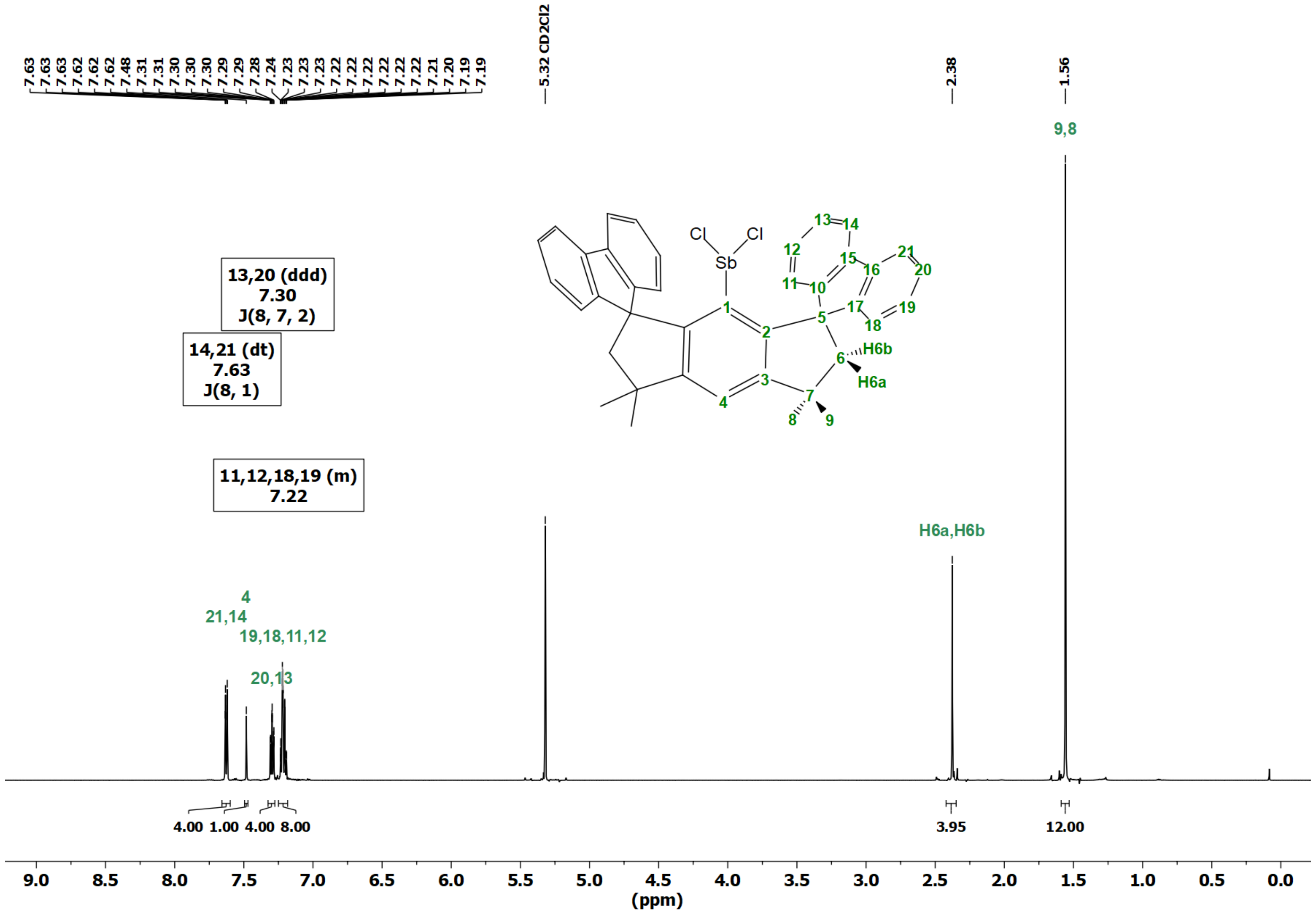

Figure S1. ^{1}H NMR (CD_2Cl_2, 600 MHz) spectrum of **1Sb**.

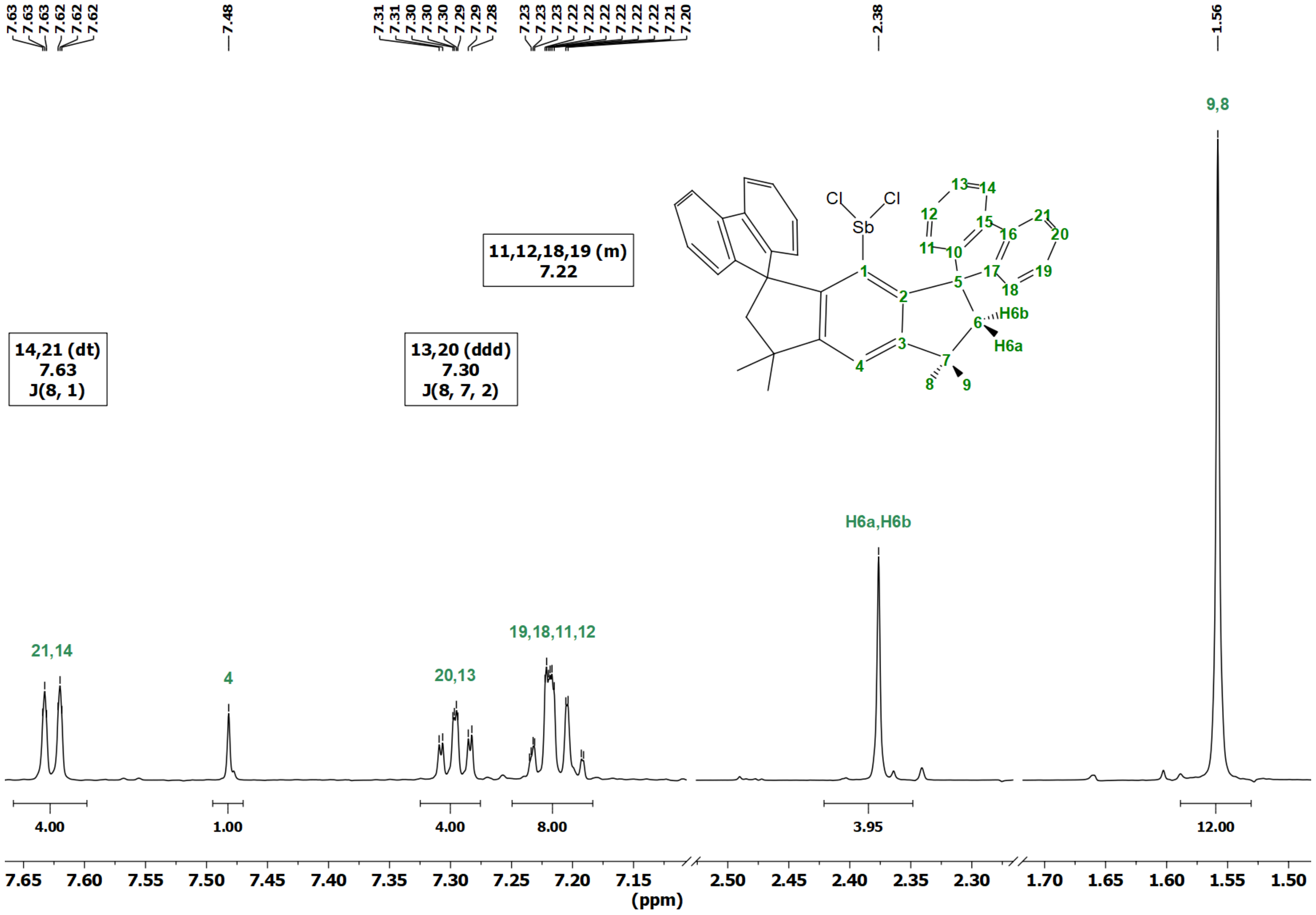

Figure S2. Detailed ^{1}H NMR (CD_2Cl_2, 600 MHz) spectrum of **1Sb**.

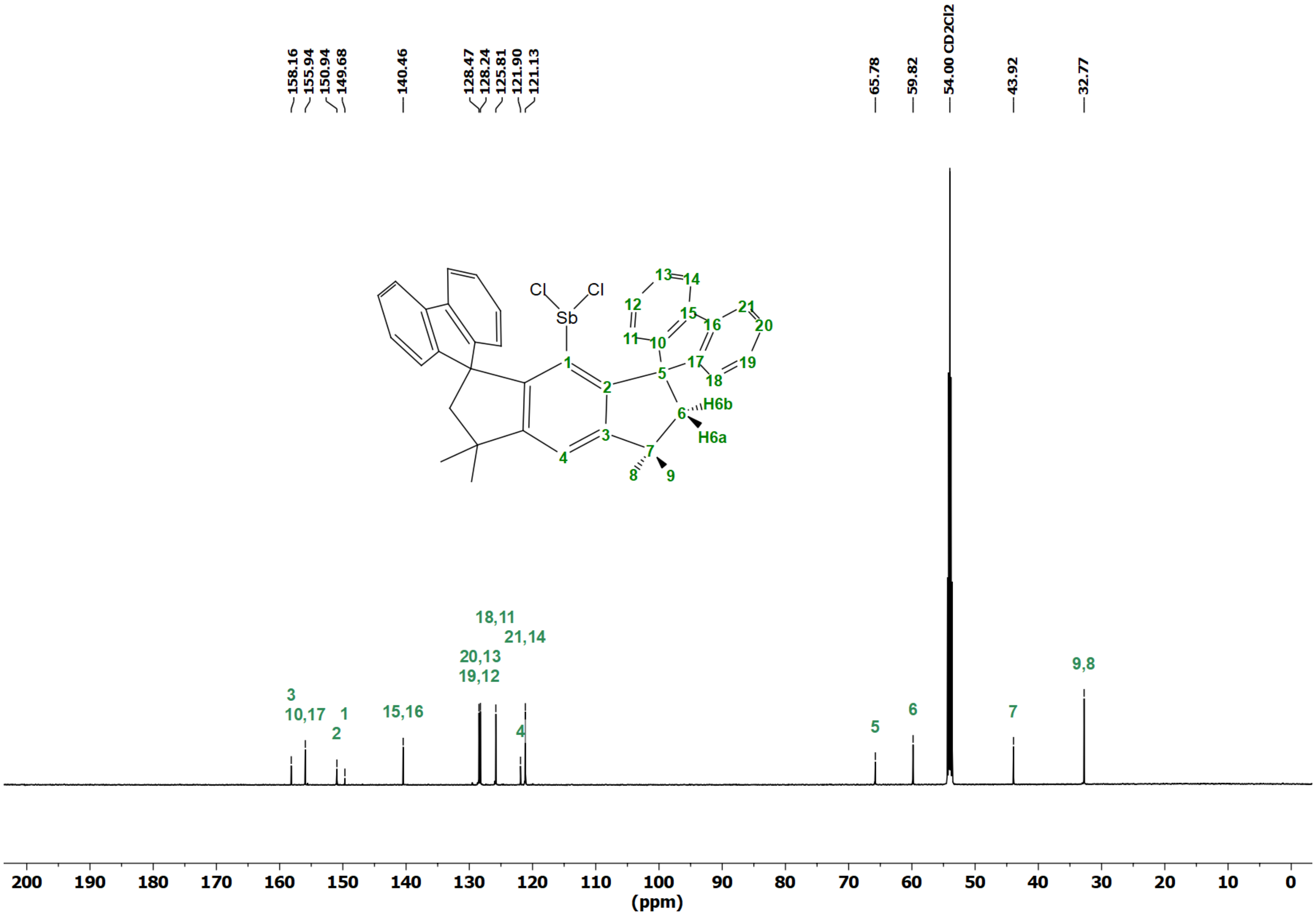

Figure S3. $^{13}C\{^{1}H\}$ NMR (CD_2Cl_2, 151 MHz) spectrum of **1Sb**.

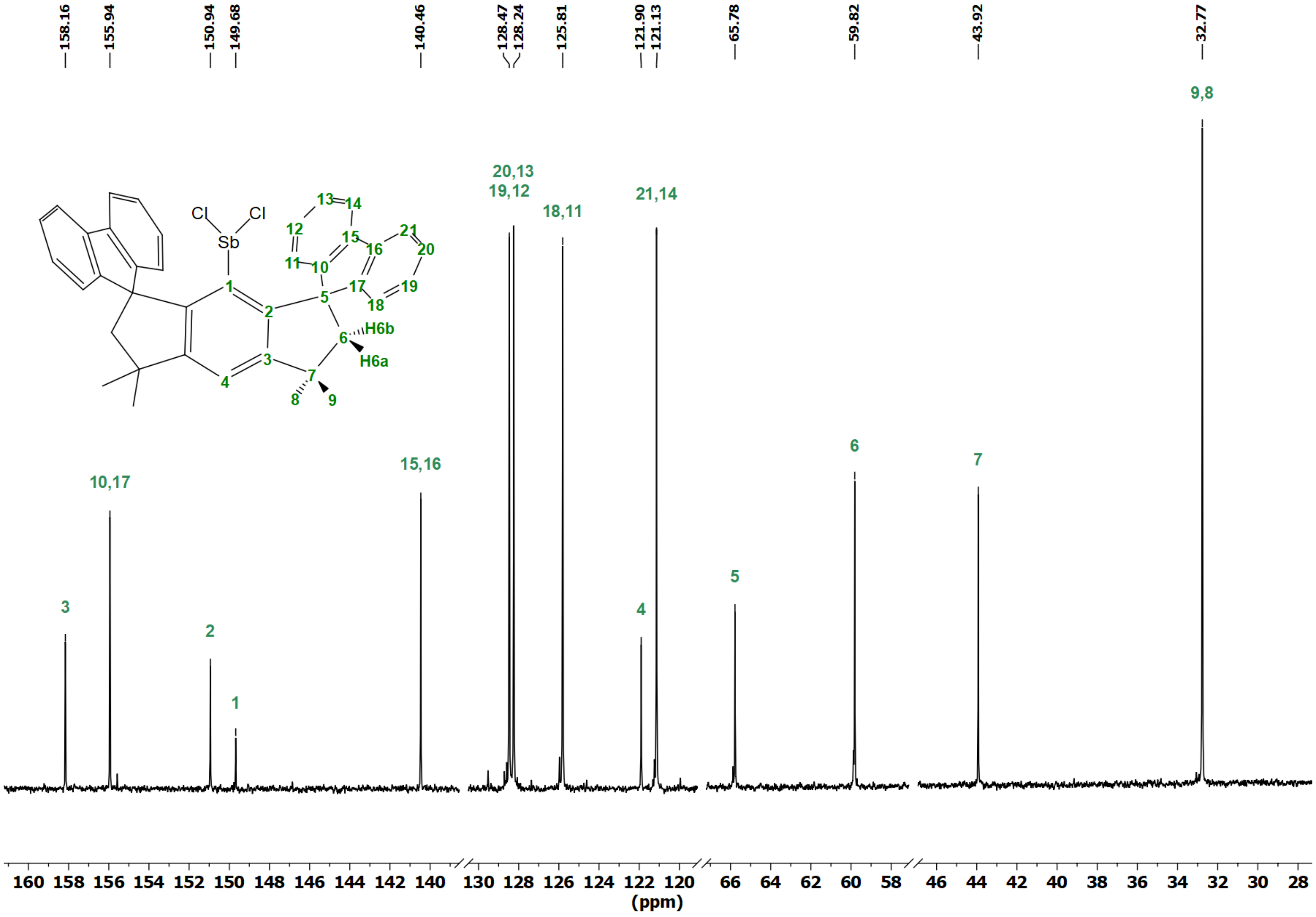

Figure S4. Detailed $^{13}C\{^1H\}$ NMR (CD_2Cl_2, 151 MHz) spectrum of **1Sb**.

Synthesis and characterization of M^SFluindBiCl$_2$ (1Bi)

The synthesis of **1Bi** followed a modified procedure according to Cornella *et al*.[4a]

To a solid mixture of M^SFluindLi(THF)$_2$ (1.00 g, 1.51 mmol) and $BiCl_3$ (476 mg, 1.51 mmol) was added toluene (15 mL) and the resulting suspension was stirred at room temperature for 2 hours. The solvent was removed under vacuum. To the remaining solid was added CH_2Cl_2 (60 mL) and the turbid solution was filtered under argon to remove LiCl. After evaporation of the solvent the solid product was recrystallized from boiling toluene to obtain M^SFluindBiCl$_2$ (**1Bi**) as a pale yellow solid (786 mg, 0.990 mmol, 66%).

^{1}H NMR (600 MHz, CD_2Cl_2): δ = 7.63 (dt, $^3J(^1H\text{-}^1H)$ = 8 Hz, $^4J(^1H\text{-}^1H)$ = 1 Hz, 4H, H14, H21), 7.59 (s, 1H, H4), 7.31 (ddd, $^3J(^1H\text{-}^1H)$ = 8 Hz, $^3J(^1H\text{-}^1H)$ = 7 Hz $^4J(^1H\text{-}^1H)$ = 2 Hz, 4H, H13, H20), 7.25 (m, 8H, H11, H12, H18 and H19), 2.37 (s, 4H, H6a, H6b), 1.59 (s, 12H, H8, H9). **$^{13}C\{^1H\}$ NMR (151 MHz, CD_2Cl_2):** δ = 220.8 (s (br), C1), 161.9 (s, C3), 155.4 (s, C10 and C17), 155.1 (s, C2), 139.9 (s, C15 and C16), 128.9 (s, C12 and C19), 128.7 (s, 13 and C20), 126.1 (s, C11 and C18), 121.5 (s, C14 and C21), 120.1 (s, C4), 65.9 (s, C5), 59.3 (s, C6), 45.1 (s, C7), 32.9 (s, C8 and C9).

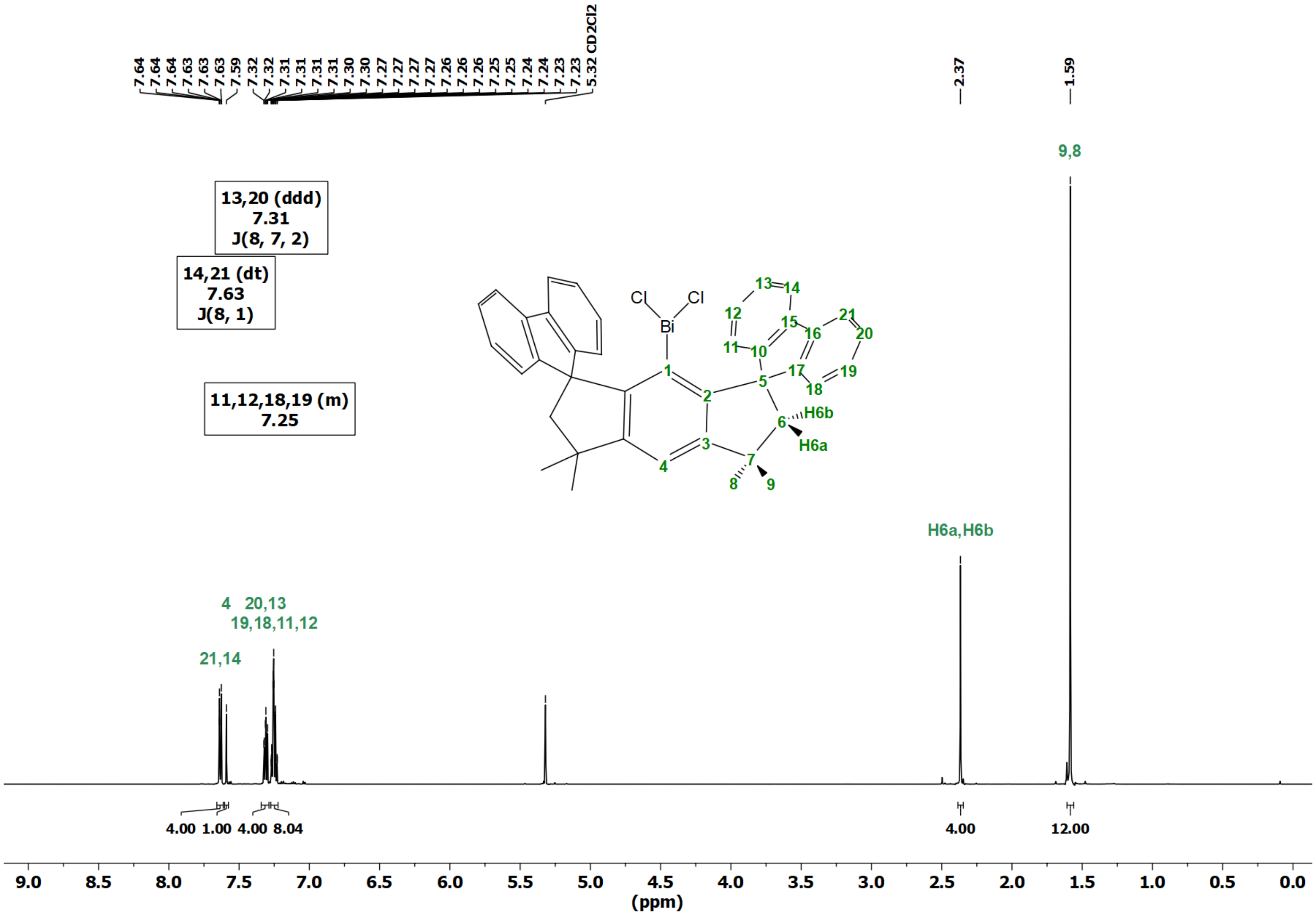

Figure S5. ^{1}H NMR (CD_2Cl_2, 600 MHz) spectrum of **1Bi**.

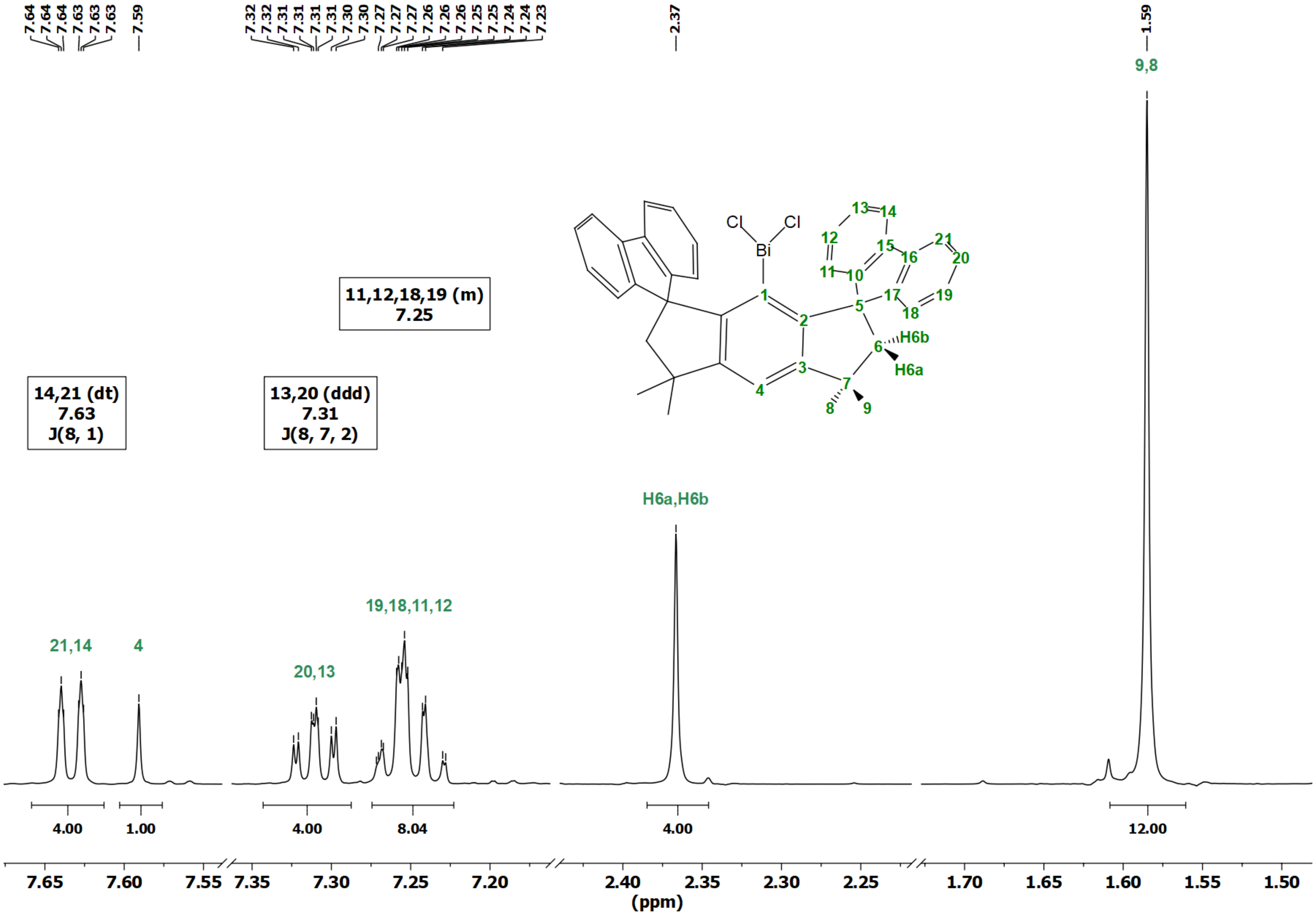

Figure S6. Detailed ^{1}H NMR (CD_2Cl_2, 600 MHz) spectrum of **1Bi**.

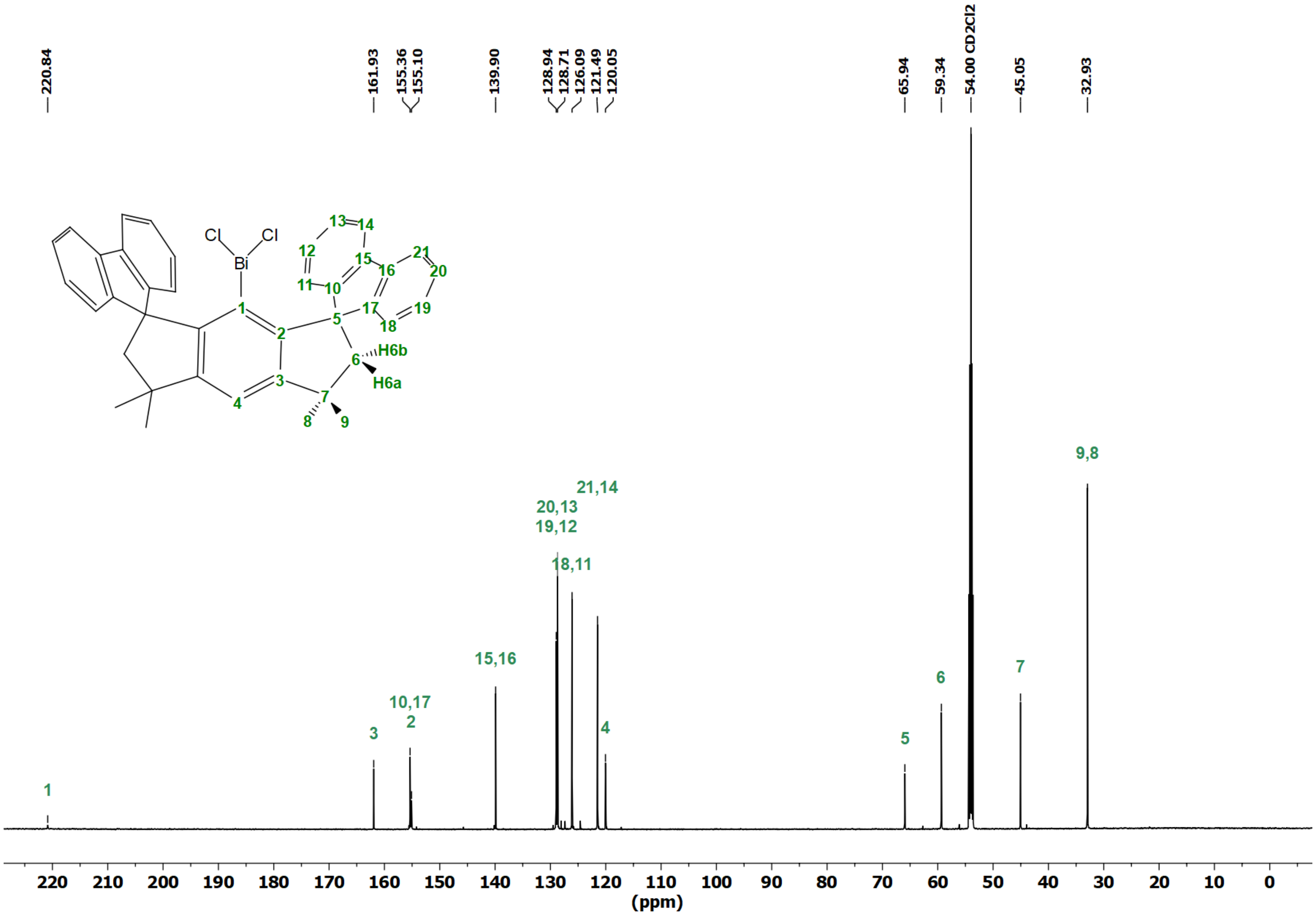

Figure S7. $^{13}C\{^{1}H\}$ NMR (CD_2Cl_2, 151 MHz) spectrum of **1Bi**.

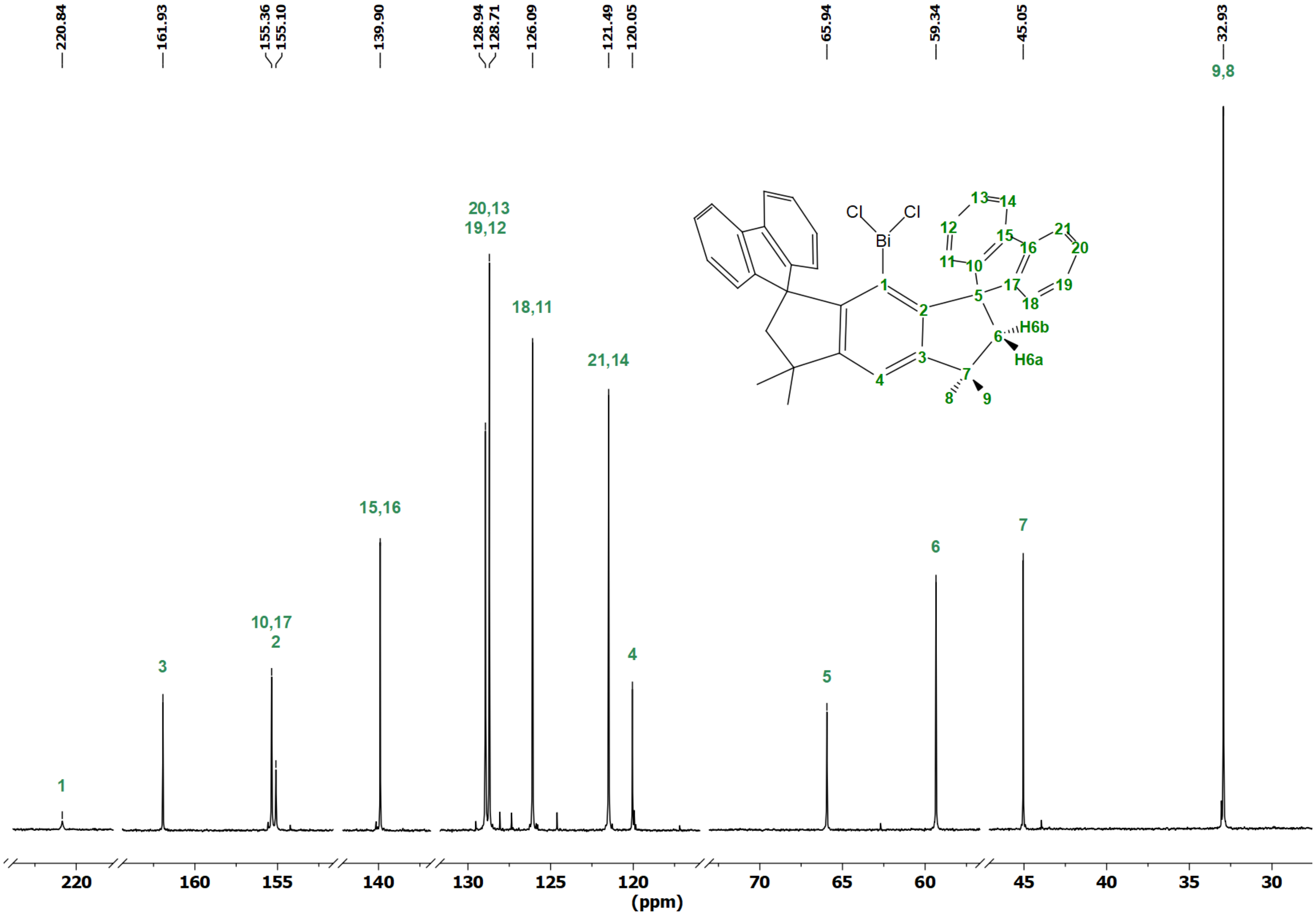

Figure S8. Detailed $^{13}C\{^{1}H\}$ NMR (CD_2Cl_2, 151 MHz) spectrum of **1Bi**.

Synthesis and characterization of [M^{S}FluindAsCl][B(C_6F_5)$_4$] (2As)

M^{S}FluindAsCl$_2$ (**1As**, 400 mg, 0.607 mmol) and K[B(C_6F_5)$_4$] (436 mg, 0.607 mmol) were suspended in CH_2Cl_2 (10 mL) and stirred for 3 hours at room temperature. The turbid reaction mixture was filtered under argon to remove KCl. Hexane (20 mL) was layered over the CH_2Cl_2 filtrate. The crystalline solid was decanted and dried at reduced pressure to obtain [M^{S}FluindAsCl][B(C_6F_5)$_4$] (**2As**) as a dark red crystalline solid (507 mg, 0.389 mmol, 64%).

^{1}H NMR (601 MHz, CD_2Cl_2): δ = 7.75 (dt, ^3J = 8 Hz, ^4J = 1 Hz, 2H, H21), 7.66 – 7.62 (m, 2H, H14), 7.59 (td, ^3J = 8 Hz, ^4J = 1 Hz, 2H, H20), 7.55 (td, ^3J = 7 Hz, ^4J = 1 Hz, 2H, H19), 7.46 – 7.41 (m, 3H, H4 and H11), 7.40 – 7.34 (m, 6H, H12, H13 and H18), 2.71 (d, ^3J = 15 Hz, 2H, H6a), 2.65 (d, ^3J = 15 Hz, 2H, H6b), 1.77 (s, 6H, H8), 1.63 (s, 6H, H9). **^{13}C{^{1}H} NMR (151 MHz, CD_2Cl_2):** δ = 156.5 (s, C2), 155.5 (s, C3), 153.1 (s, C10), 151.7 (s, C17), 148.7 (d, br, $^1J(^{13}C–^{19}F)$ = 239 Hz, *C*$_6$F$_5$), 147.6 (s, C4), 145.2 (s, C16), 138.9 (d, br, $^1J(^{13}C–^{19}F)$ = 246 Hz, *C*$_6$F$_5$), 137.0 (d, br, $^1J(^{13}C–^{19}F)$ = 248 Hz, *C*$_6$F$_5$), 135.8 (s, C15), 135.6 (s, C20), 133.8 (s, C19), 132.2 (s, C12), 129.7 (s, C18), 129.4 (s, C13), 125.9 (s, C21), 125.8 (s, C11), 123.9 (s, C14), 123.3 (s, C4), 63.0 (s, C5), 52.4 (s, C6), 47.7 (s, C7), 32.8 (s, C9), 32.3 (s, C8). **^{11}B NMR (193 MHz, CD_2Cl_2):** $\delta = -16.7$ (s). **^{19}F NMR (565 MHz, CD_2Cl_2):** $\delta = -133.9$ (br, 8F, *o*-C_6F_5), −162.9 (t, $^3J(^{19}F\text{-}^{19}F)$ = 20 Hz, 4F, *p*-C_6F_5), −166.9 (t, br, $^3J(^{19}F\text{-}^{19}F)$ = 19 Hz, 8F, *m*-C_6F_5). **HRMS ESI (m/z):** [M]$^+$ calculated for $C_{40}H_{33}AsCl$, 623.14813; found 623.14772. **Mp.** 238-240 °C.

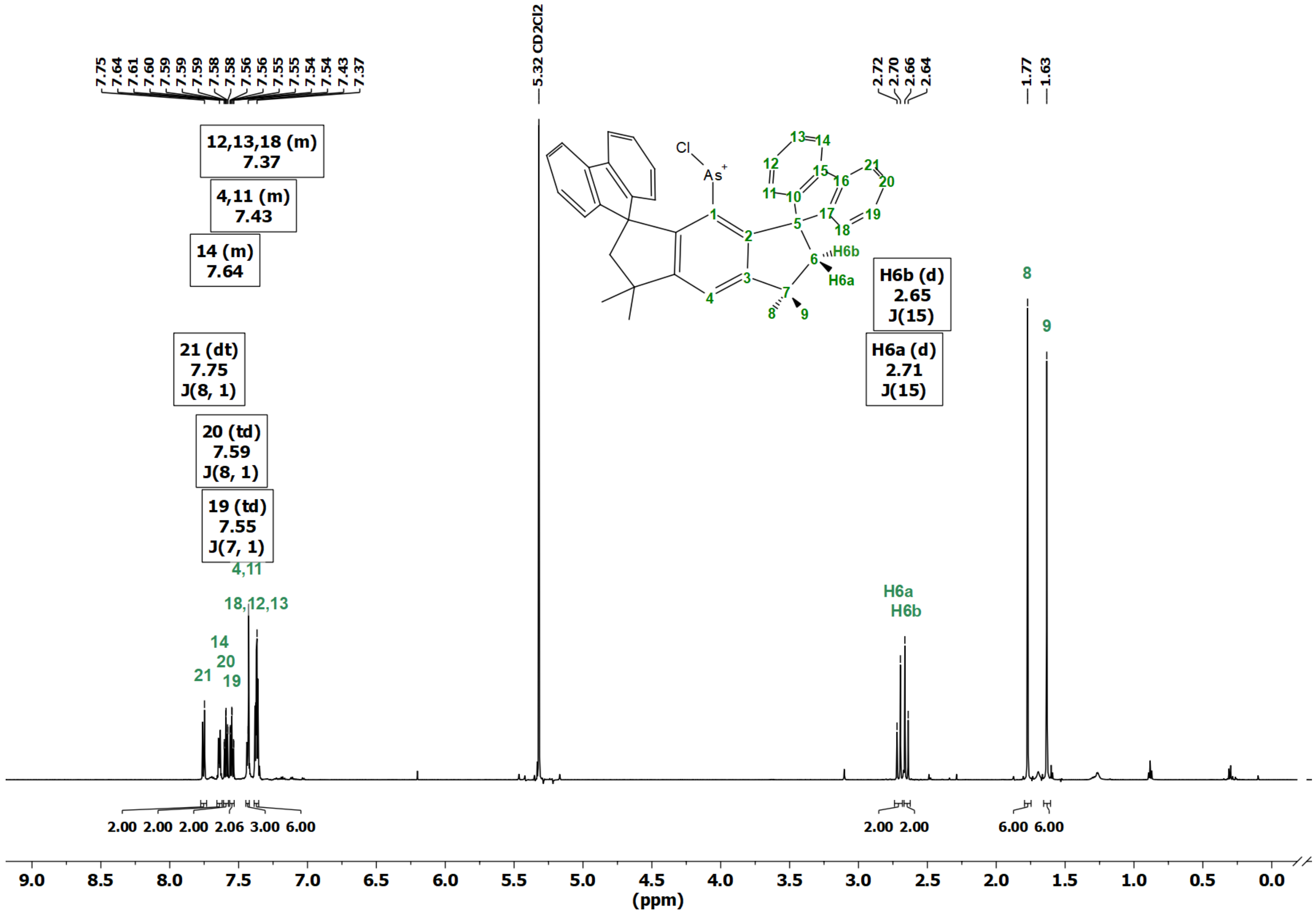

Figure S9. ^{1}H NMR (CD_2Cl_2, 600 MHz) spectrum of **2As**.

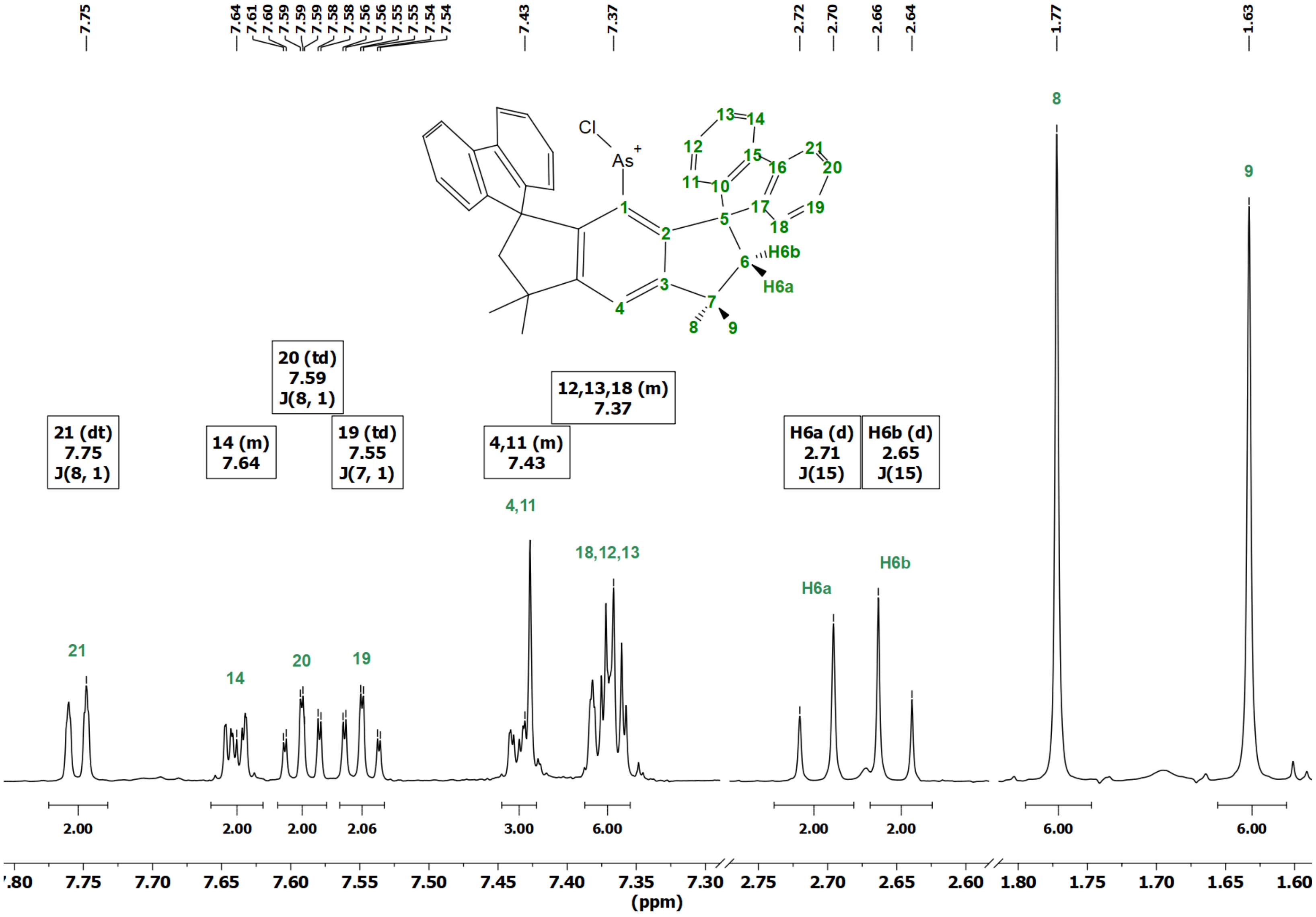

Figure S10. Detailed ^{1}H NMR (CD_2Cl2, 600 MHz) spectrum of **2As**.

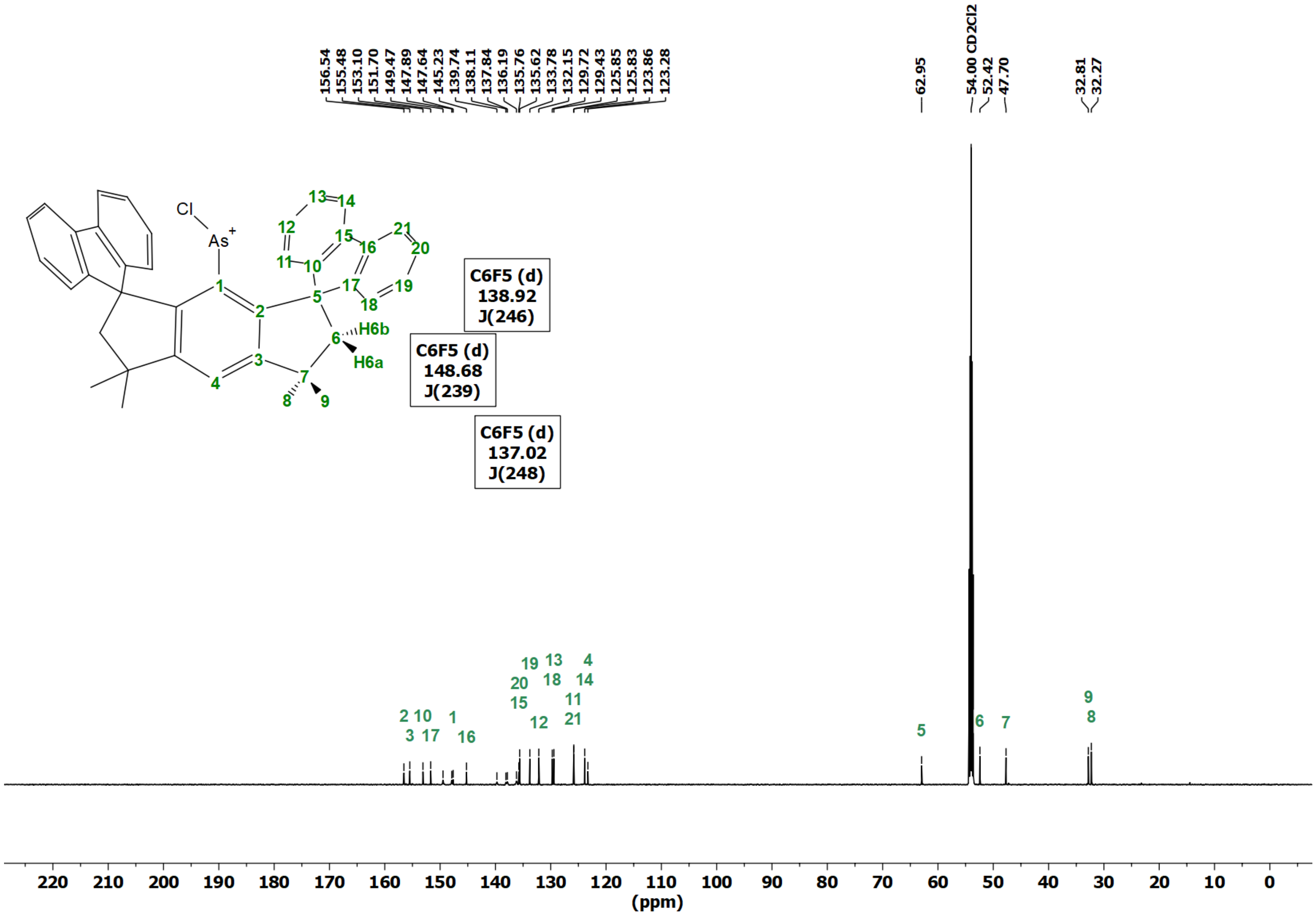

Figure S11. $^{13}C\{^{1}H\}$ NMR (CD_2Cl_2, 151 MHz) spectrum of **2As**.

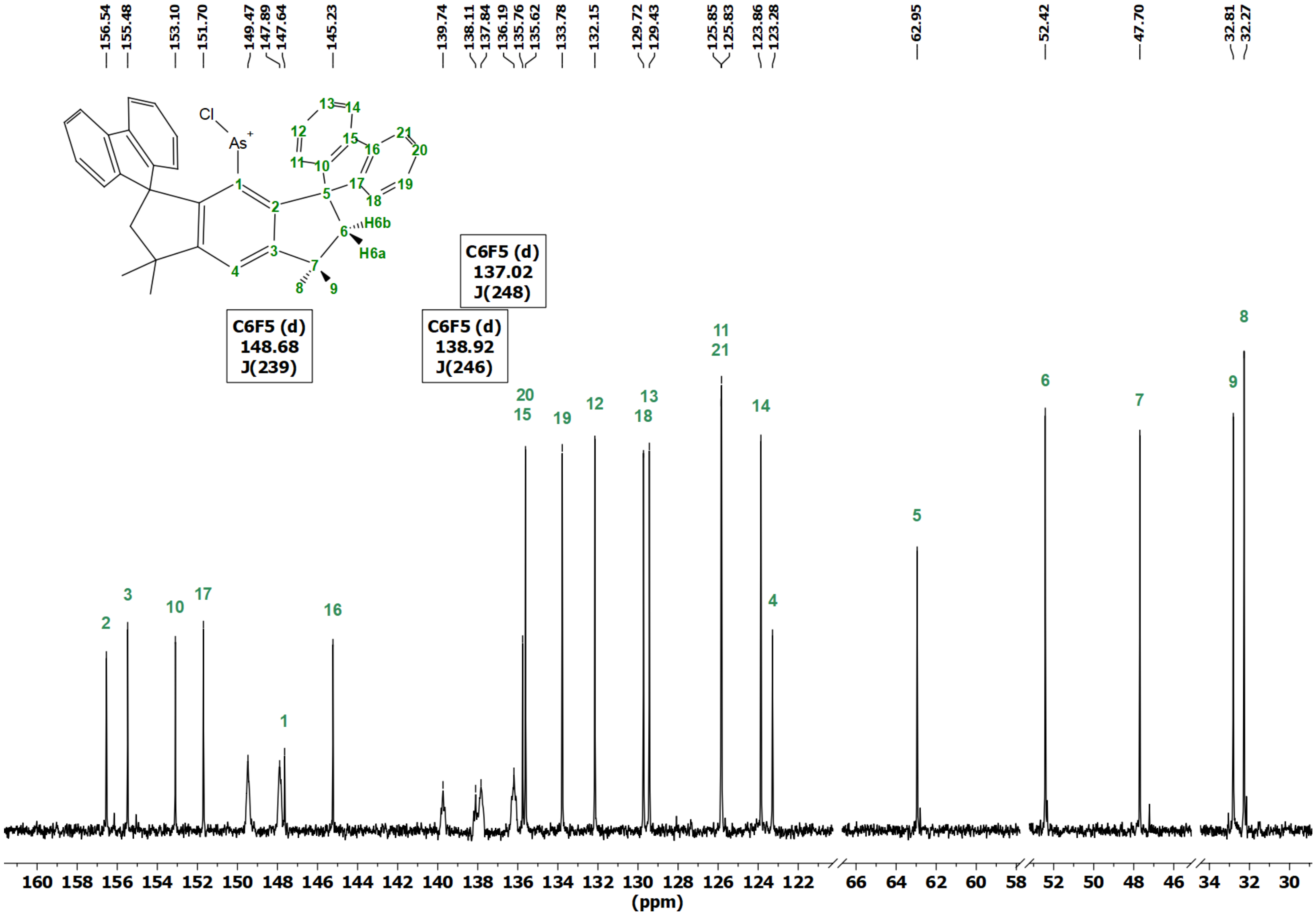

Figure S12. Detailed $^{13}C\{^{1}H\}$ NMR (CD_2Cl_2, 151 MHz) spectrum of **2As**.

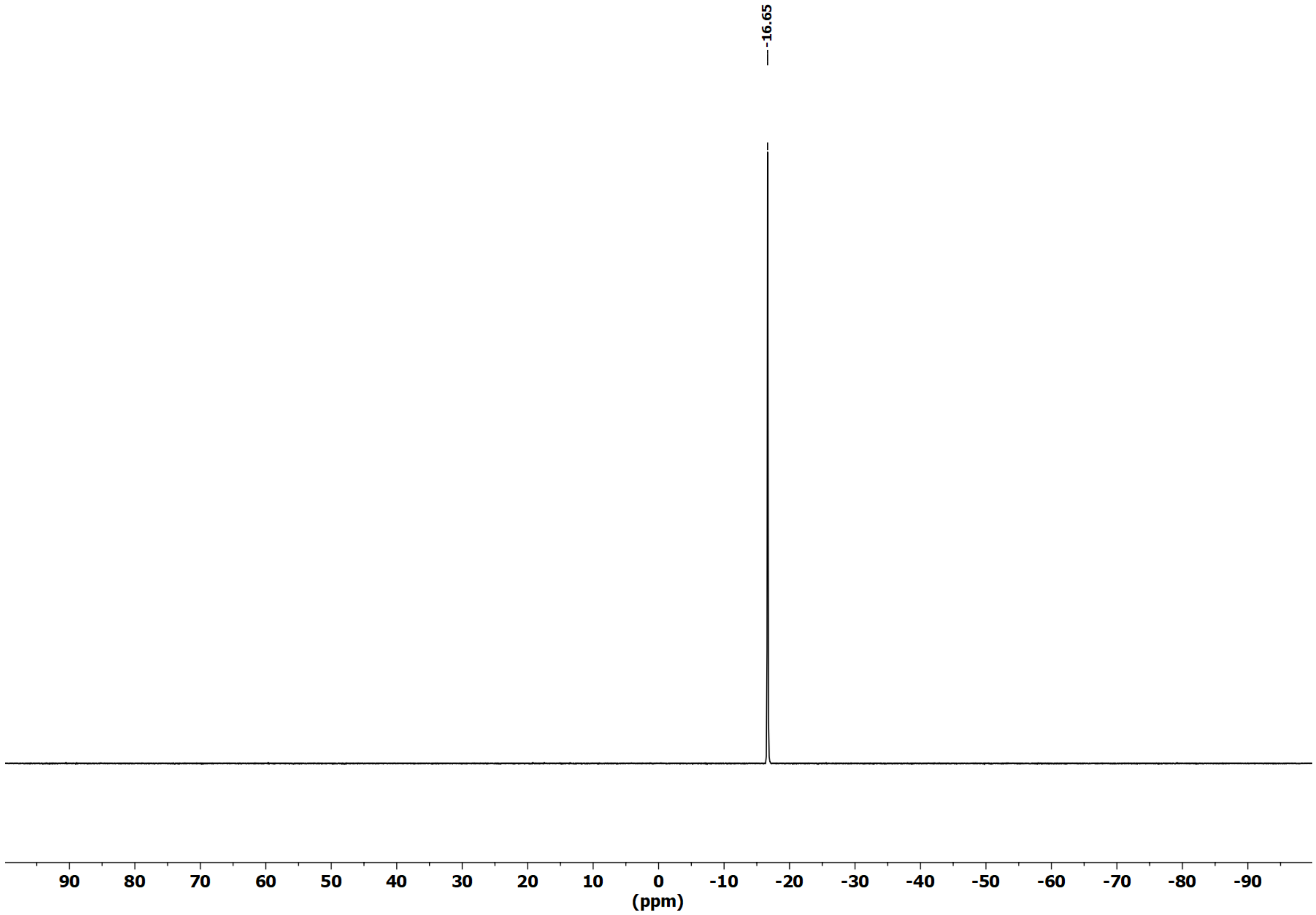

Figure S13. ^{11}B NMR (CD_2Cl_2, 193 MHz) spectrum of **2As**.

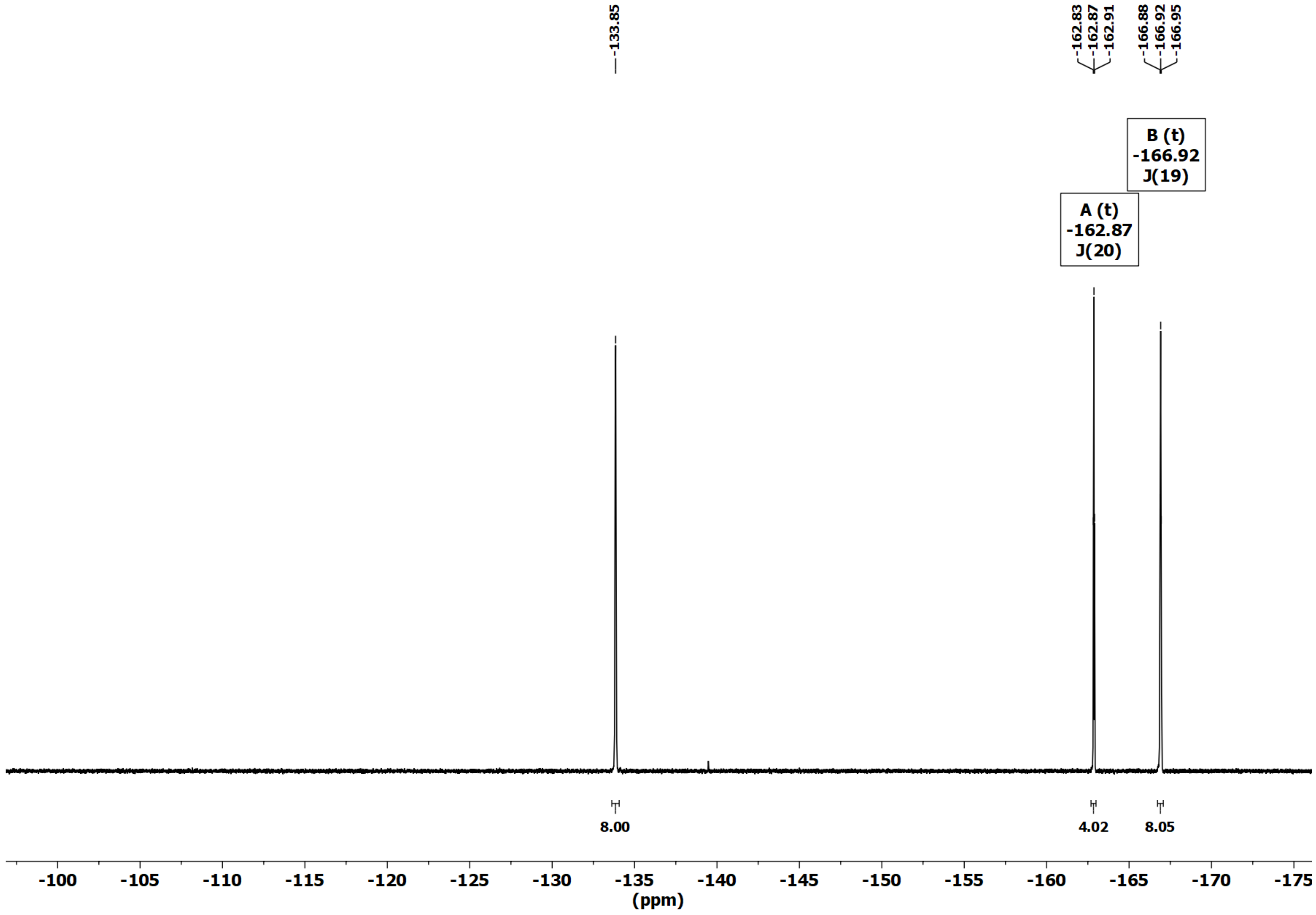

Figure S14. ^{19}F NMR (CD_2Cl_2, 565 MHz) spectrum of **2As**.

Synthesis and characterization of [M^{S}FluindSbCl][B(C_6F_5)$_4$] (2Sb)

To a solid mixture of M^{S}FluindSbCl_2 (**1Sb**) (400 mg, 0.566 mmol) and K[B(C_6F_5)$_4$] (407 mg, 0.566 mmol) was added CH_2Cl_2 (10 mL) and the reaction mixture was stirred for 1 hour at room temperature. After filtration under argon to remove KCl the filtrate was layered with hexane (20 mL). The crystalline product was decanted and dried at reduced pressure to obtain **2Sb** as a dark orange crystalline solid (563 mg, 0.417 mmol, 74%).

^{1}H NMR (600 MHz, CD_2Cl_2): δ = 7.93 (dt, $^3J(^1H-^1H)$ = 8 Hz, $^4J(^1H-^1H)$ = 1 Hz, 2H, H21), 7.61 (m, 4H, H12 and H19), 7.56 (td, $^3J(^1H-^1H)$ = 8 Hz, $^4J(^1H-^1H)$ = 1 Hz, 2H, H20), 7.52 (s, 1H, H4), 7.45 (dt, $^3J(^1H-^1H)$ = 8 Hz, $^4J(^1H-^1H)$ = 1 Hz, 2H, H18), 7.36 (m, 6H, H11, H13 and H14), 2.73 (d, $^3J(^1H-^1H)$ = 15 Hz, 2H, H6a), 2.67 (d, $^3J(^1H-^1H)$ = 15 Hz, 2H, H6b), 1.77 (s, 6H, H8), 1.64 (s, 6H, H9). **^{13}C{^{1}H} NMR (151 MHz, CD_2Cl_2):** δ = 162.4 (s, C1), 157.8 (s, C2), 156.1 (s, C3), 153.5 (s, C17), 152.3 (s, C10), 148.7 (d), 142.4 (s, C16), 138.8 (d), 136.9 (d), 134.8 (s, C20), 134.8 (s, C15), 134.0 (s, C19), 132.1 (s, C13), 129.2 (s, C11), 129.2 (s, C18), 126.1 (s, C21), 125.6 (s, C14), 123.6 (s, C12), 122.5 (s, C4), 63.6 (s, C5), 52.2 (s, C6), 47.1 (s, C7), 33.2 (s, C9), 32.4 (s, C8). **^{11}B NMR (193 MHz, CD_2Cl_2):** δ = −16.6 (s). **^{19}F NMR (565 MHz, CD_2Cl_2):** δ = −133.1 (br, 8F, *o*-C_6F_5), −163.7 (t, $^3J(^{19}F-^{19}F)$ = 20 Hz, 4F, *p*-C_6F_5), −167.6 (t, br, $^3J(^{19}F-^{19}F)$ = 19 Hz, 8F, *m*-C_6F_5). **HRMS ESI (m/z):** $[M]^+$ calculated for $C_{40}H_{33}ClSb$, 669.13035; found 669.12903. **Mp.** 252 °C (decomposition)

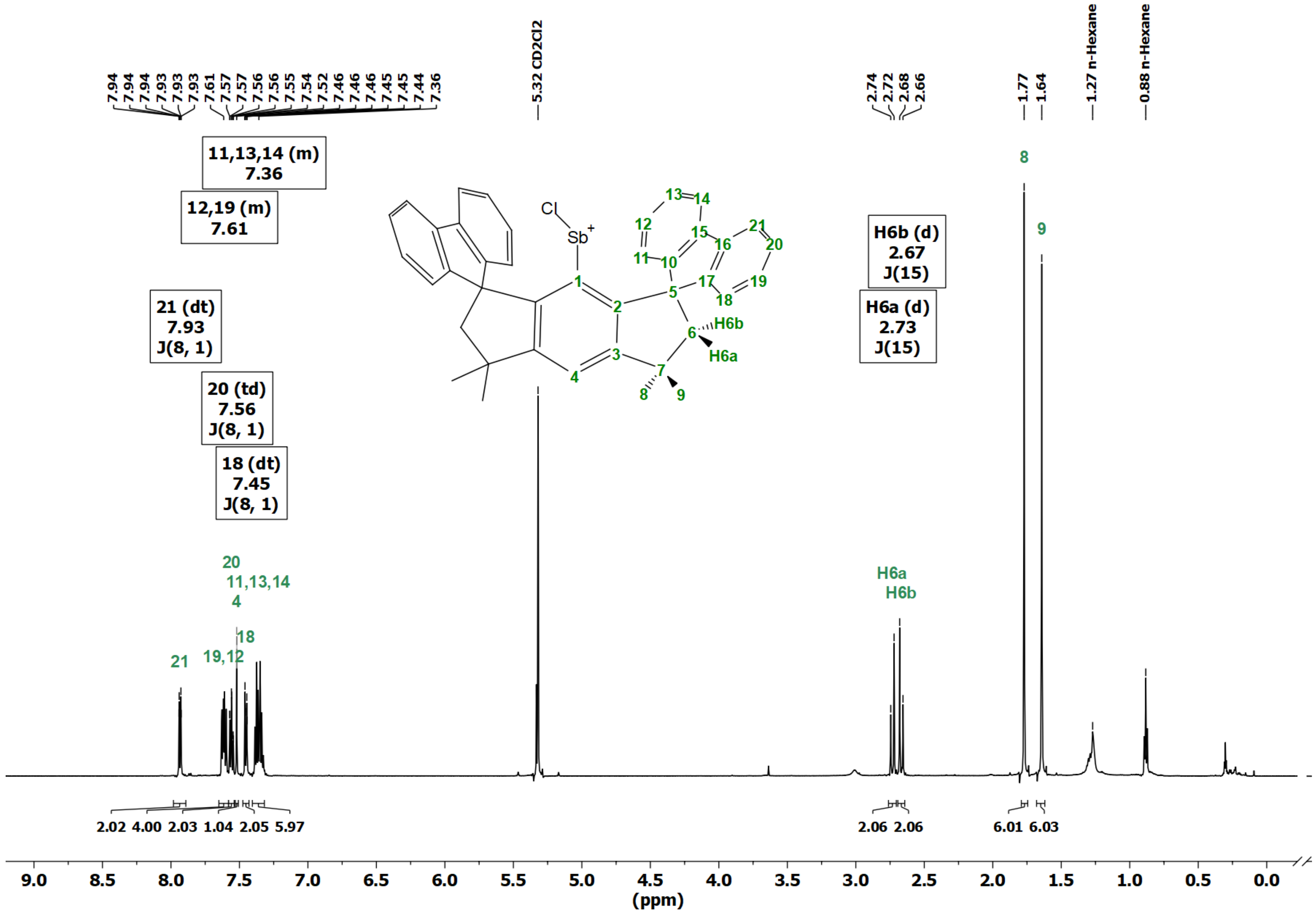

Figure S15. ^{1}H NMR (CD_2Cl_2, 600 MHz) spectrum of **2Sb**.

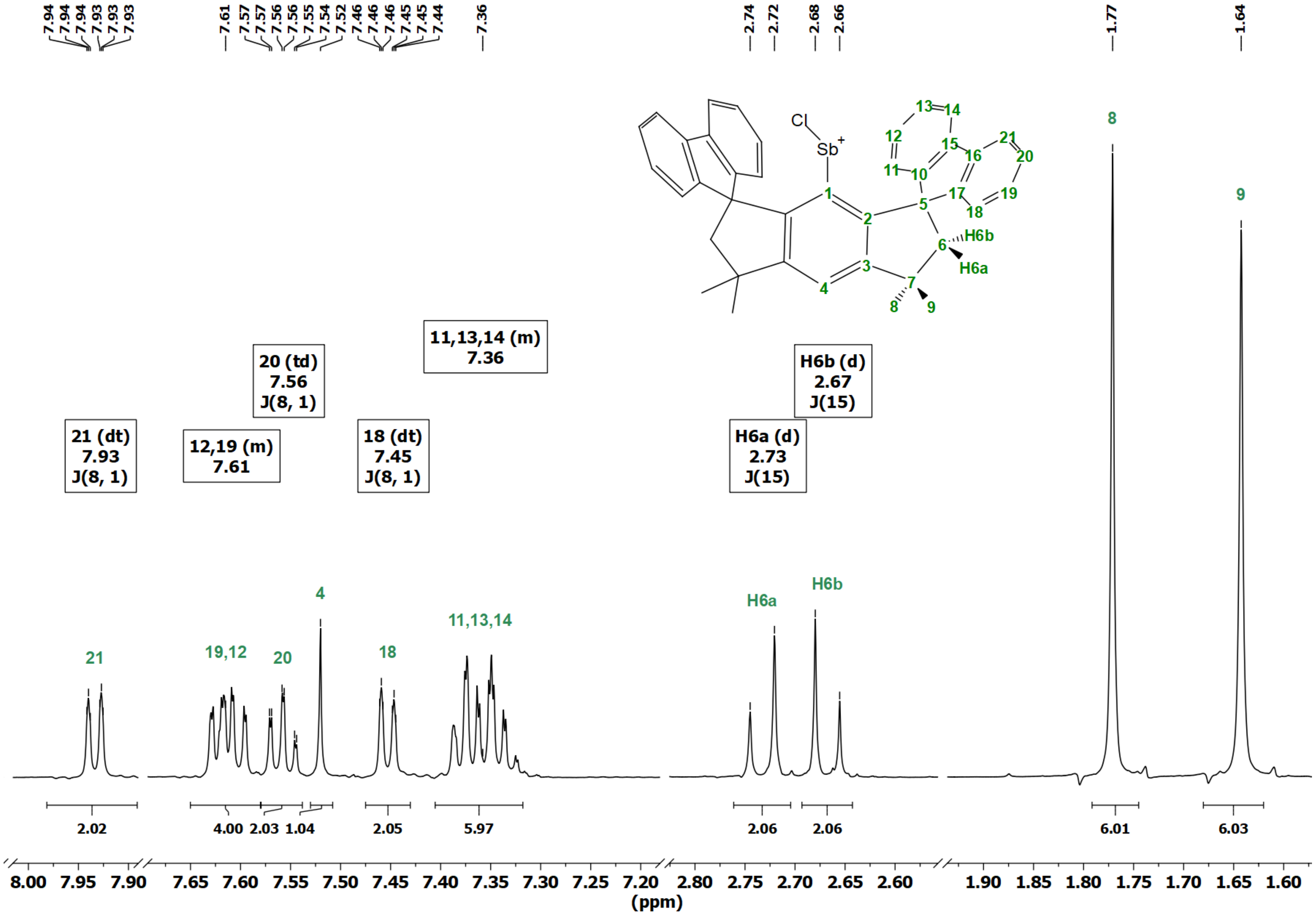

Figure S16. Detailed ^{1}H NMR (CD_2Cl_2, 600 MHz) spectrum of **2Sb**.

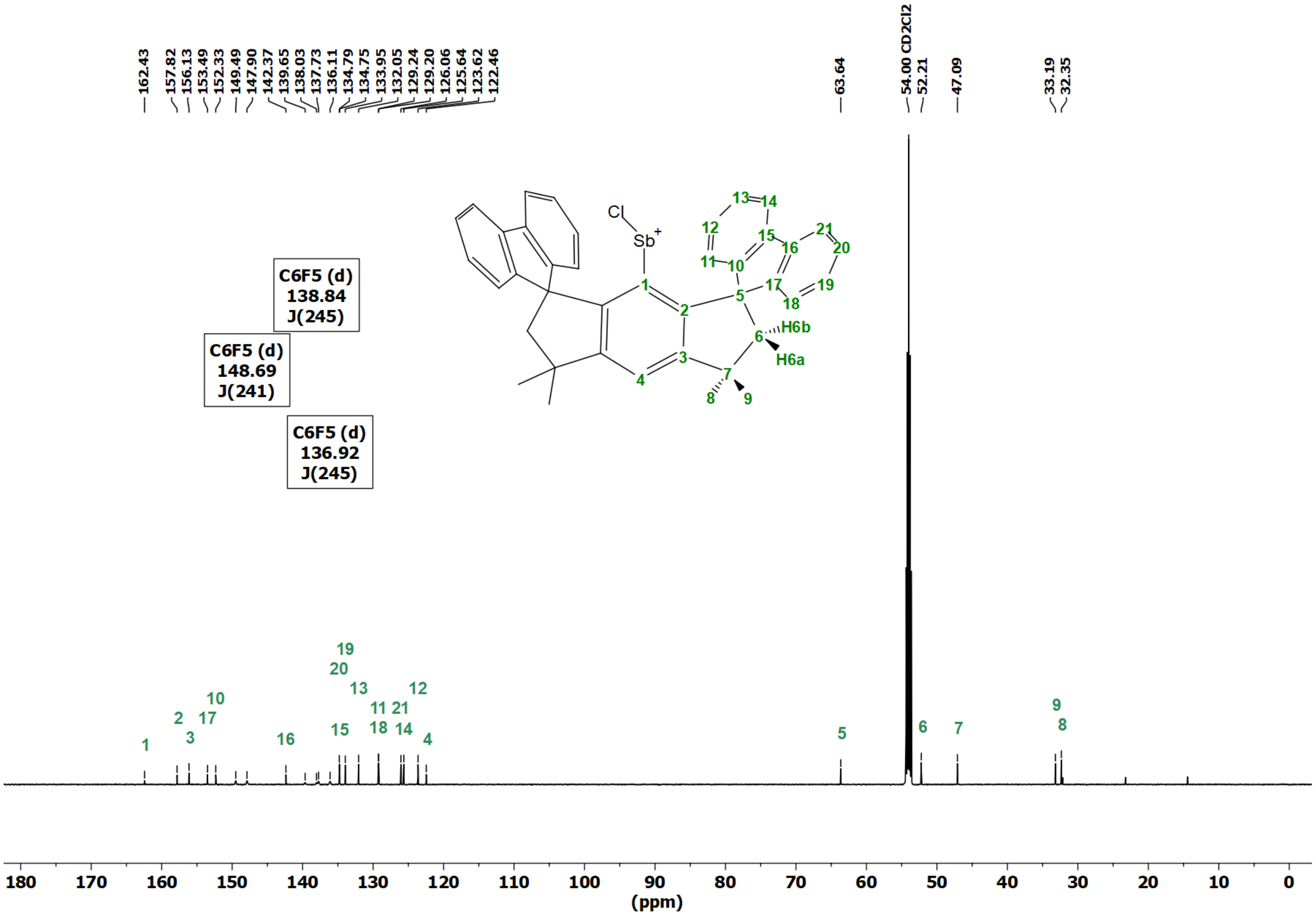

Figure S17. $^{13}C\{^{1}H\}$ NMR (CD_2Cl_2, 151 MHz) spectrum of **2Sb**.

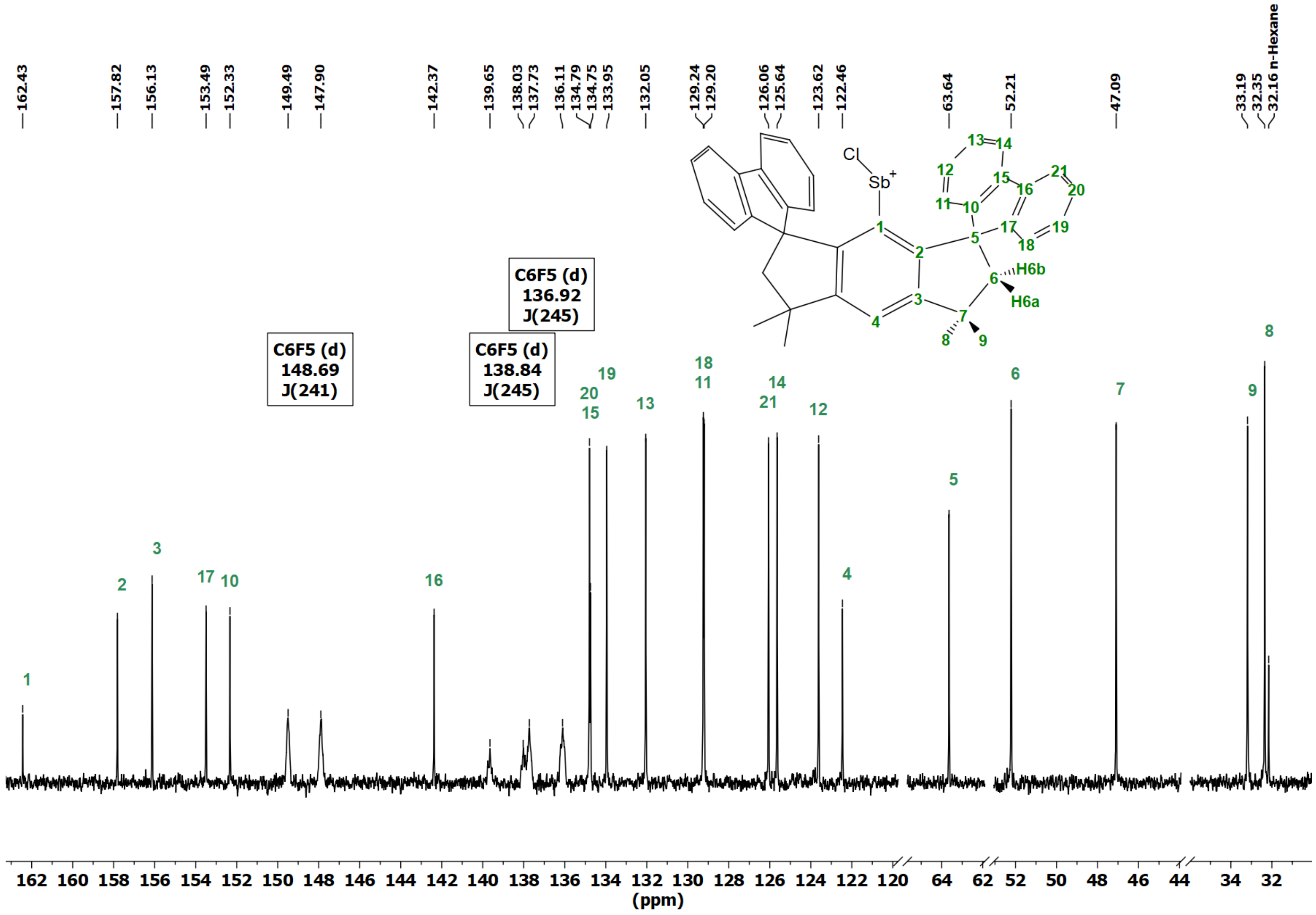

Figure S18. Detailed $^{13}C\{^{1}H\}$ NMR (CD_2Cl_2, 151 MHz) spectrum of **2Sb**.

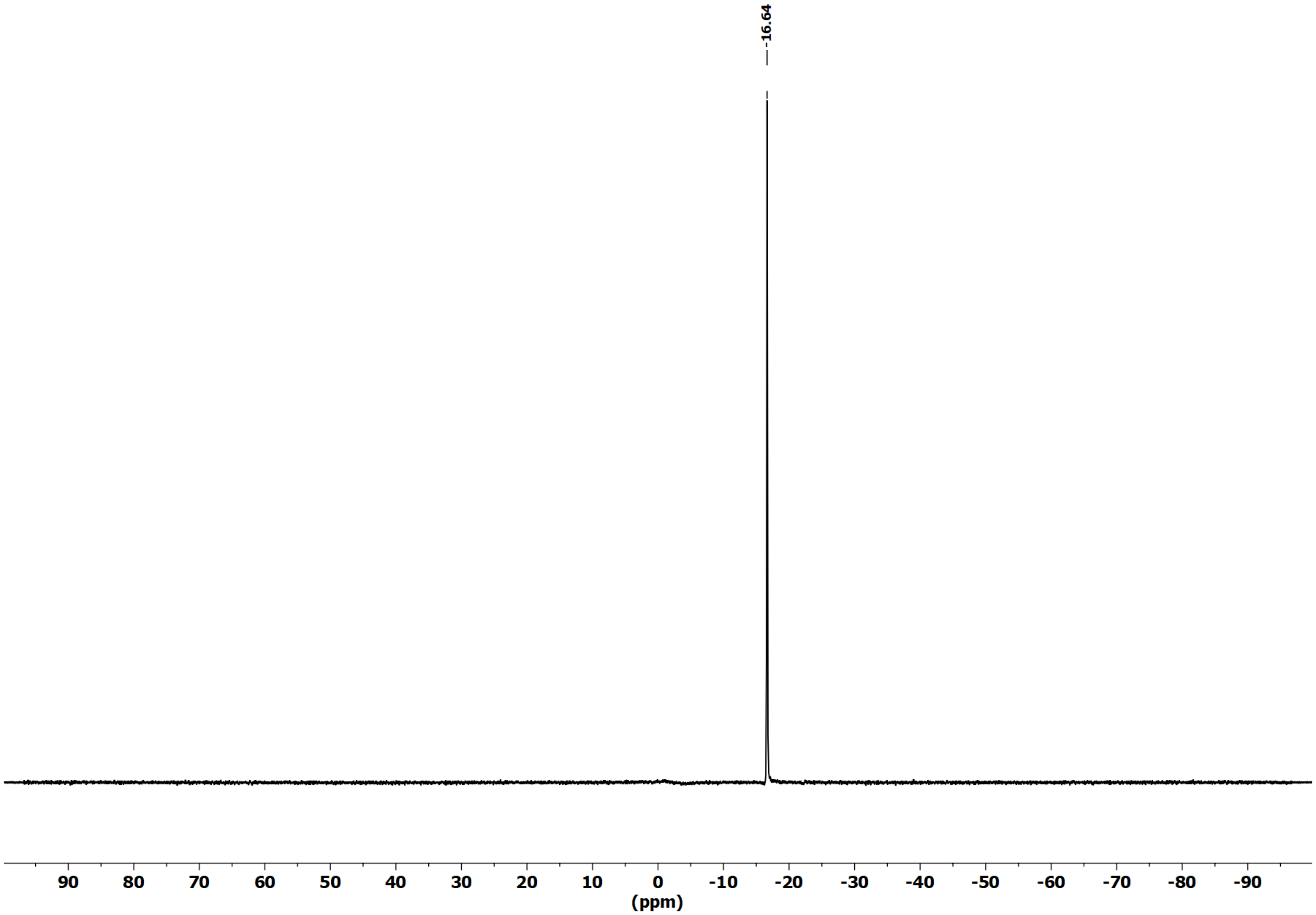

Figure S19. ^{11}B NMR (CD_2Cl_2, 193 MHz) spectrum of **2Sb**.

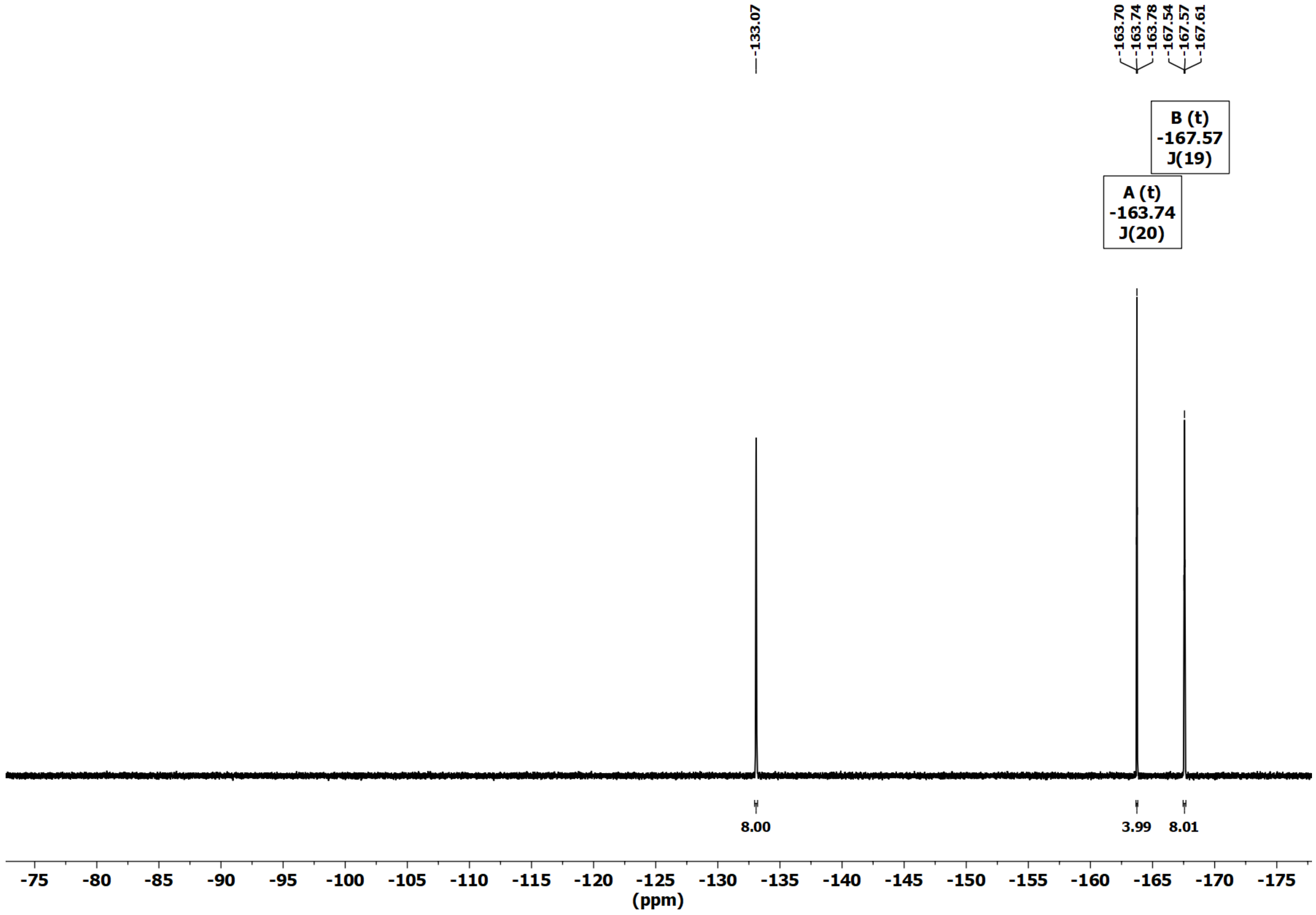

Figure S20. ^{19}F NMR (CD_2Cl_2, 565 MHz) spectrum of **2Sb**.

Synthesis and characterization of [M^{S}FluindBiCl][B(C_6F_5)$_4$] (2Bi)

To a solid mixture of M^{S}FluindBiCl$_2$ (**1Bi**) (400 mg, 0.504 mmol) and K[B(C_6F_5)$_4$] (362 mg, 0.504 mmol) was added CH_2Cl_2 (15 mL) and the reaction mixture was stirred at room temperature for 2 hours. The reaction mixture was filtered under argon to remove KCl. Hexane (20 mL) was layered over the filtrate. The resulting crystalline product was dried at reduced pressure to obtain [M^{S}FluindBiCl][B(C_6F_5)$_4$] (**2Bi**) as a dark orange crystalline solid (490 mg, 0.341 mmol, 68%).

^{1}H NMR (600 MHz, CD_2Cl_2): δ = 8.20 (s, 1H, H4), 8.03 (dt, 7.62, $^3J(^1H\text{-}^1H)$ = 8 Hz, $^4J(^1H\text{-}^1H)$ = 1 Hz, 2H, H14), 7.68 (td, $^3J(^1H\text{-}^1H)$ = 8 Hz, $^4J(^1H\text{-}^1H)$ = 1 Hz, 2H, H12), 7.57 (d, , $^3J(^1H\text{-}^1H)$ = 8 Hz, 2H, H21), 7.48 (td, $^3J(^1H\text{-}^1H)$ = 8 Hz, $^4J(^1H\text{-}^1H)$ = 1 Hz, 2H, H13), 7.40 (d, $^3J(^1H\text{-}^1H)$ = 8 Hz, 2H, H18), 7.36 (m, 4H, H11 and H20), 7.30 (td, $^3J(^1H\text{-}^1H)$ = 8 Hz, $^4J(^1H\text{-}^1H)$ = 1 Hz, 2H, H19), 2.76 (d, $^3J(^1H\text{-}^1H)$ = 15 Hz, 2H, H6a), 2.72 (d, $^3J(^1H\text{-}^1H)$ = 15 Hz, 2H, H6b), 1.79 (s, 6H, H8), 1.68 (s, 6H, H9). **^{13}C{^{1}H} NMR (151 MHz, CD_2Cl_2):** 263.6 (s, C1), 162.8 (s, C3), 162.4 (s, C2), 155.1 (s, C10), 152.7 (s, C17), 148.7 (d, br, $^1J(^{13}C\text{–}^{19}F)$ = 241 Hz, C_6F_5), 140.6 (s, C15), 138.8 (d, br, $^1J(^{13}C\text{–}^{19}F)$ = 244 Hz, C_6F_5), 136.9 (d, br, $^1J(^{13}C\text{–}^{19}F)$ = 244 Hz, C_6F_5), 134.3 (s, C13), 134.0 (s, C16), 133.0 (s, C12), 132.3 (s, C19), 129.0 (s, C20), 128.1 (s, C11), 125.4 (s, C18), 125.2 (s, C14), 123.9 (s, C21), 119.4 (s, C4), 64.3 (s, C5), 51.4 (s, C6), 47.8 (s, C7), 33.4 (s, C9), 32.9 (s, C8). **^{11}B NMR (193 MHz, CD_2Cl_2):** δ = −16.6 (s). **^{19}F NMR (565 MHz, CD_2Cl_2):** δ = −133.2 (br, 8F, *o*-C_6F_5), −163.5 (t, $^3J(^{19}F\text{-}^{19}F)$ = 20 Hz, 4F, *p*-C_6F_5), −167.4 (t, br, $^3J(^{19}F\text{-}^{19}F)$ = 20 Hz, 8F, *m*-C_6F_5). **HRMS ESI (m/z):** $[M]^+$ calculated for $C_{40}H_{33}BiCl$, 757.20693; found 757.20667. **Mp.** 224-226 °C.

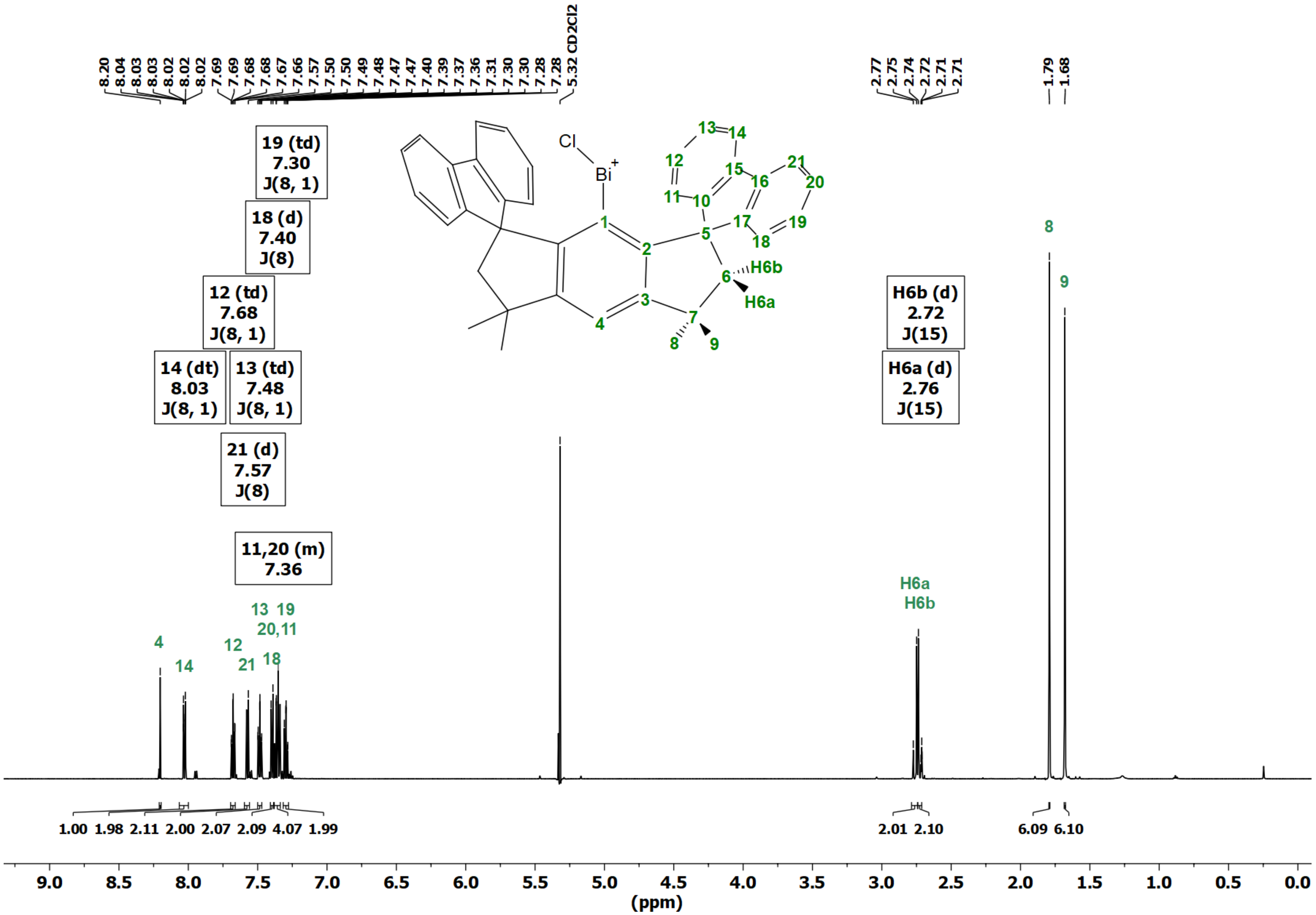

Figure S21. ^{1}H NMR (CD_2Cl_2, 600 MHz) spectrum of **2Bi**.

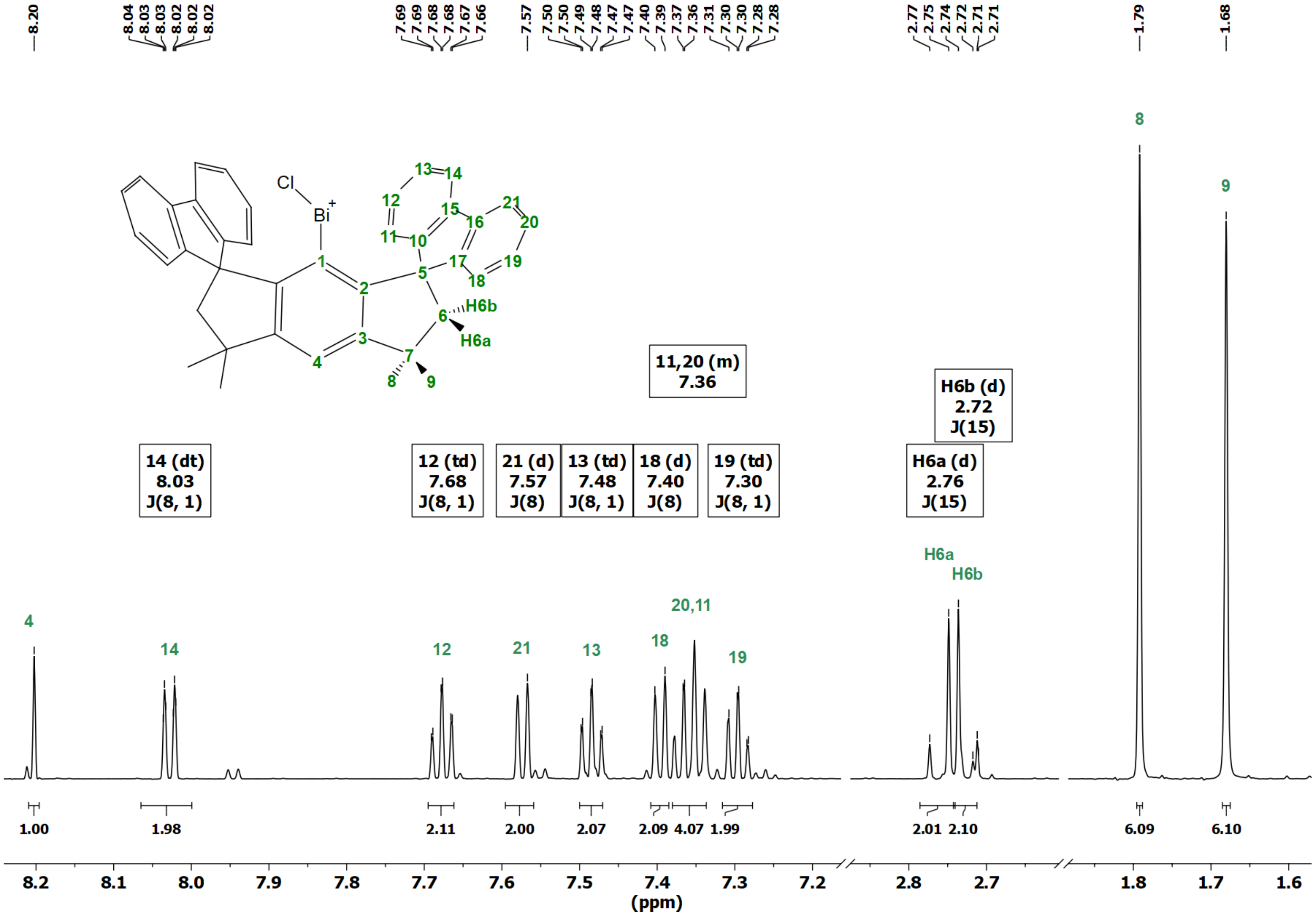

Figure S22. Detailed ^{1}H NMR (CD_2Cl_2, 600 MHz) spectrum of **2Bi**.

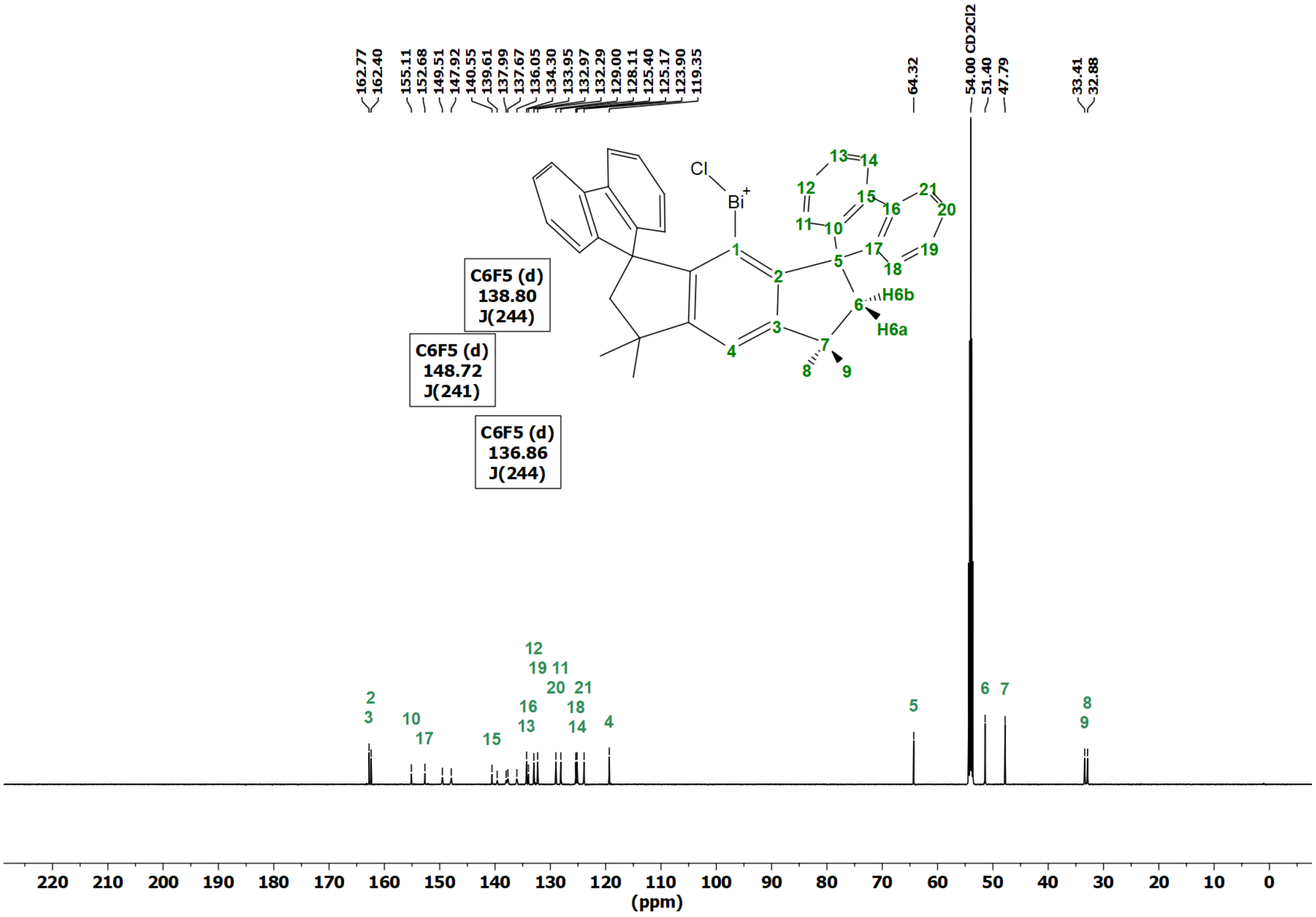

Figure S23. $^{13}C\{^1H\}$ NMR (CD_2Cl_2, 151 MHz) spectrum of **2Bi**.

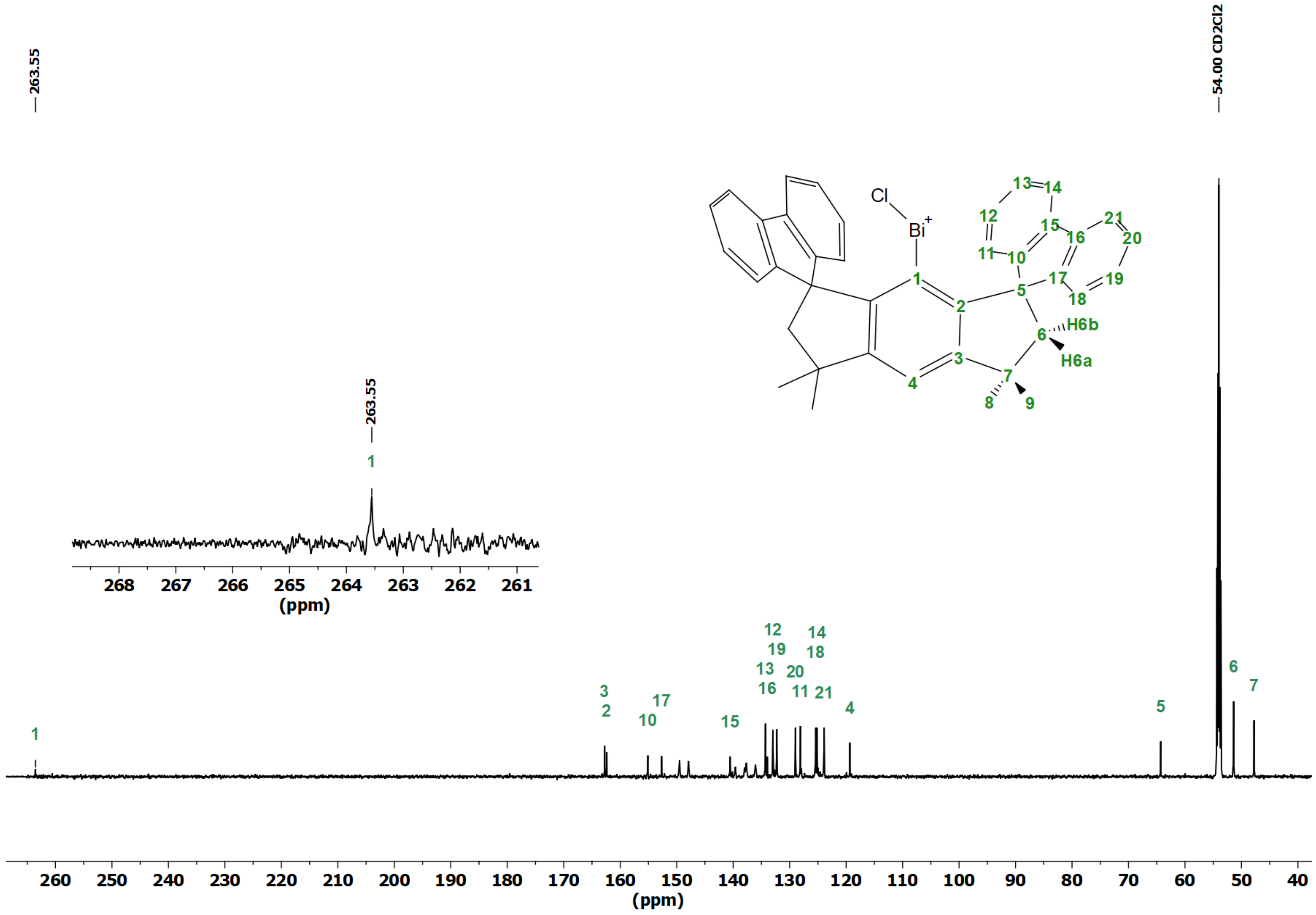

Figure S24. $^{13}C\{^1H\}$ NMR (CD_2Cl_2, 151 MHz) spectrum of **2Bi**.

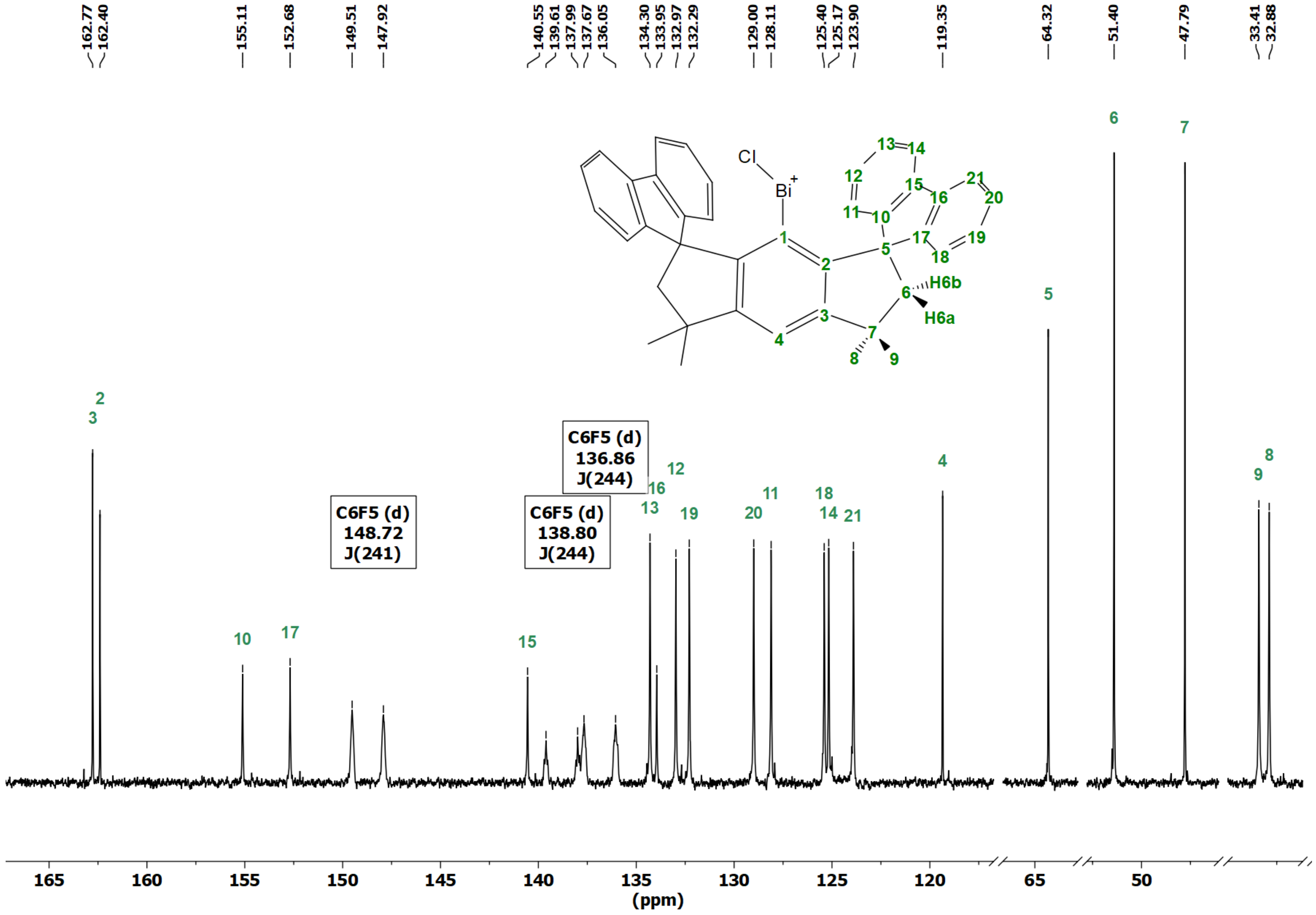

Figure S25. Detailed $^{13}C\{^{1}H\}$ NMR (CD_2Cl_2, 151 MHz) spectrum of **2Bi**.

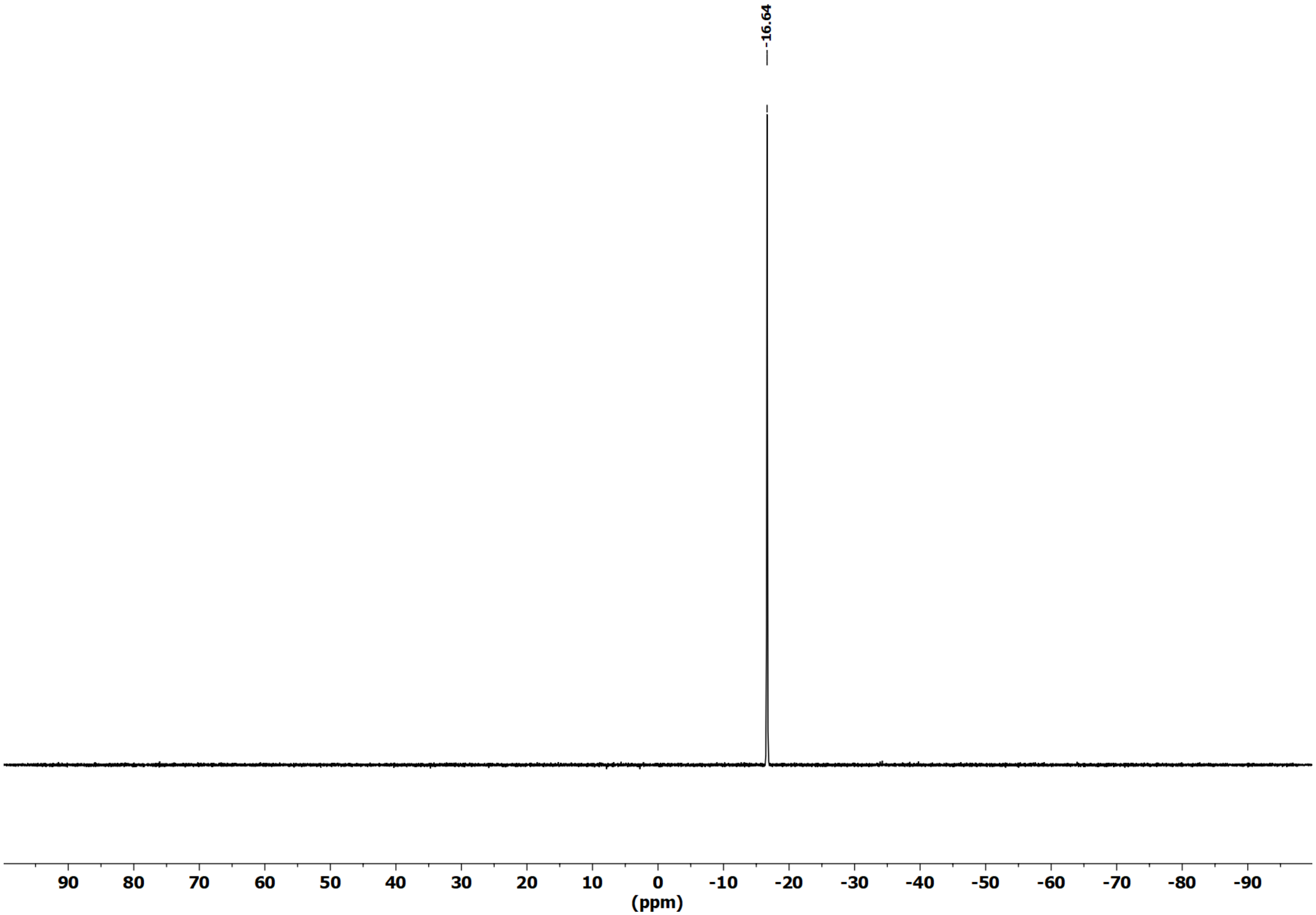

Figure S26. ^{11}B NMR (CD_2Cl_2, 193 MHz) spectrum of **2Bi**.

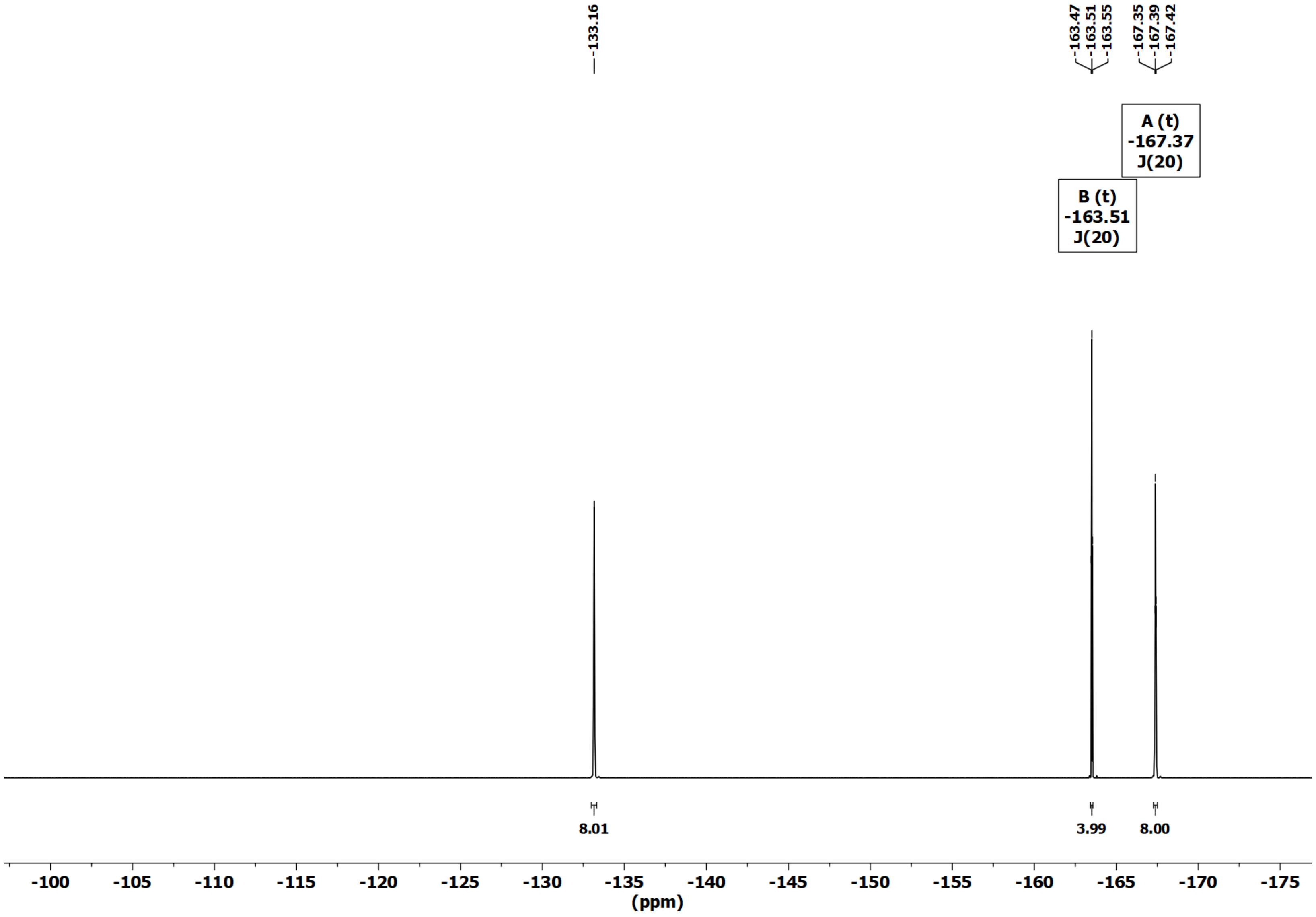

Figure S27. ^{19}F NMR (CD_2Cl_2, 565 MHz) spectrum of **2Bi**.

Synthesis and characterization of M^SFluindAsH_2 (3)

Method A

To a solution of [M^SFluindAsCl][B(C_6F_5)$_4$] (**2As**, 100 mg, 0.767 mmol) in dichloromethane (5 mL) was added triethylsilane (12.3 µL, 0.767 mmol, 1.00 Eq) and the reaction mixture was stirred for 30 minutes at room temperature. NMR spectra of the crude reaction mixture indicated formation of the arylarsenic dihydride **3** and after evaporation of the solvent the solid product was suspended in toluene (20 mL) and filtered. The filtrate was recrystallized from boiling toluene to obtain M^SFluindAsH_2 (**3**) as a colourless crystalline solid (21.0 mg, 0.356 mmol, 46%).

Crystals suitable for X-ray structure determination were grown from a hot toluene solution.

Method B

M^SFluindAsCl_2 (250 mg, 0.379 mmol) was suspended in diethylether (10 mL). The suspension was cooled to –50 °C and $LiAlH_4$ (43.2 mg, 1.14 mmol, 3.00 eq.) was added as a solid. The reaction mixture was stirred for an additional 2 hours at –20 °C. After this, the solvent was removed under reduced pressure. The residual solid was dissolved in toluene (30 mL) and filtered through a PTFE syringe filter. The solvent was removed under vacuum to obtain **3** as a colourless solid (127 mg, 0.215 mmol, 57%).

^{1}H NMR (600 MHz, C_6D_6): 7.42 (d, ^3J = 7 Hz, 4H, H14 and H21), 7.31 (s, 1H, H4), 7.14 (d, ^3J = 8 Hz, 4H, H11 and H18), 7.07 (td, ^3J = 7 Hz, ^4J = 1 Hz, 4H, H13 and H20), 6.99 (td, ^3J = 7 Hz, ^4J = 1 Hz, 4H, H12 and H 19), 2.39 (s, 4H, H6a and H6b), 1.68 (s, 2H, H22 and H23), 1.47 (s, 12H, H8 and H9). **^{13}C{^{1}H} NMR (151 MHz, C_6D_6)** δ 155.7 (s, C3), 154.1 (s, C10 and C17), 148.6 (s, C2), 141.1 (s, C15 and C16), 129.6 (s, C1), 128.0 (s, C12 and C19), 127.4 (s, C13 and C20), 124.7 (s, C11 and C18), 120.2 (s, C14 and C21), 116.6 (s, C4), 64.9 (s, C5), 57.4 (s, C6), 42.9 (s, C7), 32.8 (s, C8 and C9). **HRMS ESI (m/z):** $[M+K]^+$ calculated for $C_{40}H_{35}AsK$, 629.15863; found 629.15858. **Mp.** 280 °C (decomposition).

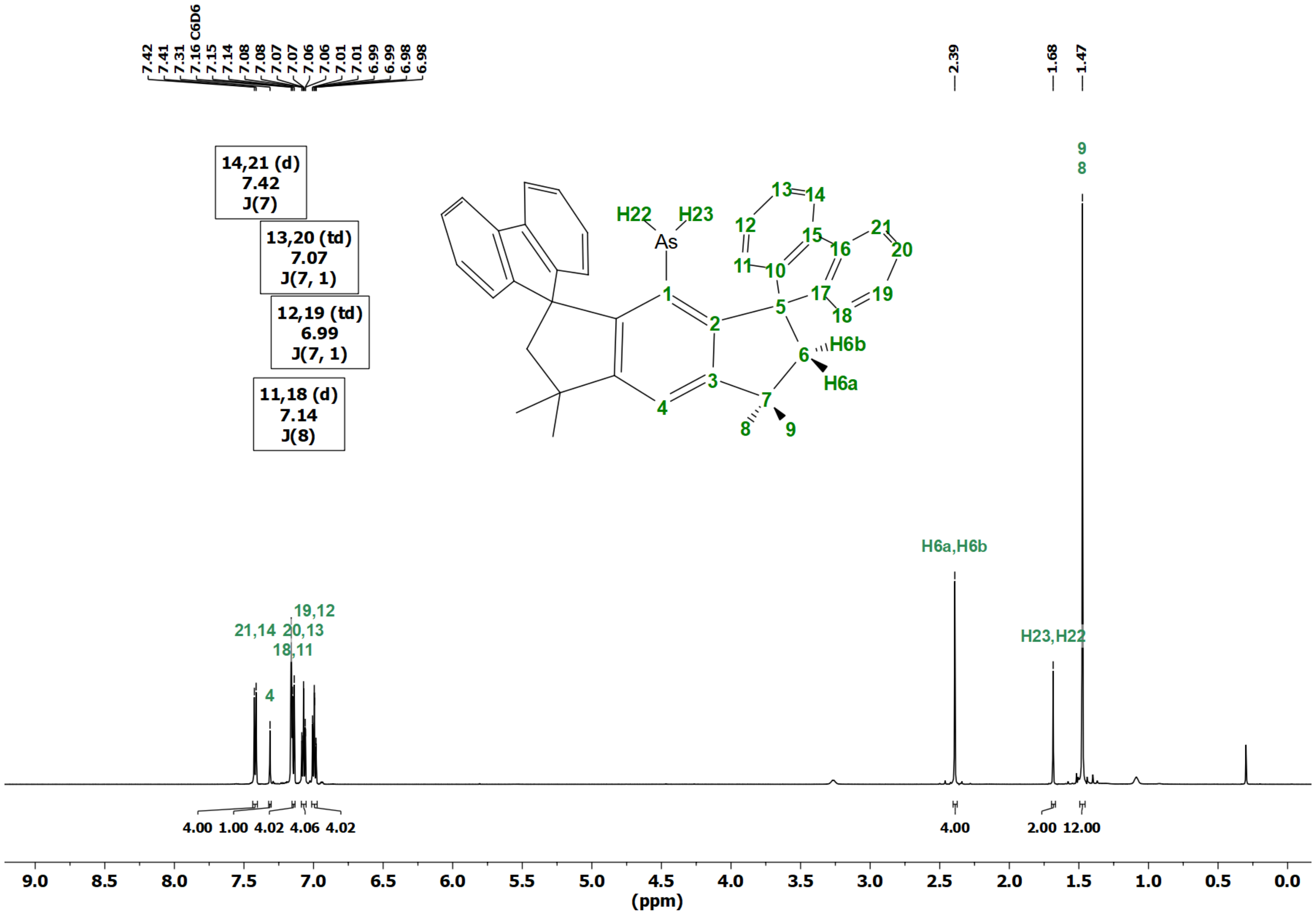

Figure S28. ^{1}H NMR (C_6D_6, 600 MHz) spectrum of **3**.

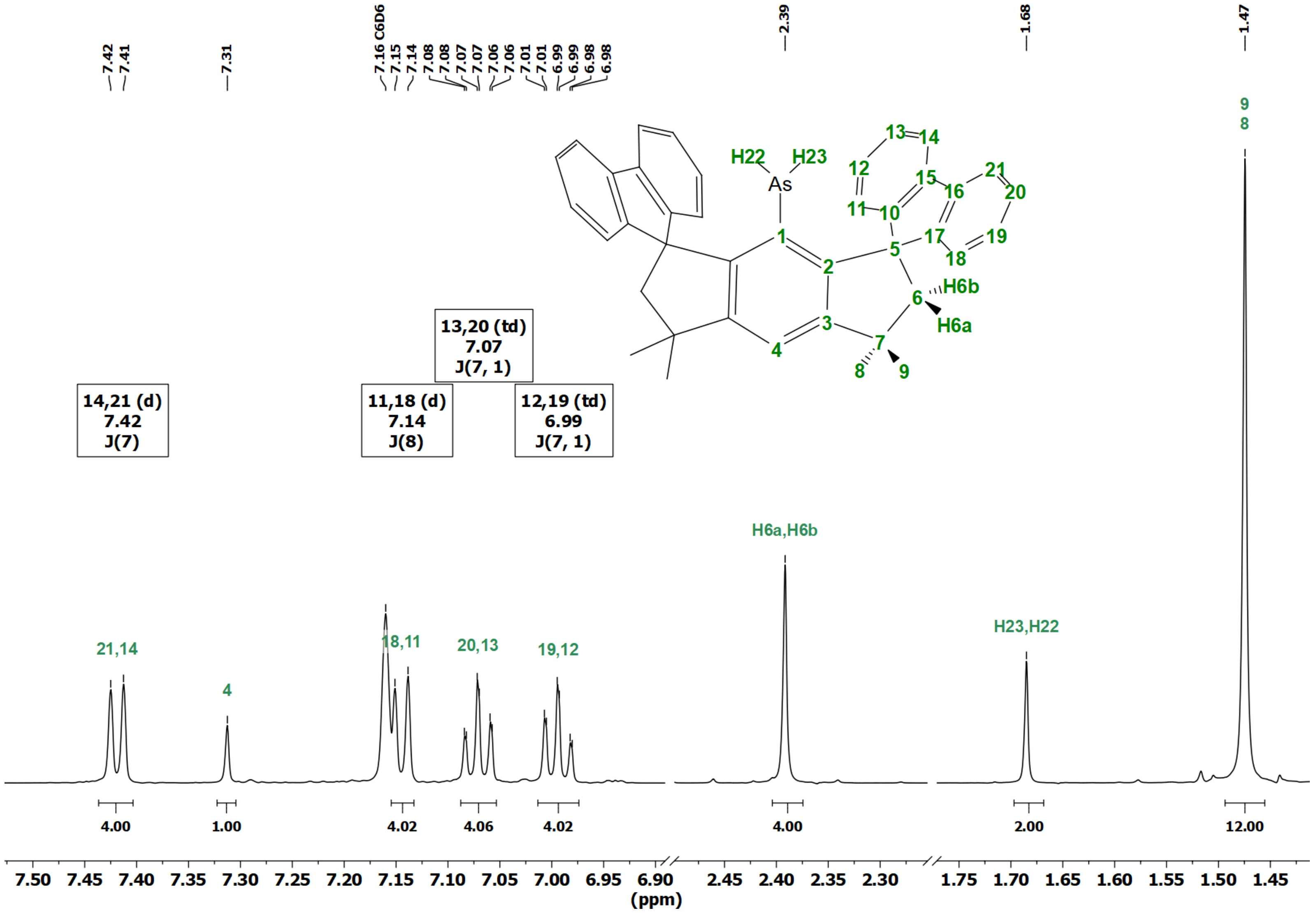

Figure S29. Detailed ^{1}H NMR (C_6D_6, 600 MHz) spectrum of **3**.

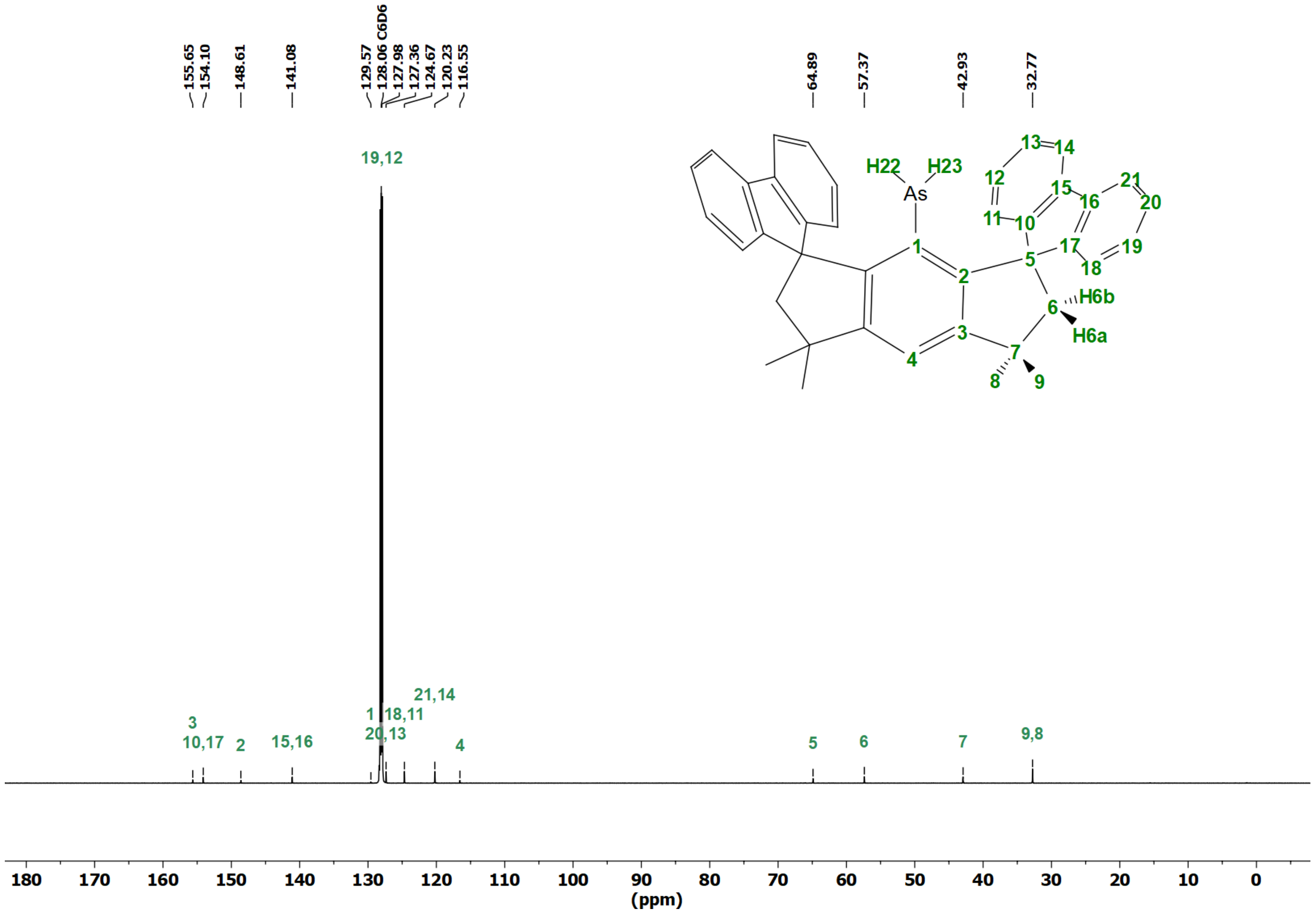

Figure S30. $^{13}C\{^{1}H\}$ NMR (C_6D_6, 151 MHz) spectrum of **3**.

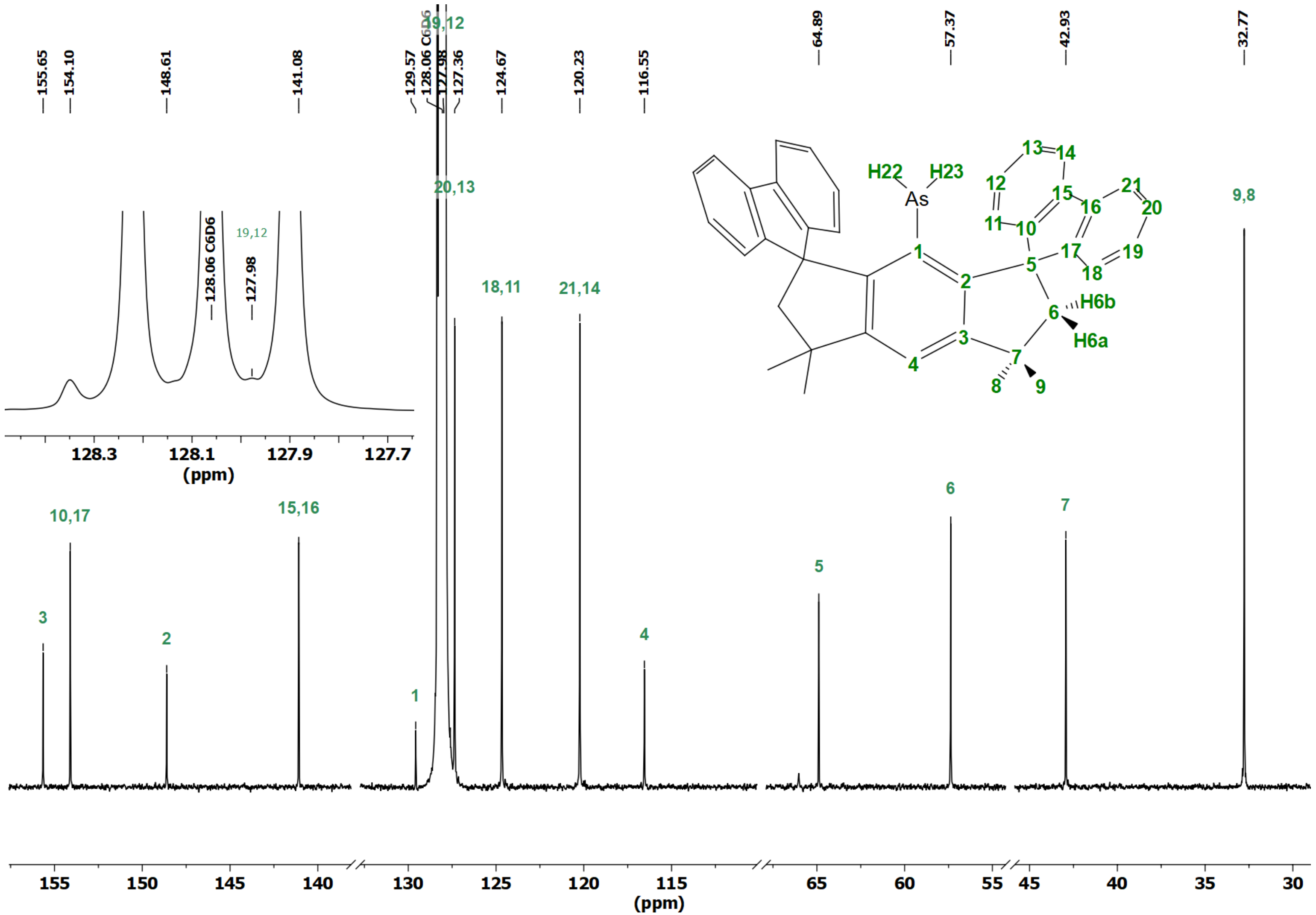

Figure S31. Detailed $^{13}C\{^{1}H\}$ NMR (CD_6Cl_6, 151 MHz) spectrum of **3**.

Synthesis and characterization of [M^{S}FluindSbH][B(C_6F_5)$_4$] (4)

To a solution of [M^{S}FluindSbCl][B(C_6F_5)$_4$] (**2Sb**, 200 mg, 0.148 mmol) in 1,2-dichlorobenzene (8 mL) was added triethylsilane (71.0 µL, 0.296 mmol, 3.00 Eq.) and reaction mixture was stirred at room temperature for 4 hours and layered with hexane (15 mL). The resulting crystalline solid was decanted, washed with hexane (2 × 5 mL) and dried under reduced pressure to obtain [M^{S}FluindSbH][B(C_6F_5)$_4$] (**4**) as a yellow crystalline solid (137 mg, 0.104 mmol, 70%).

^{1}H NMR (600 MHz, CD_2Cl_2): δ = 18.06 (s, 1H, H22), 7.73 (d, ($^3J(^1H-^1H)$ = 8 Hz, 2H, H14, H21), 7.44 (m, 5H, H4, H13 and H20), 7.36 (m, 8H, H11, H12, H18 and H19), 2.70 (s, 4H, H6a, H6b), 1.70 (s, 12H, H8 and H9). **$^{13}C\{^1H\}$ NMR (151 MHz, CD_2Cl_2):** δ = 157.4 (s, C2), 154.8 (s, C3), 154.3 (s, C10 and C17), 148.7 (d, br, $^1J(^{13}C-^{19}F)$ = 240 Hz, C_6F_5), 144.2 (s, C15 and C16), 138.8 (d, br, $^1J(^{13}C-^{19}F)$ = 245 Hz, C_6F_5), 136.9 (d, br, $^1J(^{13}C-^{19}F)$ = 241 Hz, C_6F_5), 133.1 (s, C1), 132.8 (s, C12 and C19), 131.3 (s, C13 and C20), 127.1 (s, C11 and C18), 123.2 (s, C14 and C21), 121.4 (s, C4), 64.0 (s, C5), 50.4 (s, C6), 46.7 (s, C7), 32.9 (s, C8 and C9). **^{11}B NMR (193 MHz, CD_2Cl_2):** δ = −16.6 (s). **^{19}F NMR (565 MHz, CD_2Cl_2):** δ = −133.8 (br, 8F, *o*-C_6F_5), −164.0 (t, $^3J(^{19}F-^{19}F)$ = 20 Hz, 4F, *p*-C_6F_5), −168.4 (t, br, $^3J(^{19}F-^{19}F)$ = 19 Hz, 8F, *m*-C_6F_5). **HRMS ESI (m/z):** $[M]^+$ calculated for $C_{40}H_{34}Sb$, 635.16932; found 635.16882. **Mp.** 170-175 °C (decomposition)

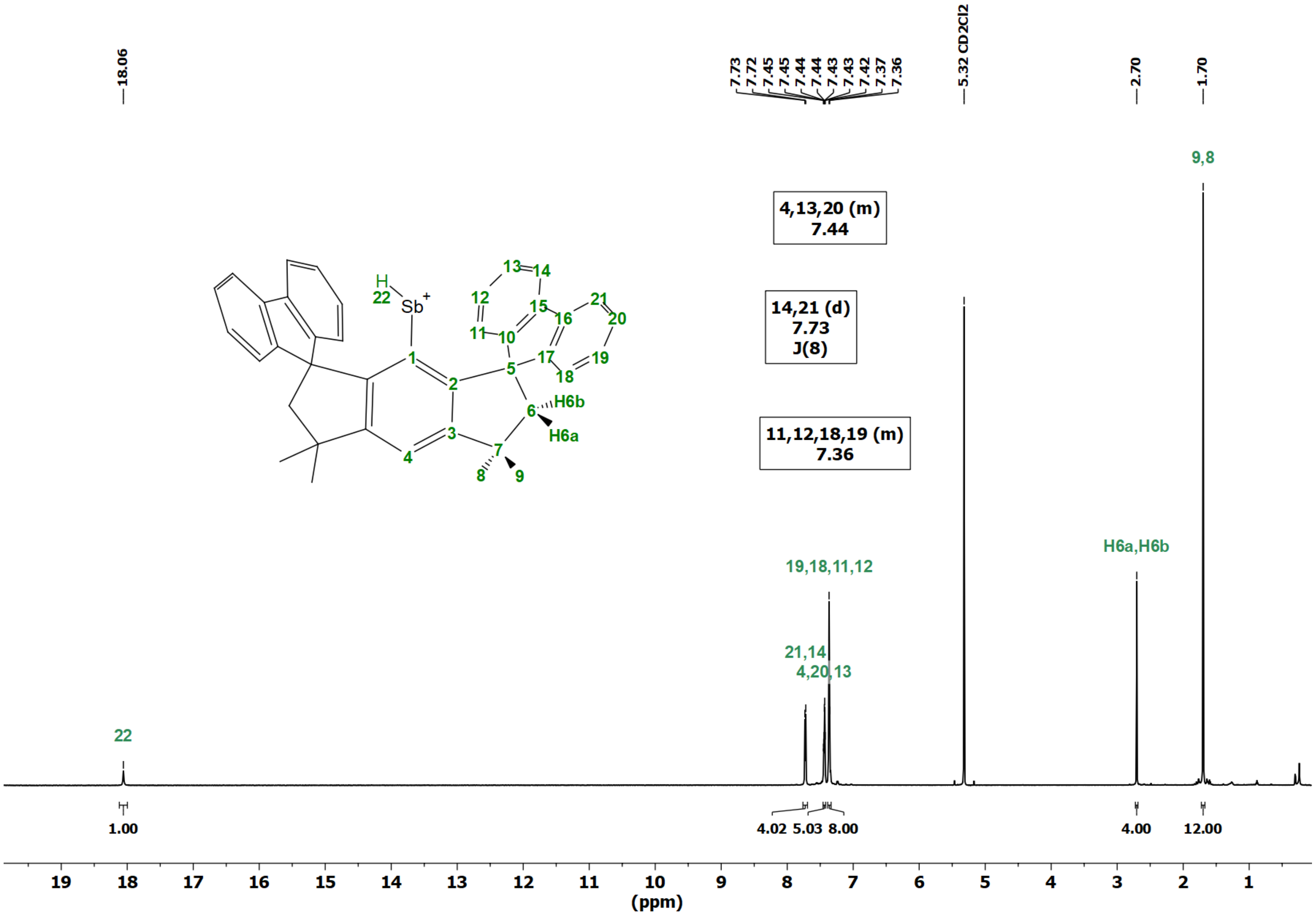

Figure S32. ^{1}H NMR (CD_2Cl_2, 600 MHz) spectrum of **4**.

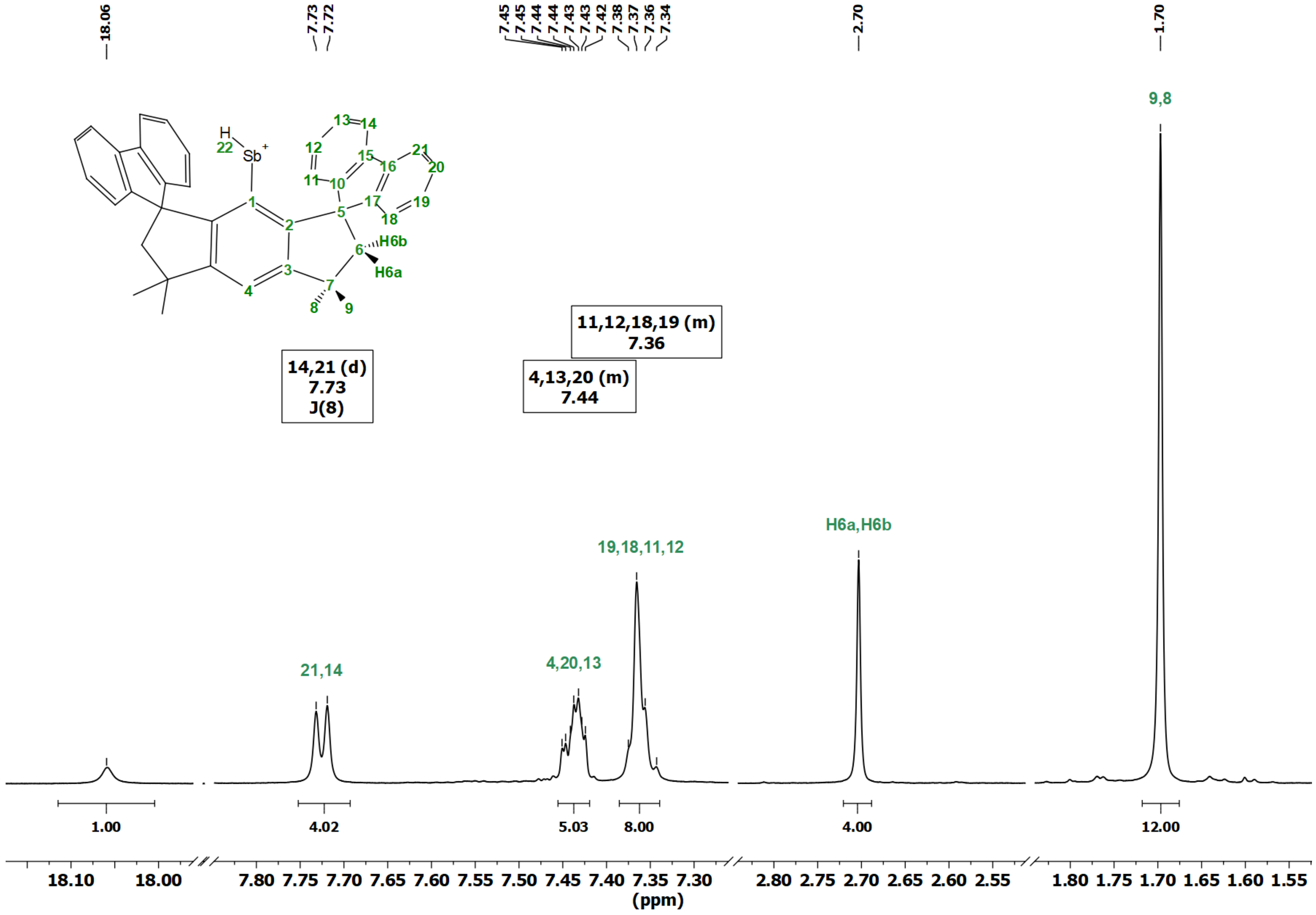

Figure S33. Detailed ^{1}H NMR (CD_2Cl_2, 600 MHz) spectrum of **4**.

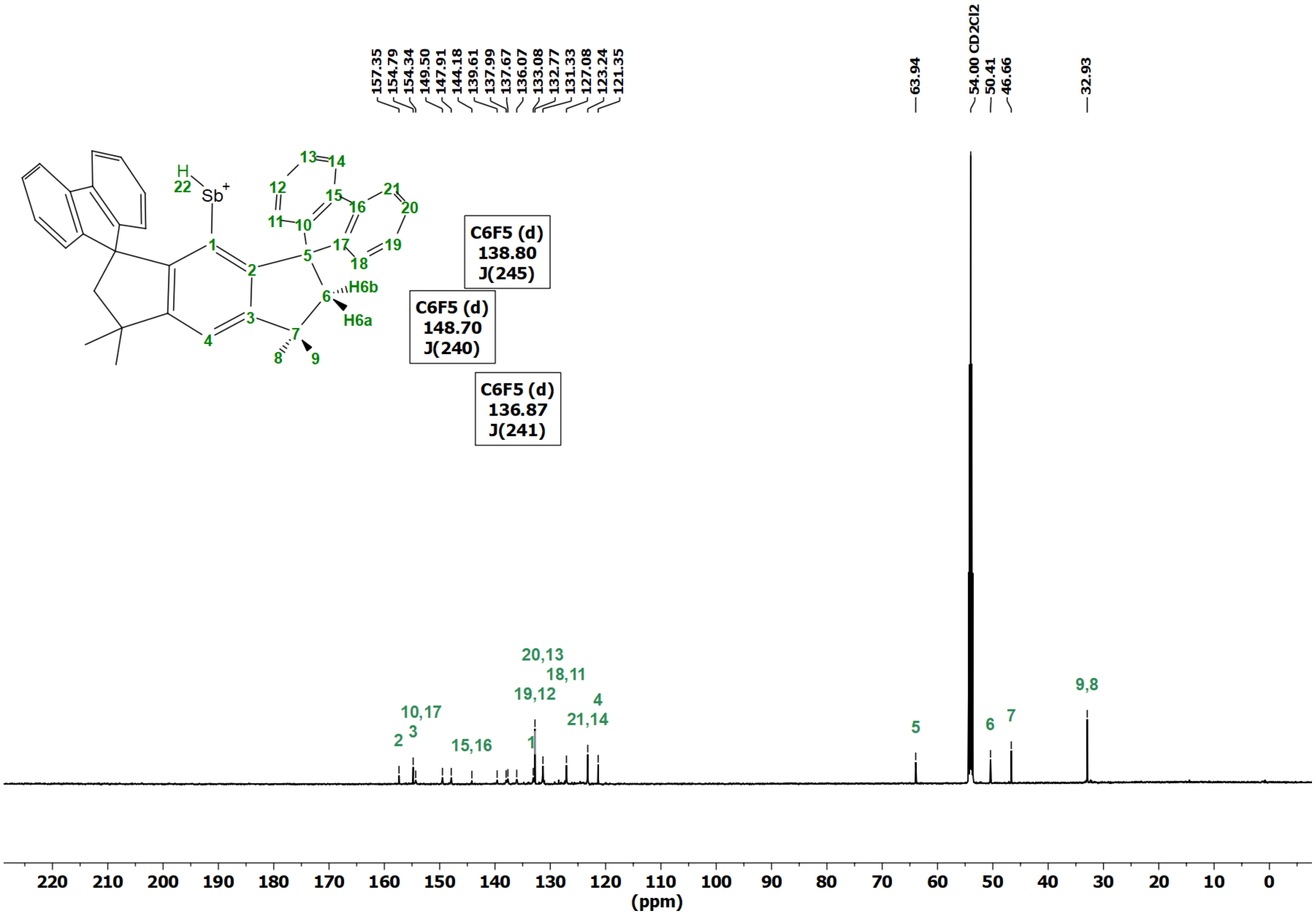

Figure S34. $^{13}C\{^{1}H\}$ NMR (CD_2Cl_2, 151 MHz) spectrum of **4**.

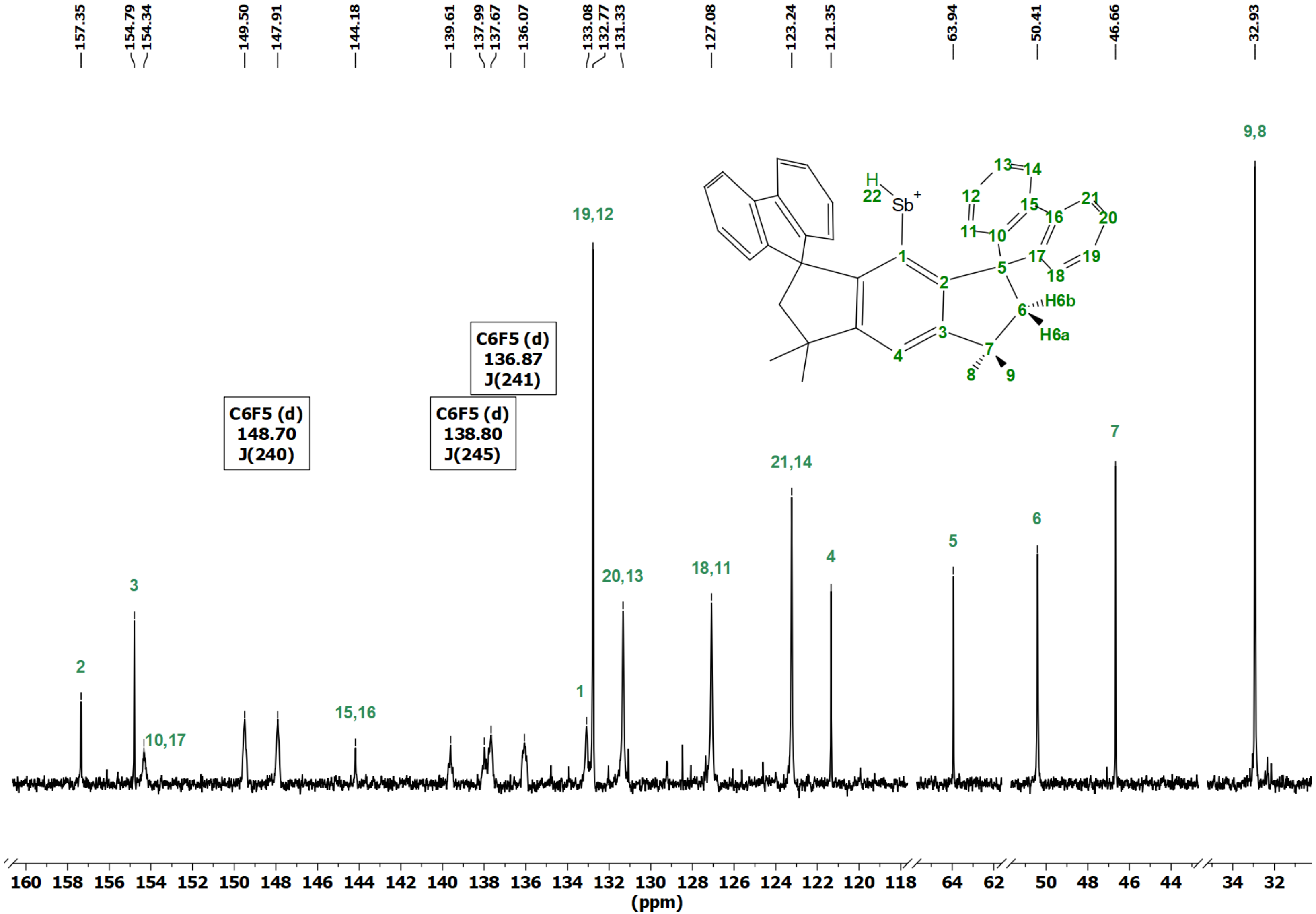

Figure S35. Detailed $^{13}C\{^{1}H\}$ NMR (CD_2Cl_2, 151 MHz) spectrum of **4**.

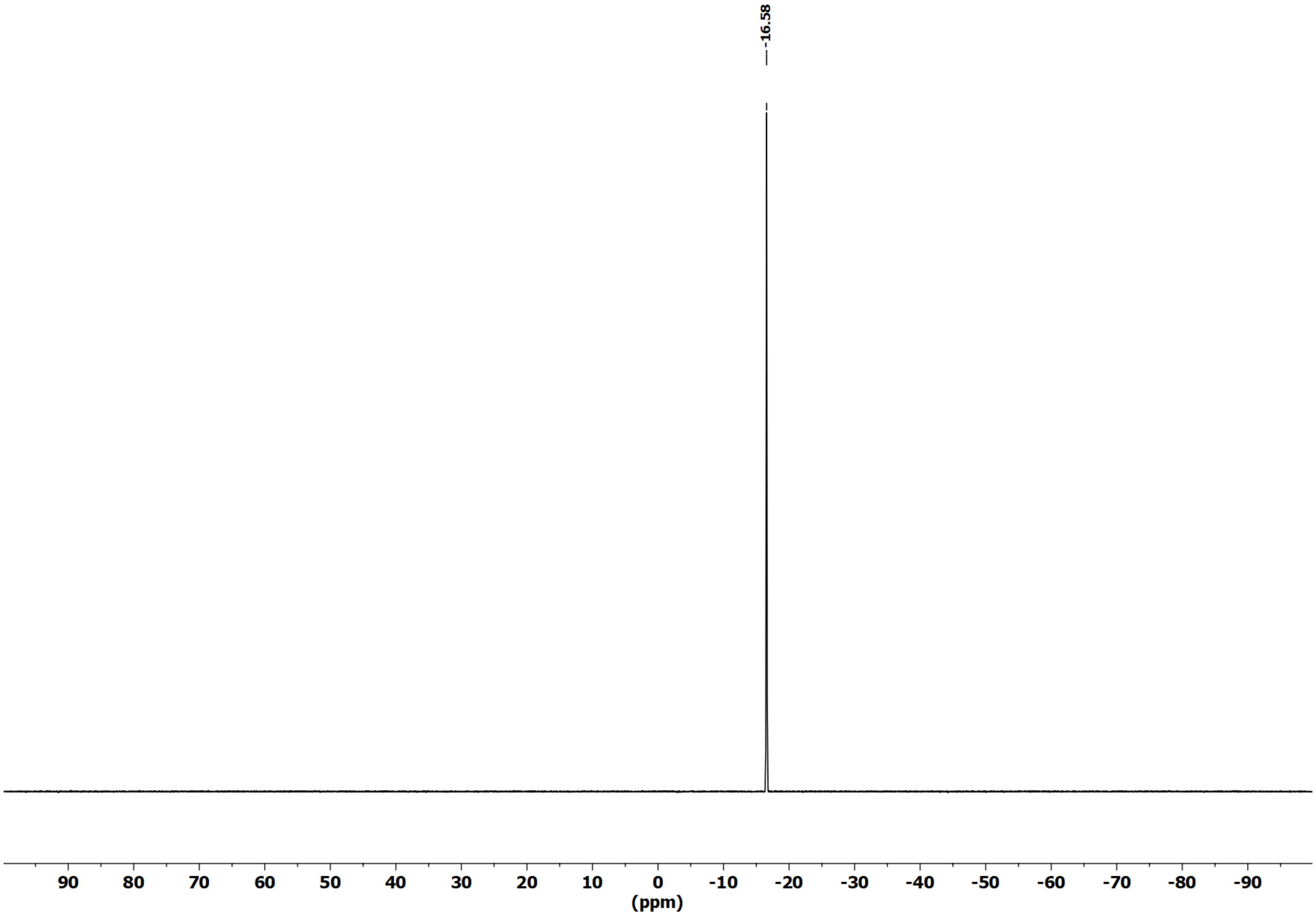

Figure S36. ^{11}B NMR (CD_2Cl_2, 193 MHz) spectrum of **4**.

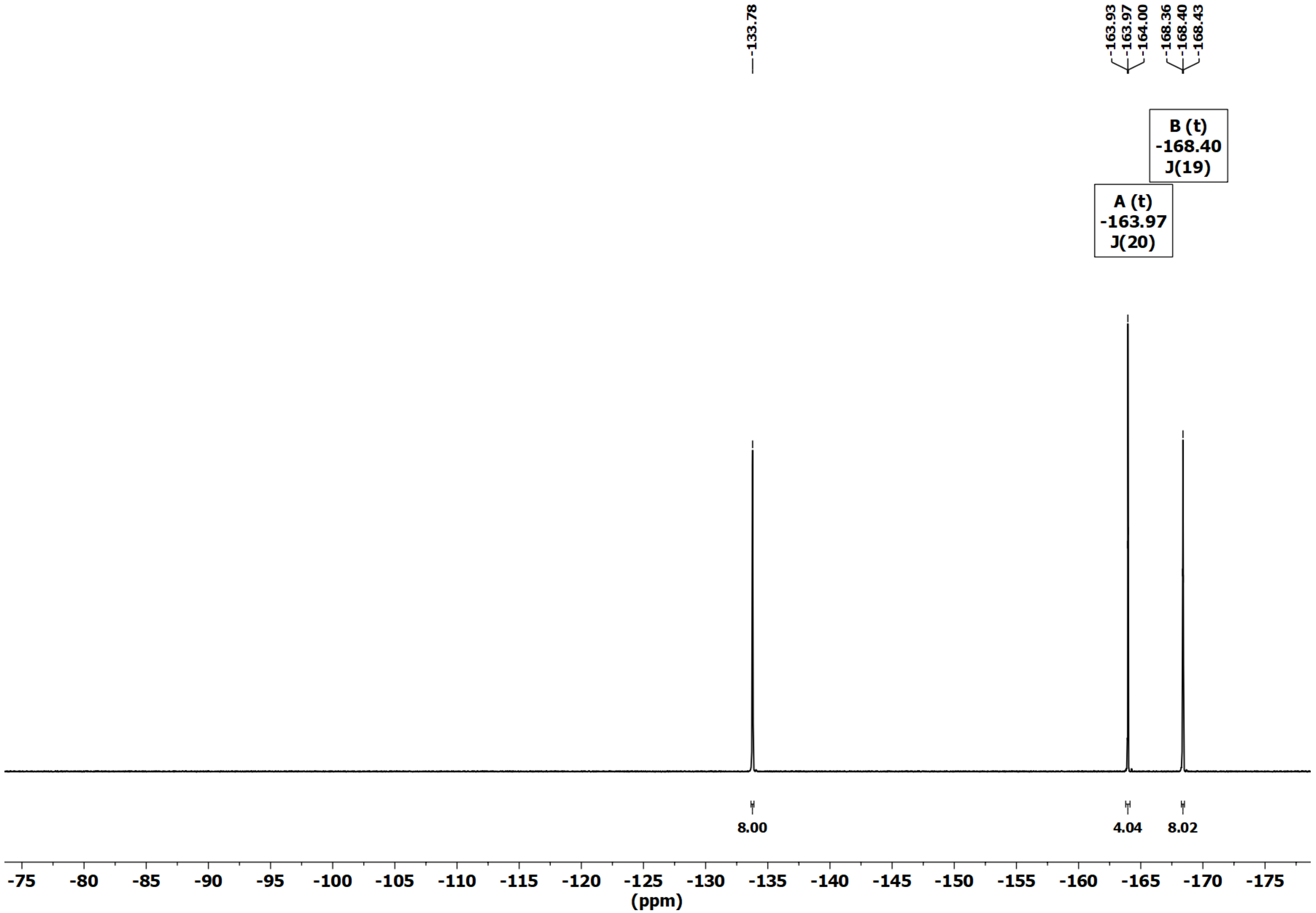

Figure S37. ^{19}F NMR (CD_2Cl_2, 565 MHz) spectrum of **4**

Synthesis and characterization of [M^{S}FluindSbInMe$_2$][B(C$_6$F$_5$)$_4$] (5In)

In a J. Young NMR Tube [M^{S}FluindSbH][B(C$_6$F$_5$)$_4$] (**4**, 15.0 mg, 11.4 µmol) and Me$_3$In (1.82 mg, 11.4 µmol) were dissolved in C$_6$D$_6$ (0.5 mL) at room temperature. The resulting colorless solution was layered with *n*-hexane (0.5 mL). The crystalline product was decanted, washed with *n*-hexane (3 × 0,5 mL) and dried at reduced pressure to obtain **5In** as a colorless crystalline solid (9.10 mg, 6.54 µmol, 57%).

^{1}H NMR (601 MHz, CD$_2$Cl$_2$): δ = 7.70 (d, ^3J(^{1}H-^{1}H) = 8 Hz, 2H, Ar-H), 7.67 (d, ^3J(^{1}H-^{1}H) = 7 Hz, 2H, Ar-H), 7.51 (s, 1H, H4), 7.42 – 7.34 (m, 8H, Ar-H), 7.30 (t, ^3J(^{1}H-^{1}H) = 8 Hz, 2H, Ar-H), 7.20 (d, ^3J(^{1}H-^{1}H) = 8 Hz, 2H, Ar-H), 2.46 (d, J = ^3J(^{1}H-^{1}H) = 14 Hz, 2H, H6a), 2.41 (d, ^3J(^{1}H-^{1}H) = 14 Hz, 2H, H6b), 1.61 (s, 6H, H8), 1.58 (s, 6H, H9), 0.09 (s, br, 6H, H22 and H23). **^{13}C{^{1}H} NMR (151 MHz, CD$_2$Cl$_2$):** δ = 157.8 (s, C$_{Ar}$), 156.2 (s, C$_{Ar}$), 155.4 (s, C$_{Ar}$), 149.7 (s, C$_{Ar}$), 148.7 (d, br, ^1J(^{13}C–^{19}F) = 240 Hz, *C*$_6$F$_5$), 140.6 (s, C$_{Ar}$), 138.7 (d, br, ^1J(^{13}C–^{19}F) = 232 Hz, *C*$_6$F$_5$), 136.9 (d, br, ^1J(^{13}C–^{19}F) = 241 Hz, *C*$_6$F$_5$), 130.2 (s, C$_{Ar}$), 129.2 (s, C$_{Ar}$), 128.9 (s, C$_{Ar}$), 126.7 (s, C$_{Ar}$), 125.6 (s, C$_{Ar}$), 121.8 (s, C$_{Ar}$), 119.8 (s, C$_{Ar}$), 66.3 (s, C5), 58.3 (s, C6), 43.5 (s, C7), 33.0 (s, C9), 32.6 (s, C8). C22 and C23 were not observed. **^{11}B NMR (193 MHz, CD$_2$Cl$_2$):** δ = −16.0 (s). **^{19}F NMR (565 MHz, CD$_2$Cl$_2$):** δ = −133.1 (br, 8F, *o*-C$_6$F$_5$), −163.5 (t, ^3J(^{19}F-^{19}F) = 21 Hz, 4F, *p*-C$_6$F$_5$), −167.5 (t, br, ^3J(^{19}F-^{19}F) = 20 Hz, 8F, *m*-C$_6$F$_5$).

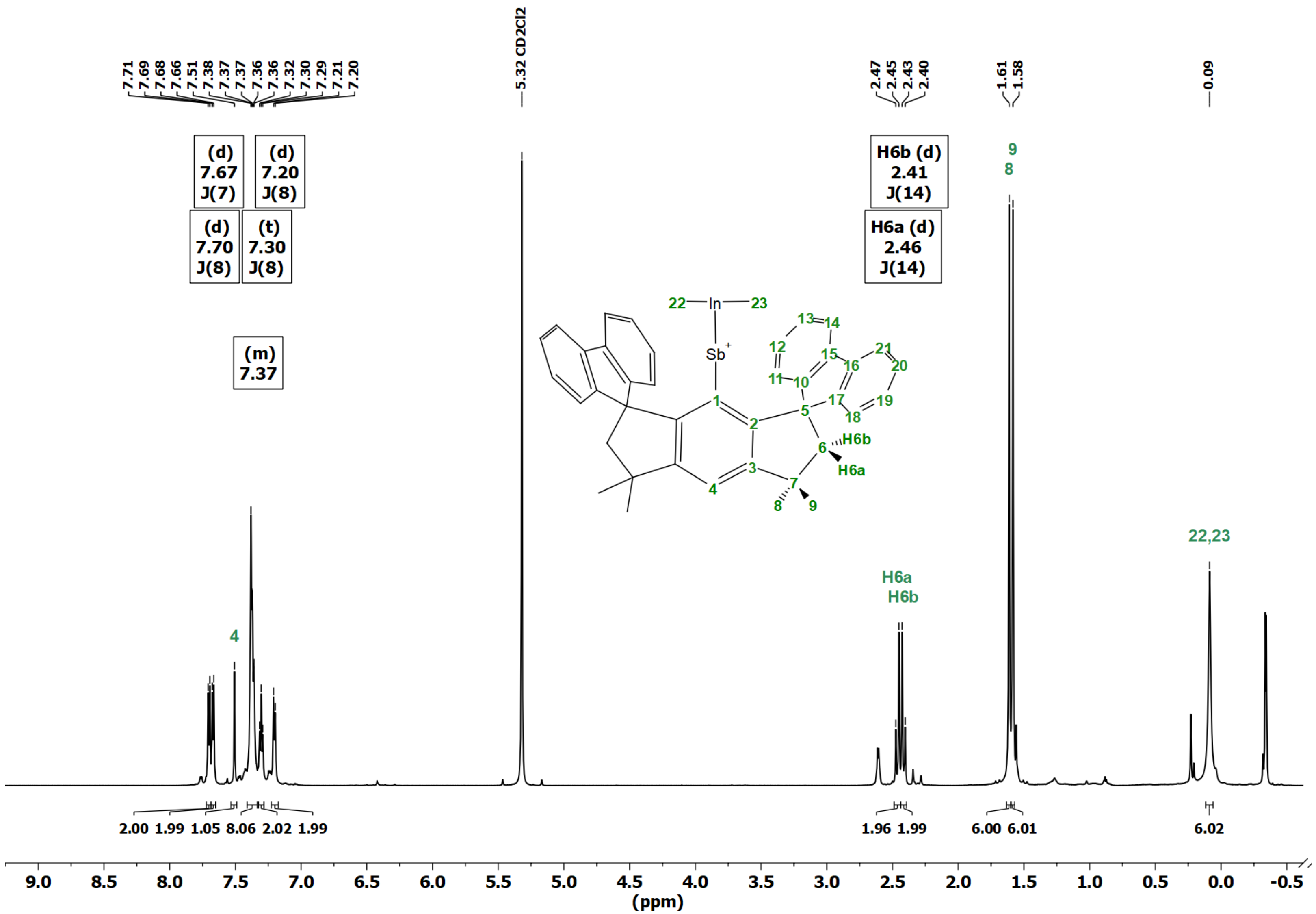

Figure S38. ^{1}H NMR (CD_2Cl_2, 600 MHz) spectrum of **5In**.

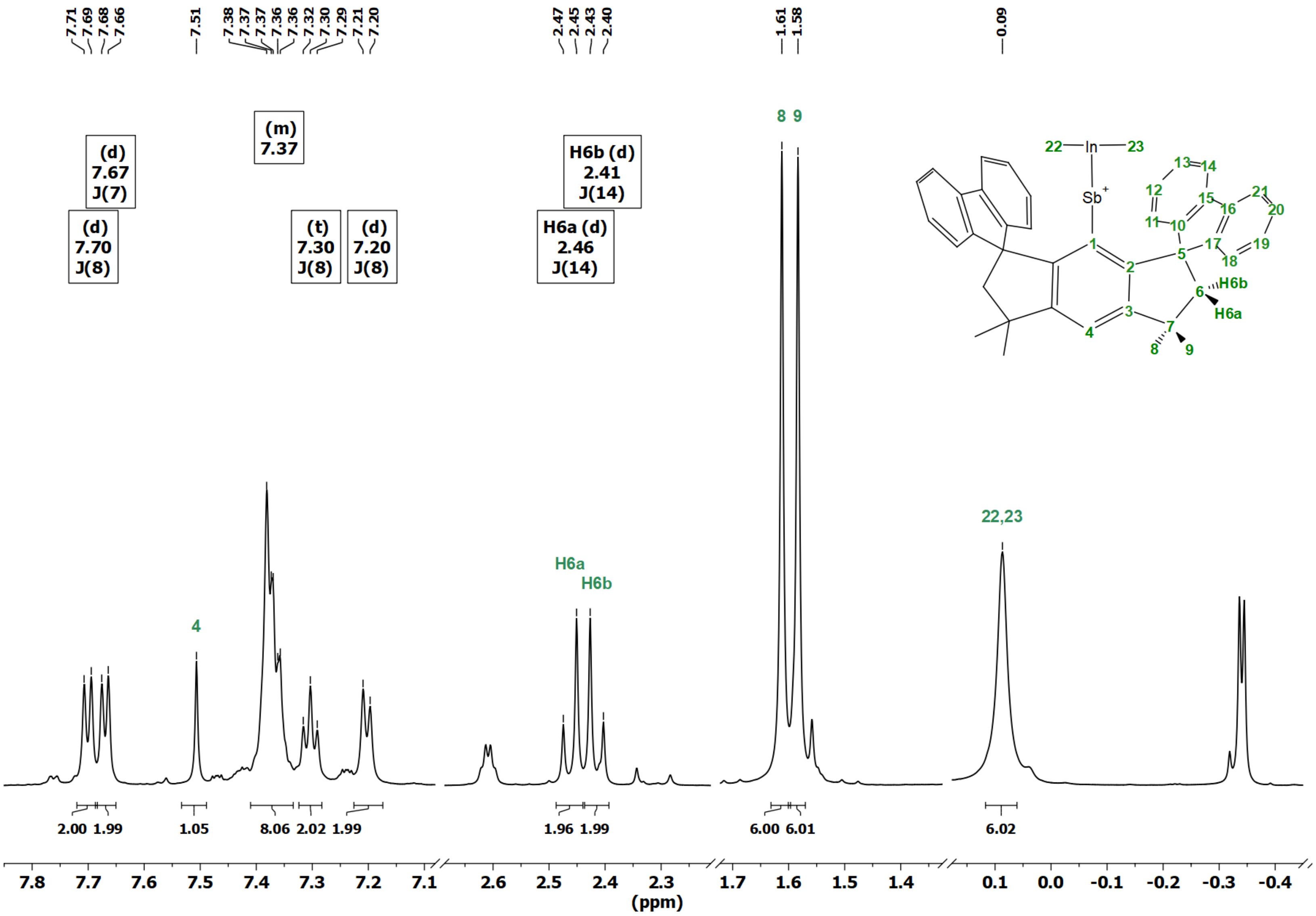

Figure S39. Detailed ^{1}H NMR (CD_2Cl_2, 600 MHz) spectrum of **5In**.

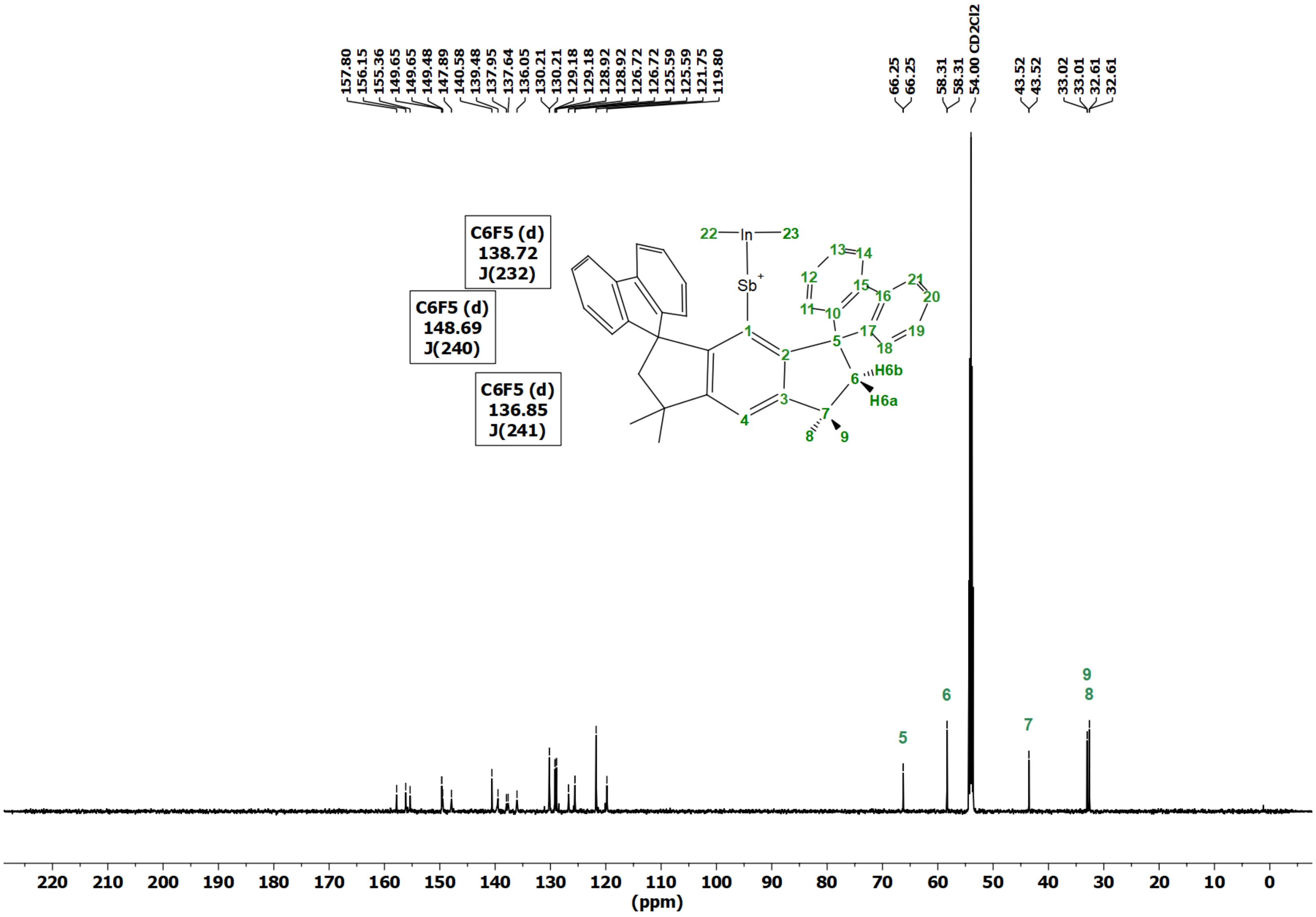

Figure S40. $^{13}C\{^1H\}$ NMR (CD_2Cl_2, 151 MHz) spectrum of **5In**.

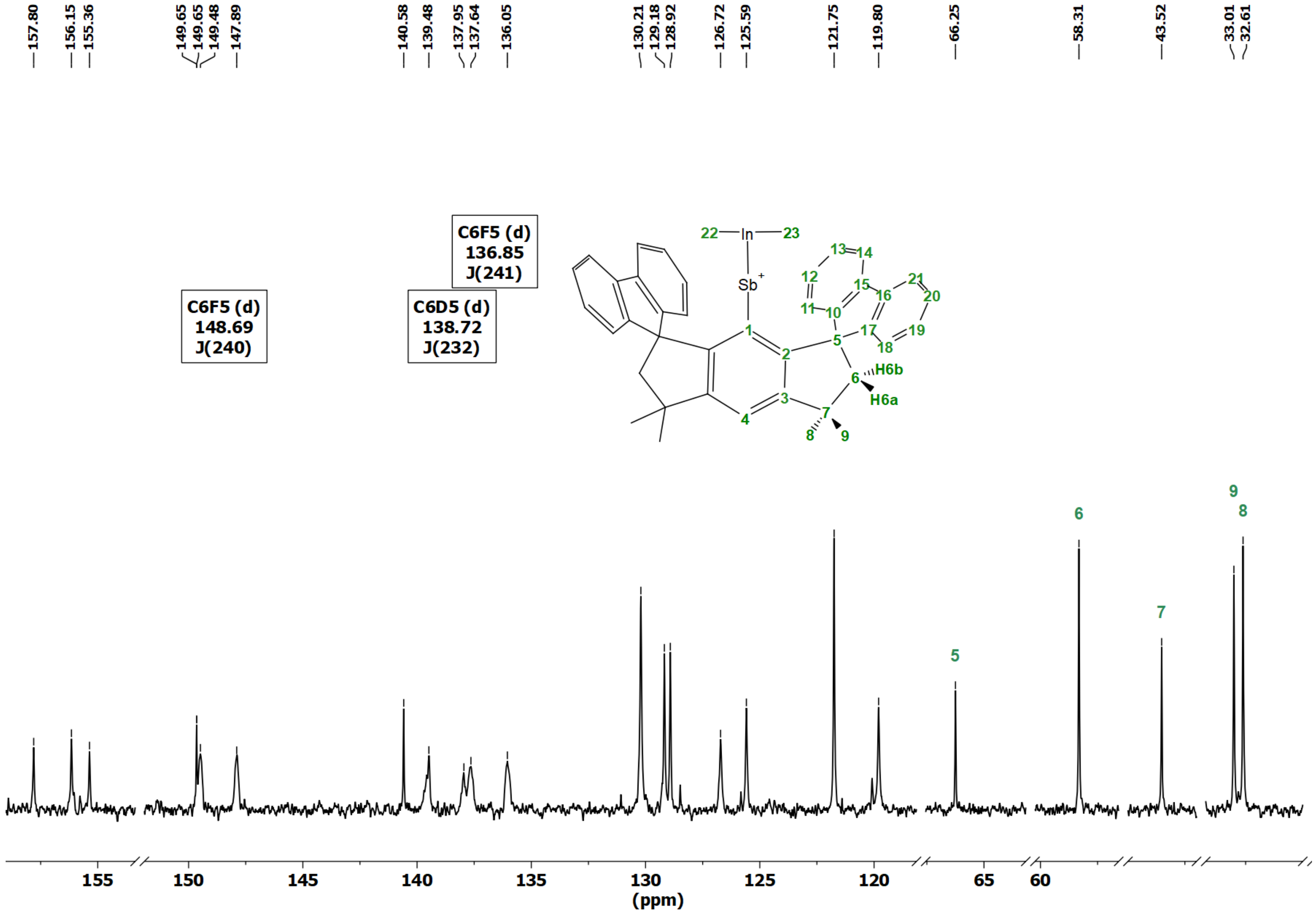

Figure S41. $^{13}C\{^{1}H\}$ NMR (CD_2Cl_2, 151 MHz) spectrum of **5In**.

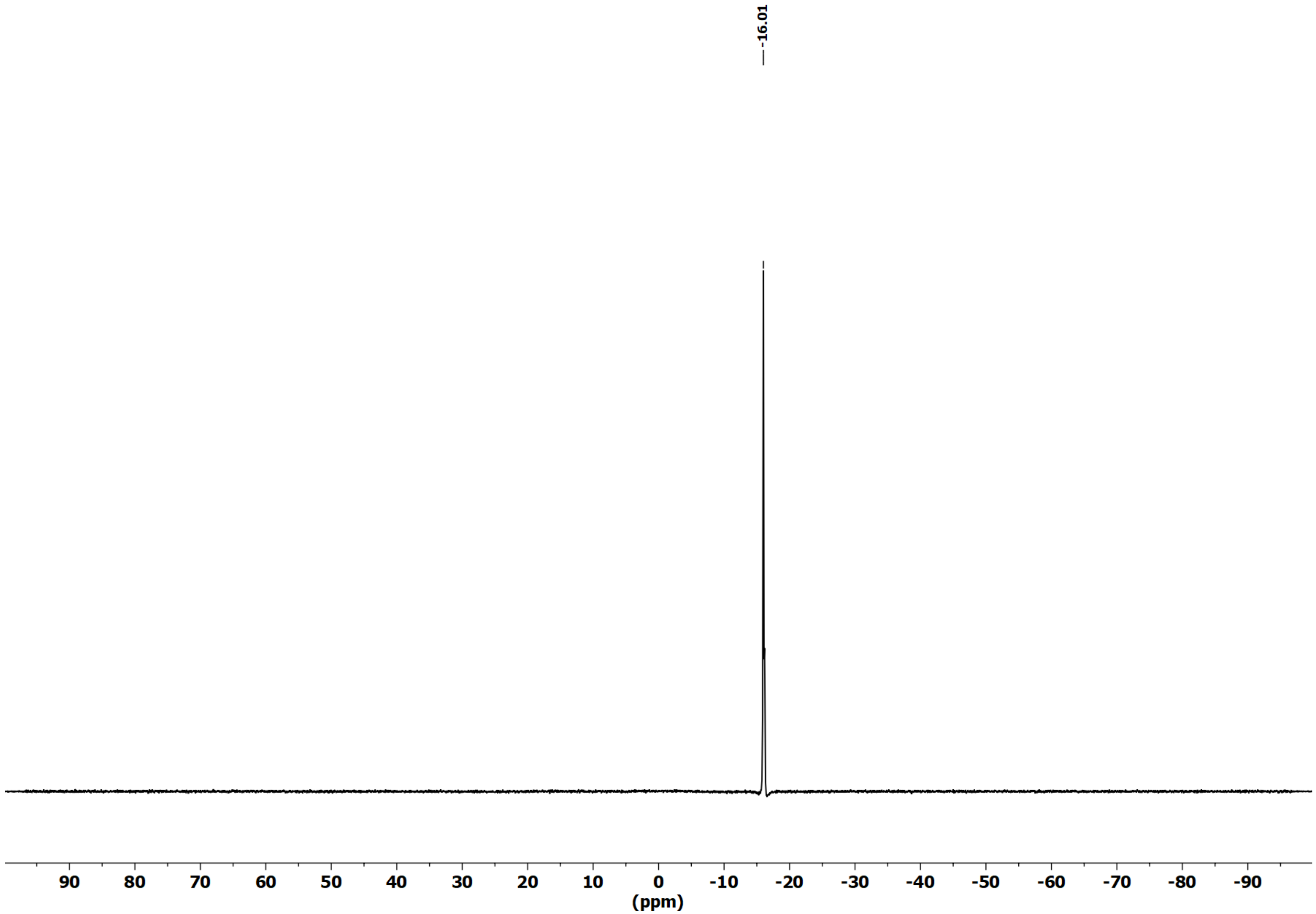

Figure S42. ^{11}B NMR (CD_2Cl_2, 193 MHz) spectrum of **5In**.

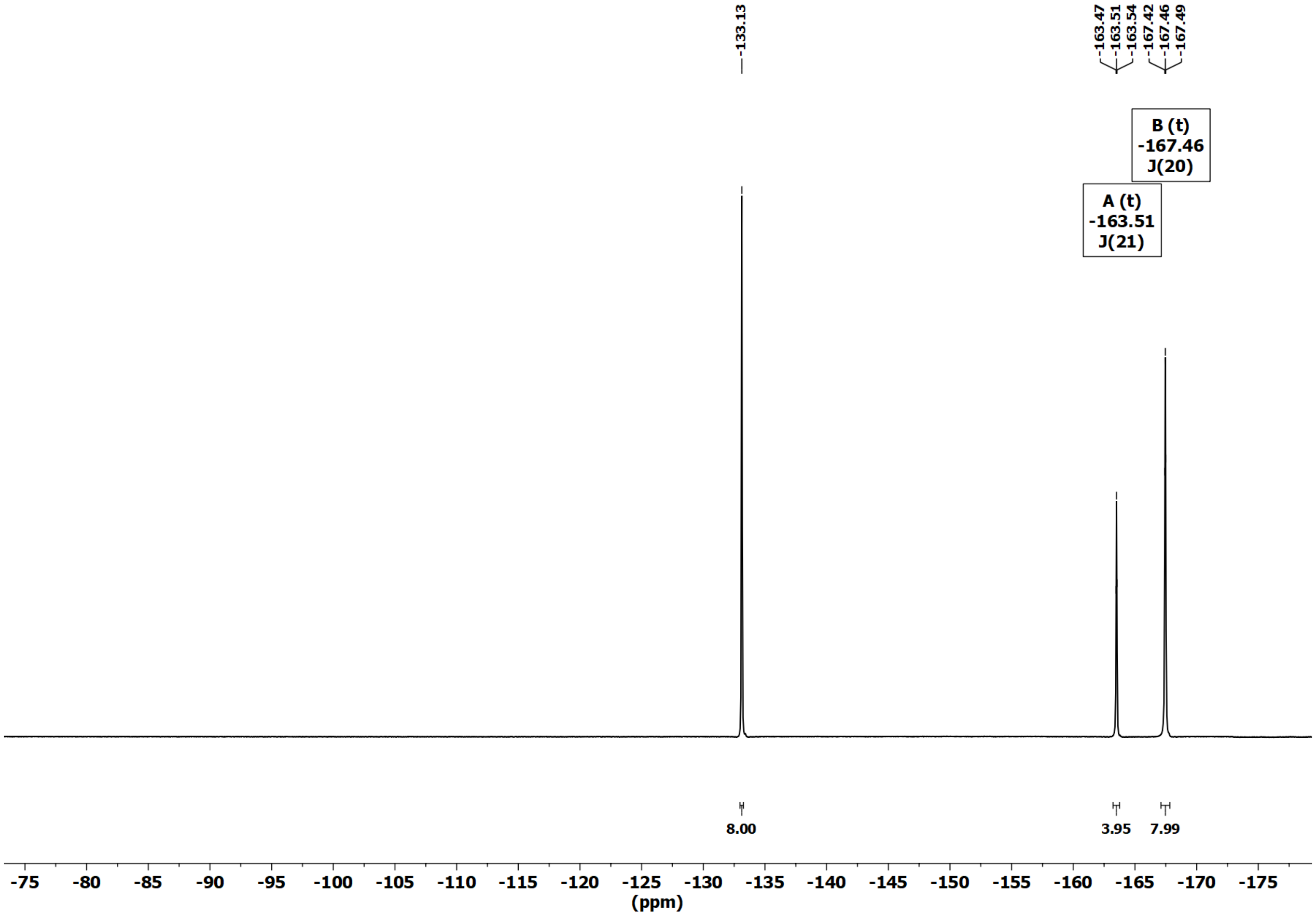

Figure S43. ^{19}F NMR (CD_2Cl_2, 565 MHz) spectrum of **5In**.

Reactivity of [M^SFluindSbH][$B(C_6F_5)_4$] (4) towards Me_3Ga

In a J. Young NMR Tube Me_3Ga (1.50 µL, 14.8 µmol) was added to a solution of [M^SFluindSbH][$B(C_6F_5)_4$] (15.0 mg, 11.4 µmol) in CD_2Cl_2 (0.5 mL) at room temperature and the resulting colorless solution was monitored by ^1H NMR spectroscopy. A quantitative conversion was observed to give one product, which was assigned to **5Ga** on the basis of NMR spectroscopy. All attempts to isolate 5Ga failed due to rapid decomposition.

^{1}H NMR (601 MHz, CD_2Cl_2): δ = 7.71 (d, $^3J(^1H-^1H)$ = 8 Hz, 2H, Ar-H), 7.67 – 7.62 (m, 2H), 7.54 (s, 1H, Ar-H), 7.44 – 7.35 (m, 8H, Ar-H), 7.32 (t, $^3J(^1H-^1H)$ = 7 Hz, 2H, Ar-H), 7.20 (d, $^3J(^1H-^1H)$ = 8 Hz, 2H, Ar-H), 2.49 (d, $^3J(^1H-^1H)$ = 14 Hz, 2H, CH_2), 2.44 (d, $^3J(^1H-^1H)$ = 14 Hz, 2H, CH_2), 1.63 (s, 6H, CH_3), 1.59 (s, 6H, CH_3), -0.02 (s, br, 6H, CH_3). **$^{13}C\{^1H\}$ NMR (151 MHz, CD_2Cl_2):** ^{13}C NMR (151 MHz, CD_2Cl_2) δ 158.0 (s, C_{Ar}), 156.2 (s, C_{Ar}), 154.9 (s, C_{Ar}), 149.5 (s, C_{Ar}), 148.7 (d, br, $^1J(^{13}C–^{19}F)$ = 244 Hz, C_6F_5), 139.5 (s, C_{Ar}), 139.3 (s, C_{Ar}), 138.8 (d, br, $^1J(^{13}C–^{19}F)$ = 243 Hz, C_6F_5), 136.8 (d, br, $^1J(^{13}C–^{19}F)$ = 246 Hz, C_6F_5), 130.9 (s, C_{Ar}), 130.5 (s), 130.1 (s, C_{Ar}), 129.2 (s, C_{Ar}), 127.0 (s, C_{Ar}), 125.7 (s, C_{Ar}), 122.4 (s, (C_{Ar}), 122.1 (s,C_{Ar}), 120.4 (s, C_{Ar}), 66.0 (s), 58.2 (s), 43.7 (s), 33.1 (s), 32.6 (s). **^{11}B NMR (193 MHz, CD_2Cl_2):** $\delta = -16.7$ (s). **^{19}F NMR (565 MHz, CD_2Cl_2):** $\delta = -133.1$ (br, 8F, *o*-C_6F_5), −163.7 (t, $^3J(^{19}F-^{19}F)$ = 20 Hz, 4F, *p*-C_6F_5), −167.5 (t, br, $^3J(^{19}F-^{19}F)$ = 19 Hz, 8F, *m*-C_6F_5).

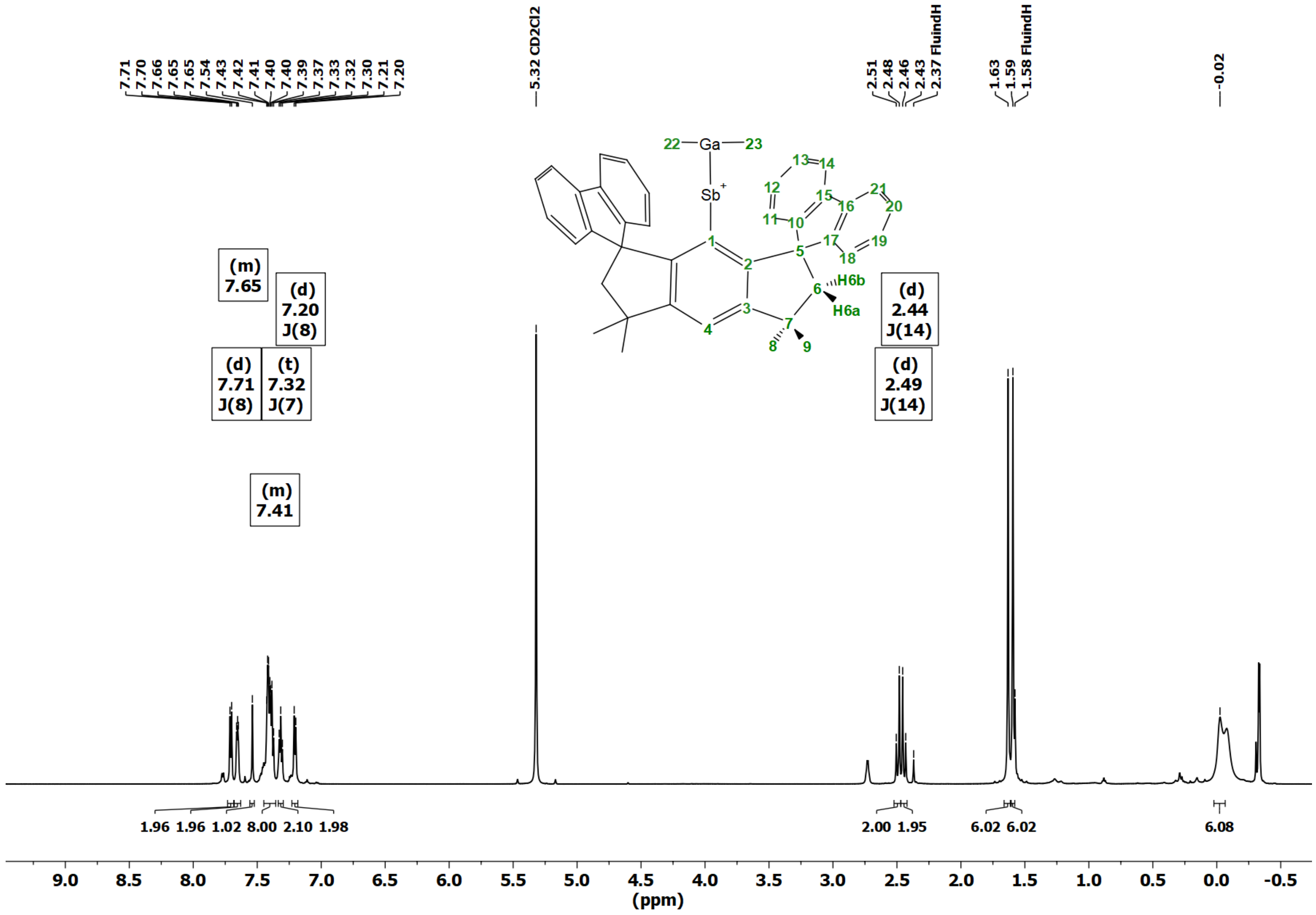

Figure S44. ^{1}H NMR (CD_2Cl_2, 600 MHz) spectrum of **5Ga**.

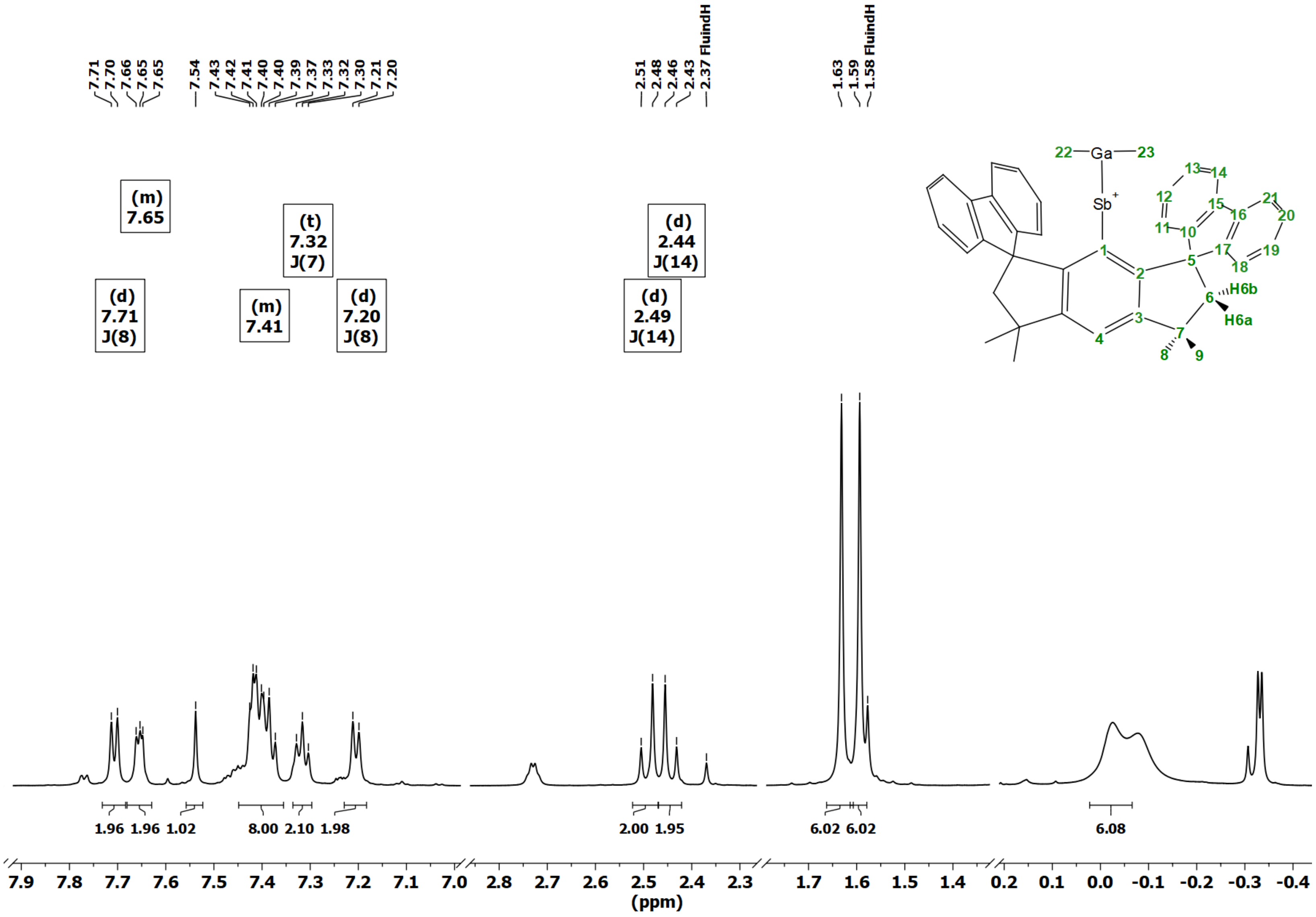

Figure S45. Detailed ^{1}H NMR (CD_2Cl_2, 600 MHz) spectrum of **5Ga**.

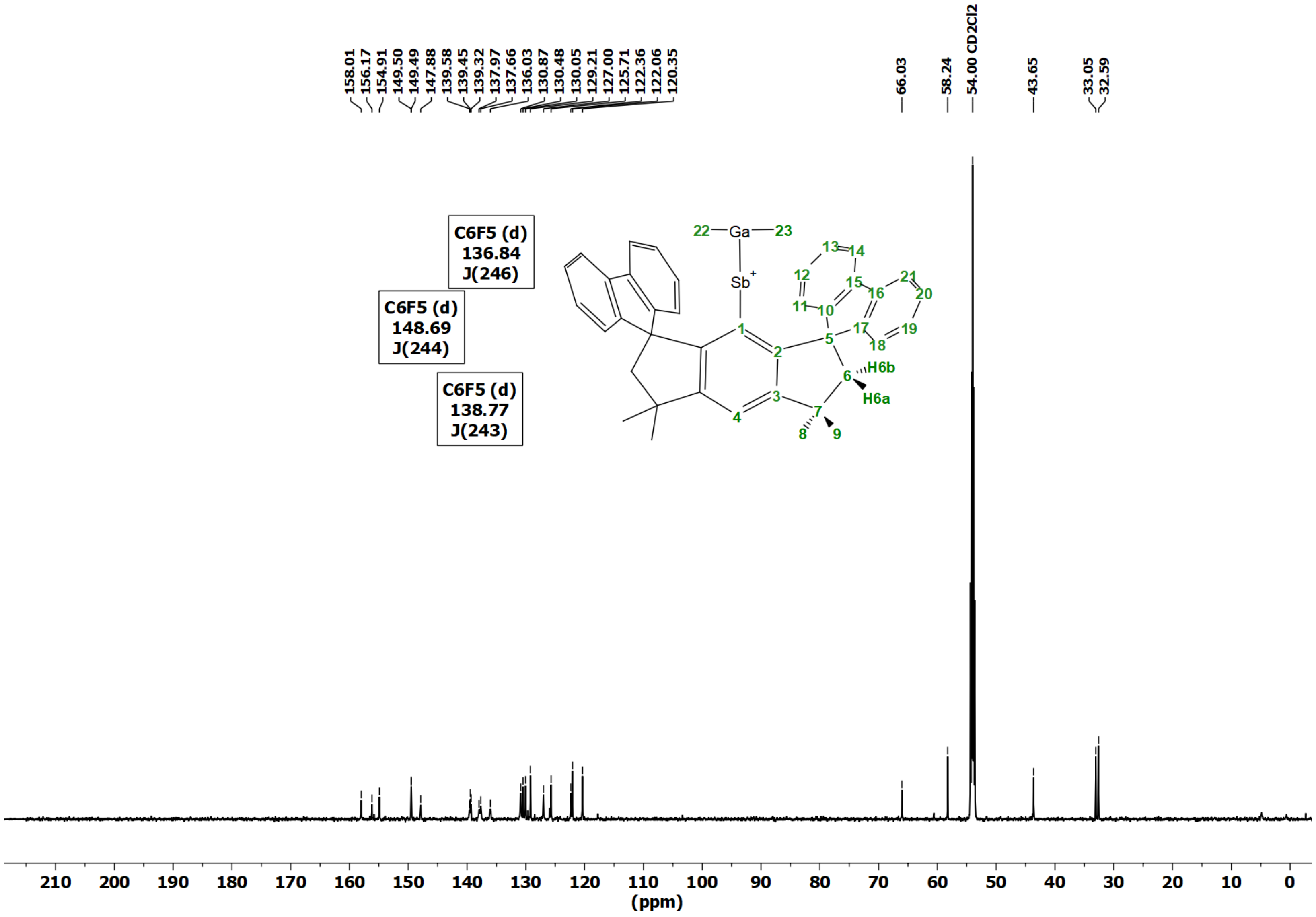

Figure S46. $^{13}C\{^{1}H\}$ NMR (CD_2Cl_2, 151 MHz) spectrum of **5Ga**.

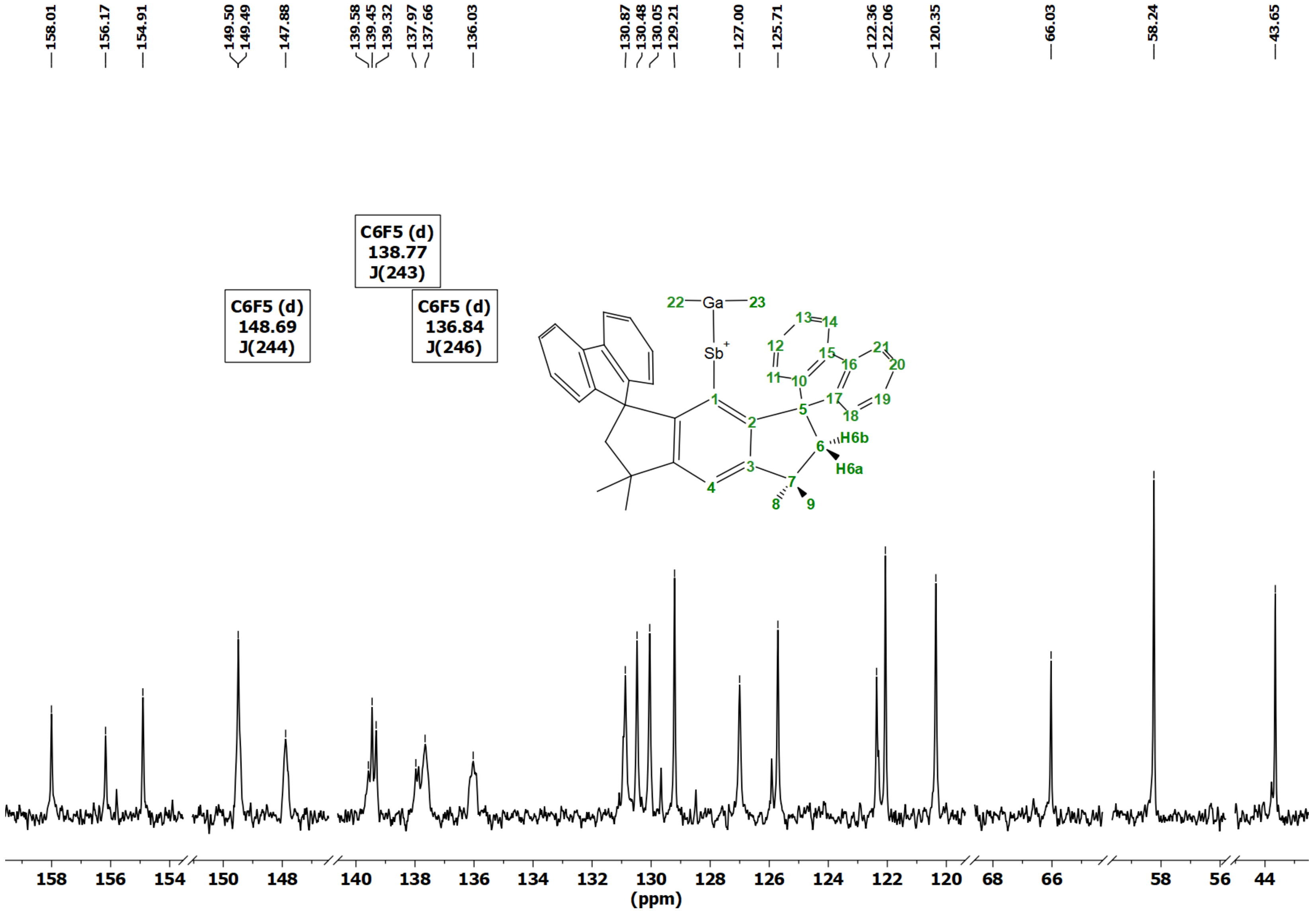

Figure S47. Detailed $^{13}C\{^1H\}$ NMR (CD_2Cl_2, 151 MHz) spectrum of **5Ga**.

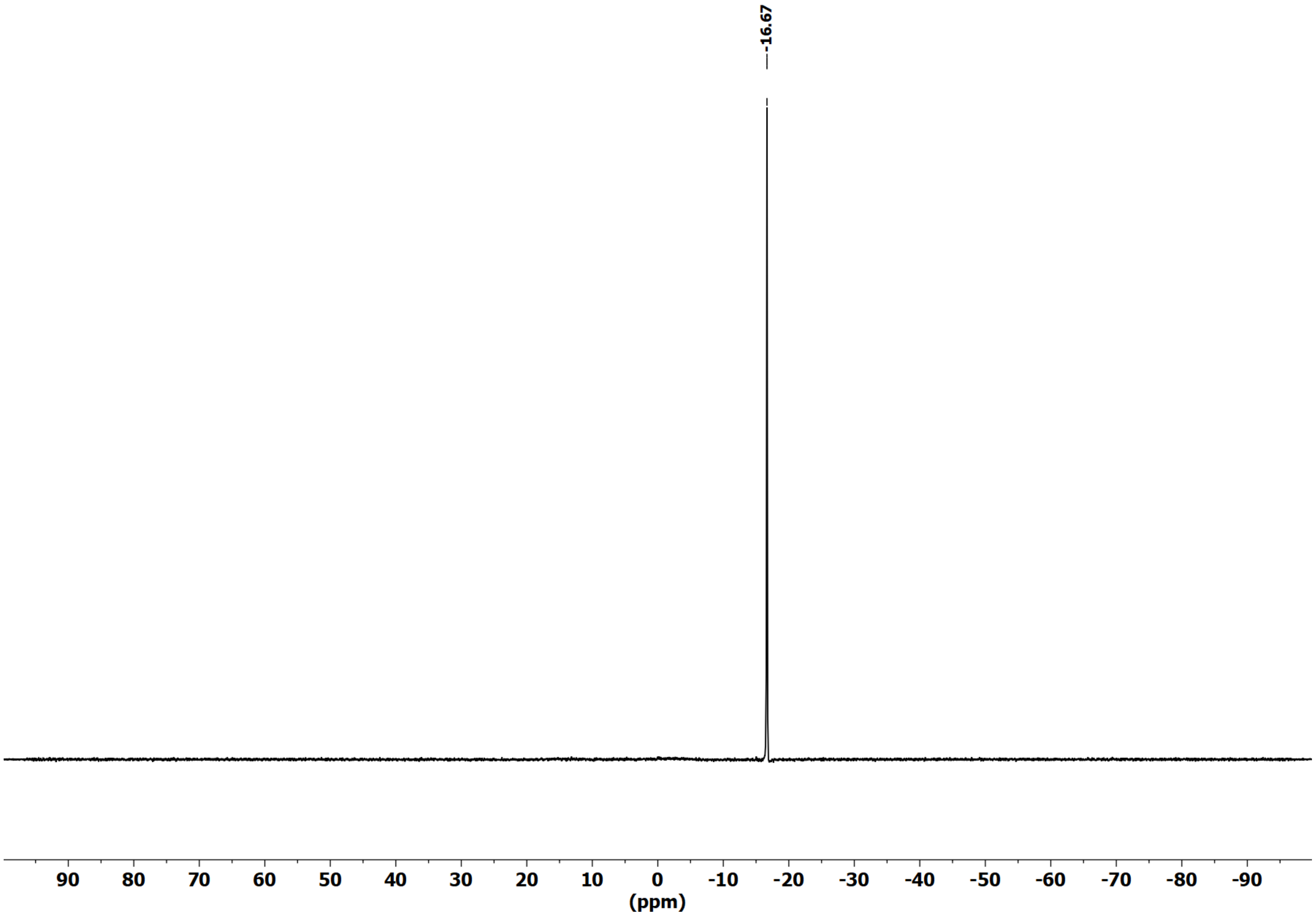

Figure S48. ^{11}B NMR (CD_2Cl_2, 193 MHz) spectrum of **5Ga**.

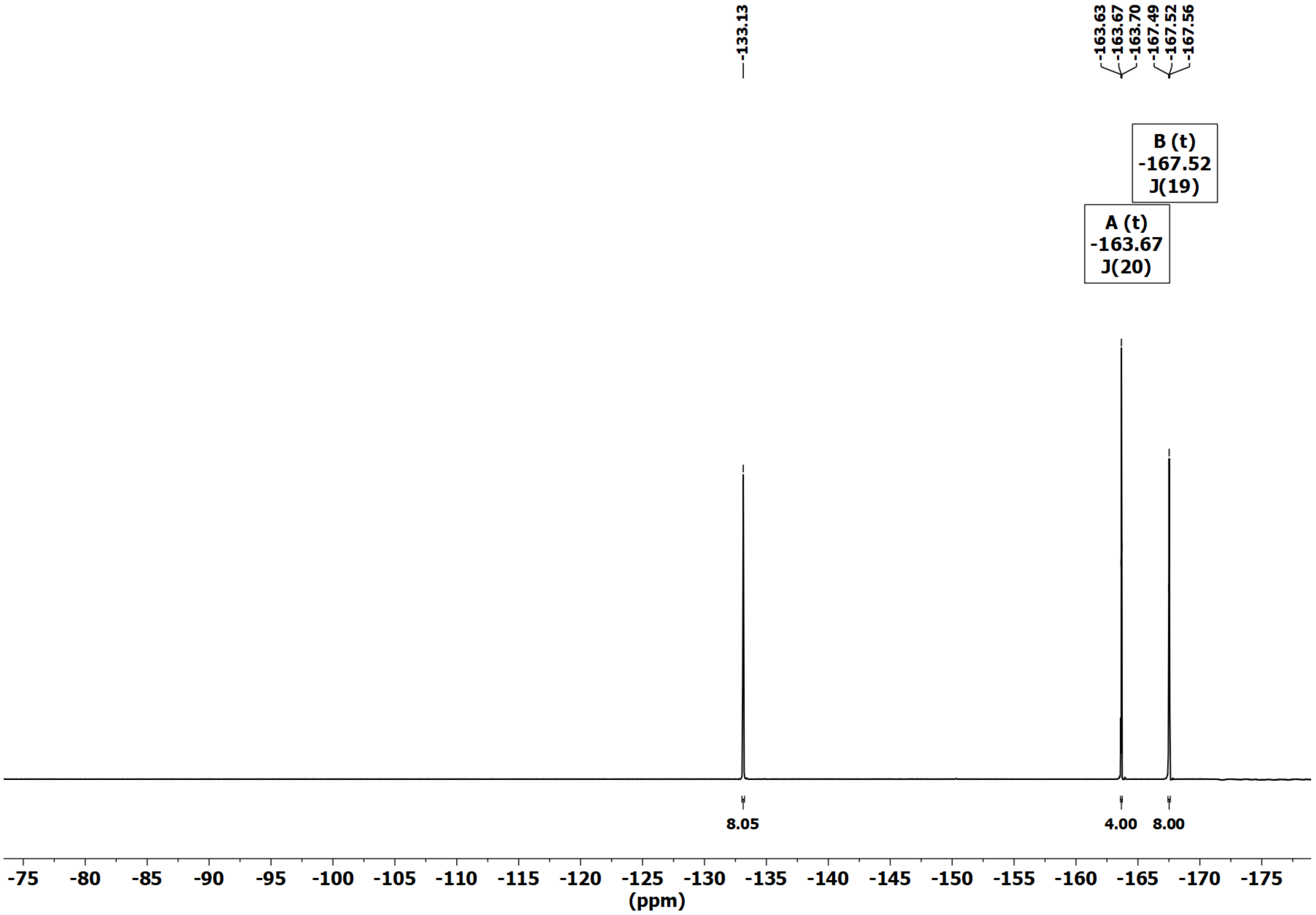

Figure S49. ^{19}F NMR (CD_2Cl_2, 565 MHz) spectrum of **5Ga**.

Synthesis and characterization of [M^{S}FluindSbSPh][B(C_6F_5)$_4$] (6S)

To a solid mixture of [M^{S}FluindSbH][B(C_6F_5)$_4$] (**4**, 50.0 mg, 38.0 µmol) and diphenyl disulfide (8.30 mg, 38.0 µmol) was added dichloromethane (5 mL). The resulting mixture was stirred at room temperature for 1 hour and afterwards *n*-hexane (5 mL) was layered over the solution. The crystalline product was decanted and washed with *n*-hexane (3 × 1 mL) and dried at reduced pressure. The title compound **6S** was obtained as an orange crystalline solid (37.2 mg, 26.1 µmol, 69%).

^{1}H NMR (601 MHz, CD_2Cl_2): δ 7.59 (d, $^3J(^1H\text{-}^1H)$ = 5 Hz, 2H, Ar-H), 7.55 (s, 1H, H4, Ar-H), 7.52 – 7.45 (m, 8H, Ar-H), 7.45 – 7.36 (m, 5H, Ar-H), 7.37 – 7.31 (m, 2H, Ar-H), 7.34 – 7.28 (m, 2H, Ar-H), 6.87 – 6.81 (m, 2H, H23 and H27), 2.64 (d, $^3J(^1H\text{-}^1H)$ = 14 Hz, 2H, H6a), 2.57 (d, $^3J(^1H\text{-}^1H)$ = 14 Hz, 2H, H6b), 1.78 (s, 6H, H9), 1.64 (s, 6H, H8). **^{13}C{^{1}H} NMR (151 MHz, CD_2Cl_2):** δ = 156.7 (s, C3), 155.5 (s, C2), 153.0 (s, C10), 150.5 (s, C17), 149.4 (s, C1), 148.7 (d, br, $^1J(^{13}C–^{19}F)$ = 238 Hz, *C*$_6$F$_5$), 143.3 (s, C16), 138.8 (d, br, $^1J(^{13}C–^{19}F)$ = 242 Hz, *C*$_6$F$_5$), 136.8 (d, br, $^1J(^{13}C–^{19}F)$ = 247 Hz, *C*$_6$F$_5$), 135.5 (s, C15), 133.7 (s, C_{Ar}), 132.6 (s, C_{Ar}), 132.3 (s, C_{Ar}), 131.4 (s, C_{Ar}), 130.5 (s, C_{Ar}), 130.4 (s, C_{Ar}), 130.2 (s, C_{Ar}), 129.2 (s, C_{Ar}), 129.1 (s, C_{Ar}), 126.3 (s, C_{Ar}), 123.4 (s, C_{Ar}), 122.6 (s, C_{Ar}), 122.0 (s, C4), 63.7 (s, C5), 53.9 (s, C6)*, 46,6 (s, C7) , 33.3 (s, C8), 32.3 (s, C9).*signal overlaps with signal of CD_2Cl_2. **^{11}B NMR (193 MHz, CD_2Cl_2):** $\delta = -16.7$ (s). **^{19}F NMR (565 MHz, CD_2Cl_2):** $\delta = -133.9$ (br, 8F, *o*-C_6F_5), −162.9 (t, $^3J(^{19}F\text{-}^{19}F)$ = 20 Hz, 4F, *p*-C_6F_5), −166.9 (t, br, $^3J(^{19}F\text{-}^{19}F)$ = 19 Hz, 8F, *m*-C_6F_5). **HRMS ESI (m/z):** $[M]^+$ calculated for $C_{46}H_{38}SSb$, 743.1727; found 743.1716. **M.p.:** >240 °C (decomp.).

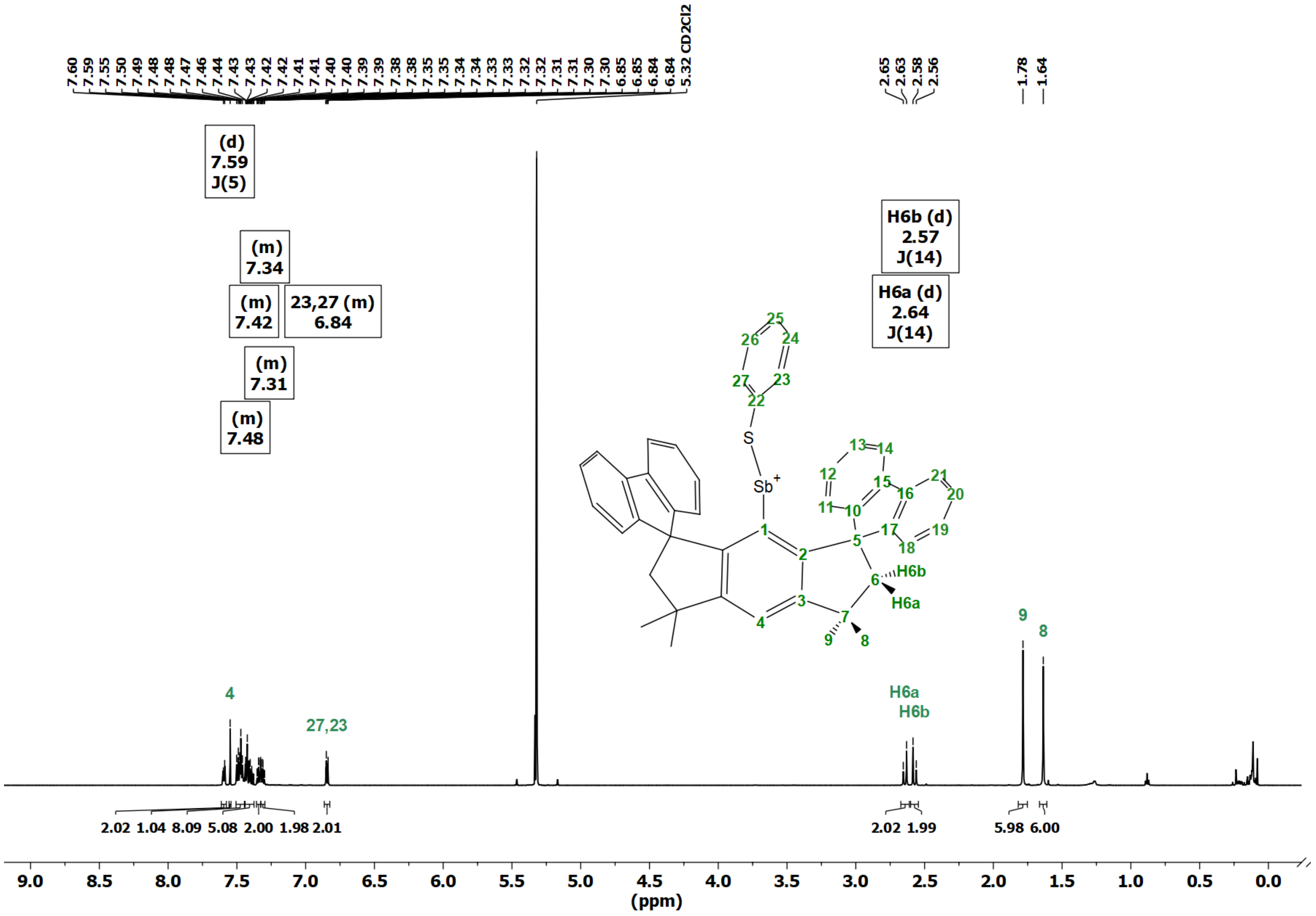

Figure S50. ^{1}H NMR (CD_2Cl_2, 600 MHz) spectrum of **6S**.

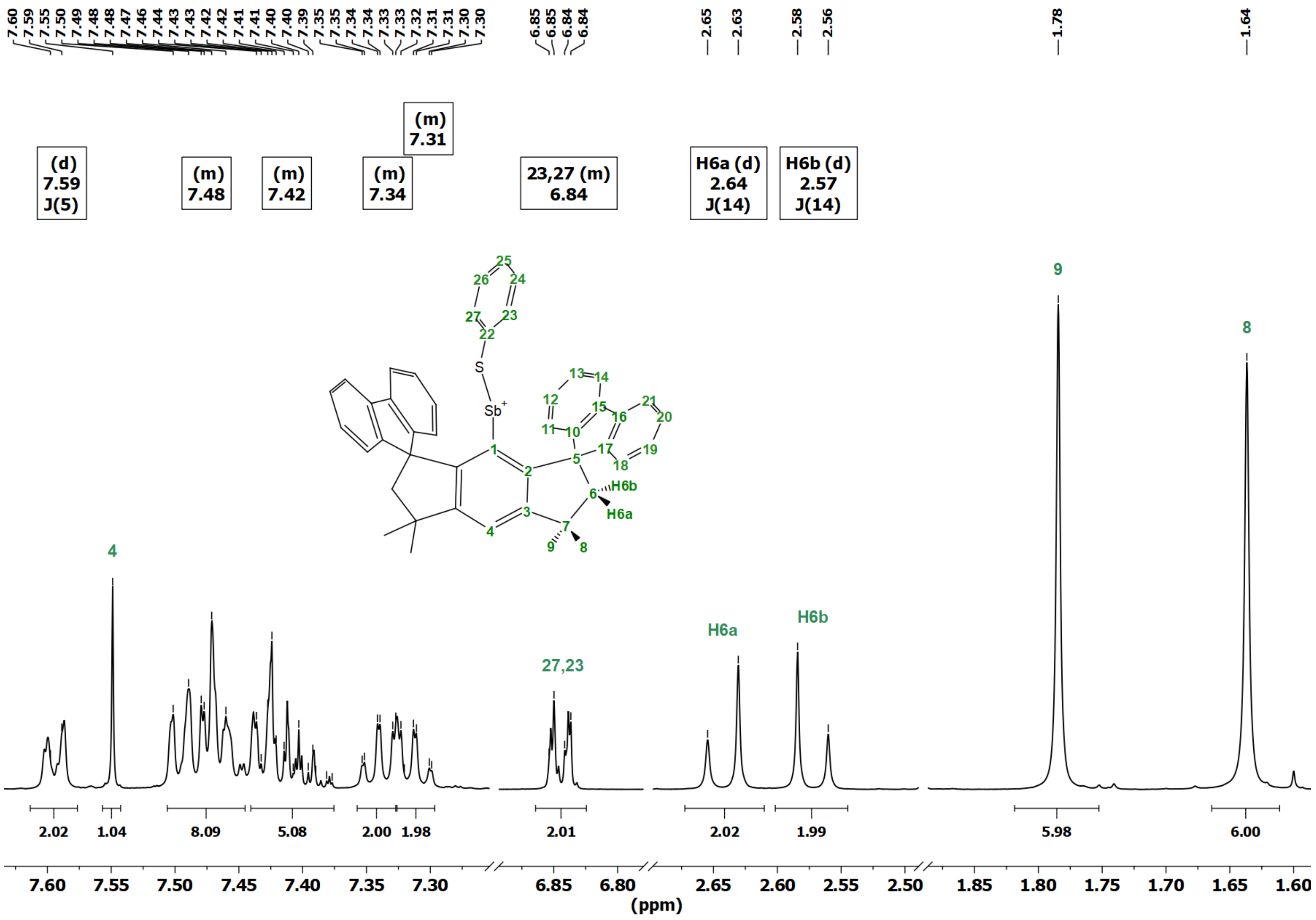

Figure S51. ^{1}H NMR (CD_2Cl_2, 600 MHz) spectrum of **6S**.

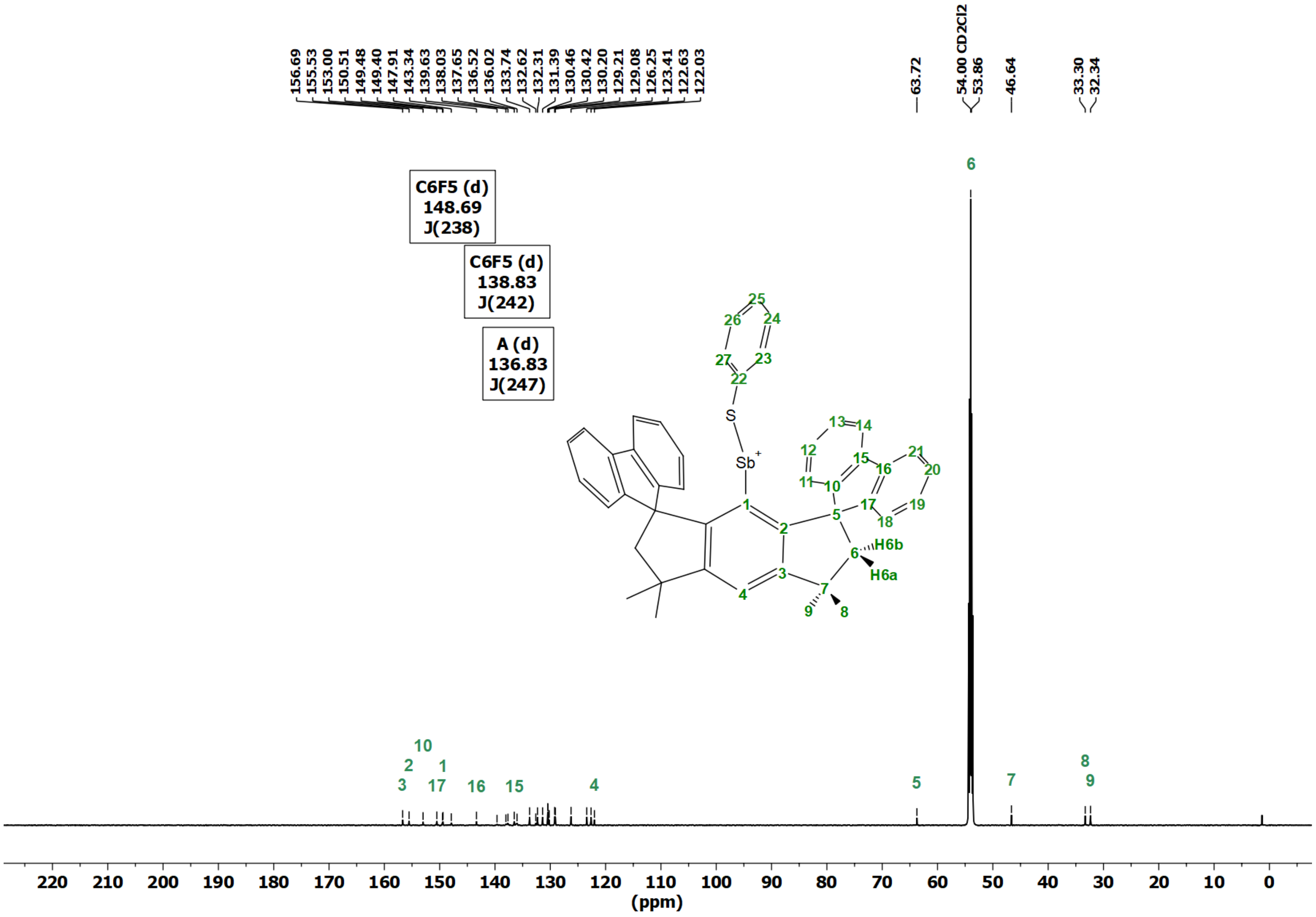

Figure S52. $^{13}C\{^{1}H\}$ NMR (CD_2Cl_2, 151 MHz) spectrum of **6S**.

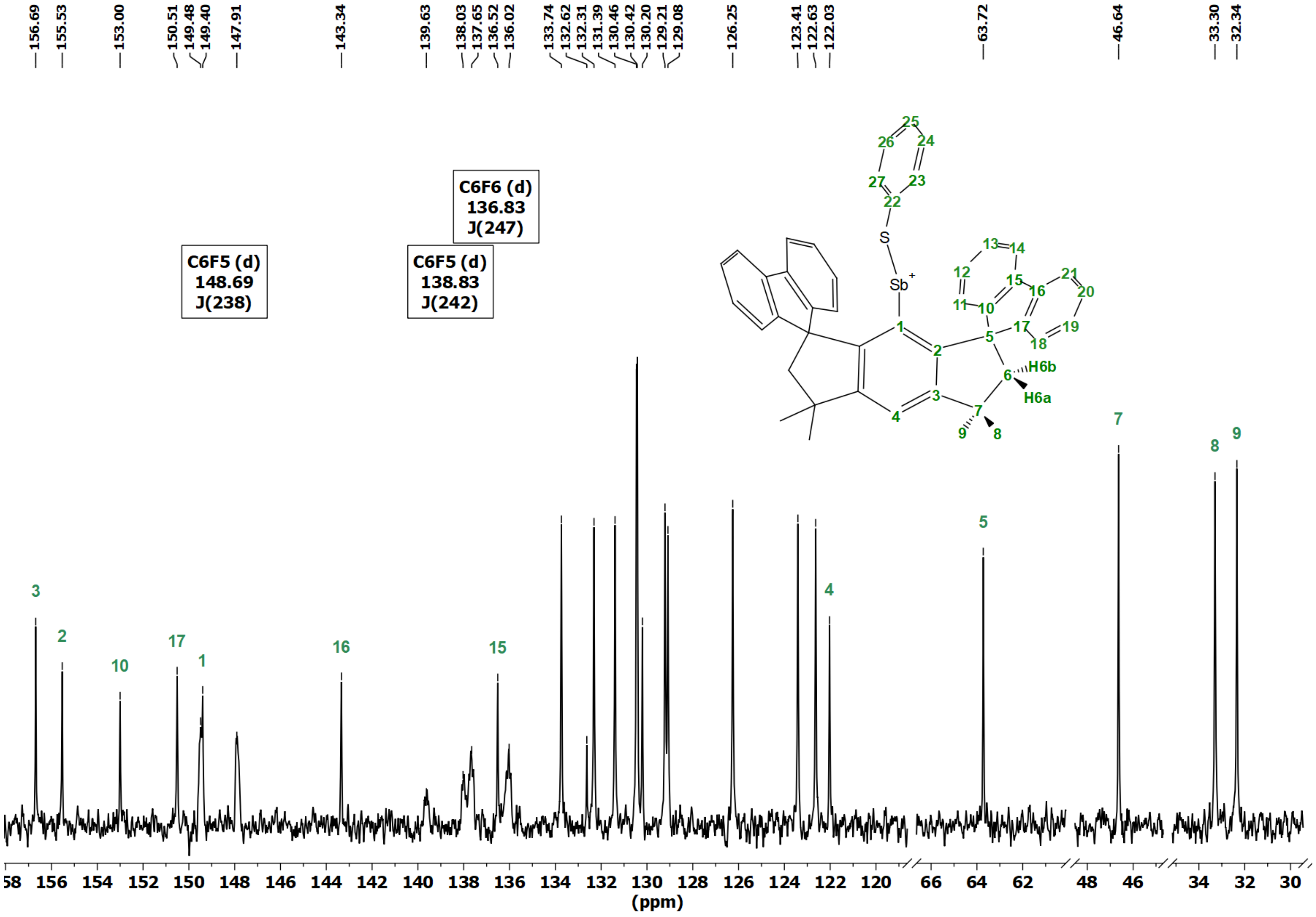

Figure S53. Detailed $^{13}C\{^{1}H\}$ NMR (CD_2Cl_2, 151 MHz) spectrum of **6S**.

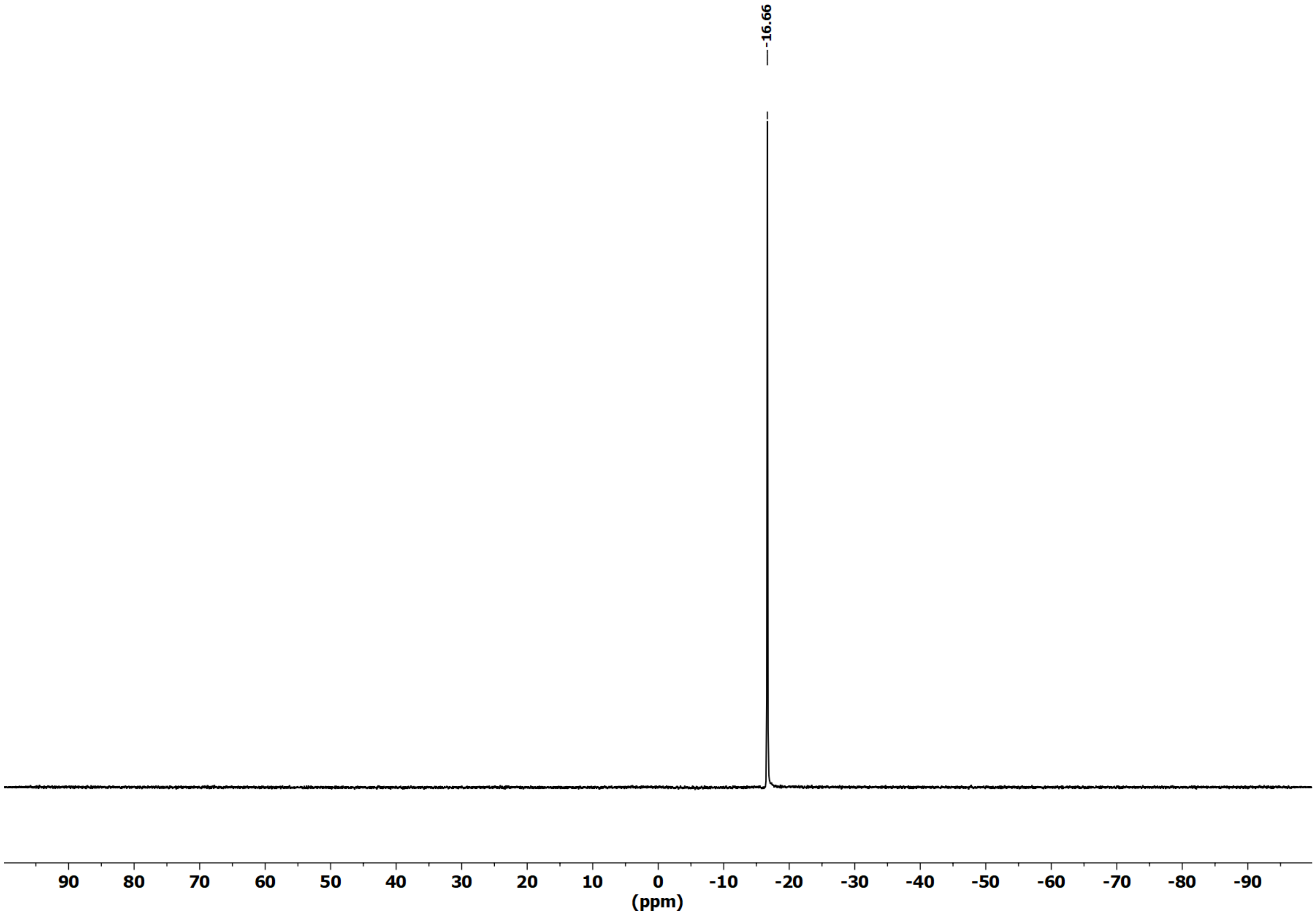

Figure S54. ^{11}B NMR (CD_2Cl_2, 193 MHz) spectrum of **6S**.

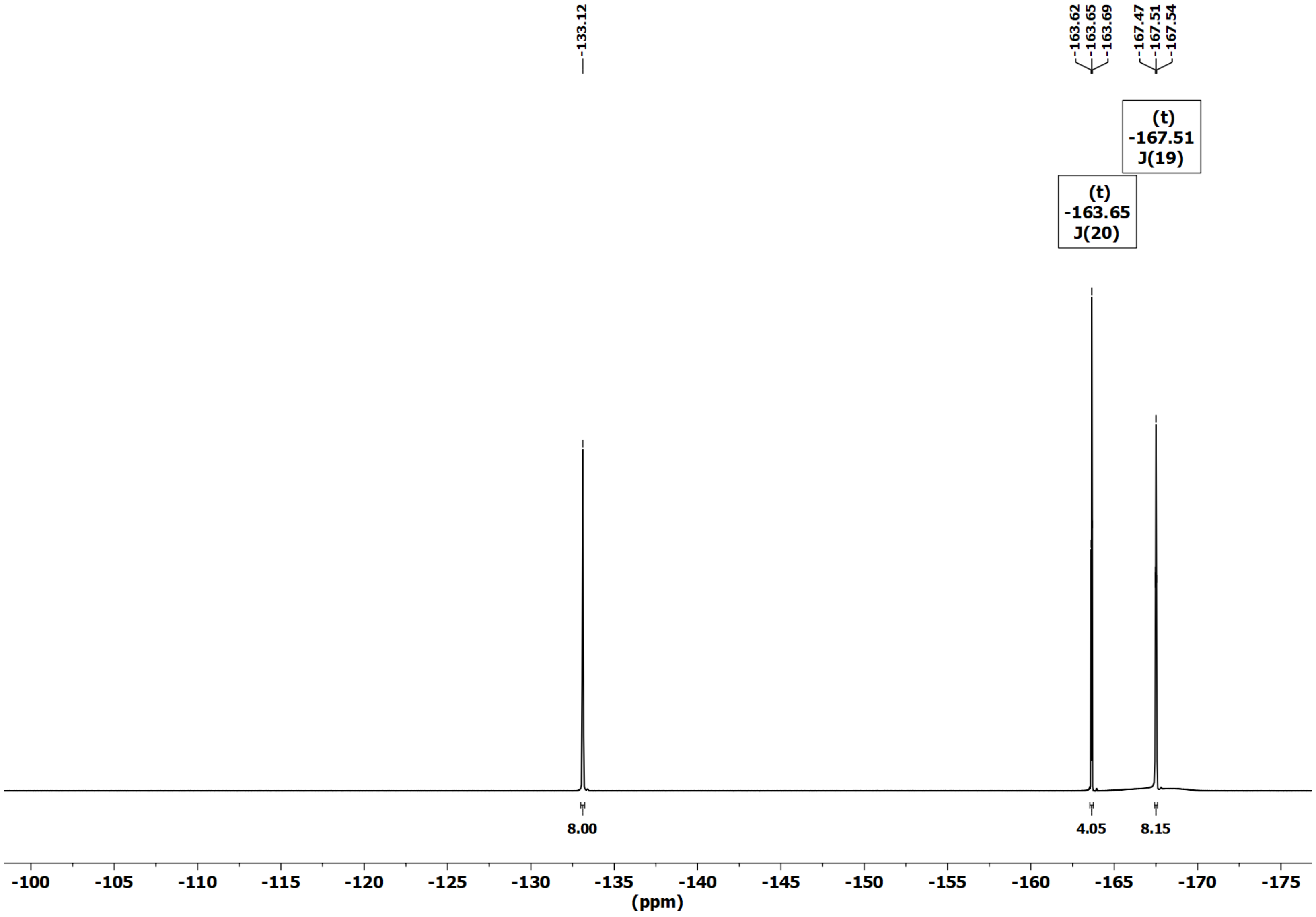

Figure S55. ^{19}F NMR (CD_2Cl_2, 565 MHz) spectrum of **6S**.

Synthesis and characterization of [M^{S}FluindSbSePh][B(C_6F_5)$_4$] (6Se)

To a solid mixture of [M^{S}FluindSbH][B(C_6F_5)$_4$] (50.0 mg, 38.0 µmol) and diphenyl diselenide (11.7 mg, 3.80 µmol) was added dichloromethane (5 mL). The resulting mixture was stirred at room temperature for 1 hour. Then *n*-hexane (5 mL) was layered over the solution. The crystalline product was decanted and washed with *n*-hexane (3 × 1 mL) and dried at reduced pressure. The title compound **6Se** was obtained as an orange crystalline solid (34.6 mg, 23.5 µmol, 62%).

^{1}H NMR (601 MHz, CD_2Cl_2): δ = 7.57 – 7.53 (m, 3H, H4 and H14), 7.56 – 7.52 (m, 2H, H24 and H26), 7.49 – 7.44 (m, 2H, H21), 7.47 – 7.39 (m, 9H, H11, H18, H19, H20 and H25), 7.36 (td, ^3J(^{1}H-^{1}H) = 7 Hz, ^4J(^{1}H-^{1}H) = 1 Hz, 2H, H13), 7.27 (td, ^3J(^{1}H-^{1}H) = 8 Hz, ^4J(^{1}H-^{1}H) = 1 Hz, 2H, H12), 7.00 – 6.97 (m, 2H, H23 and H27), 2.62 (d, ^3J(^{1}H-^{1}H) = 14 Hz, 2H, H6a), 2.55 (d, ^3J(^{1}H-^{1}H) = 14 Hz, 2H, H6b), 1.78 (s, 6H, H8), 1.63 (s, 6H, H9). **^{13}C{^{1}H} NMR (151 MHz, CD_2Cl_2):** δ = 156.8 (s, C3), 154.8 (s, C2), 152.8 (s, C10), 149.8 (s, C17), 148.7 (d, br, ^1J(^{13}C–^{19}F) = 241 Hz, *C*$_6$F$_5$), 148.1 (s, C1), 142.9 (s, C16), 138.8 (d, br, ^1J(^{13}C–^{19}F) = 248 Hz, *C*$_6$F$_5$), 138.9 (d, br, ^1J(^{13}C–^{19}F) = 246 Hz, *C*$_6$F$_5$), 137.1 (s, C15), 133.5 (s, C20), 132.4 (s, C23 and C27), 132.1 (s, C19), 131.2 (s, C12), 130.8 (s, C18), 130.5 (s, C25), 129.2 (s, C13), 128.9 (s, C21), 128.5 (s, C22), 126.4 (s, C11), 123.1 (s, C14), 122.5 (s, C24 and C26), 122.0 (s, C4), 63.8 (s, C5), 54.0 (s, C6)*, 46.5 (s, C7), 33.3 (s, C8), 32.3 (s, C9).*signal overlaps with signal of CD_2Cl_2. **^{11}B NMR (193 MHz, CD_2Cl_2):** *δ* = −16.6 (s). **^{19}F NMR (565 MHz, CD_2Cl_2):** *δ* = −133.1 (br, 8F, *o*-C_6F_5), −163.8 (t, ^3J(^{19}F-^{19}F) = 20 Hz, 4F, *p*-C_6F_5), −167.6 (t, br, ^3J(^{19}F-^{19}F) = 19 Hz, 8F, *m*-C_6F_5). **^{77}Se NMR (114 MHz, CD_2Cl_2):** δ = 1659.1 (s). **HRMS ESI (m/z):** $[M]^+$ calculated for $C_{46}H_{38}SbSe$, 791.1181; found 791.1190. **M.p.:** > 230 °C (decomp.).

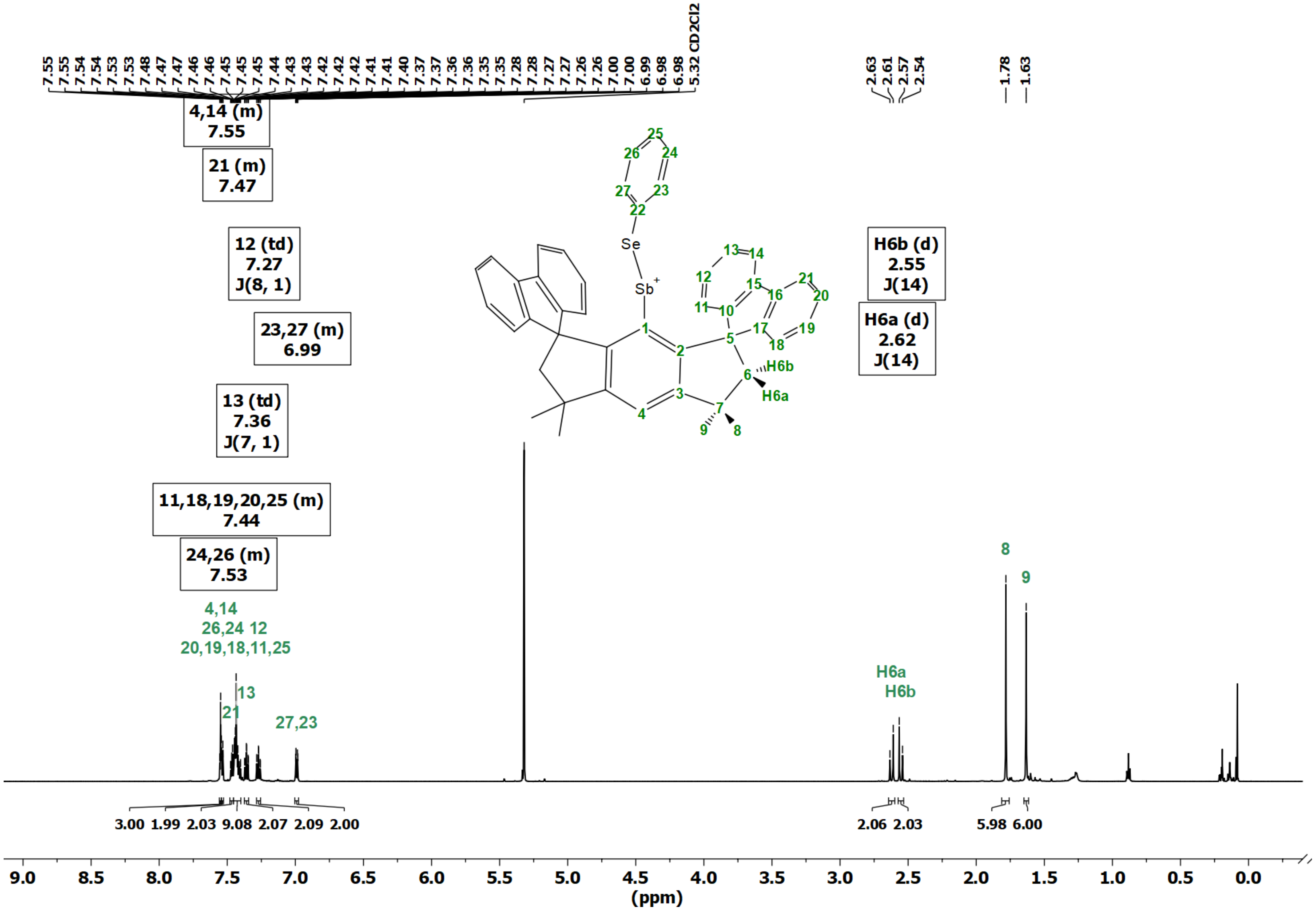

Figure S56. ^{1}H NMR (CD_2Cl_2, 600 MHz) spectrum of **6Se**.

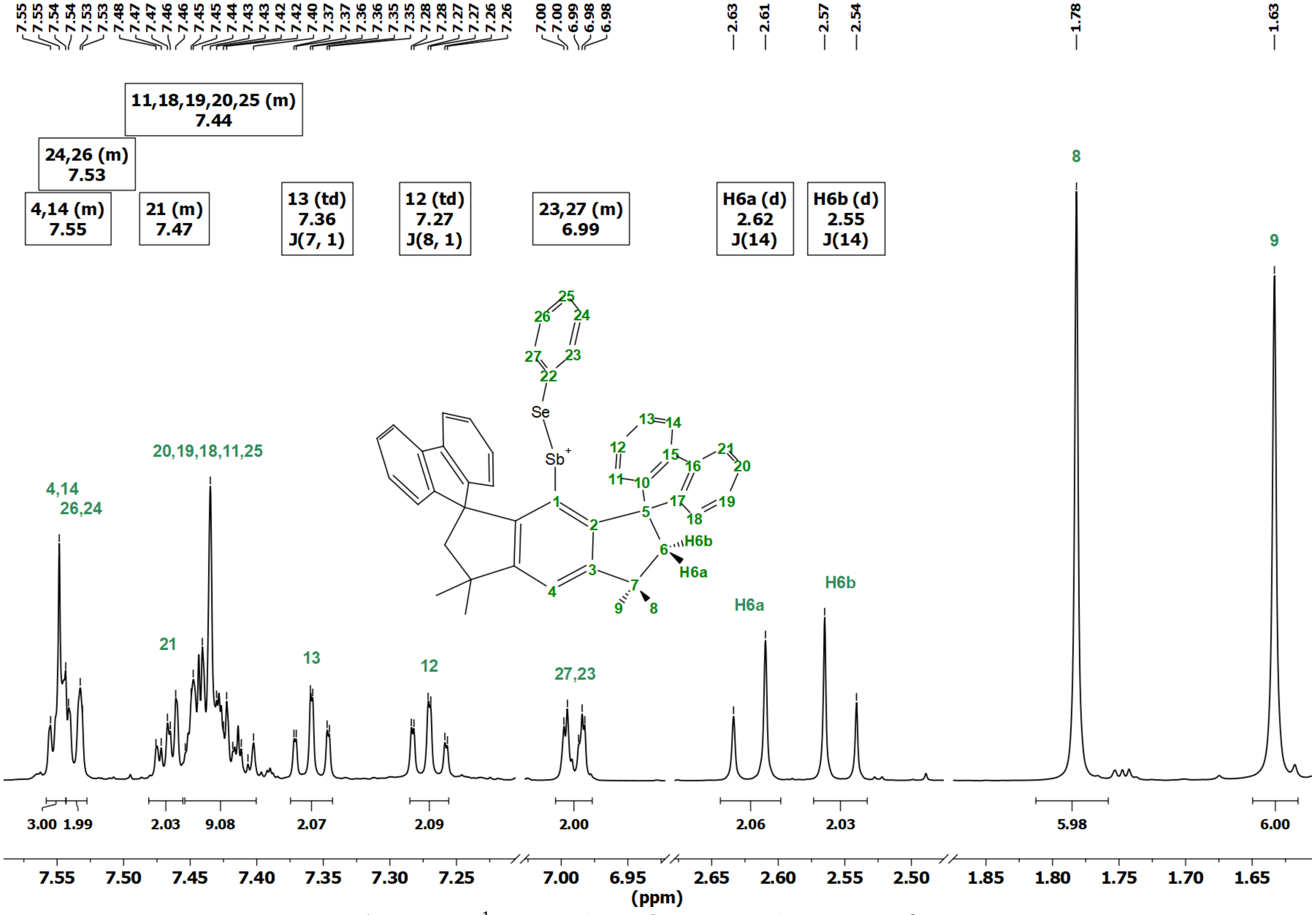

Figure S57. ^{1}H NMR (CD_2Cl_2, 600 MHz) spectrum of **6Se**.

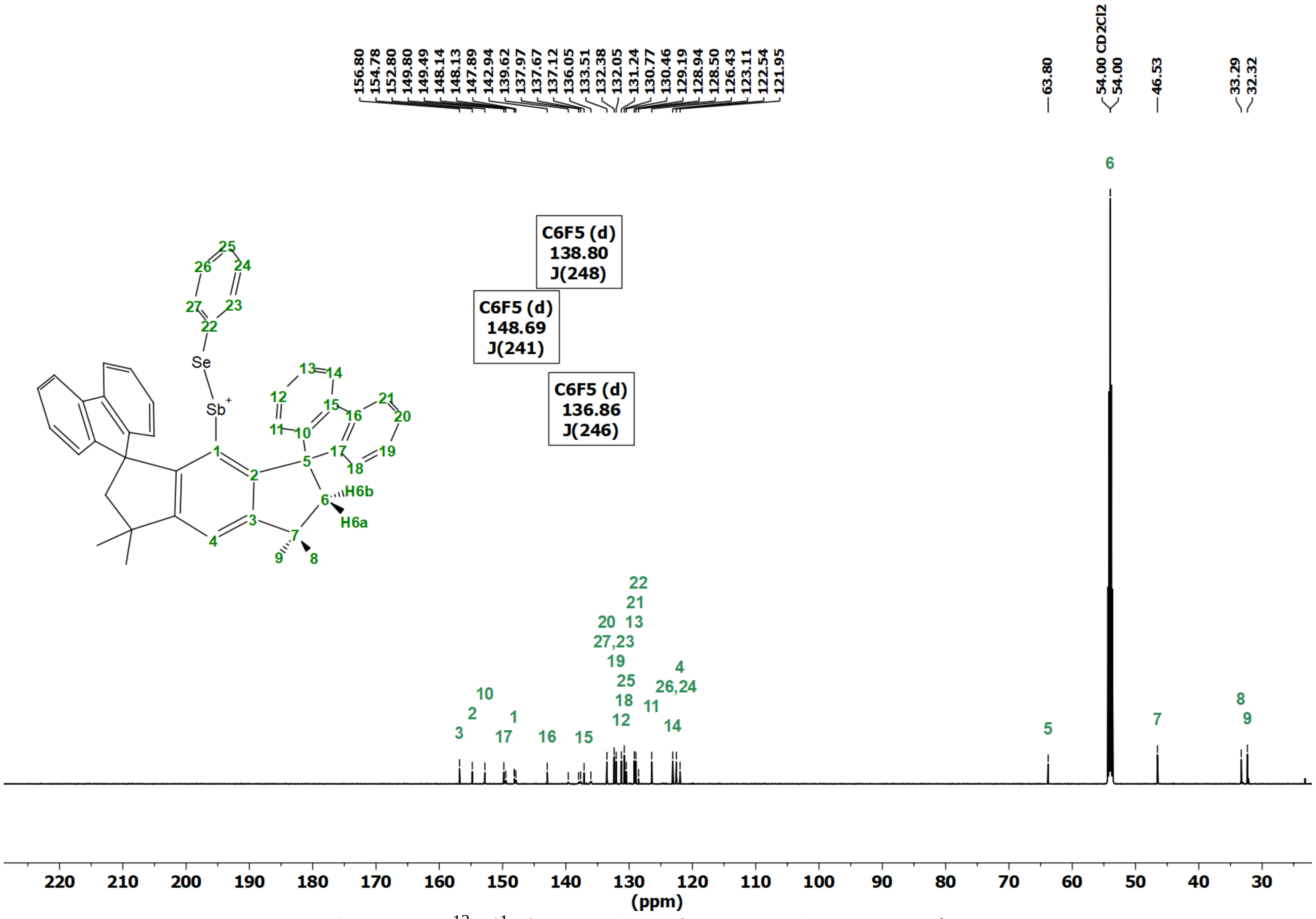

Figure S58. $^{13}C\{^{1}H\}$ NMR (CD_2Cl_2, 151 MHz) spectrum of **6Se**.

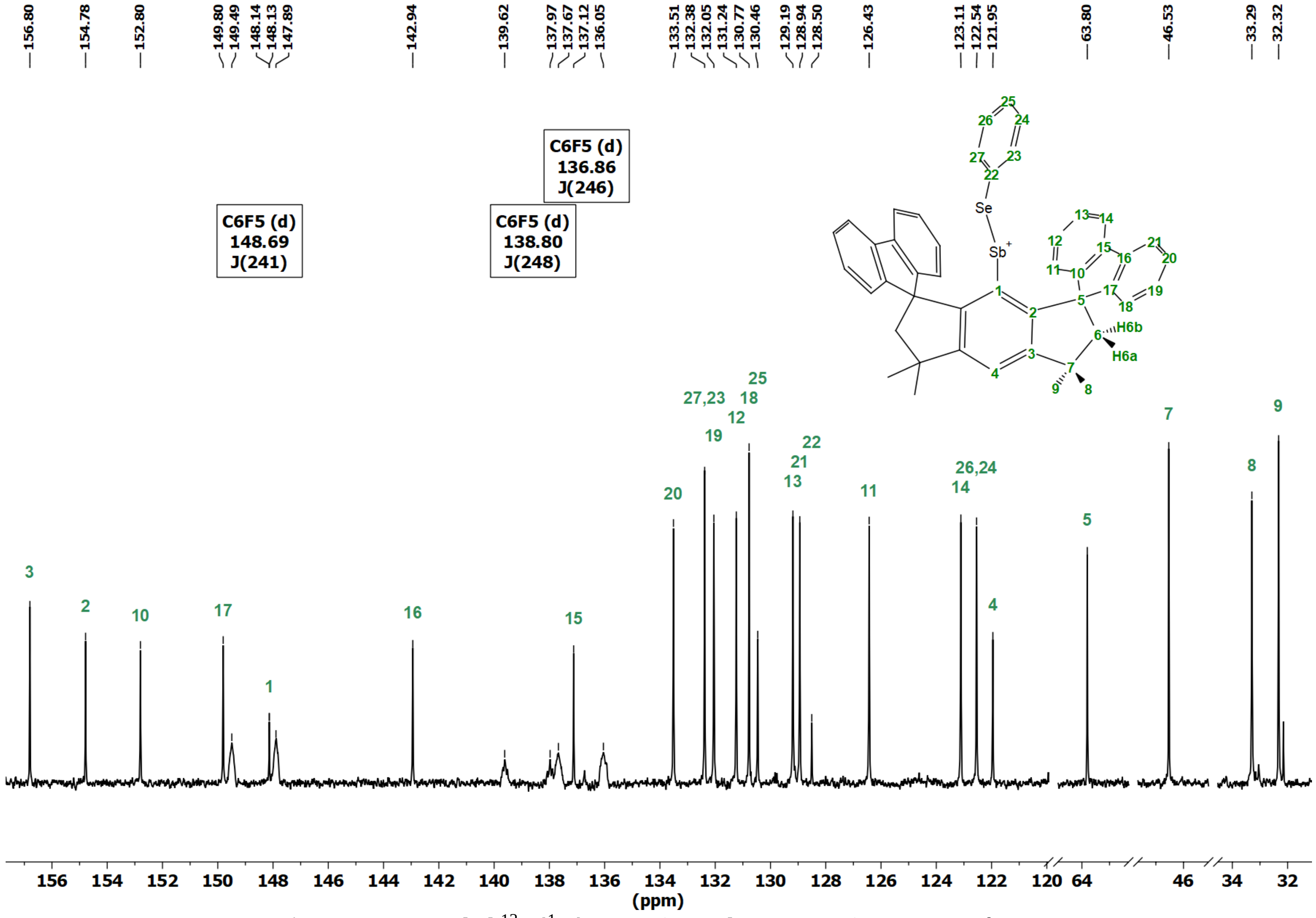

Figure S59. Detailed $^{13}C\{^1H\}$ NMR (CD_2Cl_2, 151 MHz) spectrum of **6Se**.

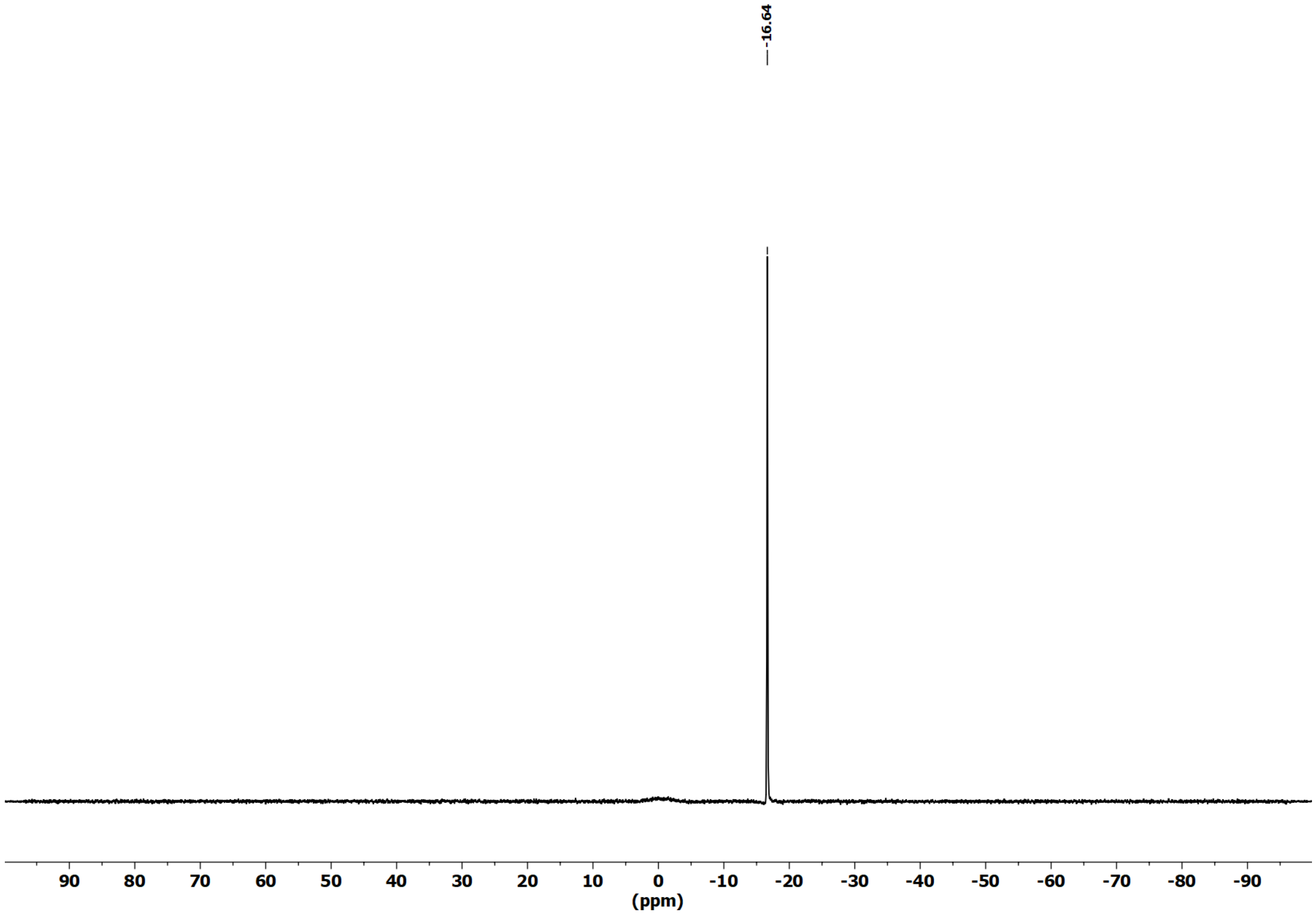

Figure S60. ^{11}B NMR (CD_2Cl_2, 193 MHz) spectrum of **6Se**.

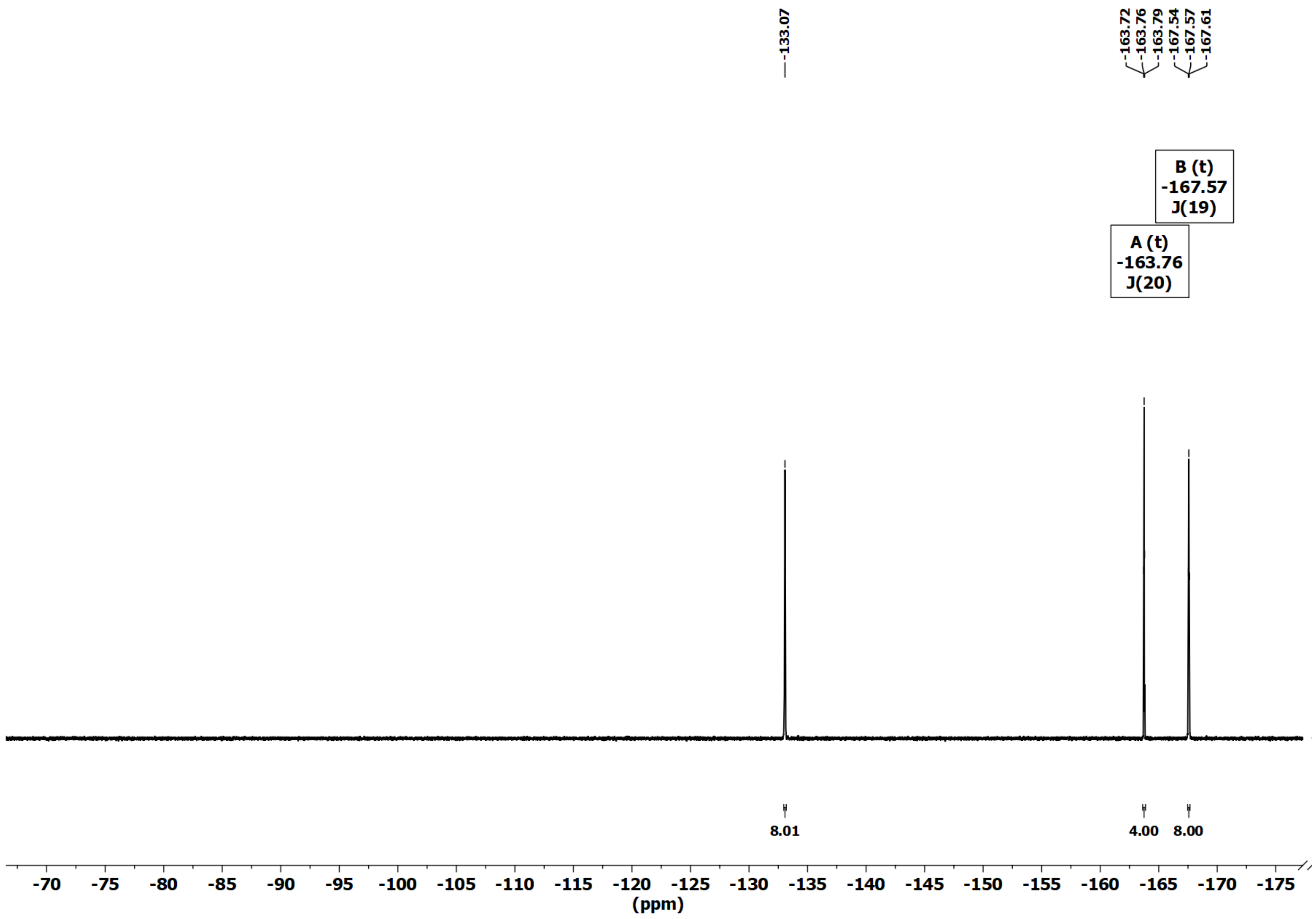

Figure S61. ^{19}F NMR (CD_2Cl_2, 565 MHz) spectrum of **6Se**.

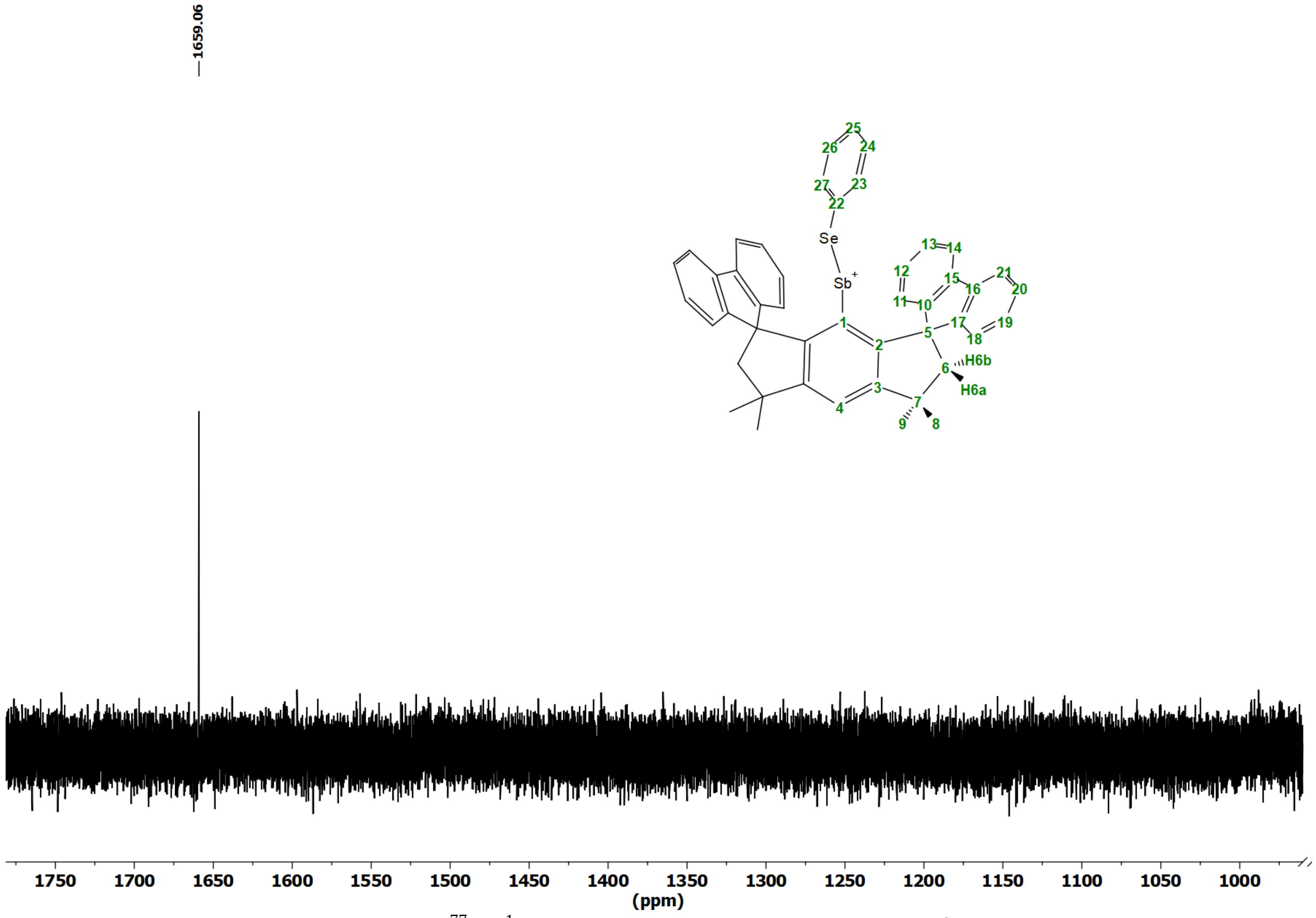

Figure S62. $^{77}Se\{^1H\}$ NMR (CD_2Cl_2, 115 MHz) spectrum of **6Se**.

Synthesis and characterization of [M^SFluindSbTePh][$B(C_6F_5)_4$] (6Te)

In a J. Young NMR Tube [M^SFluindSbH][$B(C_6F_5)_4$] (**4**) (20.0 mg, 15.2 µmol, 1.00 Eq.) and diphenyl ditelluride (3.11 mg, 7.60 mmol, 0.50 Eq.) were dissolved in benzene (0.5 mL) at room temperature. Then *n*-hexane (0.5 mL) was layered over the solution. The crystalline product was decanted and washed with *n*-hexane (3 × 1 mL) and dried at reduced pressure. The title compound **6Te** was obtained as a dark red crystalline solid (10.7 mg, 7.04 µmol, 46%).

^{1}H NMR (601 MHz, CD_2Cl_2): δ = 7.56 (dt, $^3J(^1H-^1H)$ = 7 Hz, $^4J(^1H-^1H)$ = 1 Hz, 2H, H14), 7.51 (s, 1H, H4), 7.48 – 7.43 (m, 7H, H11, H18, H21 and H25), 7.46 – 7.39 (m, 4H, H20, H24 and H26), 7.37 (td, $^3J(^1H-^1H)$ = 8 Hz, $^4J(^1H-^1H)$ = 1 Hz, 2H, H19), 7.37 – 7.31 (m, 2H, H13), 7.26 – 7.21 (m, 2H, H23 and H27), 7.20 (td, $^3J(^1H-^1H)$ = 8 Hz, $^4J(^1H-^1H)$ = 1 Hz, 2H, H12), 2.59 (d, $^3J(^1H-^1H)$ = 14 Hz, 2H, H6a), 2.52 (d, $^3J(^1H-^1H)$ = 14 Hz, 2H, H6b), 1.74 (s, 6H, H8), 1.63 (s, 6H, H9). **$^{13}C\{^1H\}$ NMR (151 MHz, CD_2Cl_2):** δ = 156.9 (s, C3), 153.5 (s, C2), 152.5 (s, C10), 148.8 (s, C17), 148.7 (d, br, $^1J(^{13}C–^{19}F)$ = 243 Hz, C_6F_5), 144.2 (s, C1), 142.1 (s, C16), 138.8 (d, br, $^1J(^{13}C–^{19}F)$ = 244 Hz, C_6F_5) ,138.4 (s, C15), 137.4 (s, C23 and C27), 136.9 (d, br, $^1J(^{13}C–^{19}F)$ = 240 Hz, C_6F_5) , 132.9 (s, C20), 131.7 (s, C13), 131.3 (s, C24 and C26), 131.0 (s, C12), 131.0 (s, C22), 129.5 (s, C19), 128.3 (s, C18), 126.8 (s, C11), 122.6 (s, C21 and C25), 122.3 (s, C14), 121.9 (s, C4) , 63.9 (s, C5), 54.0 (s, C6)*, 46.3 (s, C7), 33.3 (s, C9), 32.3 (s, C8). *signal overlaps with signal of CD_2Cl_2. **^{11}B NMR (193 MHz, CD_2Cl_2):** *δ* = −16.7 (s). **^{19}F NMR (565 MHz, CD_2Cl_2):** *δ* = −133.2 (br, 8F, *o*-C_6F_5), −163.4 (t, $^3J(^{19}F-^{19}F)$ = 20 Hz, 4F, *p*-C_6F_5), −167.4 (t, br, $^3J(^{19}F-^{19}F)$ = 20 Hz, 8F, *m*-C_6F_5).**^{125}Te NMR (189 MHz, CD_2Cl_2):** δ = 1526.5 (s). **HRMS ESI (m/z):** $[M]^+$ calculated for $C_{46}H_{38}SbTe$, 841.1068; found 841.1070. **M.p.:** > 200 °C (decomp.).

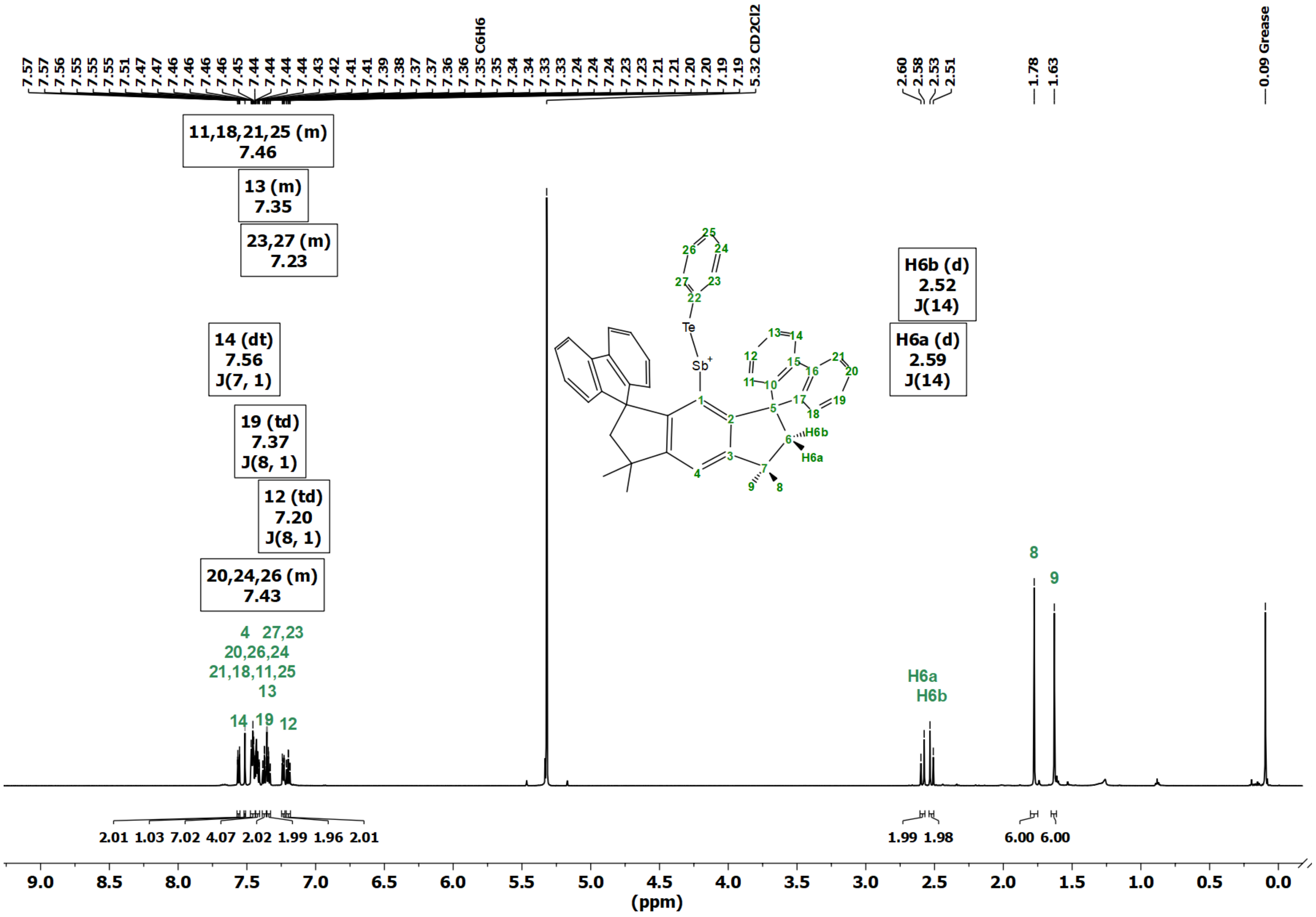

Figure S63. ^{1}H NMR (CD_2Cl_2, 600 MHz) spectrum of **6Te**.

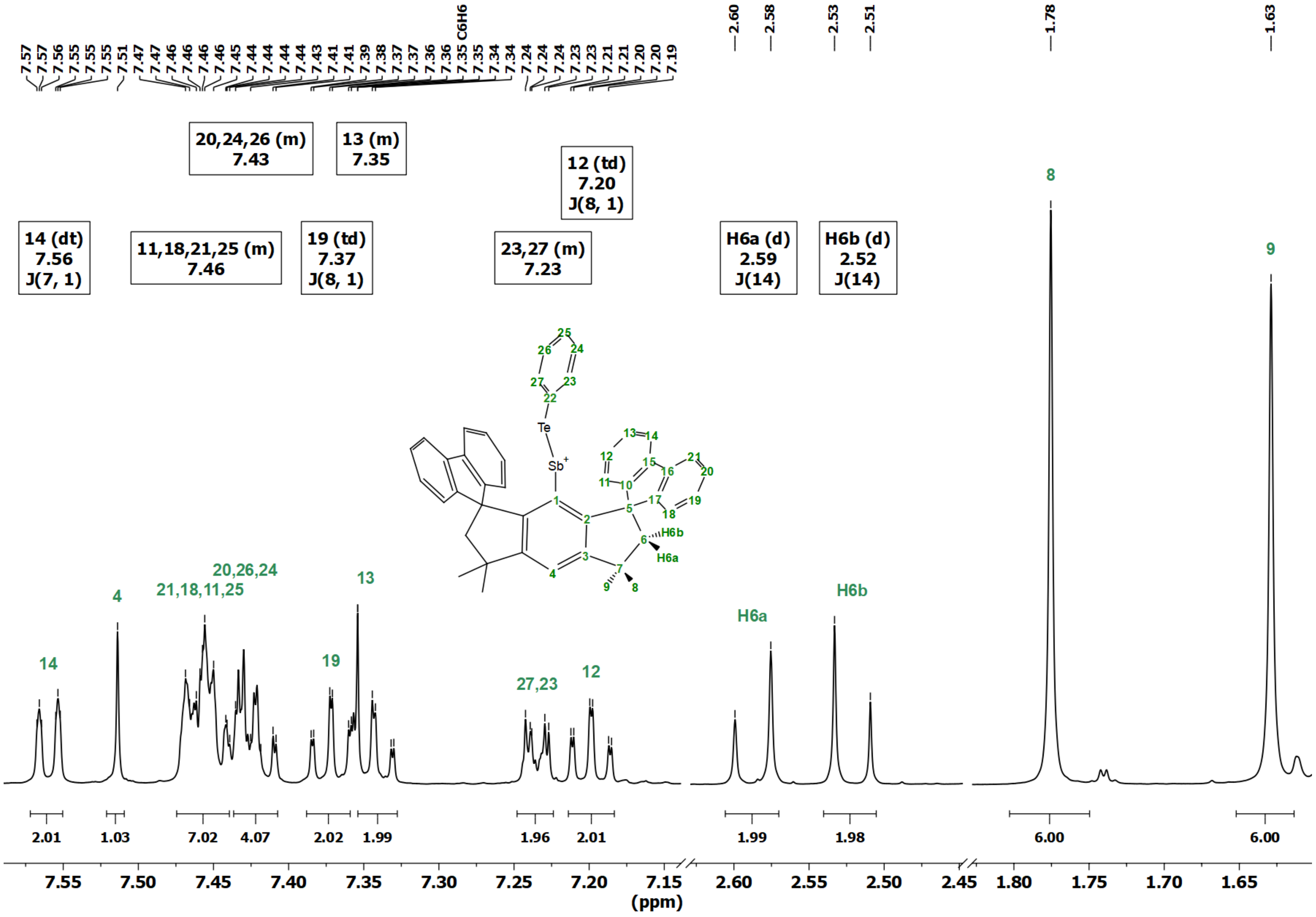

Figure S64. Detailed ^{1}H NMR (CD_2Cl_2, 600 MHz) spectrum of **6Te**.

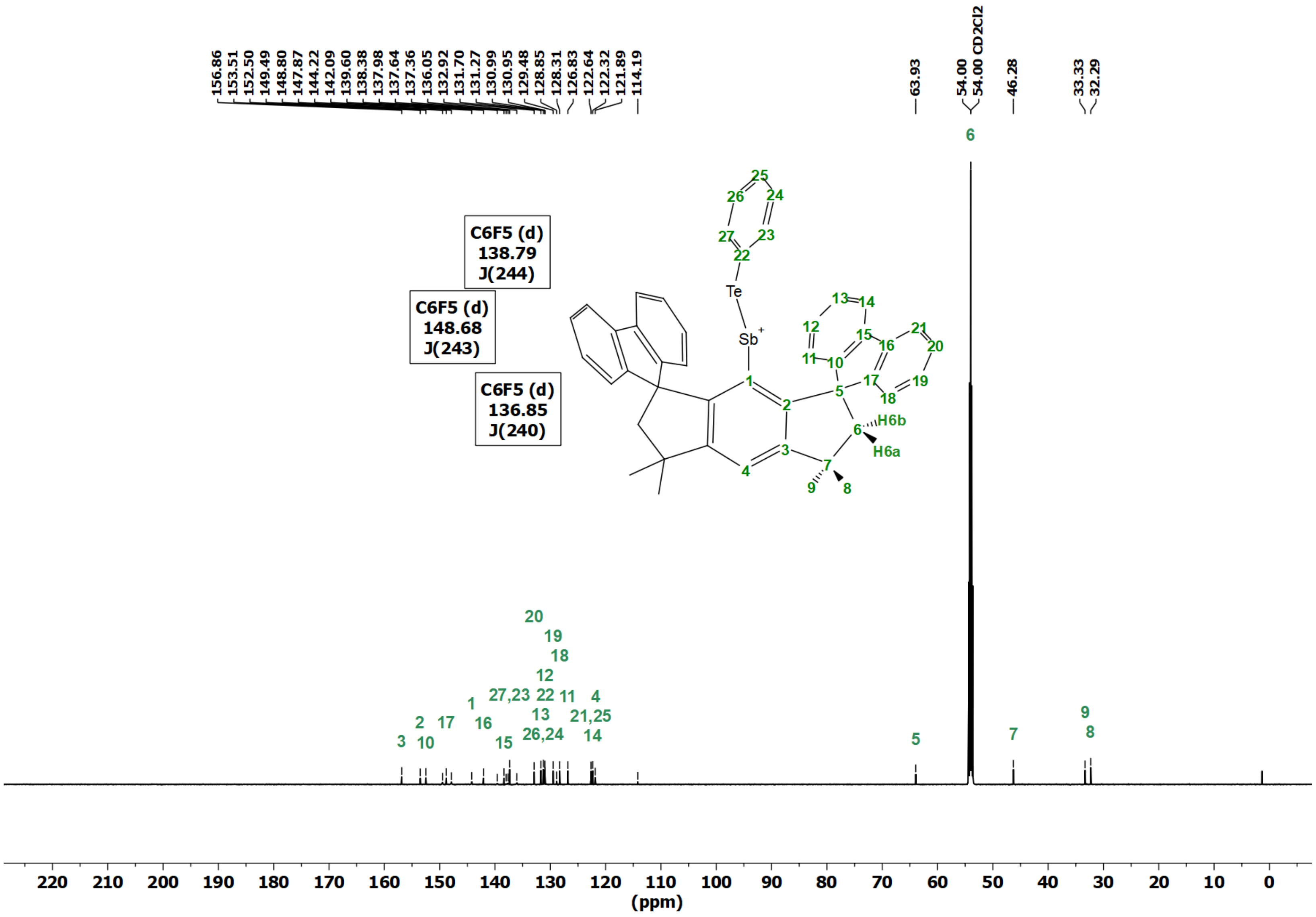

Figure S65. $^{13}C\{^{1}H\}$ NMR (CD_2Cl_2, 151 MHz) spectrum of **6Te**.

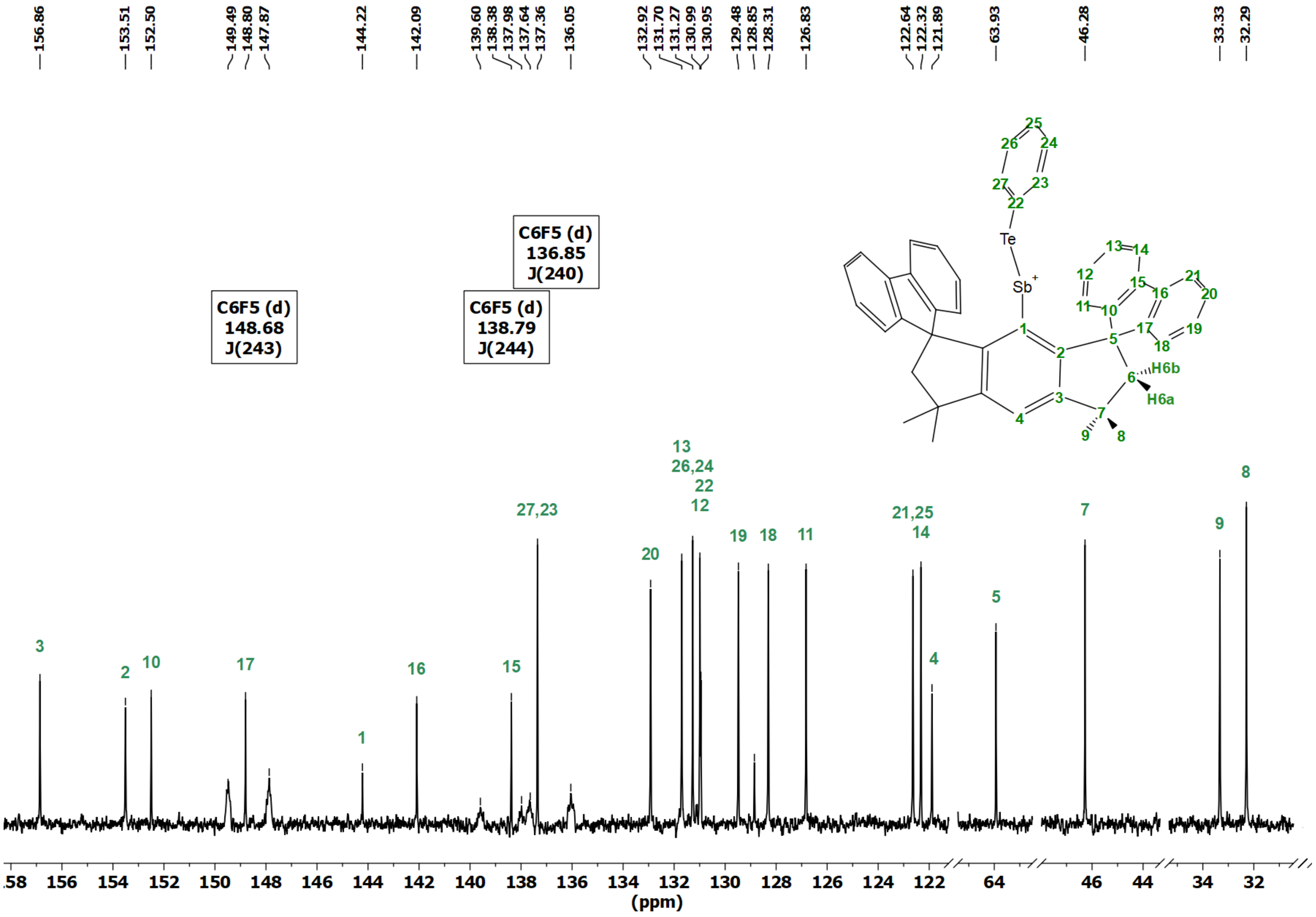

Figure S66. Detailed $^{13}C\{^{1}H\}$ NMR (CD_2Cl_2, 151 MHz) spectrum of **6Te**.

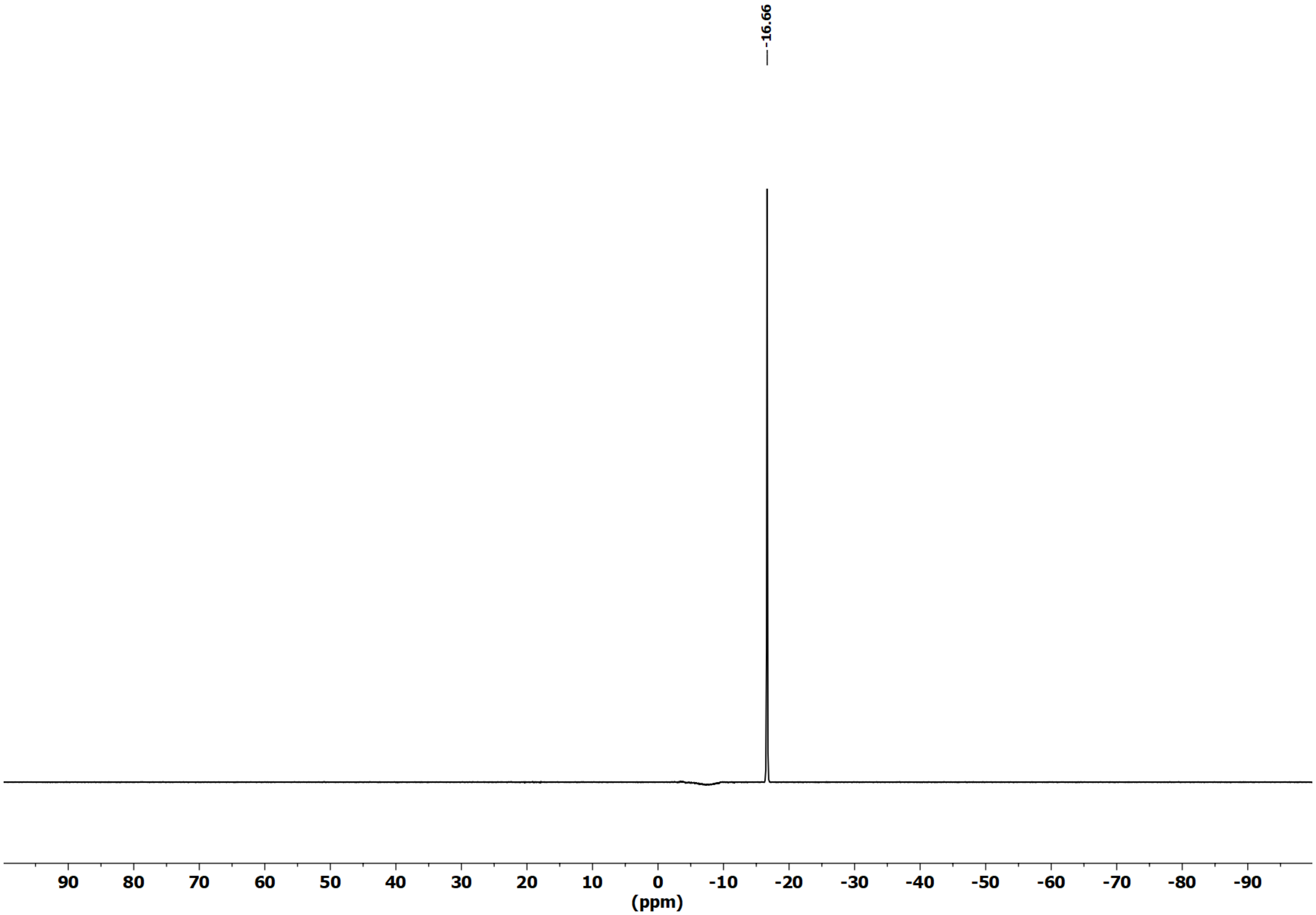

Figure S67. ^{11}B NMR (CD_2Cl_2, 193 MHz) spectrum of **6Te**.

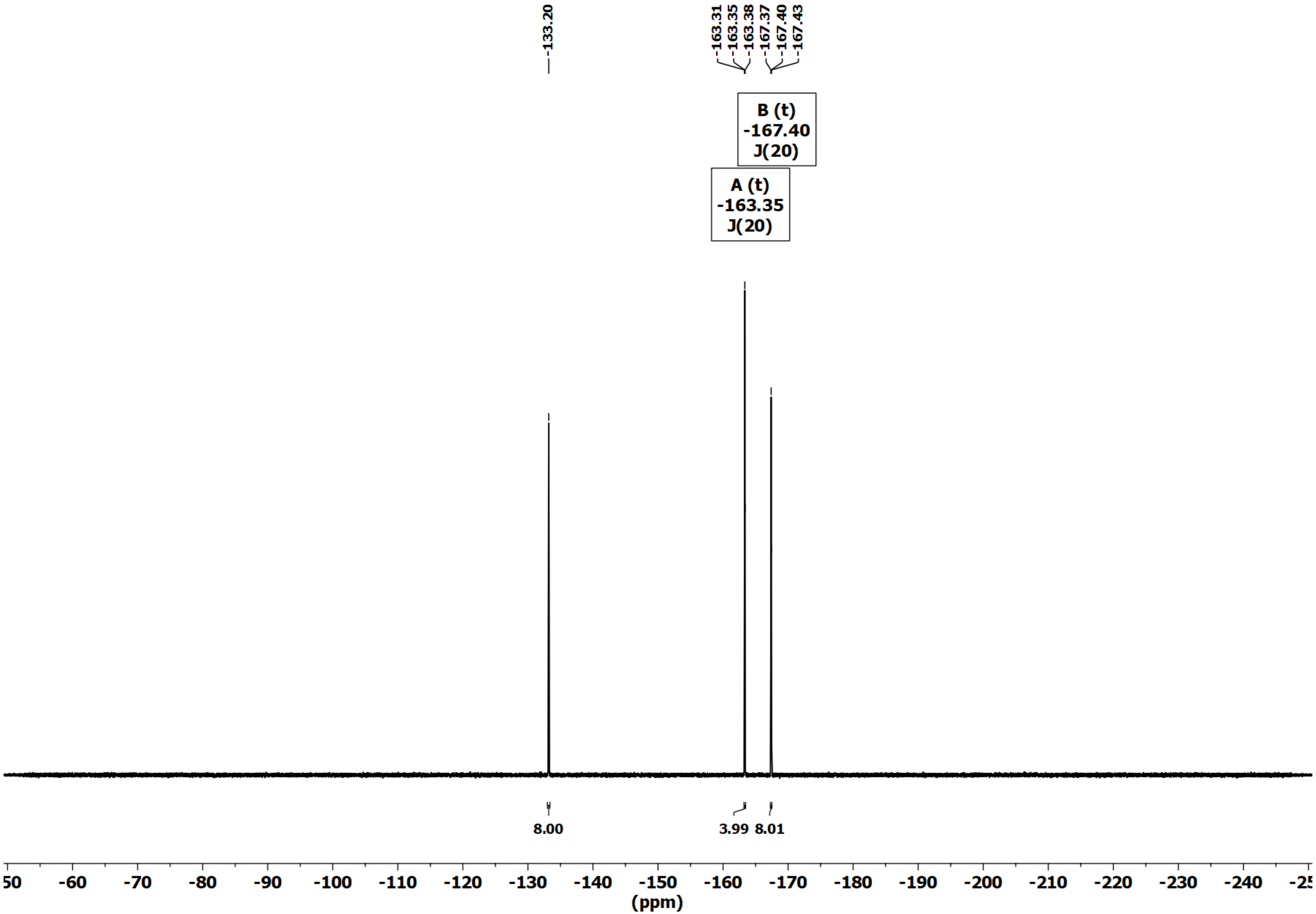

Figure S68. ^{19}F NMR (CD_2Cl_2, 565 MHz) spectrum of **6Te**.

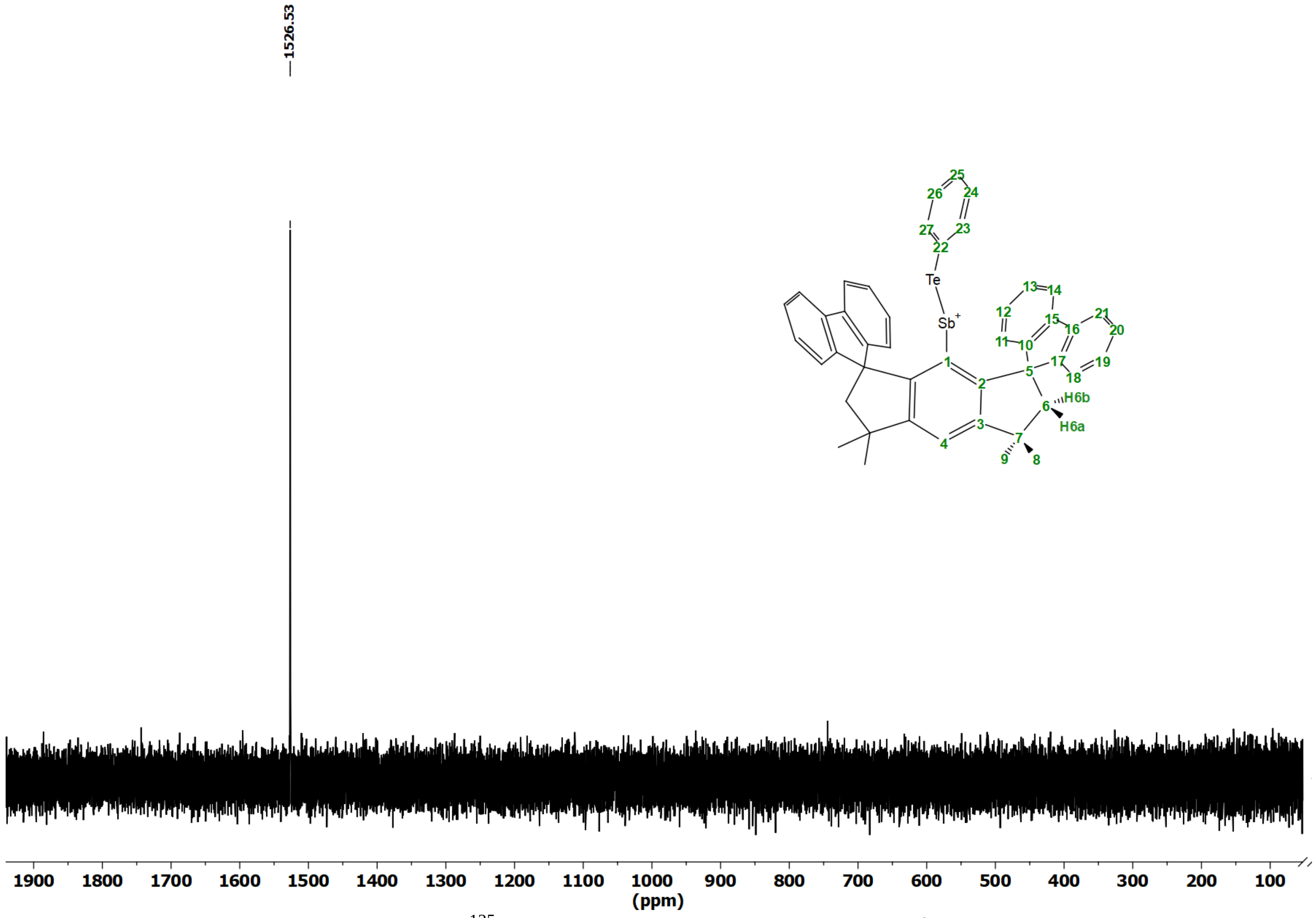

Figure S69. ^{125}Te NMR (CD_2Cl_2, 189 MHz) spectrum of **6Te**.

Synthesis and characterization of [M^{S}FluindBi$_3$][B(C_6F_5)$_4$]$_2$ (7)

1,2-dichlorobenzene (8 mL) and triethylsilane (106 μL, 0.628 mmol, 5.00 Eq) were added to [M^{S}FluindBiCl][B(C_6F_5)$_4$] (**2Bi**) (190 mg, 0.132 mmol) and the reaction mixture was stirred at room temperature for 2 hours resulting in a dark brown colour change of the reaction mixture. After cannula filtration, the reaction mixture was layered with *n*-hexane (16 mL). The resulting crystalline product was dried at reduced pressure to obtain [M^{S}FluindBi$_3$][B(C_6F_5)$_4$]$_2$ (**7**) as a dark brown crystalline solid (55.0 mg, 0.022 mmol, 17%).

^{1}H NMR (600 MHz, CD_2Cl_2): δ = 8.05 (d, ^3J = 8 Hz, 4H, H11 and H18),7.65 (s, 1H, H4), 7.45 (t, ^3J = 8 Hz, 4H, H13 and H20), 7.40 (d, ^3J = 8 Hz, 4H, H14 and H21), 6.99 (t, ^3J = 7.4 Hz, 4H, H12 and H19), 2.53 (s, 4H, H6a and H6b), 1.71 (s, 12H, H8 and H9). **^{13}C{^{1}H} NMR (151 MHz, CD_2Cl_2):** 179.5 (s, C2), 162.0 (s, C2), 155.8 (s, C10 and C17), 148.7 (d, br, $^1J(^{13}C–^{19}F)$ = 240 Hz, C_6F_5), 144.0 (s, C15 and C16), 138.9 (d, br, $^1J(^{13}C–^{19}F)$ = 246 Hz, C_6F_5), 138.2 (s, C4), 137.0 (d, br, $^1J(^{13}C–^{19}F)$ = 247 Hz, C_6F_5), 133.9 (s, C12 and C19), 132.7 (s, C13 and C20), 128.2 (s, C11 and C18), 125.8 (s, C14 and C21), 64.6 (s, C5), 51.8 (s, C6), 45.4 (s, C7), 32.3 (s, C8 and C9), C1 was not observed. **^{11}B NMR (193 MHz, CD_2Cl_2):** δ = −16.6 (s). **^{19}F NMR (565 MHz, CD_2Cl_2):** δ = −132.3 (br, 8F, *o*-C_6F_5), −162.5 (t, $^3J(^{19}F-^{19}F)$ = 21 Hz, 4F, *p*-C_6F_5), −166.1 (t, br, $^3J(^{19}F-^{19}F)$ = 20 Hz, 8F, *m*-C_6F_5). **HRMS ESI (m/z):** $[M]^{2+}$ calculated for $C_{40}H_{33}Bi_3$, 570.09916; found 570.09787; $[M+B(C_6F_5)_4]^+$ calculated for $C_{64}H_{33}BBi_3F_{20}$, 1819.17728; found 1819.18292. **Mp.** 208-210 °C.

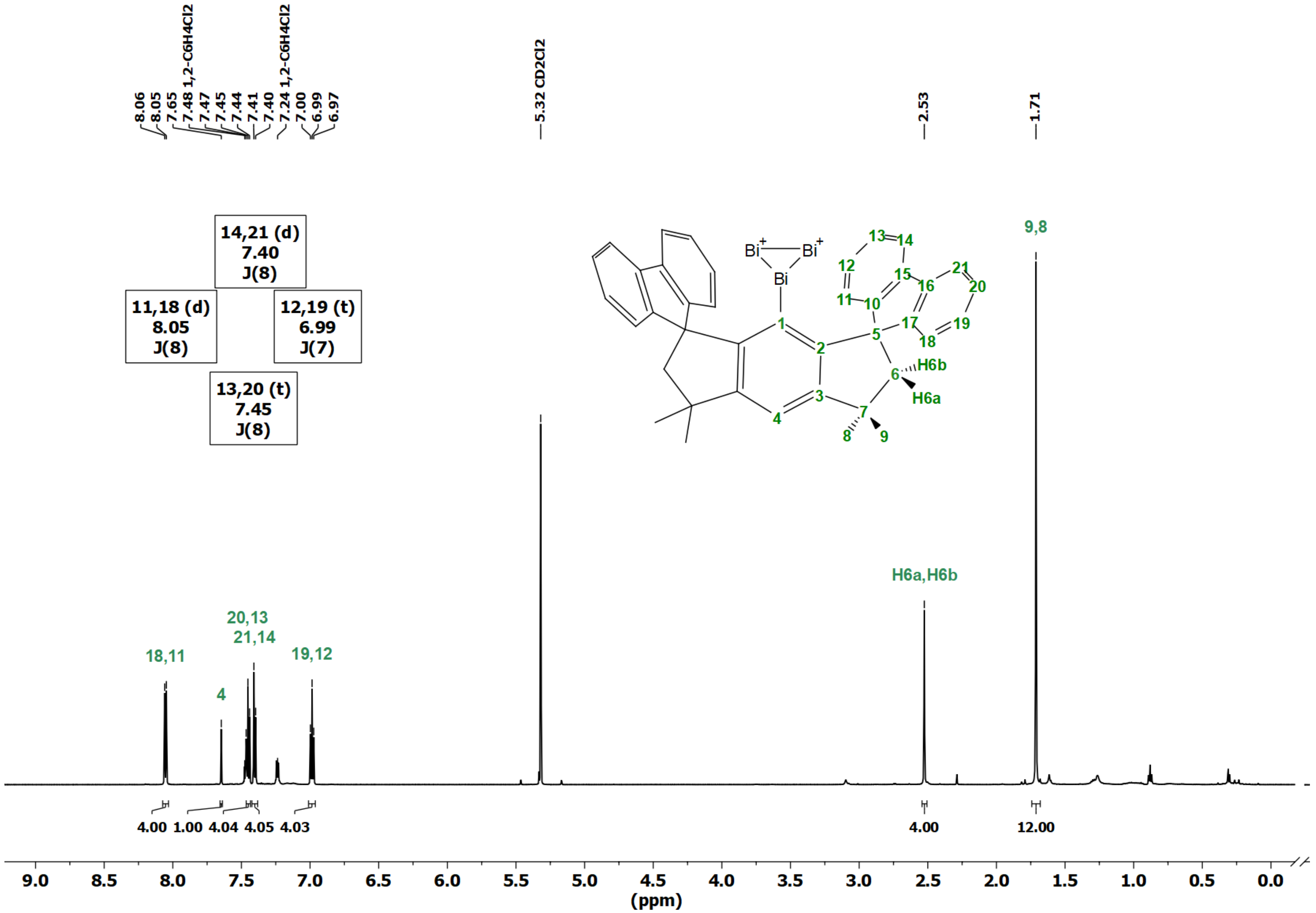

Figure S70. ^{1}H NMR (CD_2Cl_2, 600 MHz) spectrum of **7**.

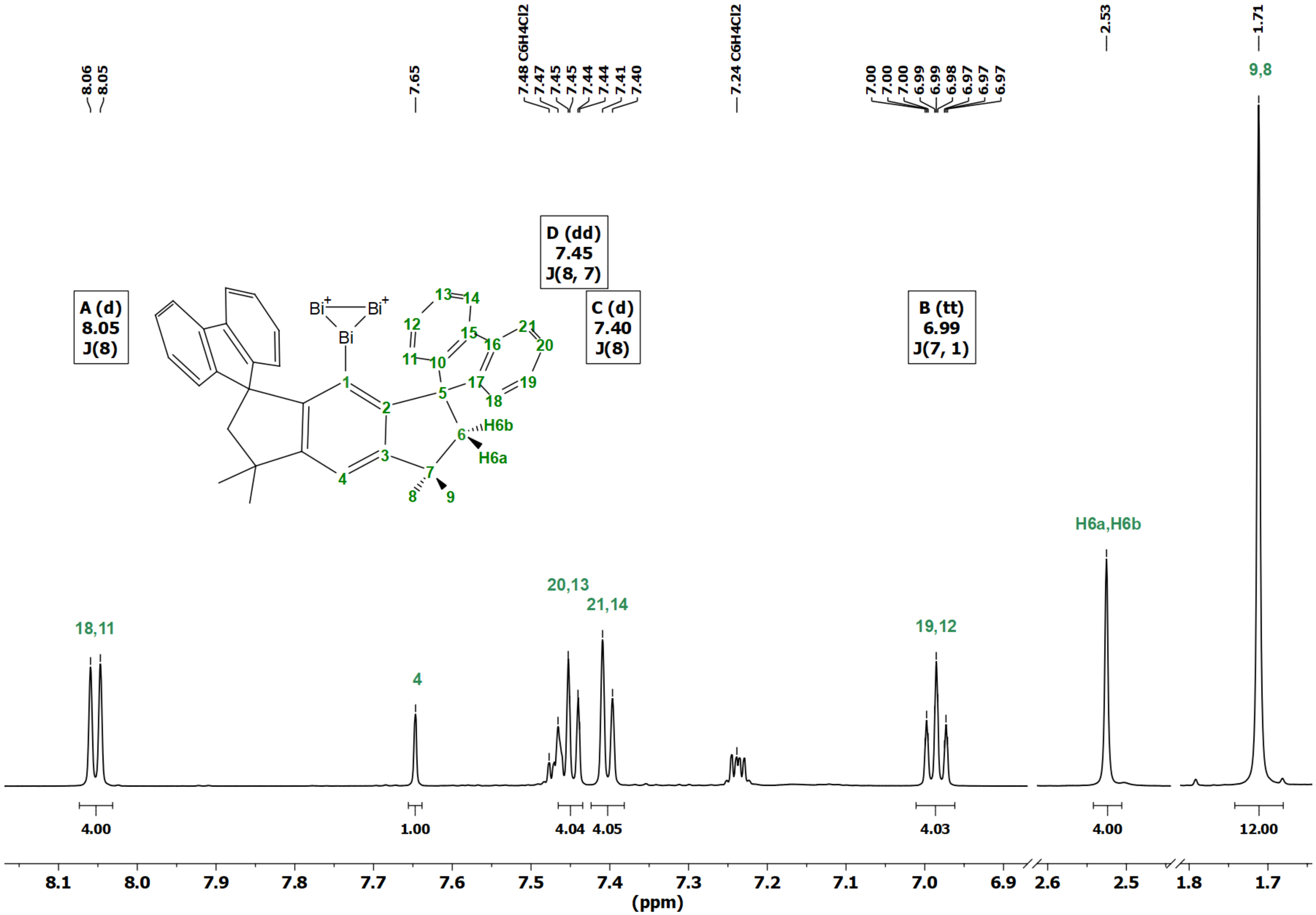

Figure S71. ^{1}H NMR (CD_2Cl_2, 600 MHz) spectrum of **7**.

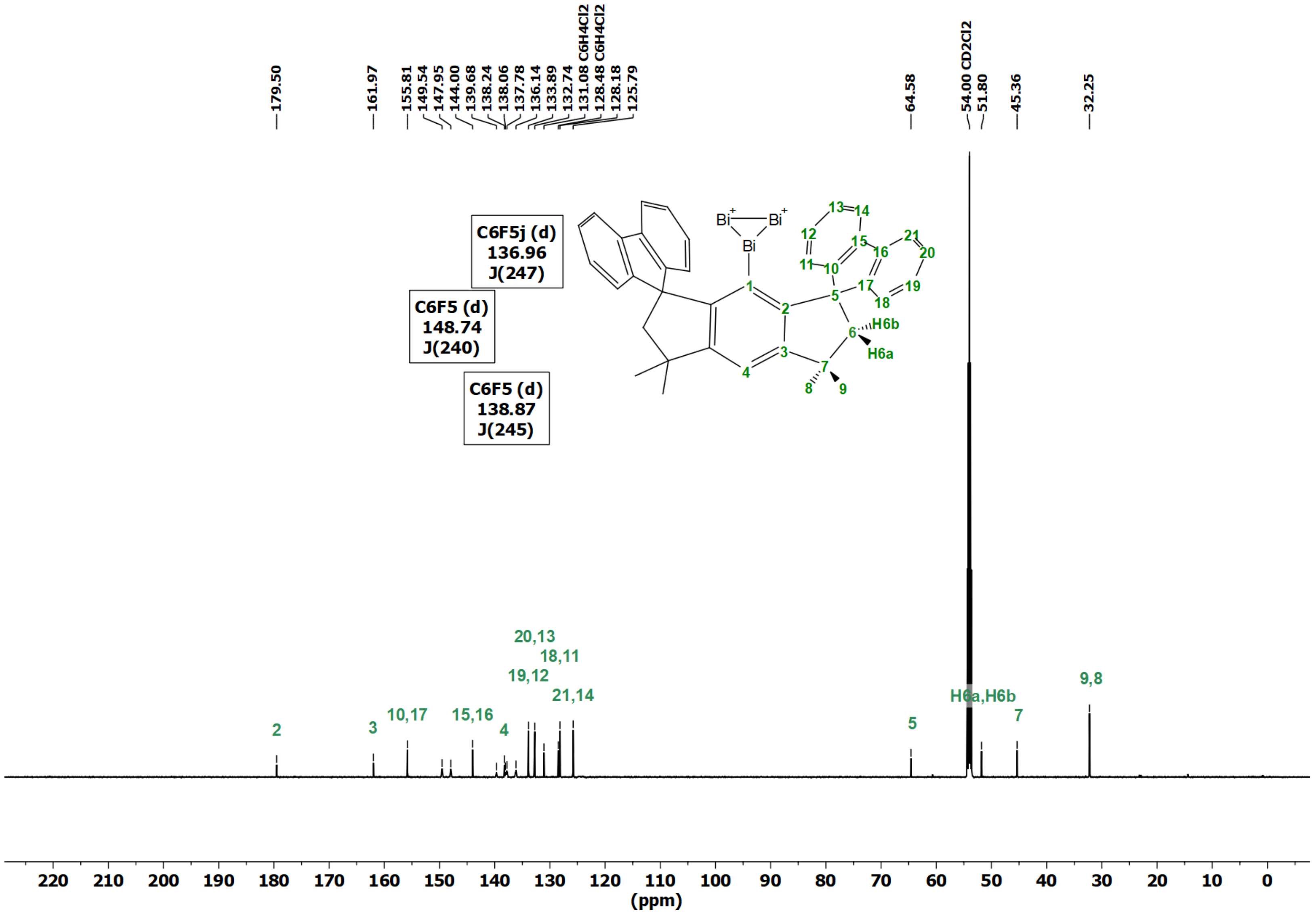

Figure S72. $^{13}C\{^{1}H\}$ NMR (CD_2Cl_2, 151 MHz) spectrum of 7.

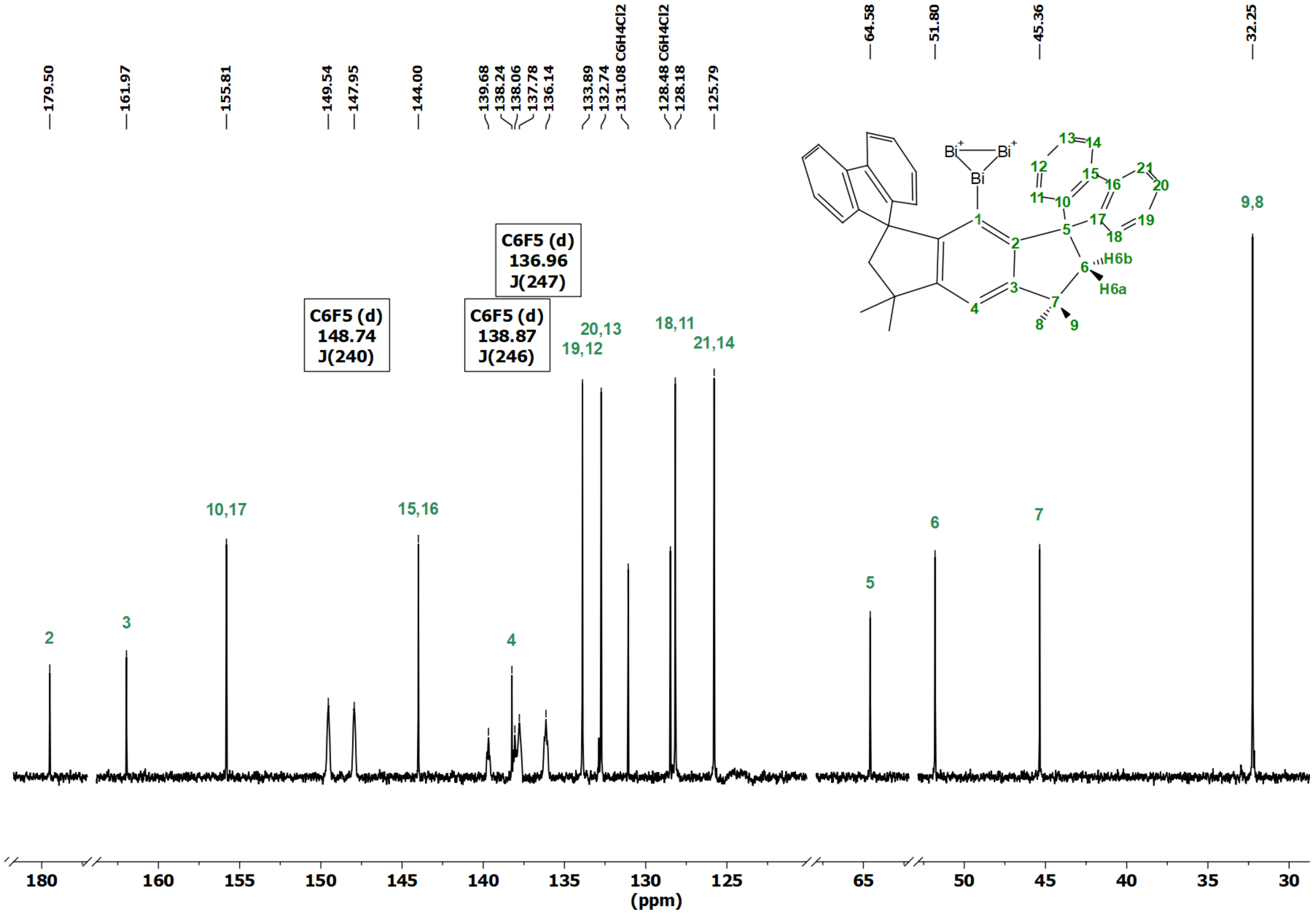

Figure S73. $^{13}C\{^{1}H\}$ NMR (CD_2Cl_2, 151 MHz) spectrum of **7**.

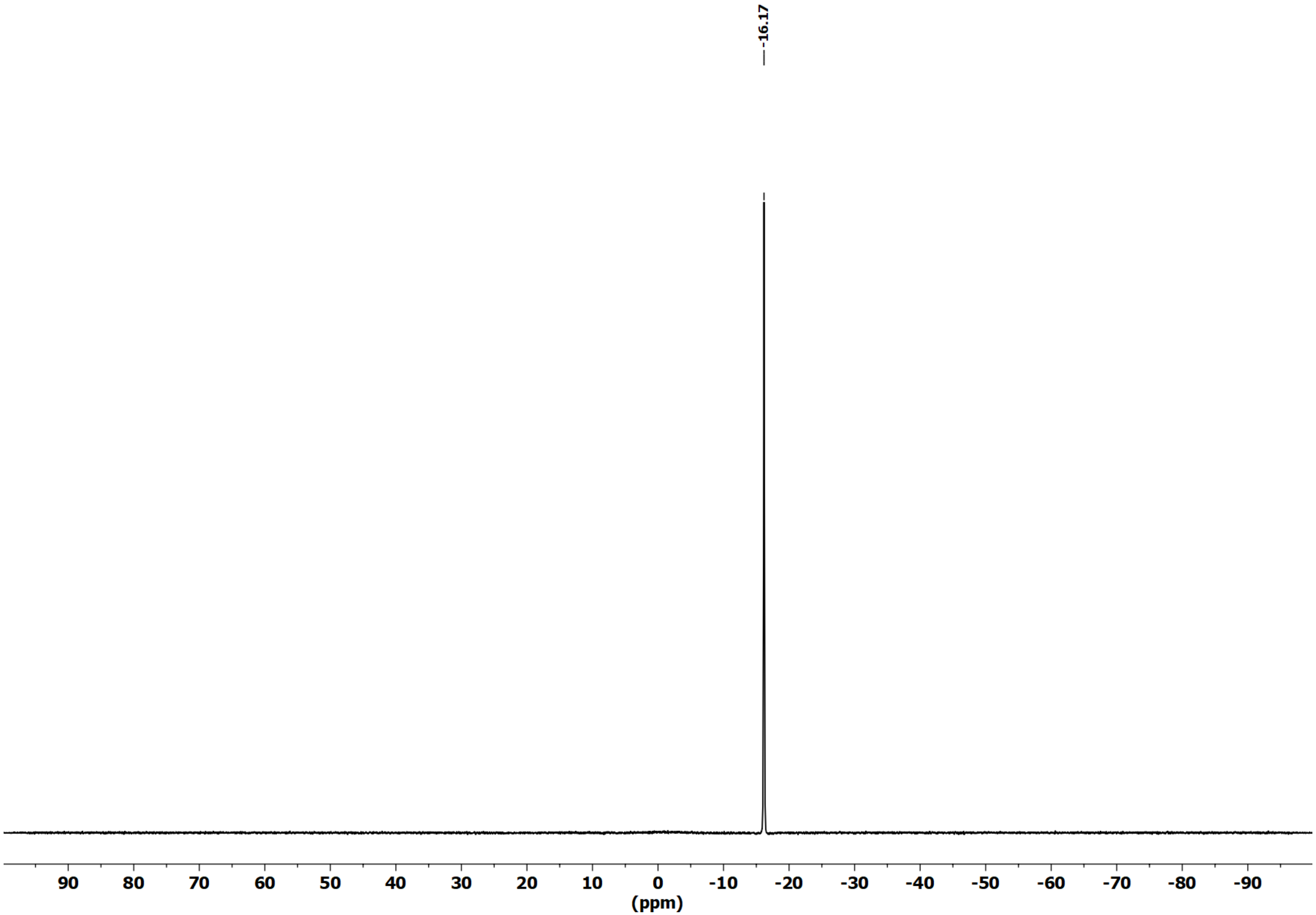

Figure S74. ^{11}B NMR (CD_2Cl_2, 193 MHz) spectrum of **7**.

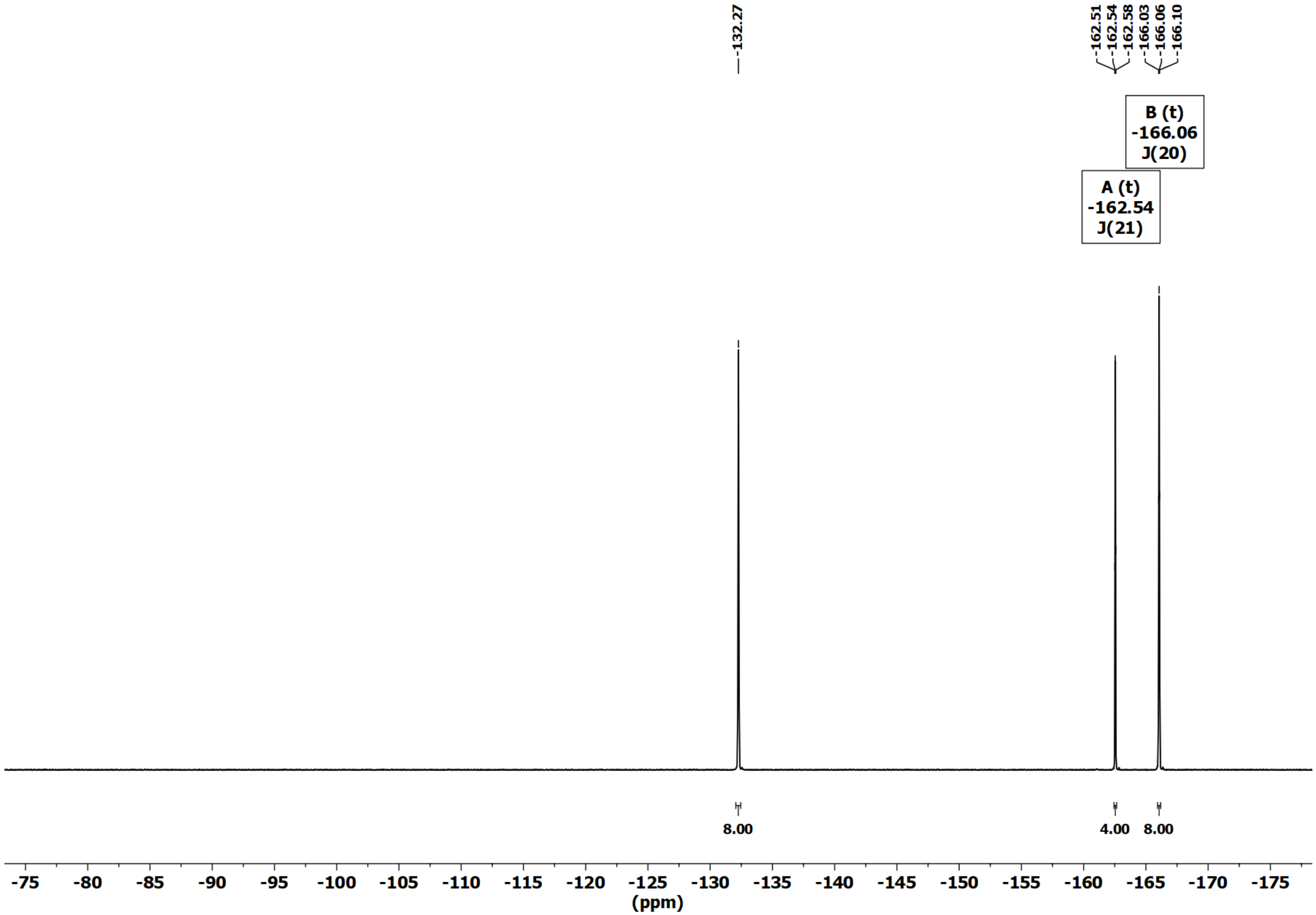

Figure S75: ^{19}F NMR (CD_2Cl_2, 565 MHz) spectrum of **7**.

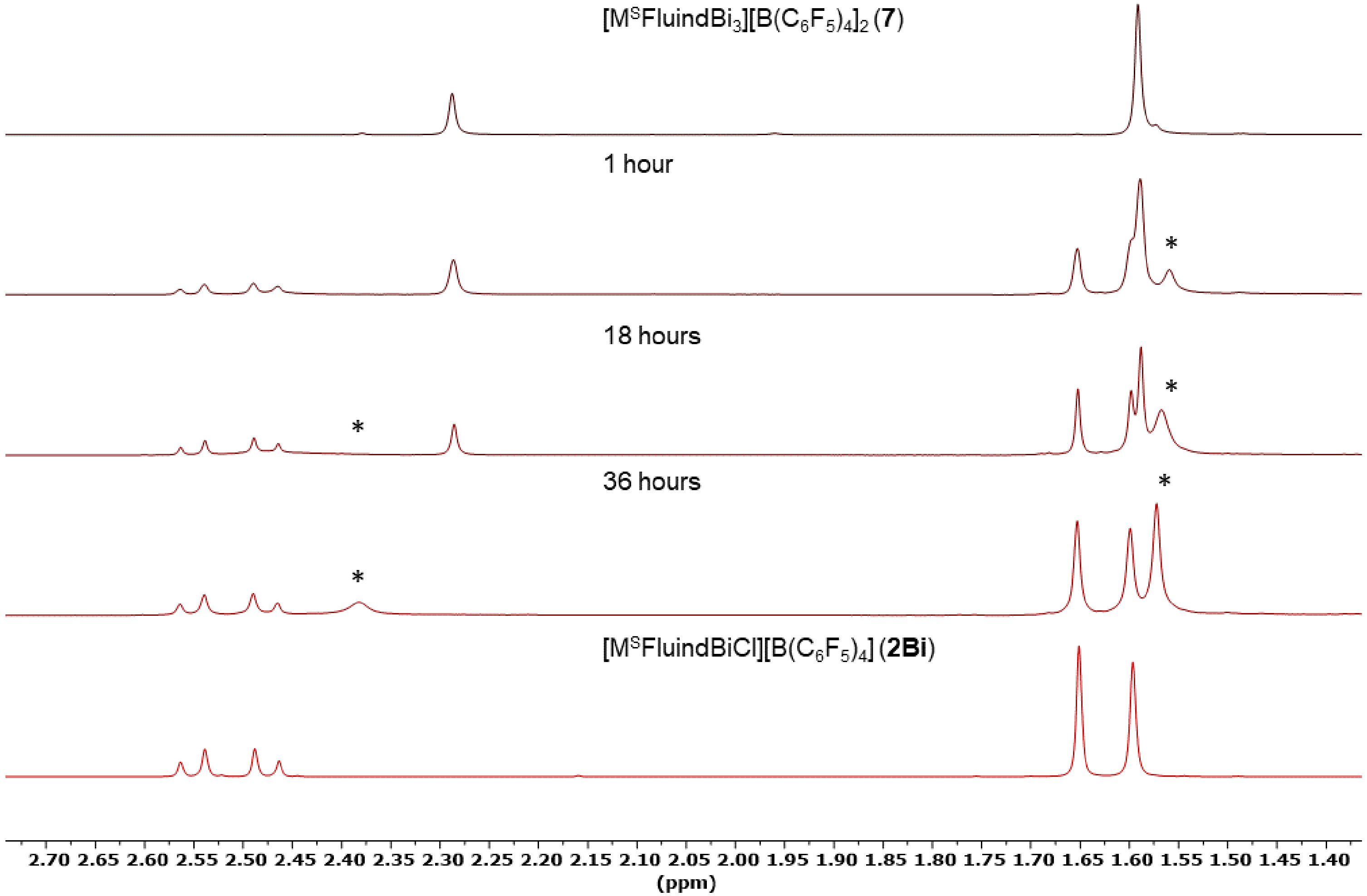

Figure S76. Stacked ^{1}H NMR (CD_2Cl_2, 600 MHz) spectra of **7** upon heating at 80°C. Spectra are recorded at the specified times and the formation of M^{S}FluindH is denoted with an asterisk. Bottom: Pure **2Bi** is shown as reference.

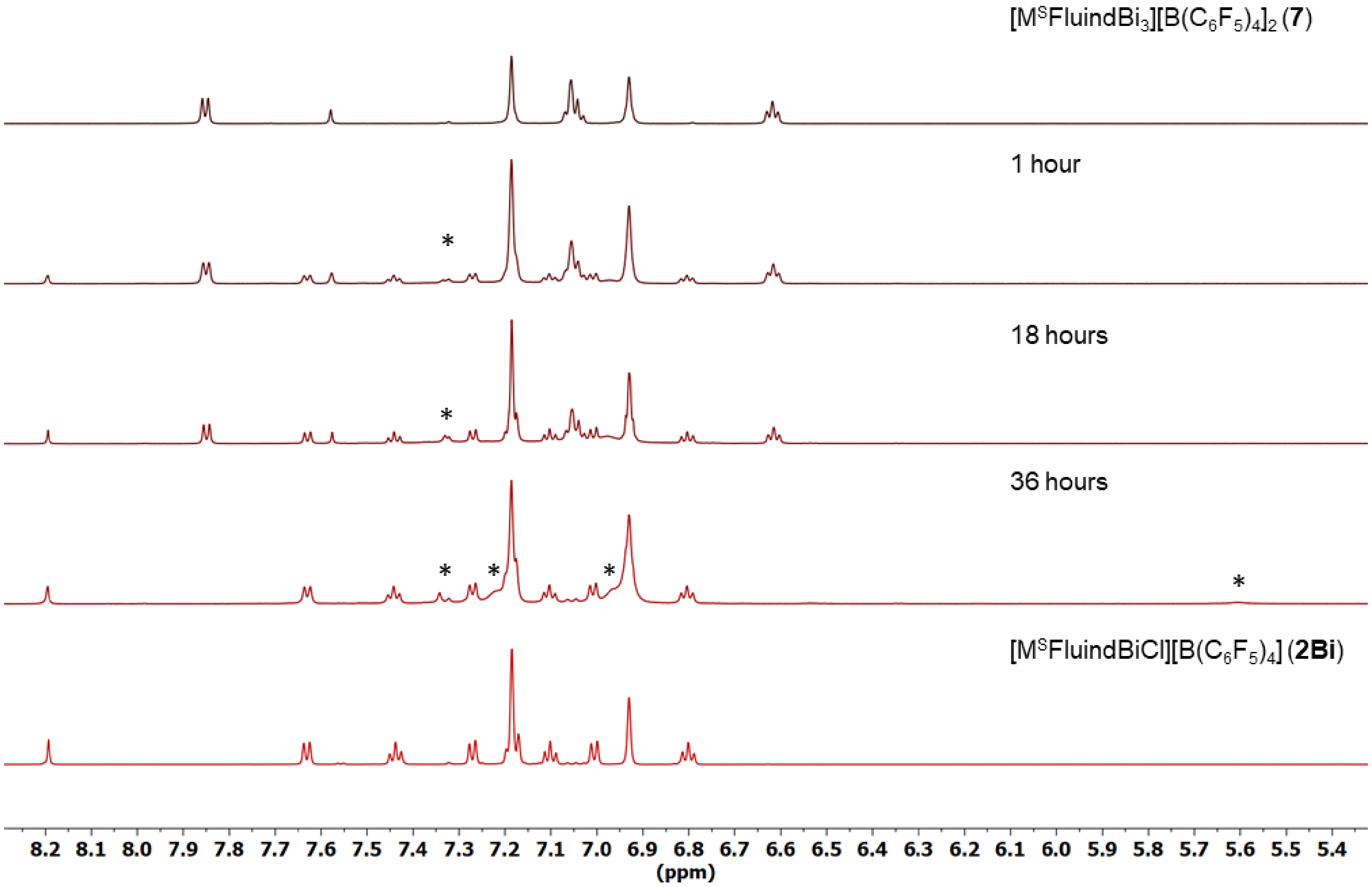

Figure S77. Stacked ^{1}H NMR (CD_2Cl_2, 600 MHz) spectra of **7** upon heating at 80°C. Spectra are recorded at the specified times and the formation of M^{S}FluindH is denoted with an asterisk. Bottom: Pure **2Bi** is shown as reference

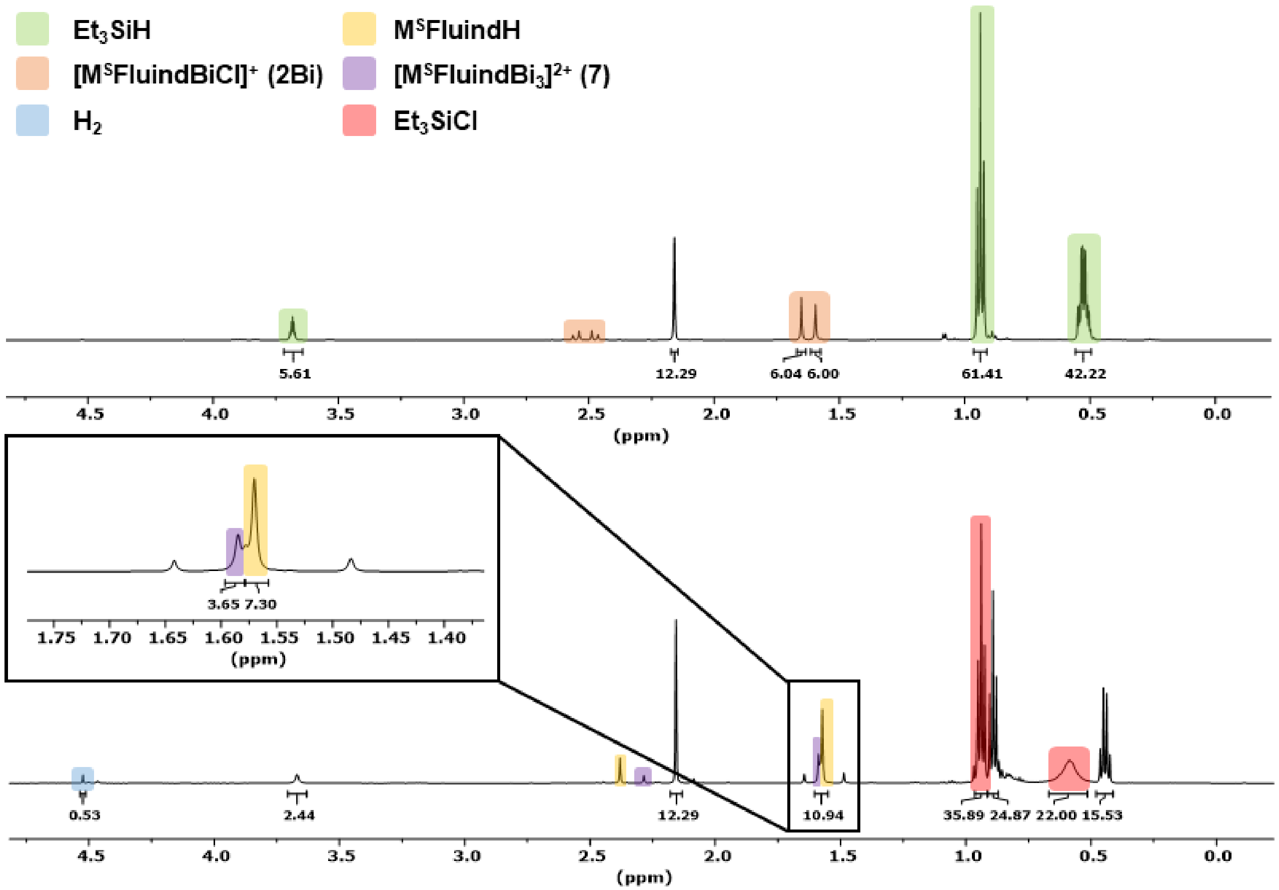

Figure S78. *Top:* ^{1}H NMR (CD_2Cl_2, 600 MHz) spectrum of the reaction of **2Bi** with 5 eq. of Et_3SiH using mesitylene as internal standard. Integral set to the CH_3 group of the M^{S}Fluind substituent, so that this represents 1 eq. of **2Bi**. The resulting integral of the mesitylene (12.29) is then used for the bottom spectrum. *Bottom:* ^{1}H NMR (CD_2Cl_2, 600 MHz) spectrum after completion of the reaction. Formation of **7** and M^{S}FluindH and H_2 in a ratio of 1:2:1. Formation of Et_3SiCl is also detected, however respective Et signals are overlaid with different triethylsilyl species.

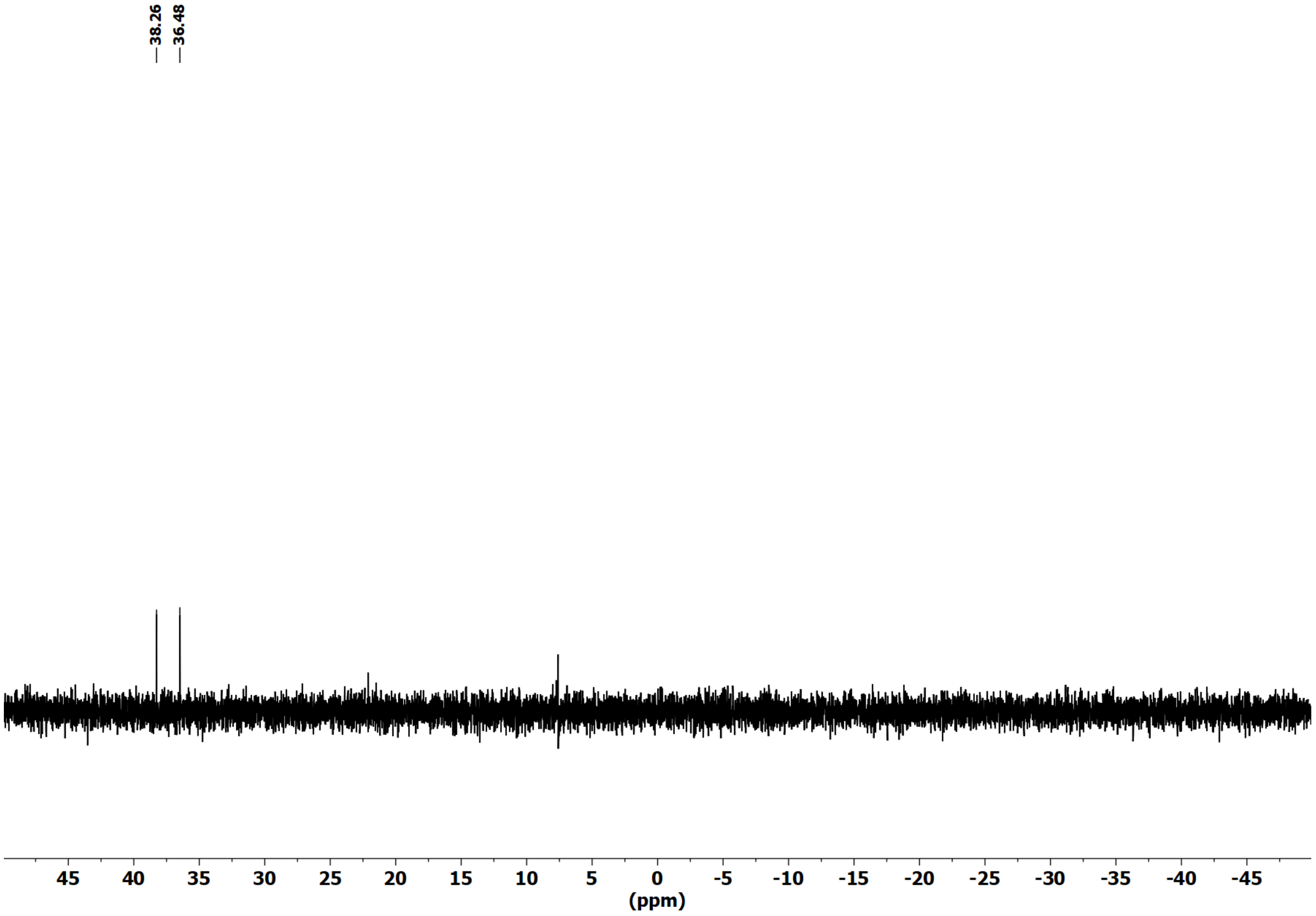

Figure S79. $^{29}Si\{^{1}H\}$ NMR (CD_2Cl_2, 119 MHz) spectrum of the reaction of **2Bi** with 5 eq. of Et_3SiH after the completion of the reaction. The chemical shift at 36.5 ppm is assigned to Et_3SiCl.

Determination of Gutmann-Beckett acceptor numbers

Lewis acidity determination experiments by the Gutmann-Beckett method were carried out according to the reported procedure.[13] A J. Young NMR tube was charged with ca. 10-15 mg of the chlorophosphenium salt with one eq. of Et_3PO (ca. 1 mg) in CD_2Cl_2. Using an excess of Et_3PO (ca. 3 mg, 2−3 eq.) in CD_2Cl_2 resulted for **2As** and **4** in the formation of an additional high-field shift, presumably due to the coordination of a second molecule Et_3PO. The ^{31}P NMR spectra were recorded at room temperature (22 °C) and the chemical shifts are listed in the table below. The acceptor numbers (AN) were determined from the following equation:

$$AN = \frac{\delta_{interacting\ OPEt_3} - 41.0}{86.14 - 41.0} \cdot 100$$

Table S1. Gutmann-Beckett acceptor numbers

Compound	^{31}P NMR OPEt$_3$ (ppm)	Acceptor Number (AN)
**[Ar^1AsCl][$B(C_6F_5)_4$]**	99.50	129.6
**[Ar^1SbCl][$B(C_6F_5)_4$]**	91.07	110.9
**[Ar^1BiCl][$B(C_6F_5)_4$]**	72.95	70.8
**[Ar^1SbH][$B(C_6F_5)_4$]**	86.20	100.1
**[Ar^1Bi_3][$B(C_6F_5)_4$]$_2$**	65.53	54.3

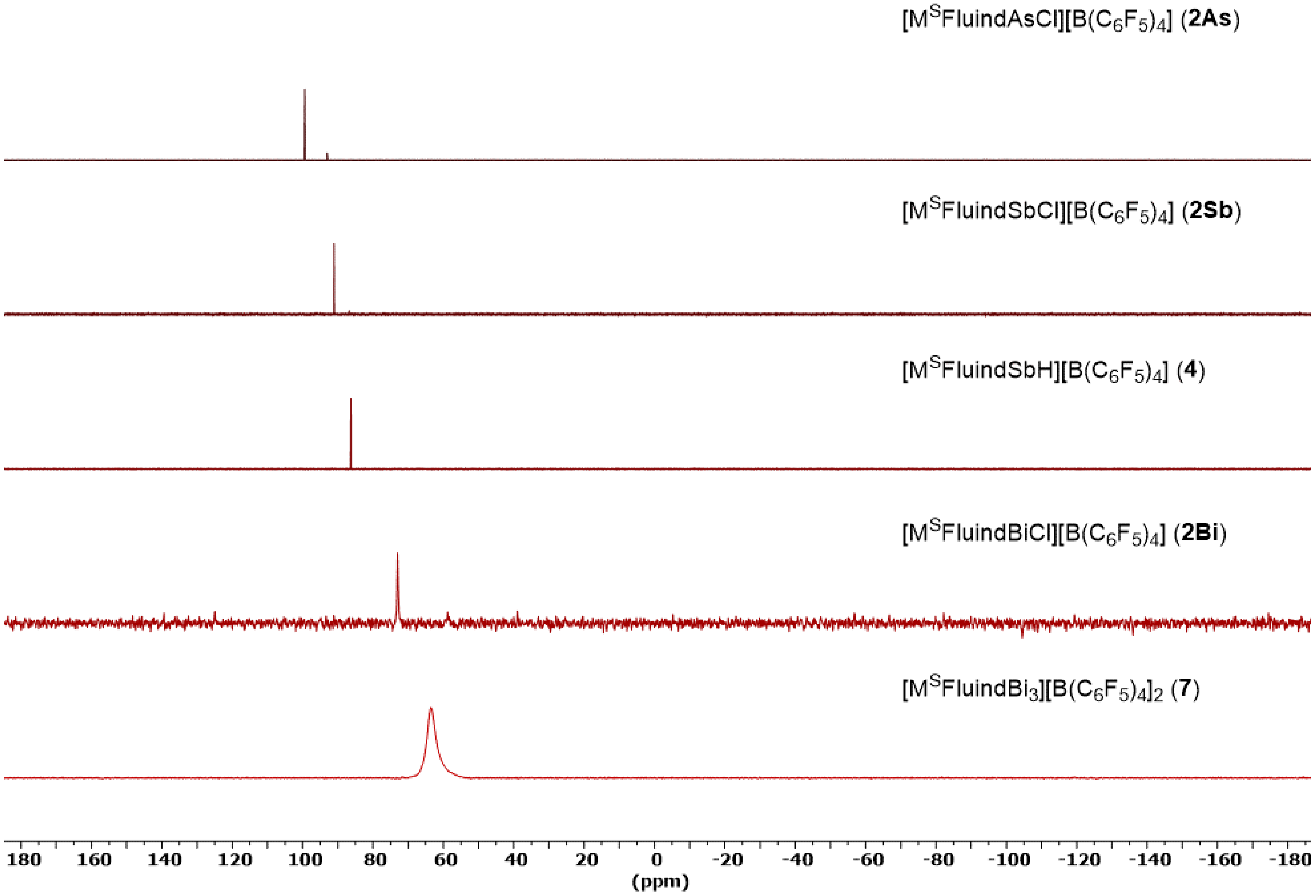

Figure S80. Stacked ^{31}P NMR (CD_2Cl_2, 243 MHz) spectra for the Lewis acidity determination experiments by the Gutmann-Beckett method of **2As**, **2Sb**, **2Bi**, **4**, and **7** using 1 eq. of Et_3PO.

UV-vis absorption spectra

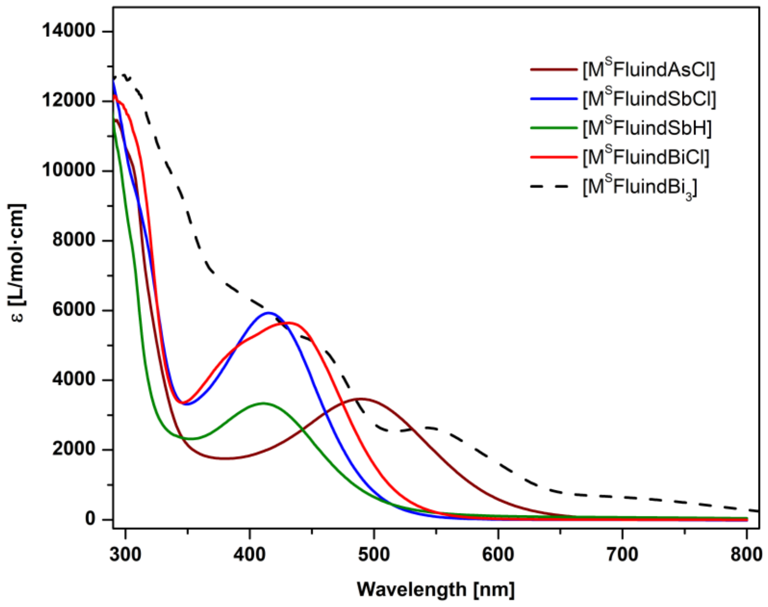

Figure S81: UV-vis absorption spectra of **2As**, **2Sb**, **2Bi**, **4** and **7** in CH_2Cl_2 (0.25 mM). Absorption maxima λ_{max} = 490 nm for [M^{S}FluindAsCl][B(C_6F_5)$_4$] (**2As**), λ_{max} = 417 nm for [M^{S}FluindSbCl][B(C_6F_5)$_4$] (**2Sb**), λ_{max} = 409 nm for [M^{S}FluindSbH][B(C_6F_5)$_4$] (**4**), λ_{max} = 430 nm for [M^{S}FluindBiCl][B(C_6F_5)$_4$] (**2Bi**), λ_{max} = 730 nm, 545 nm, 460 nm and 410 nm for [M^{S}FluindBi$_3$][B(C_6F_5)$_4$]$_2$ (**7**).

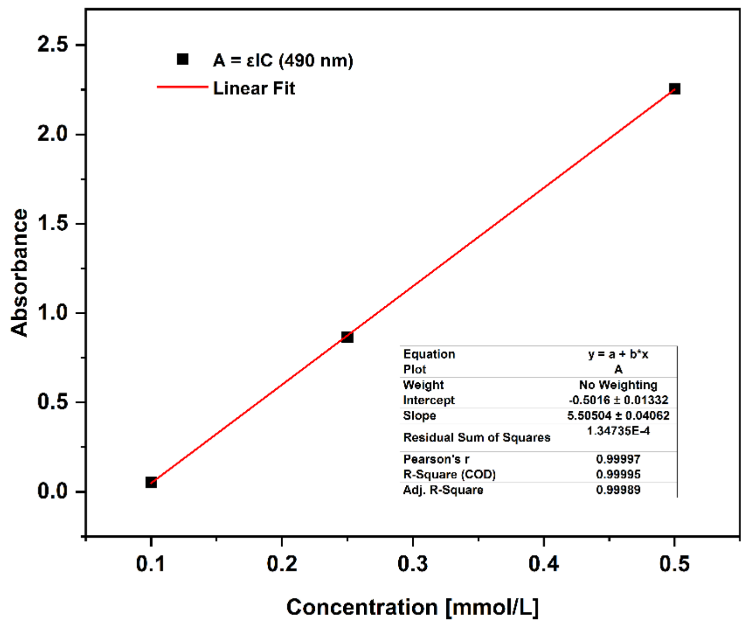

Figure S82. Concentration–absorbance dependence at 490 nm, absorption maximum of [M^{S}FluindAsCl][B(C_6F_5)$_4$] (**2As**) in CH_2Cl_2. Molar absorptivity in the presented range ($\varepsilon_{490\ nm}$ = 5505 cm^{-1} M) was determined using the Lambert–Beer law.

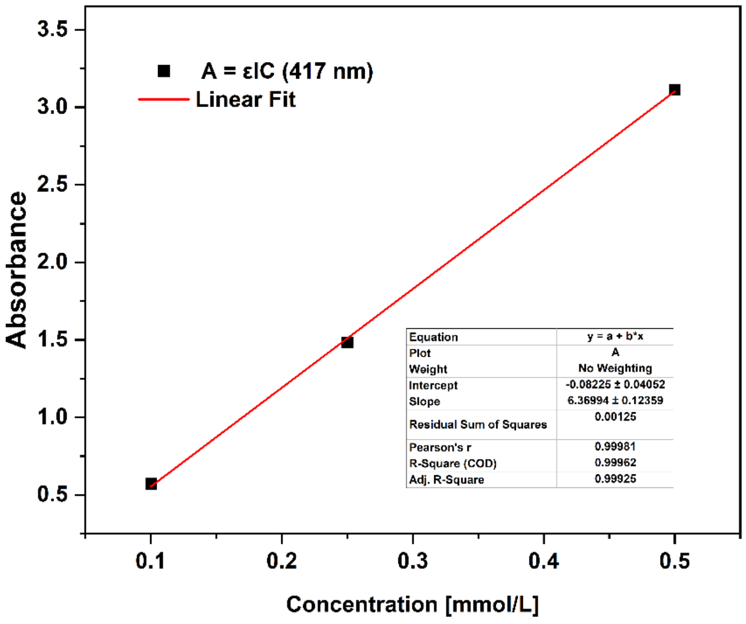

Figure S83. Concentration–absorbance dependence at 417 nm, absorption maximum of $[M^{S}FluindSbCl][B(C_6F_5)_4)]$ (**2Sb**) in CH_2Cl_2. Molar absorptivity in the presented range ($\varepsilon_{417\ nm}$ = 6370 cm^{-1} M) was determined using the Lambert–Beer law.

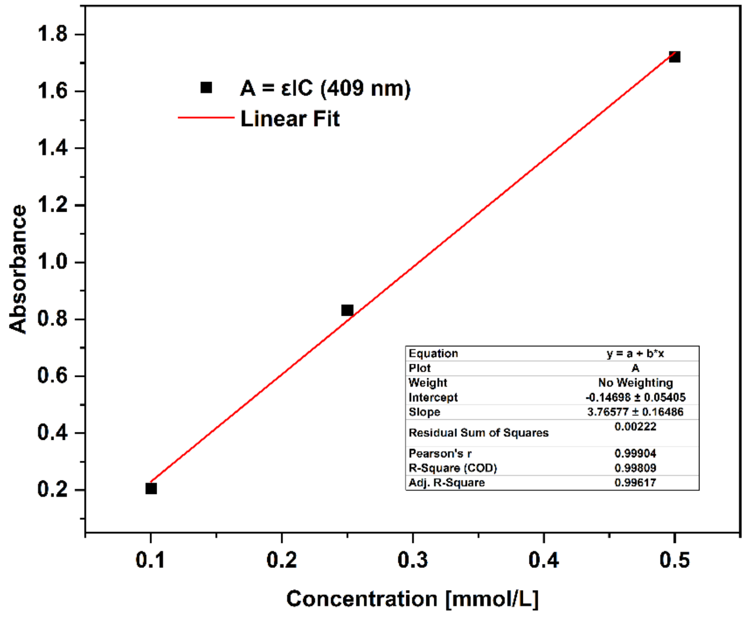

Figure S84. Concentration–absorbance dependence at 417 nm, absorption maximum of $[M^{S}FluindSbH][B(C_6F_5)_4)]_2$ (**4**) in CH_2Cl_2. Molar absorptivity in the presented range ($\varepsilon_{409\ nm}$ = 3766 cm^{-1} M) was determined using the Lambert–Beer law.

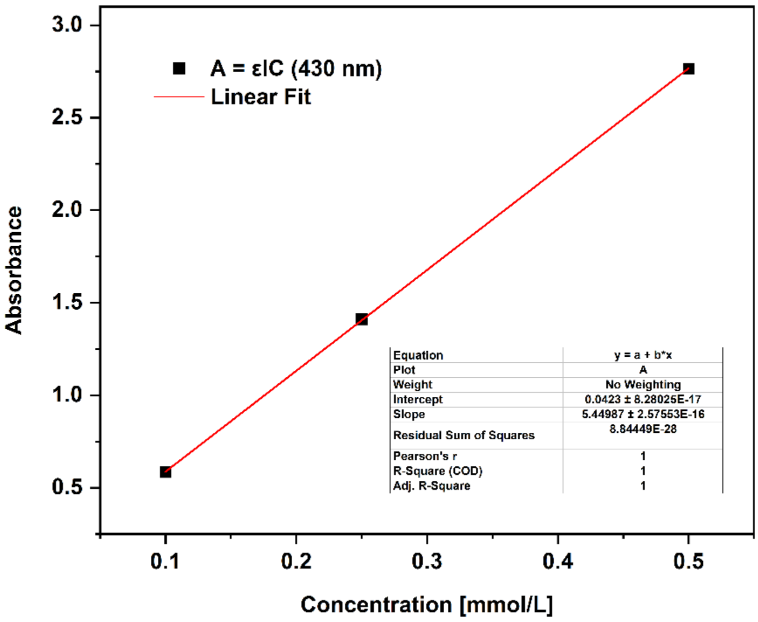

Figure S85. Concentration–absorbance dependence at 430 nm, absorption maximum of [M^{S}FluindBiCl][B(C_6F_5)$_4$)]$_2$ (**2Bi**) in CH_2Cl_2. Molar absorptivity in the presented range ($\varepsilon_{430\ nm}$ = 5450 cm^{-1} M) was determined using the Lambert–Beer law.

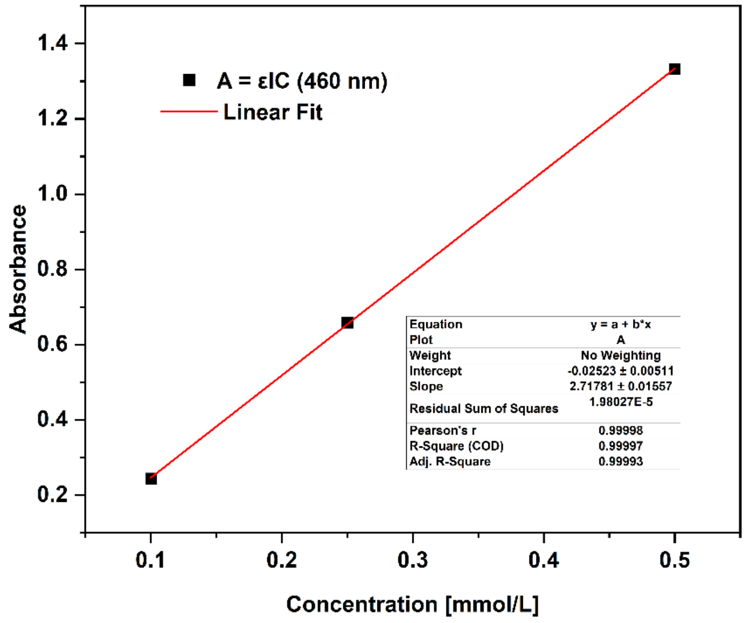

Figure S86. Concentration–absorbance dependence at 460 nm, absorption maximum of [M^{S}FluindBi$_3$][B(C_6F_5)$_4$)]$_2$ (**7**) in CH_2Cl_2. Molar absorptivity in the presented range ($\varepsilon_{460\ nm}$ = 2718 cm^{-1} M) was determined using the Lambert–Beer law.

X-ray crystallography

Intensity data of **2As**, **2Sb**·3 CH_2Cl_2, **2Bi**·2 CH_2Cl_2, **3**, **4**, **5In**, **6S**, **6Se**, **6Te** and **7**·0.5 1,2-$Cl_2C_6H_4$ were collected at 100 K on a Bruker Venture D8 diffractometer with graphite-monochromated Mo-Kα (0.7107 Å) radiation. All structures were solved by direct methods and refined based on F^2 by use of the SHELX program package as implemented in OLEX 2 version 1.5.[S4] All non-hydrogen atoms were refined using anisotropic displacement parameters. Hydrogen atoms attached to carbon atoms were included in geometrically calculated positions using a riding model. Crystal and refinement data are collected in Table S2. Figures were created using DIAMOND.[S5] Crystallographic data for the structural analyses have been deposited with the Cambridge Crystallographic Data Centre, nos. 2538654 – 2538663. Copies of this information may be obtained free of charge from The Director, CCDC, 12 Union Road, Cambridge CB2 1EZ, UK (Fax: +44-1223-336033; e-mail: deposit@ccdc.cam.ac.uk or http://www.ccdc.cam.ac.

Table S2 Crystal data and structure refinement of compounds **2As**, **2Sb**·3 CH_2Cl_2.

	**2As**	**2Sb**·3 CH_2Cl_2
Formula	$C_{64}H_{33}AsBClF_{20}$	$C_{67}H_{39}BCl_7F_{20}Sb$
Formula weight, g mol^{-1}	1303.08	6310.7(5)
Crystal system	triclinic	monoclinic
Crystal size, mm	0.35 × 0.15 × 0.10	0.34 × 0.28 × 0.21
Space group	$P\bar{1}$	$P2_1/c$
a, Å	13.3163(16)	14.5024(6)
b, Å	15.084(2)	17.3893(9)
c, Å	15.5045(19)	25.2753(12)
α, °	61.876(4)	90
β, °	77.835(3)	98.0900(10)
γ, °	77.312(4)	90
V, Å^3	2658.6(6)	6310.7(5)
Z	2	4
ρ_{calcd}, Mg m^{-3}	1.628	1.689
μ (Mo $K\alpha$), mm^{-1}	0.804	0.837
F(000)	1304	3184
θ range, deg	2.31 to 25.00	2.00 to 25.00
Index ranges	$-14 \le h \le 15$	$-17 \le h \le 17$
	$-17 \le k \le 17$	$-20 \le k \le 20$
	$-18 \le l \le 18$	$-30 \le l \le 30$
No. of reflns collected	26191	158125
Completeness to θ_{max}	99.7%	99.8%
No. indep. Reflns	9343	11095
No. obsd reflns with ($I>2\sigma(I)$)	8356	10421
No. refined params	788	788
GooF (F^2)	1.070	1.068
R_1 (F) ($I > 2\sigma(I)$)	0.0290	0.0276
wR_2 (F^2) (all data)	0.0770	0.0717
Largest diff peak/hole, e Å^{-3}	0.440 / –0.521	0.430 / –0.479
CCDC number	2538654	2538655

Table S2 (cont) Crystal data and structure refinement of compounds **2Bi**·2 CH_2Cl_2 and **3**.

	**2Bi**·2 CH_2Cl_2	**3**
Formula	$C_{66}H_{37}BBiCl_5F_{20}$	$C_{40}H_{35}As$
Formula weight, g mol^{-1}	1606.99	590.60
Crystal system	monoclinic	triclinic
Crystal size, mm	0.13 × 0.12 × 0.10	0.39 × 0.26 × 0.24
Space group	$P2_1/n$	$P\bar{1}$
a, Å	12.4900(4)	10.7512(5)
b, Å	14.6983(4)	14.3199(7)
c, Å	33.4683(7)	21.1704(10)
α, °	90	70.582(2)
β, °	99.7660(10)	89.500(2)
γ, °	90	79.168(2)
V, Å^3	6055.1(3)	3014.1(3)
Z	4	4
ρ_{calcd}, Mg m^{-3}	1.763	1.302
μ (Mo $K\alpha$), mm^{-1}	3.237	1.155
F(000)	3144	1232
θ range, deg	2.16 to 25.00	2.04 to 25.00
Index ranges	$-14 \le h \le 14$	$-12 \le h \le 12$
	$-17 \le k \le 17$	$-17 \le k \le 17$
	$-39 \le l \le 39$	$-25 \le l \le 25$
No. of reflns collected	125821	233104
Completeness to θ_{max}	99.8%	99.9%
No. indep. Reflns	10648	10595
No. obsd reflns with ($I>2\sigma(I)$)	9741	9751
No. refined params	812	933
GooF (F^2)	1.152	1.104
R_1 (F) ($I > 2\sigma(I)$)	0.0471	0.0421
wR_2 (F^2) (all data)	0.1120	0.1085
Largest diff peak/hole, e Å^{-3}	0.926 / –1.404	0.392 / –0.847
CCDC number	2538656	2538657

Table S2 (cont) Crystal data and structure refinement of compounds **4** and **5In**.

	**4**	**5In**
Formula	$C_{64}H_{34}BF_{20}Sb$	$C_{68.38}H_{43.64}BCl_5F_{20}InSb_{0.94}$
Formula weight, g mol^{-1}	1315.47	1662.56
Crystal system	monoclinic	triclinic
Crystal size, mm	0.23 × 0.21 × 0.19	0.23 × 0.16 × 0.11
Space group	$P2_1/c$	$P\bar{1}$
a, Å	18.9873(7)	11.2318(4)
b, Å	19.5848(7)	16.7160(6)
c, Å	15.0076(6)	18.0752(6)
α, °	90	97.2600(10)
β, °	111.6680(10)	99.9420(10)
γ, °	90	90.8710(10)
V, Å^3	5186.4(3)	3313.5(2)
Z	4	2
ρ_{calcd}, Mg m^{-3}	1.685	1.665
μ (Mo $K\alpha$), mm^{-1}	0.649	1.032
F(000)	2616	1640
θ range, deg	2.38 to 25.00	2.39 to 25.00
Index ranges	$-27 \le h \le 27$	$-13 \le h \le 13$
	$-27 \le k \le 27$	$-19 \le k \le 19$
	$-21 \le l \le 21$	$-21 \le l \le 21$
No. of reflns collected	181379	94035
Completeness to θ_{max}	99.9%	99.8%
No. indep. Reflns	15813	11659
No. obsd reflns with ($I>2\sigma(I)$)	12362	10413
No. refined params	814	835
GooF (F^2)	1.093	1.024
R_1 (F) ($I > 2\sigma(I)$)	0.0538	0.0320
wR_2 (F^2) (all data)	0.1225	0.0793
Largest diff peak/hole, e Å^{-3}	0.595 / –0.569	0.868 / –0.365
CCDC number	2538658	2538659

Table S2 (cont) Crystal data and structure refinement of compounds **6S** and **6Se**.

	**6S**	**6Se**
Formula	$C_{71}H_{40}BCl_2F_{20}SSb$	$C_{71}H_{40}BCl_2F_{20}SbSe$
Formula weight, g mol^{-1}	1508.55	1555.45
Crystal system	triclinic	triclinic
Crystal size, mm	0.25 × 0.21 × 0.19	0.23 × 0.19 × 0.15
Space group	$P\bar{1}$	$P\bar{1}$
a, Å	14.3887(6)	14.3750(7)
b, Å	14.6446(5)	14.7246(7)
c, Å	15.0465(6)	15.0893(6)
α, °	105.4440(10)	105.532(2)
β, °	91.975(2)	91.807(2)
γ, °	92.050(2)	91.986(2)
V, Å^3	3050.9(2)	3072.6(2)
Z	2	2
ρ_{calcd}, Mg m^{-3}	1.642	1.681
μ (Mo $K\alpha$), mm^{-1}	0.681	1.234
F(000)	1504	1540
θ range, deg	2.27 to 25.00	2.26 to 25.00
Index ranges	$-20 \leq h \leq 20$	$-20 \leq h \leq 20$
	$-20 \leq k \leq 20$	$-21 \leq k \leq 21$
	$-21 \leq l \leq 21$	$-21 \leq l \leq 21$
No. of reflns collected	169553	171915
Completeness to θ_{max}	99.6%	99.9%
No. indep. Reflns	18646	18744
No. obsd reflns with ($I>2\sigma(I)$)	17283	16620
No. refined params	879	879
GooF (F^2)	1.051	1.021
R_1 (F) ($I > 2\sigma(I)$)	0.0274	0.0261
wR_2 (F^2) (all data)	0.0658	0.0624
Largest diff peak/hole, e Å^{-3}	0.463 / –0.451	0.506 / –0.434
CCDC number	2538660	2538661

Table S2 (cont) Crystal data and structure refinement of compounds **6Te**·0.5 CH_2Cl_2 and **7**·0.5 1,2-$Cl_2C_6H_4$.

	**6Te**·0.5 CH_2Cl_2	**7**·0.5 1,2-$Cl_2C_6H_4$
Formula	C70.50H39BClF20SbTe	$C_{91}H_{35}B_2Bi_3ClF_{40}$
Formula weight, g mol^{-1}	1561.62	2572.27
Crystal system	monoclinic	triclinic
Crystal size, mm	0.20 × 0.17 × 0.13	0.13 × 0.12 × 0.06
Space group	$P2_1/n$	$P\bar{1}$
a, Å	18.8051(7)	14.5156(5)
b, Å	16.5144(6)	15.4287(5)
c, Å	19.5397(6)	40.2924(15)
α, °	90	82.514(2)
β, °	90.0810(10)	88.653(2)
γ, °	90	69.338(2)
V, $Å^3$	6068.1(4)	8368.9(5)
Z	4	4
ρ_{calcd}, Mg m^{-3}	1.709	2.013
μ (Mo $K\alpha$), mm^{-1}	1.076	6.462
F(000)	3068	4761
θ range, deg	2.17 to 25.00	2.04 to 25.00
Index ranges	$-22 \le h \le 22$	$-19 \le h \le 19$
	$-19 \le k \le 19$	$-20 \le k \le 20$
	$-23 \le l \le 23$	$-54 \le l \le 54$
No. of reflns collected	170759	412165
Completeness to θ_{max}	99.9%	99.9%
No. indep. Reflns	10695	29490
No. obsd reflns with ($I>2\sigma(I)$)	10254	22930
No. refined params	842	1742
GooF (F^2)	1.056	1.030
R_1 (F) ($I > 2\sigma(I)$)	0.0216	0.057
wR_2 (F^2) (all data)	0.0561	0.125
Largest diff peak/hole, e $Å^{-3}$	0.388 / –0.862	2.787 / –2.299
CCDC number	2538662	2538663

Computational Methods

Computational methodology. Geometry Optimizations of **5In**, **6S**, **6Se**, **6Te** and **7** were carried out using density functional theory (DFT) at the B3PW91/6-311+G(2df,p)[S6, S7] level of theory using the Gaussian16[S8] software package. The respective anions have been omitted in the computations. For the In, Sb, Se, Te and Bi atoms the cc-pVTZ-PP[S9] basis set were used with effective core potentials accounting for 10 (Se), 28 (In, Sb, Te) and 60 (Bi) electrons, respectively. Basis sets were obtained from the Basis Set Exchange Library.[S10] Dispersion effects were modelled using Grimme's GD3BJ parameters.[S11] Subsequent frequency analysis confirmed all structures to be local minima on the potential energy surface. For Cornella's allylic $[Aryl_2Bi_3]^+$ (**IV**) a single-point calculation at the same level of theory specified above (incl. empirical dispersion correction) was performed using the reported optimized coordinates.[11] The wavefunction files of the optimized structures were used for a topological analysis of the electron density according to the Atoms-In-Molecules partitioning scheme[17] using AIMAll.[S12] The NCI[18] grids were computed with NCIplot.[S13] NBO/NLMO[21] analysis was performed using the NBO6 software.[S14] Independent gradient model based on Hirshfeld partition (IGMH)[28] analysis and integrated region indicator (IRI)[29] analysis were obtained with Multiwfn_3.8.[S15] For the analysis of the IRI-π and the localized orbital locator of the π-orbitals (LOL-π)[30] an orbital localization analysis was performed according to the Pipek-Mezey method[S16] using Orca 6.0.1[S17] with the B3WP91/def2-TZVP[S18] level of theory and Grimme's D3BJ dispersion.[S11] Then Multiwfn was used to detect the π-orbitals and to set the occupation of all other orbitals, except the π-orbitals, to zero. Figures are displayed using VMD.[S19] Compliance constants and coupling constants[31] were obtained with the COMPLIANCE software (version 3.0.2).[S20] TD-DFT computations have been performed for the first 20 states using the X2C quasi relativistic Hamiltonian,[22, S21] with the X2C-TZVPall basis set in conjunction with the X2C/J auxiliary basis set[S22] and Grimme's D3BJ dispersion.[S11] SOC was implemented by the spin-orbit mean-field operator,[23] finite

nucleus settings,[24,25] and picture change corrections.[26] The quasi-degenerate perturbation theory was incorporated within the TD-DFT section.[27]

Table S3. Computed and experimental bond lengths [Å] and angles [°] of $[M^SFluindBi_3]^{2+}$ (**7**) and computed values for $\mathbf{[PhBi_3]^{2+}}$ (Bi-atom directly bonded to a C-atom indicated as Bi_C).

Bond	7	7 (Exp.)	$[PhBi_3]^2$
Bi_C-Bi	2.90686	2.9303(8)/2.9647(4)	2.92097
Bi_C-Bi	2.81055	2.8567(6)/2.8678(7)	2.92097
Bi_C-Bi_C	3.35216	3.4828(5)/3.5137(5)	3.12440
C-Bi_C	2.38980	2.392(12)/2.367(12)	2.38884
C-Bi_C	2.50800	2.682(13)/2.603(8)	2.38879
C_{ipso}-C_{ortho}	1.40554	1.379(17)/1.414(14)	1.42038
C_{ortho}-C_{meta}	1.38627	1.406(16)/1.395(14)	1.37557
C_{meta}-C_{para}	1.39272	1.403(18)/1.375(12)	1.40104
C_{para}-C_{meta}	1.39272	1.398(18)/1.400(14)	1.38968
C_{meta}-C_{ortho}	1.38627	1.380(19)/1.386(14)	1.38395
C_{ortho}-C_{ipso}	1.40554	1.391(19)/1.426(12)	1.42367
Q	0.01286	0.00025/0.01325	0.02893
Bi-$Bi_C$$Bi_C$C*	0.000	0.1378/0.0886	1.952
Bi_C-Bi-Bi_C	71.76833	73.989(18)/74.066(14)	64.66367
Bi_C-C-Bi_C	83.34465	86.5(4)/89.9(4)/	81.68220
C_{ortho}-C_{ipso}-C_{ortho}	117.68116	120.0(11)/117.2(8)	119.53881

* Distance of top Bi-atom from the mean plane spanned by both Bi_C atoms and C_{ipso}. The quinoid character Q is defined as = [(C_{ipso}-C_{ortho}) + (C_{meta}-C_{para}) + (C_{para}-C_{meta}) + (C_{orhto}-C_{ipso})] / 4 – [(C_{ortho}-C_{meta}) + C_{meta}-C_{ortho})] / 2. For a perfect hexagon, Q = 0, whereas for a perfect quinoid structure, Q = 0.138 with C_{ipso}-C_{ortho} = C_{meta}-C_{para} = C_{para}-C_{meta} = C_{orhto}-C_{ipso} = 1.455 Å (sp^3 – sp^3 bonds) and C_{ortho}-C_{meta} = C_{meta}-C_{ortho} = 1.317 Å (sp^2 – sp^2 bonds).

Table S4. Topological bond properties from AIM analysis of **5In** and **6Ch** (Ch = S, Se, Te).

Species	d [Å]	ρ(r) [eÅ^{-3}]	∇^2ρ(r) [eÅ^{-5}]	ε	G/ρ(r) [a.u.]	H/ρ(r) [a.u.]
**5In**						
Sb1-In2	2.895	0.34	0.4	0.07	0.34	–0.27
Sb1-C8	2.153	0.75	1.2	0.16	0.59	–0.48
In2-C22	2.157	0.66	3.3	0.04	0.72	–0.37
In2-C60	2.152	0.67	3.3	0.04	0.72	–0.37
**6S**						
Sb1-S2	2.359	0.66	1.5	0.22	0.60	–0.44
Sb1-C82	2.139	0.78	0.9	0.07	0.58	–0.50
S2-C60	1.773	1.31	–7.4	0.09	0.30	–0.69
**6Se**						
Sb1-Se2	2.473	0.60	0.7	0.25	0.50	–0.41
Sb1-C4	2.142	0.77	0.9	0.08	0.58	–0.49
Se2-C29	1.918	1.06	–3.9	0.08	0.37	–0.63
**6Te**						
Sb1-Te2	2.650	0.53	–0.2	0.31	0.38	–0.40
Sb1-C4	2.147	0.76	1.0	0.09	0.58	–0.49
Te2-C21	2.116	0.83	–0.4	0.08	0.49	–0.53

Table S4 cont. Topological bond properties from AIM analysis of $[M^{S}FluindBi_3]^{2+}$ (**7**), the allylic $[Aryl_2Bi_3]^{+}$ (**IV**) and $[PhBi_3]^{2+}$.

**Species**	**d [Å]**	**ρ(r) [eÅ⁻³]**	**∇²ρ(r) [eÅ⁻⁵]**	**ε**	**G/ρ(r) [a.u.]**	**H/ρ(r) [a.u.]**
**7**						
**core region**						
Bi74-Bi76	2.907	0.37	0.7	0.06	0.40	–0.27
Bi75-Bi76	2.812	0.43	0.9	0.09	0.46	–0.30
Bi74-C1	2.390	0.52	1.5	0.16	0.53	–0.32
Bi75-C1	2.509	0.38	1.6	0.19	0.53	–0.23
**Secondary interactions**						
Bi74-C18	3.099	0.14	1.0	2.66	0.49	–0.01
Bi74-C46	3.100	0.14	1.0	2.66	0.49	–0.01
Bi75-C28	3.078	0.14	0.9	0.81	0.46	–0.01
Bi75-C56	3.078	0.14	0.9	0.81	0.46	–0.01
Bi76-C25	3.690	0.06	0.4	1.12	0.40	0.05
Bi76-C53	3.690	0.06	0.4	1.12	0.40	0.05
**IV**						
**Core region**						
Bi2-Bi1	2.912	0.38	0.6	0.14	0.37	–0.27
Bi3-Bi1	2.913	0.38	0.6	0.14	0.37	–0.27
Bi2-C4	2.295	0.63	1.5	0.08	0.55	–0.38
Bi3-C125	2.296	0.63	1.5	0.08	0.55	–0.38
**Secondary Interactions**						
Bi2-C98	3.174	0.12	0.8	0.47	0.01	0.11
Bi2-C54	3.481	0.06	0.5	0.47	0.07	0.06
Bi2-C168	3.926	0.04	0.2	0.37	0.08	0.04
Bi2-H118	3.375	0.03	0.3	0.49	0.09	0.03
Bi1-C89	3.411	0.08	0.6	0.49	0.05	0.06
Bi3-C91	3.870	0.04	0.3	0.37	0.08	0.04
Bi3-C175	3.183	0.12	0.8	1.91	0.47	0.01
Bi-C166	3.382	0.08	0.6	2.18	0.49	0.05
Bi3-H192	3.385	0.03	0.3	0.17	0.49	0.09
Bi3-C219	3.497	0.06	0.5	0.44	0.47	0.07
**$[PhBi_3]^{2+}$**						
Bi8-Bi10	2.924	0.38	0.6	0.17	0.38	–0.27
Bi9-Bi10	2.924	0.38	0.6	0.17	0.38	–0.27
Bi8-C1	2.390	0.49	1.8	0.21	0.56	–0.31
Bi9-C1	2.390	0.49	1.8	0.21	0.56	–0.31

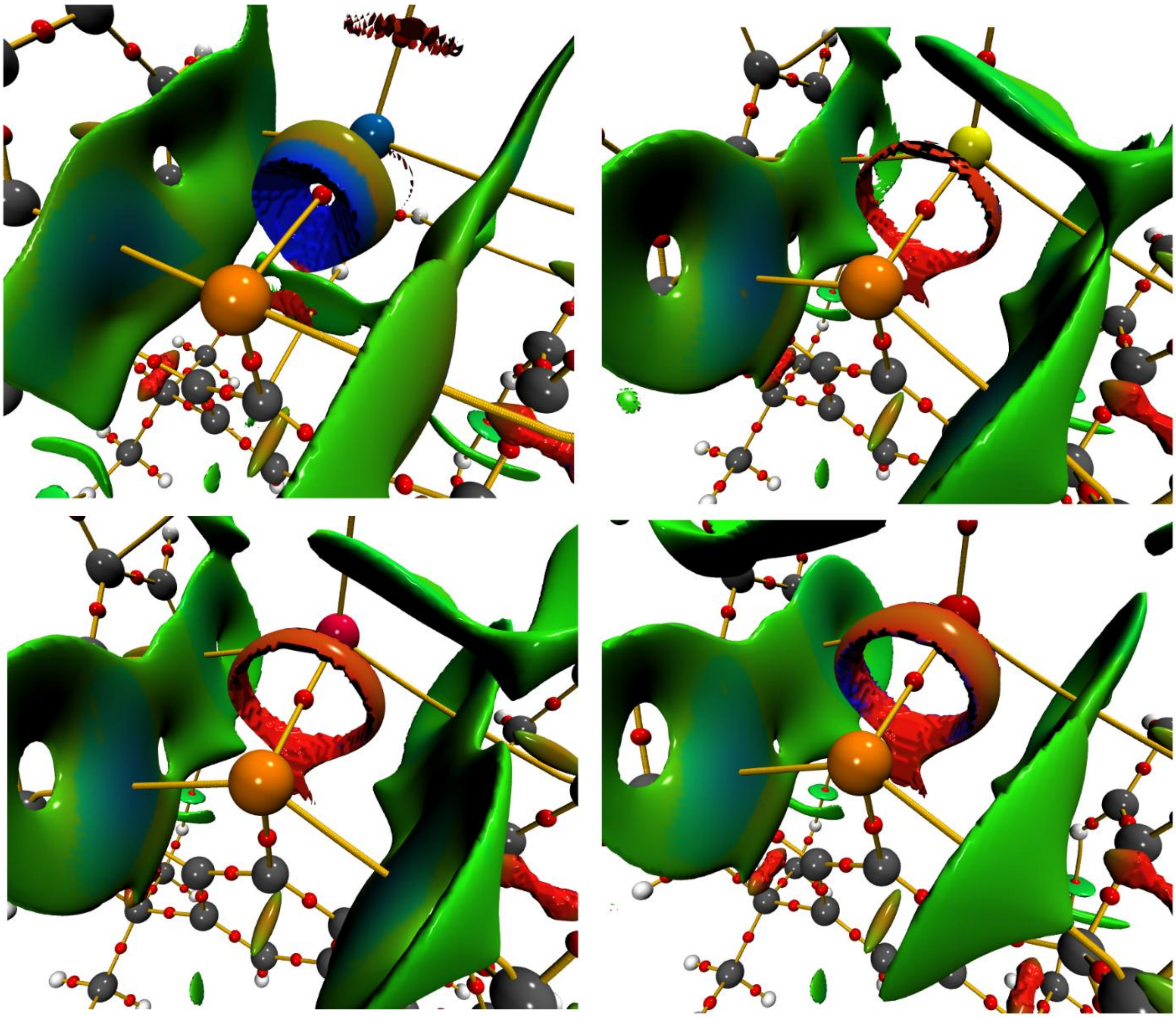

Figure S87: AIM molecular graphs of **5In** (top left), **6S** (top right), **6Se** (bottom left) and **6Te** (bottom right) with bond critical points as red spheres and bond paths in orange as well as NCI *iso*-surfaces at s(r) = 0.5 colour coded with sign(λ2)ρ in a. u. Blue surfaces refer to attractive forces and red to repulsive forces. Green indicates weak interactions.

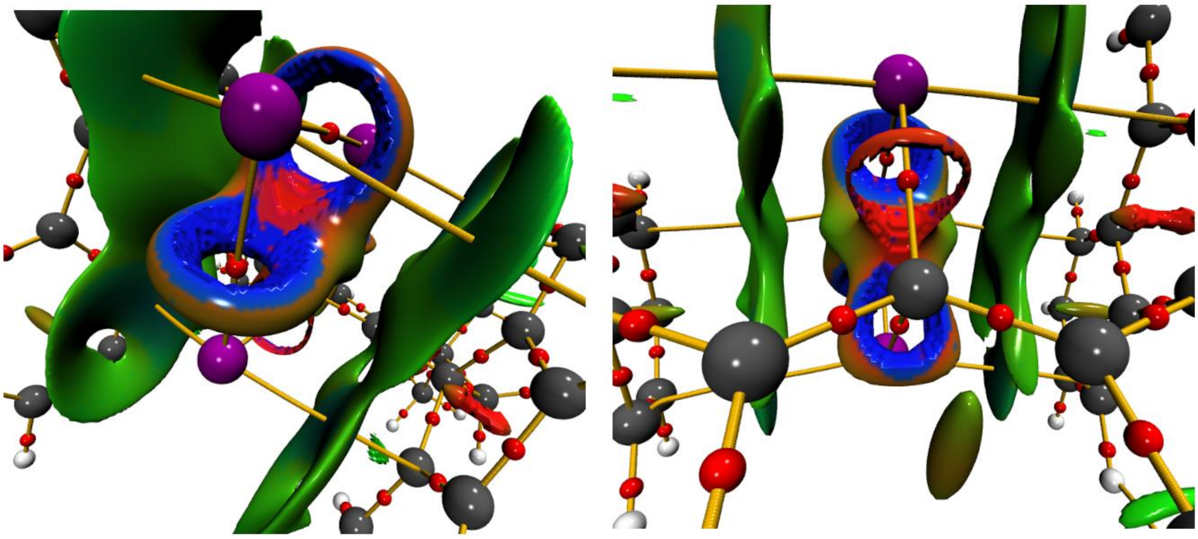

Figure S88. AIM molecular graphs of $[\mathrm{M^{S}FluindBi_3}]^{2+}$ (**7**) with bond critical points as red spheres and bond paths in orange as well as NCI *iso*-surfaces at s(r) = 0.5 colour coded with sign(λ2)ρ in a. u. Blue surfaces refer to attractive forces and red to repulsive forces. Green indicates weak interactions. Left: view from the top Bi-atom; right: view from the *ipso*-C-atom.

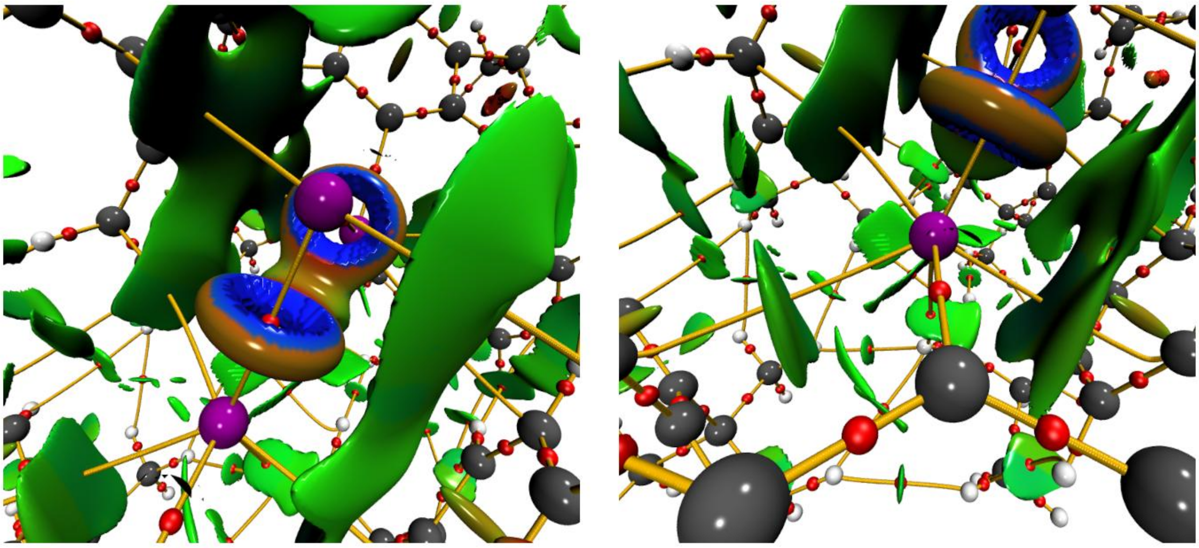

Figure S89. AIM molecular graphs of the allylic $[\mathrm{Aryl_2Bi_3}]^{+}$ (**IV**) with bond critical points as red spheres and bond paths in orange as well as NCI *iso*-surfaces at s(r) = 0.5 colour coded with sign(λ2)ρ in a. u. Blue surfaces refer to attractive forces and red to repulsive forces. Green indicates weak interactions. Left: view from the top Bi-atom; right: view from the *ipso*-C-atom.

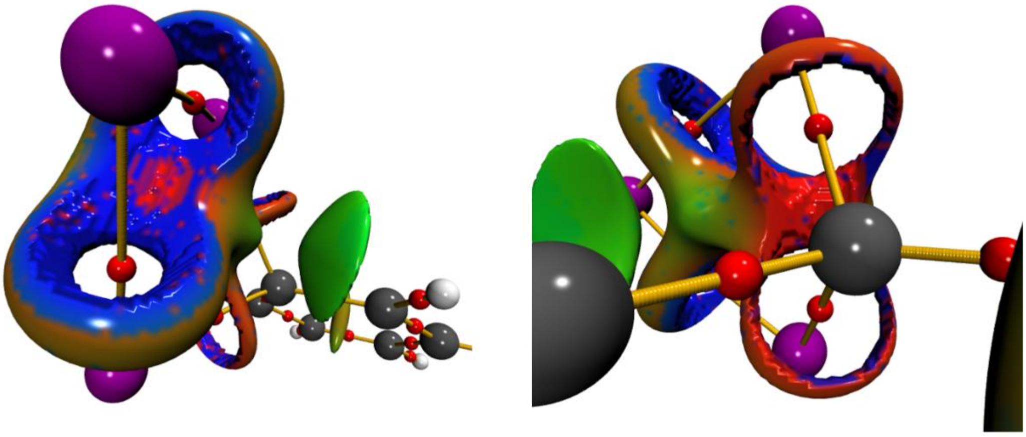

Figure S90. AIM molecular graphs of **$[PhBi_3]^{2+}$** with bond critical points as red spheres and bond paths in orange as well as NCI *iso*-surfaces at s(r) = 0.5 colour coded with sign(λ2)ρ in a. u. Blue surfaces refer to attractive forces and red to repulsive forces. Green indicates weak interactions. Left: view from the top Bi-atom; right: view from the *ipso*-C-atom.

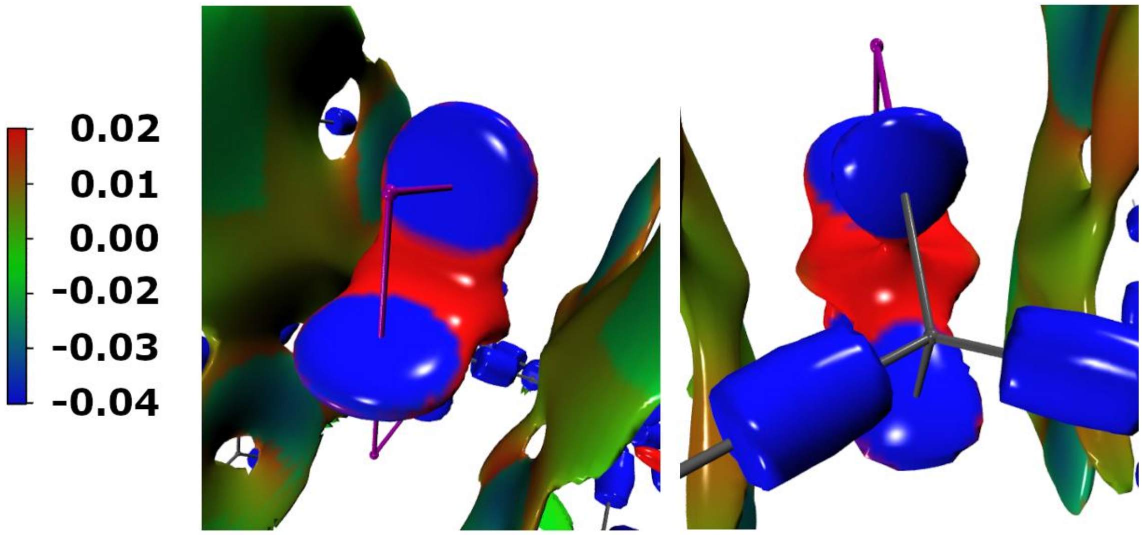

Figure S91 IRI *iso*-surfaces of [M^{S}FluindBi$_3$]$^{2+}$ (**7**) at s(r) = 1.0 colour coded with sign(λ2)ρ in a. u. Blue surfaces refer to attractive forces and red to repulsive forces. Green indicates weak interactions. Left: view from the top Bi-atom; right: view from the *ipso*-C-atom.

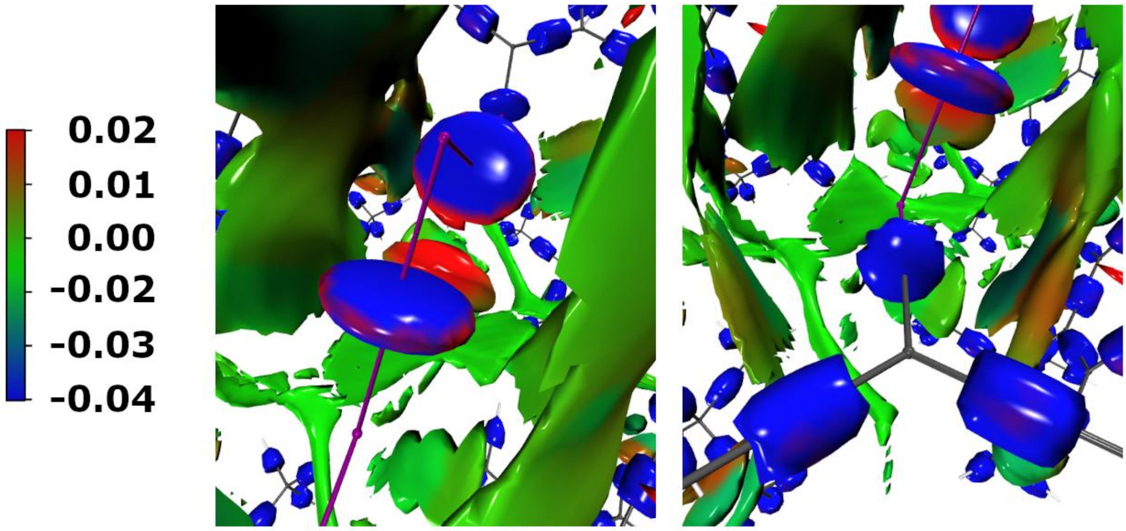

Figure S92. IRI *iso*-surfaces of the allylic [Aryl$_2$Bi$_3$]$^+$ (**IV**) at s(r) = 1.0 colour coded with sign(λ2)ρ in a. u. Blue surfaces refer to attractive forces and red to repulsive forces. Green indicates weak interactions. Left: view from the top Bi-atom; right: view from the *ipso*-C-atom.

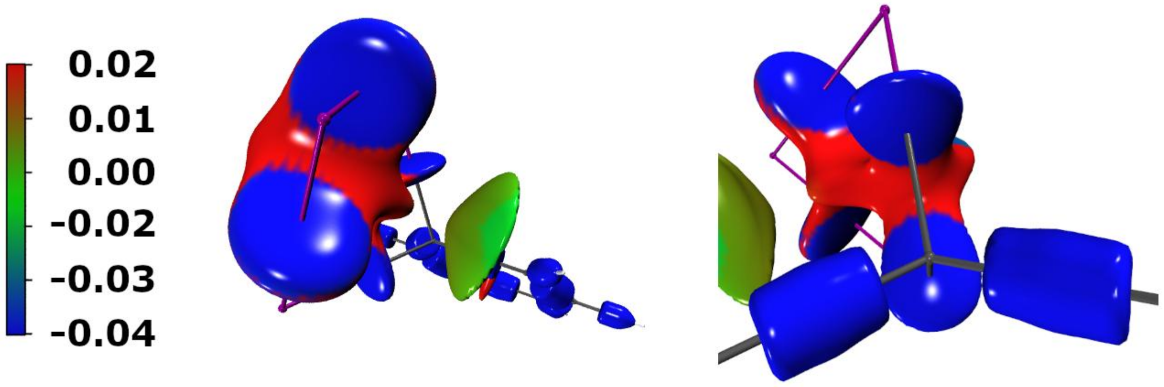

Figure S93. IRI *iso*-surfaces of **$[PhBi_3]^{2+}$** at s(r) = 1.0 colour coded with sign(λ2)ρ in a. u. Blue surfaces refer to attractive forces and red to repulsive forces. Green indicates weak interactions. Left: view from the top Bi-atom; right: view from the *ipso*-C-atom.

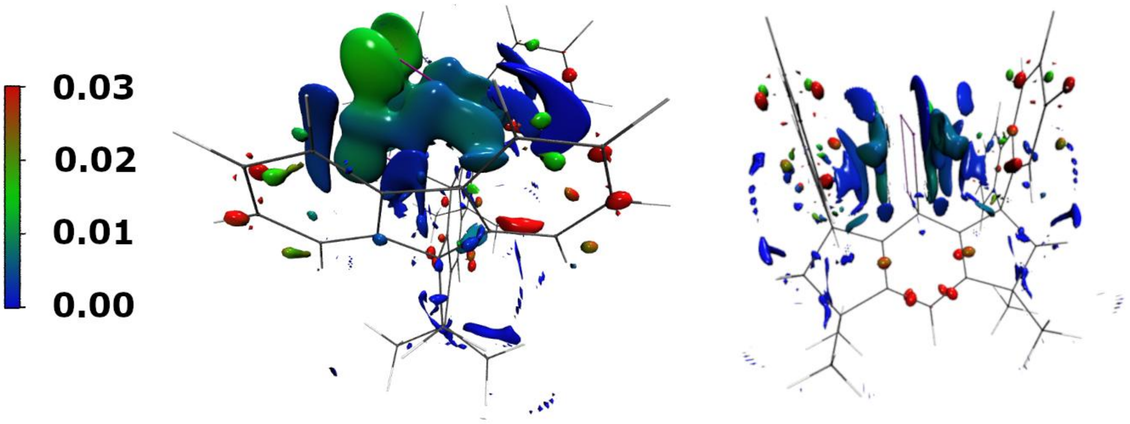

Figure S94. IRI-π *iso*-surfaces of $[M^{S}FluindBi_3]^{2+}$ (**7**) at s(r) = 0.5 with electron density mapped on the *iso*-surface in a. u.

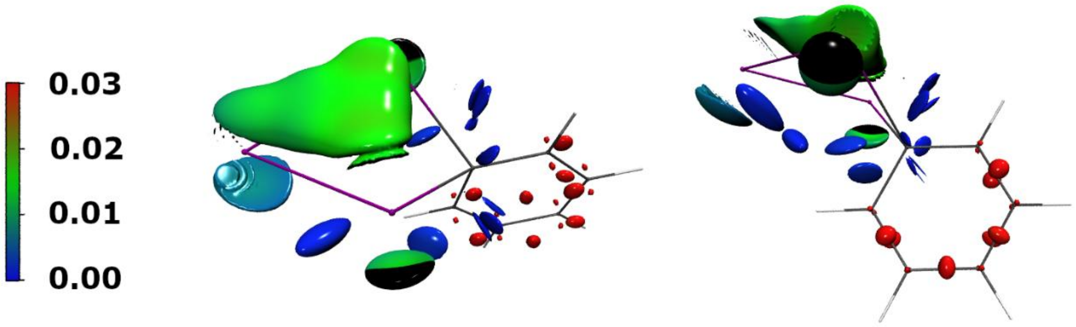

Figure S95. IRI-π *iso*-surfaces of **$[PhBi_3]^{2+}$** at s(r) = 0.5 (left), 1.0 (middle) and 1.7 (right) with the electron density mapped on the *iso*-surface in a. u.

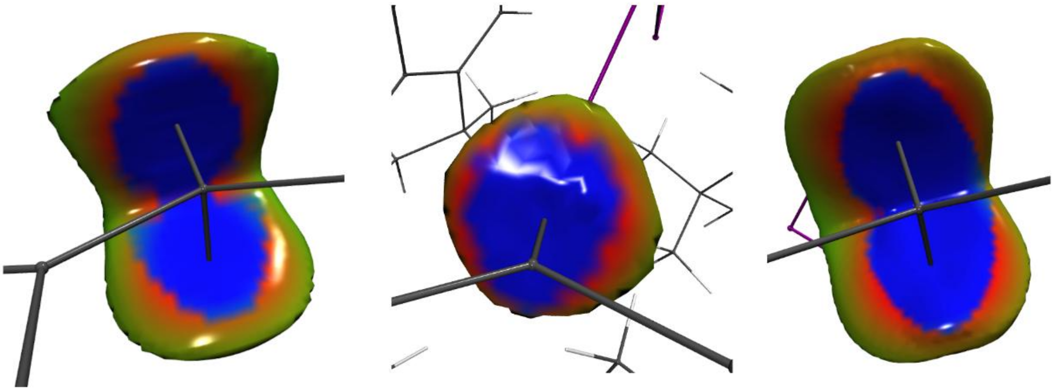

Figure S96. IGM based on a Hirshfeld partition of the molecular density of $[M^{S}FluindBi_3]^{2+}$ (**7**) (left), the allylic $[Aryl_2Bi_3]^+$ (**IV**) (middle) and $\mathbf{[PhBi_3]^{2+}}$ (right). For **7** and $\mathbf{[PhBi_3]^{2+}}$ three fragments are defined: the *ipso*-C-atom and the two Bi-atoms attached to it. For **IV** two fragments are defined: The *ipso*-C-atom and the Bi-atom attached to it. View in each case from the *ipso*-C-atom. IGMH iso-surfaces at s(r) = 0.010 colour coded with sign(λ2)ρ in a. u. Blue surfaces refer to attractive forces and red to repulsive forces. Green indicates weak interactions

Table S5. Topological and integrated bond properties from ELI-D analysis of $[M^{S}FluindBi_3]^{2+}$ (**7**), $[Aryl_2Bi_3]^{+}$ (**IV**) and $[PhBi_3]^{2+}$.

7			IV			$[PhBi_3]^{2+}$		
**Basin**	**N_{ELI} [e]**	**V_{ELI} [Å^3]**	**Basin**	**N_{ELI} [e]**	**V_{ELI} [Å^3]**	**Basin**	**N_{ELI} [e]**	**V_{ELI} [Å^3]**
**Bi74-Bi76**	0.98	5.6	**Bi2-Bi1**	0.89	8.3	**Bi8-Bi10**	1.91	33.7
**Bi74-Bi76**	0.98	5.6	**Bi2-Bi1**	1.51	8.3			
∑	1.97	11.2		2.40	16.7			
**Bi75-Bi76**	1.26	7.7	**Bi3-Bi1**	0.89	8.2	**Bi9-Bi10**	1.91	33.7
**Bi75-Bi76**	1.26	7.8	**Bi3-Bi1**	1.50	8.3			
∑	2.52	15.5		2.40	16.6			
**Bi74-C1**	2.26	7.2	**Bi2-C4***	2.04	8.5	**Bi8-C1**	0.06	0.6
						**Bi8-C1**	0.36	1.2
						**Bi8-C1**	0.97	2.9
						**Bi9-C1**	0.97	2.9
∑	2.26	7.2		2.04	8.5		2.36	7.6

*the occupation of only one disynaptic Bi-C ELI-D basin was computed.

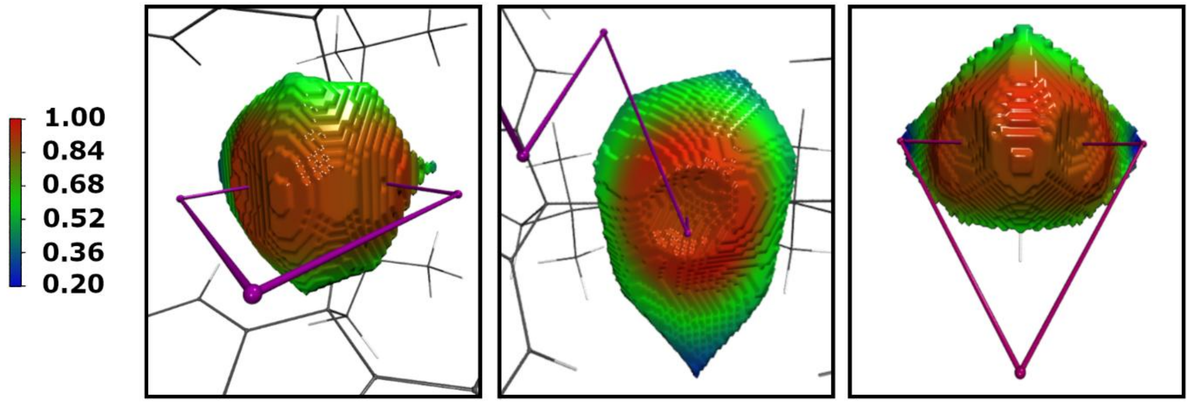

Figure S97. ELI-D distribution (unitless) color-coded onto the ELI-D basin connecting the Bi and the C-atoms of $[M^{S}FluindBi_3]^{2+}$ (**7**) (left), the allylic $[Aryl_2Bi_3]^{+}$ (**IV**) (middle) and $[PhBi_3]^{2+}$ (right), respectively. **IV** shows spherical localizability along the Bi–C bond, whereas for **7** and $[PhBi_3]^{2+}$ the electron localizability is distributed towards both Bi-atoms indicative of electron sharing.

Table S6. Selected delocalization indices (δ), Wiberg bond indices (WBI) and NLMO/NPA bond orders as well as selected AIM and NPA derived charges of **5In** and **6ch** (E = S, Se, Te).

**Species**	**δ**	**WBI**	**NLMO/ NPA**					
	**5In**			**q**	**Sb1**	**In2**	**C8**	**C22/C60**
Sb1-In2	0.64	0.64	0.52	q_{AIM}	0.65	1.04	–0.40	–0.44
Sb1-C8	0.93	0.80	0.57	q_{NPA}	0.79	1.30	–0.44	–1.16
In2-C22	0.79	0.62	0.48					
In2-C60	0.79	0.63	0.49					
	**6S**			**q**	**Sb1**	**S2**	**C82**	**C60**
Sb1-S2	1.11	1.09	0.76	q_{AIM}	1.21	–0.25	–0.42	–0.15
Sb1-C82	0.85	0.74	0.53	q_{NPA}	1.33	–0.06	–0.49	–0.22
S2-C60	1.14	1.04	0.95					
	**6Se**			**q**	**Sb1**	**Se2**	**C4**	**C29**
Sb1-Se2	1.19	1.22	0.90	q_{AIM}	1.10	–0.03	–0.42	–0.23
Sb1-C4	0.86	0.74	0.54	q_{NPA}	1.21	0.12	–0.48	–0.25
Se2-C29	1.10	1.00	0.88					
	**6Te**			**q**	**Sb1**	**Te2**	**C4**	**C21**
Sb1-Te2	1.29	1.38	1.11	q_{AIM}	0.94	0.29	–0.41	–0.34
Sb1-C4	0.88	0.76	0.55	q_{NPA}	1.02	0.42	–0.46	–0.33
Te2-C21	1.04	0.93	0.76					

Table S6 cont. Selected delocalization indices (δ), Wiberg bond indices (WBI) and NLMO/NPA bond orders as well as selected AIM and NPA derived charges of $[M^SFluindBi_3]^{2+}$ (**7**), the allylic $[M^SFluind^{tBu}{}_2Bi_3]^+$ (**IV**) and $[PhBi_3]^{2+}$.

**Species**	**δ**	**WBI**	**NLMO/ NPA**					
		**7**		**q**	**Bi76**	**Bi74**	**Bi75**	**C1**
Bi74-Bi76	1.15	1.21	1.37	q_{AIM}	0.11	0.82	0.71	–0.46
Bi75-Bi76	1.38	1.43	1.58	q_{NPA}	0.09	0.94	0.82	–0.57
Bi74-Bi75	0.37	0.36	0.70					
Bi74-C1	0.68	0.53	0.41					
Bi75-C1	0.50	0.37	0.28					
		**IV**		**q**	**Bi1**	**Bi2**	**Bi3**	**C4**[a)]
Bi1-Bi2	1.26	1.34	1.49	q_{AIM}	0.22	0.58	0.58	–0.33
Bi1-Bi3	1.26	1.34	1.49	q_{NPA}	0.16	0.68	0.67	–0.37
Bi2-Bi3	0.29	0.27	0.54	q_{NPA}[b)]	0.15	0.7	0.7	–0.34
Bi2-C4	0.88	0.79	0.58					
Bi3-C125	0.88	0.79	0.58					
		$[PhBi_3]^{2+}$		**q**	**Bi10**	**Bi8**	**Bi9**	**C1**
Bi8-Bi10	1.38	1.35	1.52	q_{AIM}	0.49	0.71	0.71	–0.50
Bi9-Bi10	1.38	1.35	1.52	q_{NPA}	0.50	0.82	0.82	–0.63
Bi8-Bi9	0.67	0.59	0.86					
Bi8-C1	0.71	0.56	0.39					
Bi9-C1	0.71	0.56	0.39					

a) for **IV**, both C-atom bear the same charge; b) q_{NPA} charges reported by Cornella *et al.*[11]

Table S7. NBO analysis of selected bonds of **5In** and **6Ch** (Ch = S, Se, Te).

	5In	6S		6Se		6Te	
**Orbital**	**Sb1**	**Sb1**	**S2**	**Sb1**	**Se2**	**Sb1**	**Te2**
**LP(1)**	1.95	1.98	1.97	1.98	1.98	1.98	1.98
**LP(2)**			1.69		1.65		1.58

Orbital	Occ.	Atom 1	Hybrid	Atom 2	Hybrid
**5In**					
BD(1) Sb1–In2	1.83	Sb1 (73.5 %)	$sp^{10.79}d^{0.09}$	In2 (26.5 %)	$sp^{3.24}d^{0.03}$
BD(1) Sb1–C8	1.95	Sb1 (28.2 %)	$sp^{11.72}d^{0.03}$	C4 (71.8 %)	$sp^{2.82}d^{0.00}$
BD(1) In2–C22	1.89	In2 (22.0%)	$sp^{1.60}d^{0.01}$	C22 (78.0 %)	$sp^{3.47}d^{0.00}$
BD(1) In2–C60	1.90	In2 (22.5%)	$sp^{1.59}d^{0.01}$	C60 (77.5 %)	$sp^{3.63}d^{0.01}$
**6S**					
BD(1) Sb1–S2	1.95	Sb1 (27.1 %)	$sp^{20.11}d^{0.06}$	S2 (73.0 %)	$sp^{5.13}d^{0.06}$
BD(1) Sb1–C82	1.95	Sb1 (26.7 %)	$sp^{11.51}d^{0.04}$	C82 (73.3 %)	$sp^{2.99}d^{0.01}$
BD(1) S2–C60	1.98	S2 (46.5 %)	$sp^{4.75}d^{0.07}$	C60 (53.5 %)	$sp^{2.92}d^{0.01}$
**6Se**					
BD(1) Sb1–Se2	1.95	Sb1 (31.9 %)	$sp^{19.03}d^{0.07}$	Se2 (68.1 %)	$sp^{6.91}d^{0.08}$
BD(1) Sb1–C4	1.95	Sb1 (27.0 %)	$sp^{11.56}d^{0.04}$	C4 (73.0 %)	$sp^{2.96}d^{0.01}$
BD(1) Se2–C29	1.97	Se2 (43.5 %)	$sp^{7.34}d^{0.06}$	C29 (56.5 %)	$sp^{3.15}d^{0.01}$
**6Te**					
BD(1) Sb1–Te2	1.95	Sb1 (38.6 %)	$sp^{18.48}d^{0.09}$	Te2 (61.4 %)	$sp^{7.96}d^{0.08}$
BD(1) Sb1–C4	1.95	Sb1 (27.6 %)	$sp^{11.49}d^{0.04}$	C4 (72.5 %)	$sp^{2.96}d^{0.01}$
BD(1) Te2–C21	1.97	Te2 (38.6 %)	$sp^{9.93}d^{0.05}$	C21 (61.4 %)	$sp^{3.28}d^{0.01}$

Donor NBO	Acceptor NBO	E2 [kcal/mol]	Donor NBO	Acceptor NBO	E2 [kcal/mol]
**5In**			**6Se**		
BD(1) In2-C22	BD*(1) Sb1-In2	12.1	LP(2) Se2	LV(1) Sb1	49.2
BD(1) In2-C22	BD*(1) In2-C60	22.7	LP(2) Se2	BD*(2) C29-C57	7.0
BD(1) In2-C60	BD*(1) Sb1-In2	11.3	**6Te**		
BD(1) In2-C60	BD*(1) In2-C22	19.2	LP(2) Te2	LV(1) Sb1	59.4
**6S**			LP(2) Te2	BD*(2) C21-C52	4.3
LP(2) S2	LV(1) Sb1	44.8			
LP(2) S2	BD*(2) C4-C60	11.0			

Table S7 cont. NBO analysis of selected bonds of $[M^SFluindBi_3]^{2+}$ (**7**), the allylic $[M^SFluind^{tBu}{}_2Bi_3]^+$ (**IV**) and $[PhBi_3]^{2+}$ with respective hybrids.

Model	Occ.	Model	Occ.	Model	Occ.
**7**		**IV**		**$[PhBi_3]^{2+}$**	
**LP(1) Bi74**	1.97	**LP(1) Bi1**	1.99	**LP(1) Bi8**	1.99
**LP(1) Bi75**	1.98	**LP(1) Bi2**	1.98	**LP(1) Bi9**	1.99
**LP(1) Bi76**	1.97	**LP(1) Bi3**	1.98	**LP(2) Bi10**	1.99
**LV(1) Bi74**	0.62	**LV1 Bi2**	0.69		
**LV(2) Bi74**	0.49				
**LV(1) Bi75**	0.44				
**LP(1) C1**	1.43				

Model	Occ.	Atom 1	Hybrid	Atom 2	Hybrid
		**7**			
**BD(1) Bi74-Bi76**	1.82	Bi74 (49.74 %)	$sp^{34.41}d^{0.22}$	Bi76 (50.26 %)	$sp^{28.95}d^{0.50}$
**BD(1) Bi75-Bi76**	1.93	Bi75 (50.97 %)	$sp^{21.95}d^{0.12}$	Bi76 (49.03 %)	$sp^{22.07}d^{0.31}$
**BD(2) Bi75-Bi76**	1.56	Bi75 (39.59 %)	$sp^{1.00}d^{0.01}$	Bi76 (60.41 %)	$sp^{1.00}d^{0.01}$
		**IV**			
**BD(1) Bi1-Bi2**	1.94	Bi1 (48.71 %)	$sp^{21.20}d^{0.16}$	Bi2 (51.29 %)	$sp^{16.89}d^{0.07}$
**BD(1) Bi1-Bi3**	1.94	Bi1 (48.67 %)	$sp^{21.19}d^{0.16}$	Bi3 (51.33 %)	$sp^{16.89}d^{0.07}$
**BD(2) Bi1-Bi3**	1.45	Bi1 (58.33 %)	$sp^{1.00}d^{0.00}$	Bi3 (41.67 %)	$sp^{99.99}d^{9.03}$
**BD(1) Bi2-C4**	1.95	Bi2 (29.63 %)	$sp^{19.69}d^{0.04}$	C4 (70.37 %)	$sp^{3.04}d^{0.00}$
**BD(1) Bi3-C125**	1.95	Bi3 (29.65 %)	$sp^{19.79}d^{0.04}$	C125 (70.35 %)	$sp^{3.04}d^{0.00}$
		**$[PhBi_3]^{2+}$**			
**BD(1) C1-Bi8**	1.67	C1 (76.37 %)	$sp^{6.81}d^{0.01}$	Bi8 (23.63 %)	$sp^{38.99}d^{0.15}$
**BD(1) C1-Bi9**	1.67	C1 (76.38 %)	$sp^{6.81}d^{0.01}$	Bi9 (23.62 %)	$sp^{38.99}d^{0.15}$
**BD(1) Bi8-Bi10**	1.95	Bi8 (50.56 %)	$sp^{34.38}d^{0.31}$	Bi10 (49.44 %)	$sp^{40.81}d^{0.41}$
**BD(1) Bi9-Bi10**	1.95	Bi9 (50.56 %)	$sp^{34.38}d^{0.31}$	Bi10 (49.44 %)	$sp^{40.81}d^{0.41}$
**3C(1) Bi8-Bi9-Bi10**	1.98	Bi8/Bi9 (both 36.56 %)	both $sp^{99.99}d^{2.90}$	Bi10 (26.88 %)	$sp^{99.99}d^{6.72}$

Donor NBO	Acceptor NBO	E2 [kcal/mol]	Donor NBO	Acceptor NBO	E2 [kcal/mol]
**7**			**IV**		
LP(1) C1	LV(2) Bi74	177.38			
LP(1) C1	LV(1) Bi75	44.32			
BD(2) C1-C2	LV(1) Bi75	42.22			
BD(2) Bi75-Bi76	LV(1) Bi74	53.53	BD(2) Bi1-Bi3	LV(1) Bi2	48.19

Table S8. Natural Localized Molecular Orbital (NLMO) Analysis of **6Ch** (E = S, Se, Te).

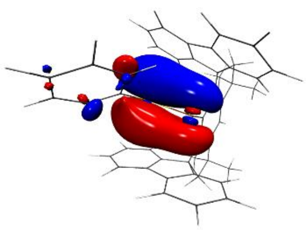
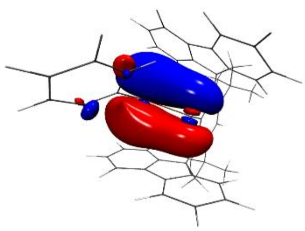
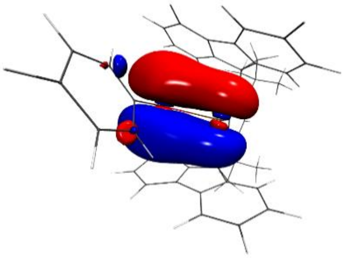

**NLMO 63**	**NLMO 67**	**NLMO 67**
82.51 % of LP(2) S	80.57% % of LP(2) Se	76.59 % of LP(2) Te
**Atomic Hybrid Contribution (with contributions > 1.00 %)**		
14.3 % Sb1	16.72 % Sb1	21.11 % Sb1
82.52 % S2	80.58 % Se2	76.60 % Te2
1.18 % C60		
**NLMO/NPA Bonder (% of total NLMO/NPA Bond order)**		
0.2854 (37.3 %)	0.3343 (37.0 %)	0.4222 (38.0 %)

Table S8 cont. Natural Localized Molecular Orbital (NLMO) Analysis of $[M^{S}FluindBi_3]^{2+}$ (**7**).

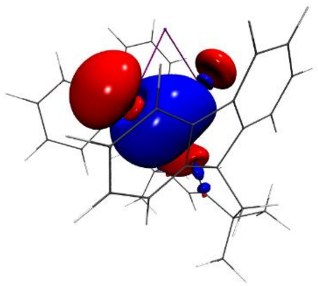
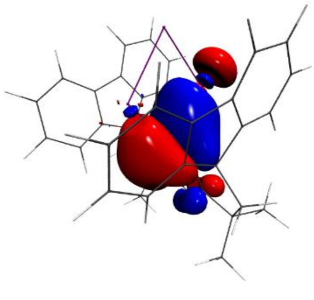
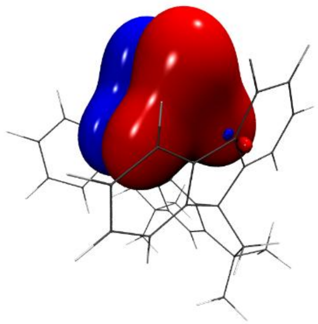

**NLMO 68**	**NLMO 73**	**NLMO 170**
68.86 % of LP(1) C1	81.55 % of BD(2) C1-C2	75.47% of BD(2) Bi75-Bi76
**Atomic Hybrid Contribution (with contributions > 1.00 %)**		
69.21 % C1	48.65 % C1	22.44 % Bi74
19.96 % Bi74	33.222 % C2	29.02 % Bi75
8.51 % Bi75	4.39 % C6	46.47 % Bi76
	1.07 % C7	
	1.54 % C8	
	4.15 % C17	
	5.48 % Bi75	

Table S8 cont. Natural Localized Molecular Orbital (NLMO) Analysis of the allylic $[M^{S}Fluind^{tBu}{}_{2}Bi_{3}]^{+}$ (**IV**).

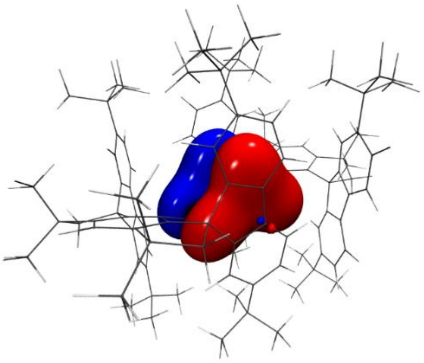

NLMO 145
69.92 % of BD(2) Bi1-Bi3
**Atomic Hybrid Contribution (with contributions > 1.00 %)**
41.99 % Bi1
27.61 % Bi2
27.96 % Bi3

Table S8 cont. Natural Localized Molecular Orbital (NLMO) Analysis of $[\mathbf{PhBi_3}]^{2+}$.

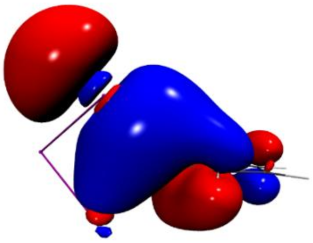

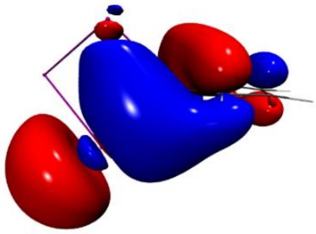

NLMO 39	NLMO 40
82.71 % of BD(1) C1-Bi8	82.70 % of BD(1) C1-Bi9
**Atomic Hybrid Contribution (with contributions > 1.00 %)**	
63.63 % C1	63.63 % C1
5.28 % C2	5.28 % C2
2.62 % C4	2.62 % C4
5.44 % C7	5.45 % C7
19.33 % Bi8	2.19 % Bi8
2.19 % Bi9	19.33 % Bi9

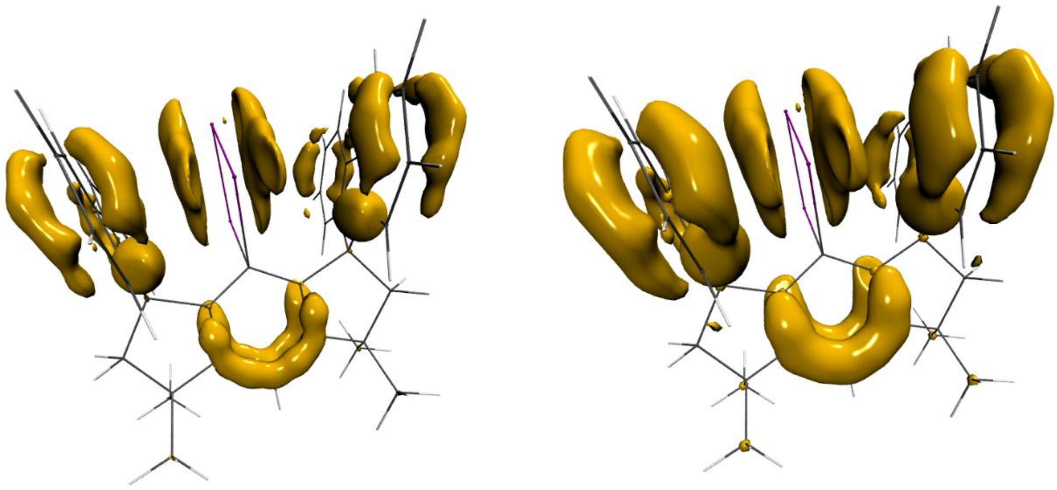

Figure S98. LOL-π of [M^{S}FluindBi$_3$]$^{2+}$ (**7**) at *iso*-surfaces of 0.5 (left) and 0.35 (right).

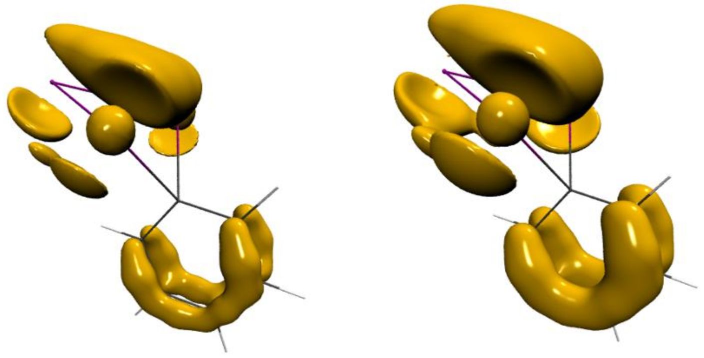

Figure S99. LOL-π of **[PhBi$_3$]$^{2+}$** at *iso*-surfaces of 0.5 (left) and 0.35 (right) from above and below the central phenyl ring.

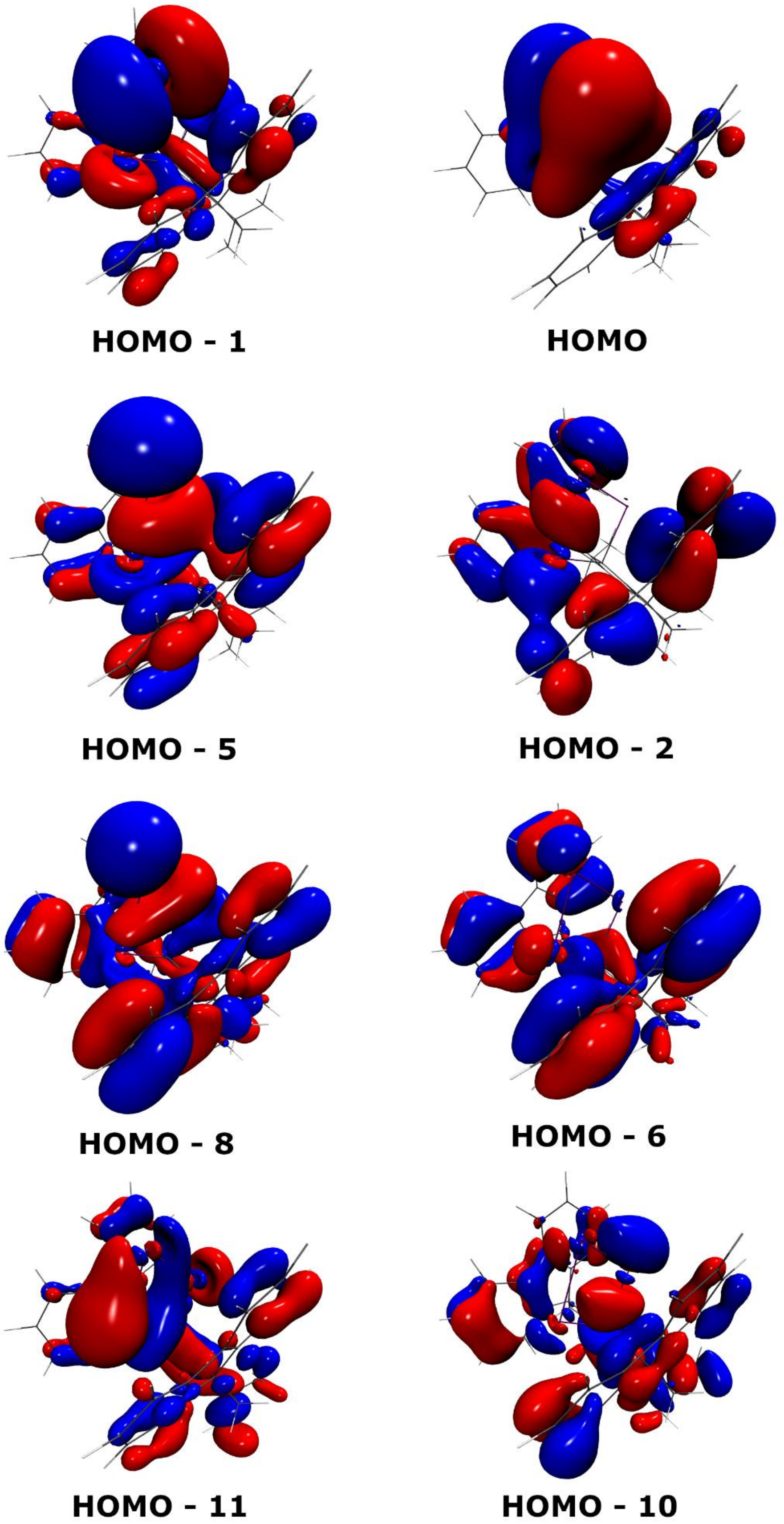

Figure S100. Selected occupied molecular orbitals of $[M^SFluindBi_3]^{2+}$ (**7**) at *iso*-surfaces of +/– 0.02 (blue/red) representing the CBi_3 core.

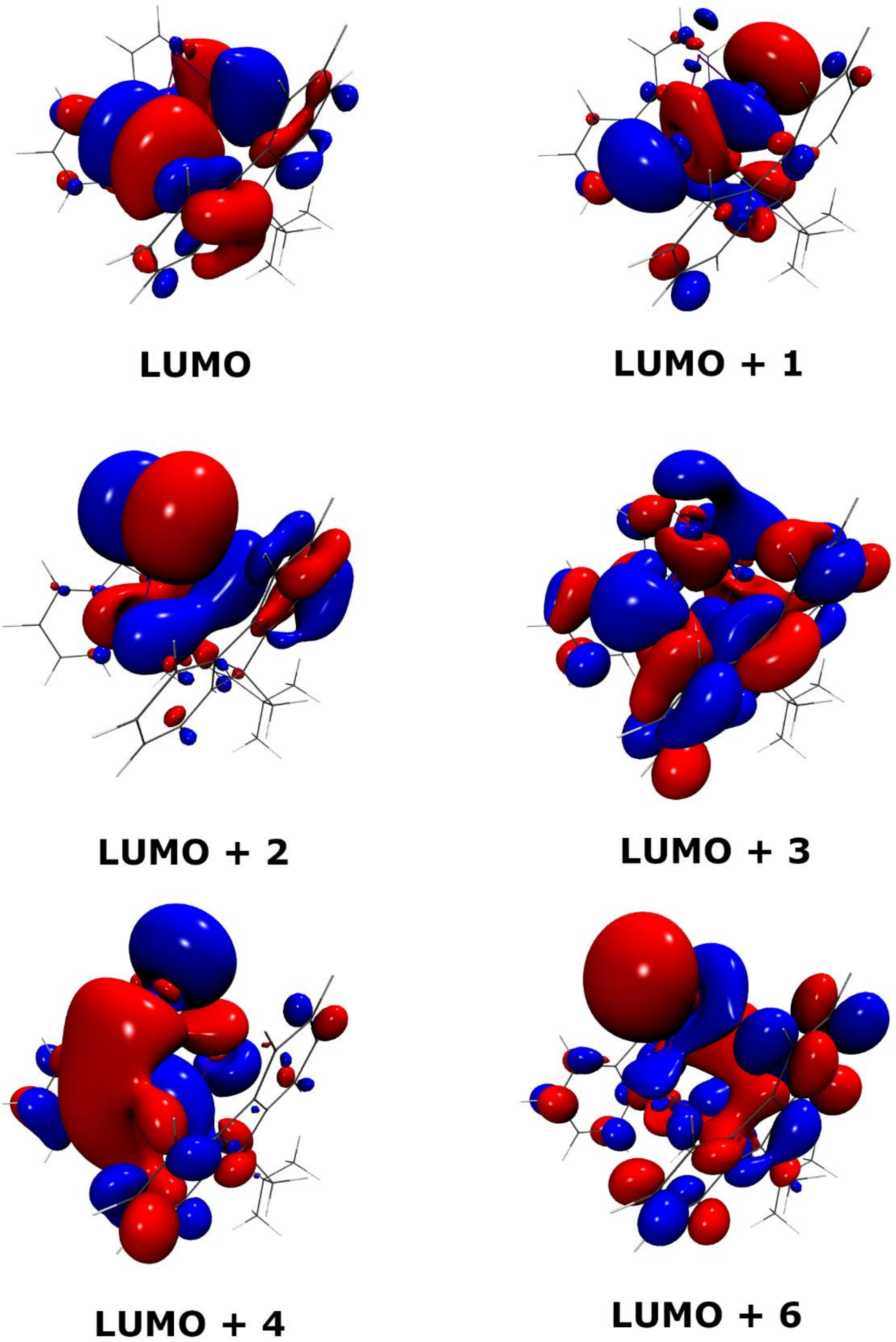

Figure S101. Lowest unoccupied molecular orbitals of $[M^{S}FluindBi_3]^{2+}$ (**7**) at *iso*-surfaces of +/– 0.02 (blue/red) representing the CBi_3 core.

Table S9. Compliance matrix for the Bi–Bi and Bi–C interactions within $[\mathrm{M^{S}FluindBi_3}]^{2+}$ (**7**) and $\mathbf{[PhBi_3]^{2+}}$. Values are in Å mdyn^{-1}.

**7**	C1–Bi74	C1–Bi75	Bi74–Bi76	Bi74–Bi76
C1–Bi74	1.156			
C1–Bi75	–0.331	1.535		
Bi74–Bi76	–0.186	0.203	0.969	
Bi75–Bi76	0.094	–0.197	–0.099	0.706
$\mathbf{[PhBi_3]}^{2+}$	C1–Bi8	C1–Bi9	Bi8–Bi10	Bi9–Bi10
C1–Bi8	1.158			
C1–Bi9	–0.368	1.159		
Bi8–Bi10	–0.120	0.089	0.891	
Bi9–Bi10	0.089	–0.120	0.018	0.891

Table S10. TD-DFT computed first 25 transitions of **7** including SO coupling.

Ex. State #	Wavelength [nm]	F	S-Weight [%]	T -Weight[%]
**0**			83.3	5.9
**1**	955.7	0.000167023		95.1
**2**	952.2	0.001236838		95.0
**3**	899.8	0.000013300	33.8	61.9
**4**	863.3	0.005011548	3.7	91.9
**5**	830.8	0.000000289	1.7	93.2
**6**	817.6	0.000140118	12.5	80.8
**7**	761.9	0.009159356	12.7	83.1
**8**	707.7	0.000044288	3.7	93.4
**9**	698.9	0.004087526	2.6	90.2
**10**	691.9	0.000018851	12.7	82.5
**11**	629	0.120571382	12.4	81.5
**12**	619.4	0.039985960	24.6	71.1
**13**	610.7	0.000011886	3.2	92.1
**14**	588.3	0.016058600	15.9	74.5
**15**	570.4	0.000125517	10.9	80.6
**16**	561.1	0.156882975	26.6	64.2
**17**	558.2	0.000192483	65.0	28.2
**18**	553.7	0.000000662	48.5	41.0
**19**	546.9	0.040783308	23.4	68.0
**20**	542.5	0.002304026	7.8	85.5
**21**	539	0.055697734	11.6	78.5
**22**	534.8	0.002215589	14.5	73.5
**23**	530.9	0.068463572	33.2	60.9
**24**	522.9	0.048202778	14.9	76.2
**25**	521.6	0.000060906	2.0	87.5

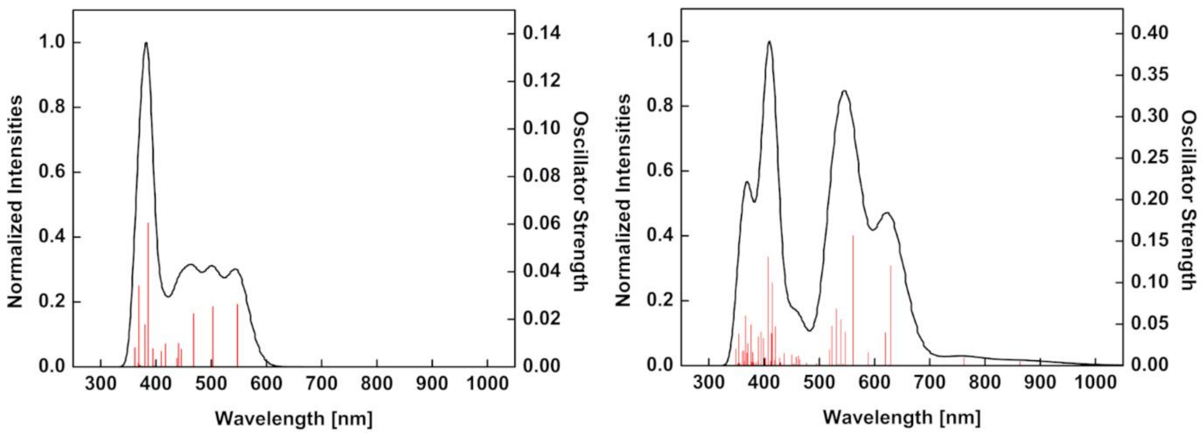

Fig. S102. Computed UV-vis absorption spectra of **7** without (left) and with the inclusion of spin orbit coupling (right). The phenomenological broadening (Gaussian) used to generate the illustrated spectra is σ = 0.2 eV.

Additional References

[S1] Ito, M.; Hashizume, D.; Fukunaga, T.; Matsuo, T.; Tamao, K, *J. Am. Chem. Soc.* **2009**, *131*, 18024-18025.

[S2] P. Romanato, S. Duttwyler, A. Linden, K. K. Baldridge, J. S. Siegel, *J. Am. Chem. Soc.* **2010**, *132*, 7828-7829

[S3] Fulmer, G. R., Miller, A.J.M., Sherden, N.H., Gottlieb, H.E., Nudelman, A., Stoltz, B.M., Bercaw, J.E., Goldberg, K.I., *Organometallics*, **2010**, *29*, 2176–2179.

[S4] Dolomanov, O. V.; Bourhis, L. J.; Gildea, R. J.; Howard, J. A. K.; Puschmann, H. *J. Appl. Crystallogr.* **2009**, *42*, 339−341.

[S5] Putz, H.; Brandenburg, K. *GbR, Diamond–Crystal and Molecular Structure Visualization*, Crystal Impact: Kreuzherrenstr. 102, 53227 Bonn, Germany, 2023.

[S6] (a) Becke, A. D. *J. Chem. Phys.*, **1993**, *98*, 5648−5652. (b) Perdew, J. P.; Chevary, J. A.; Vosko, S. H.; Jackson, K. A.; Pederson, M. R.; Singh, D. J.; Fiolhais, C. *Phys. Rev. B: Condens. Matter Mater. Phys.* **1992**, *46*, 6671−6687.

[S7] (a) Krishnan, R.; Binkley, J. S.; Seeger, R.; Pople, J. A. *J. Chem. Phys.* **1980**, *72*, 650−654. (b) McLean, A. D.; Chandler, G. S. *J. Chem. Phys.* **1980**, *72*, 5639−5648.

[S8] Gaussian16 (RevisionC.01), Frisch, M. J.; Trucks, G. W.; Schlegel, H. B.; Scuseria, G. E.; Robb, M. A.; Cheeseman, J. R.; Scalmani, G.; Barone, V.; Petersson, G. A.; Nakatsuji, H.; Li, X.; Caricato, M.; Marenich, A. V.; Bloino, J.; Janesko, B. G.; Gomperts, R.; Mennucci, B.; Hratchian, H. P.; Ortiz, J. V.; Izmaylov, A. F.; Sonnenberg, J. L.; Williams-Young, D.; Ding, F.; Lipparini, F.; Egidi, F.; Goings, J.; Peng, B.; Petrone, A.; Henderson, T.; Ranasinghe, D.; Zakrzewski, V. G.; Gao, J.; Rega, N.; Zheng, G.; Liang, W.; Hada, M.; Ehara, M.; Toyota, K.; Fukuda, R.; Hasegawa, J.; Ishida, M.: Nakajima, T.; Honda, Y.; Kitao, O.; Nakai, H.; Vreven, T.; Throssell, K.; Montgomery Jr., J. A.; Peralta, J. E.; Ogliaro, F.; Bearpark, M. J.; Heyd, J. J.; Brothers, E. N.; Kudin, K. N.; Staroverov, V. N.; Keith, T. A.; Kobayashi, R.; Normand, J.; Raghavachari, K.; Rendell, A. P.; Burant, J. C.; Iyengar, S. S.; Tomasi, J.; Cossi, M.; Millam, J. M.; Klene, M.; Adamo, C.; Cammi, R.; Ochterski, J. W.; Martin, R. L.; Morokuma, K.; Farkas, O.; Foresman J. B.; Fox, D. J. Gaussian, Inc., Wallingford CT **2019**.

[S9] (a) Metz, B.; Stoll, H.; Dolg, M. *J. Chem. Phys.* **2000**, *113*, 2563-2569. (b) Peterson, K. A. *J. Chem. Phys.* **2003**, *119*, 11099−11112. (c) Peterson, K. A.; Figgen, D.; Goll, E.; Stoll, H.; Dolg, M. *J. Chem. Phys.* **2003**, *119*, 11113-11123.

[S10] (a) Feller, D. *J. Comput. Chem.* **1996**, *17*, 1571−1586. (b) Schuchardt, K. L.; Didier, B. T.; Elsethagen, T.; Sun, L.; Gurumoorthi, V.; Chase, J.; Li, J.; Windus, T. L. *J. Chem. Inf. Model.* **2007**, *47*, 1045−1052. (c) Pritchard, B. P.; Altarawy, D.; Didier, B.; Gibson, T. D.; Windus, T. L. *J. Chem. Inf. Model.* **2019**, *59*, 4814-4820.

[S11] Grimme, S.; Ehrlich, S.; Goerigk, L. *J. Comp. Chem.*, **2011**, *32*, 1456-1465.

[S12] *AIMAll* (Version 15.09.27), Keith, T. A.; TK Gristmill Software, Overland Park KS, USA, 2015 (aim.tkgristmill.com)

[S13] Contreras-García, J.; Johnson, E.; Keinan, S.; Chaudret, R.; Piquemal, J.-P.; Beratan, D.; Yang, W. *J. Chem. Theory Comput.*, **2011**, *7*, 625-632.

[S14] *NBO 6.0.*, Glendening, E. D.; Badenhoop, J. K.; Reed, A. E.; Carpenter, J. E.; Bohmann, J. A.; Morales, C. M.; Landis, C. R.; Weinhold, F. Theoretical Chemistry Institute, University of Wisconsin, Madison, 2013.

[S15] T. Lu and F. Chen, *J. Comput. Chem.*, **2012**, *33*, 580−592.

[S16] Pipik, J.; Mezey, P. G. *J. Chem. Phys.* **1989**, *90*, 4916-4926.

[S17] Neese, F. *Wiley Interdiscip. Rev. Comput. Mol. Sci.* **2012**, *2*, 73–78. (b) for Version 6.0 and above also: Neese, F. *Wiley Interdiscip. Rev. Comput. Mol. Sci.* **2025**, *15*, e70019. (c) Neese, F.; Wennmohs, F.; Becker, U.; Riplinger, C. *J. Chem. Phys.* **2020**, *152, Art.Nr. L224108*

[S18] Weigend, F.; Ahlrichs, R. *Phys. Chem. Chem. Phys.* **2005**, *7*, 3297–3305.

[S19] Humphrey, W.; Dalke, A.; Schulten, K. *J. Mol. Graph.*, **1996**, *14*, 33-38.

[S20] Brandhorst, K.; Grunenberg, J. *Chem. Soc. Rev.* **2008**, *37*, 1558–1567.

[S21] (a) Kutzelnigg, W.; Liu, W. *J. Chem. Phys.* **2005**, *123*, 241102. (b) Liu, W. *Mol. Phys.* **2010**, *108*, 1679-1706. (c) Saue, T. *ChemPhysChem* **2011**, *12*, 3077-3094.

[S22] (a) Pollack, P.; Weigend, F. *J. Chem. Theory Comput.* **2017**, *13*, 3696-3705. (b) Franzke, Y. J.; Treß, R.; Pazedera, T. M.; Weigend, F. *Phys. Chem. Chem. Phys.* **2019**, *21*, 16658-16664.
